# Supplementary figures and images for: A remarkable adaptive paradigm of heart performance and protection emerges in response to marked cardiac-specific overexpression of ADCY8 (part 1 of 3)
Source: eLife. 2022 Dec 14;11:e80949. doi: 10.7554/eLife.80949 (PMC9822292; doi:10.7554/eLife.80949)

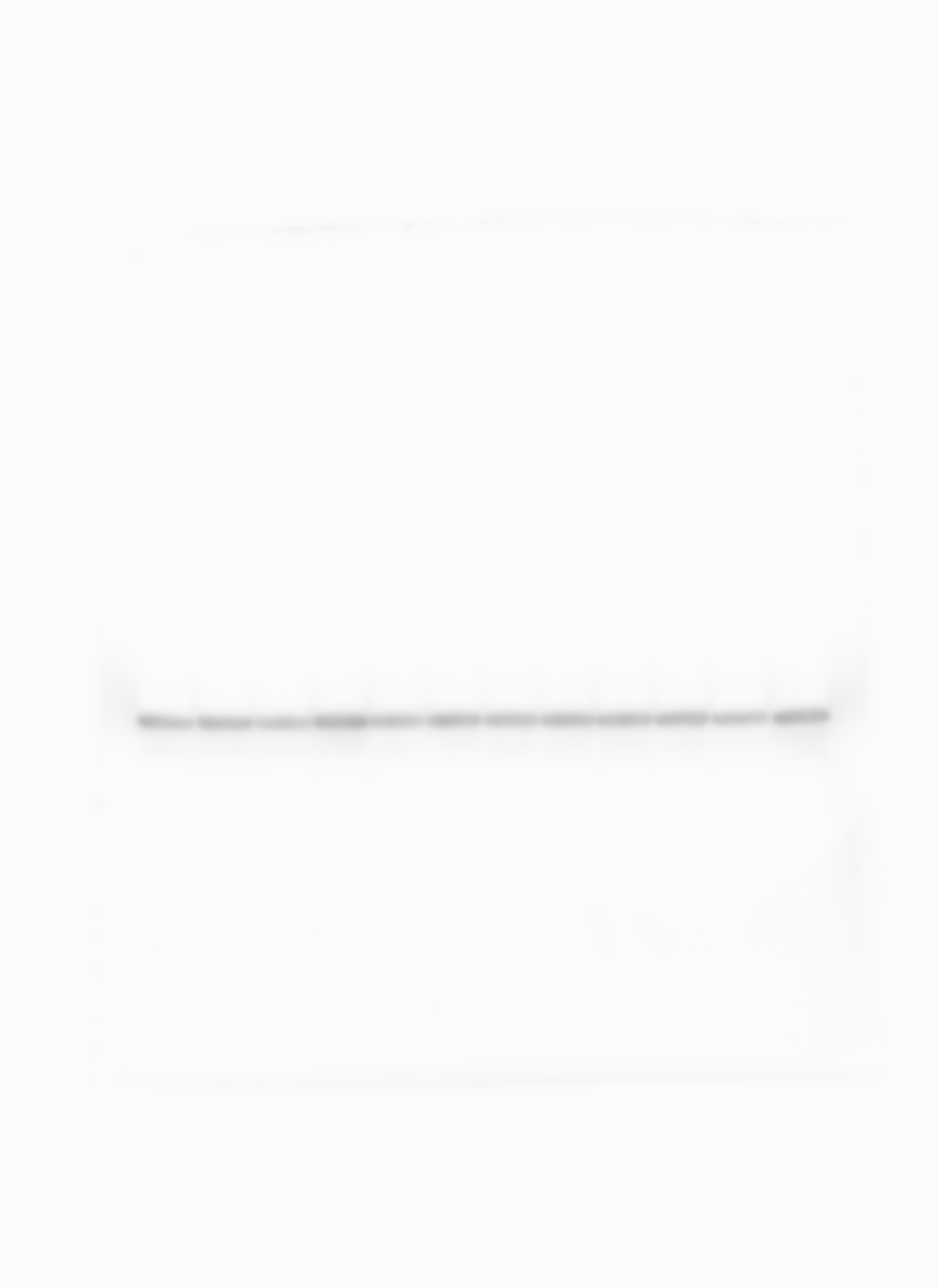

Supplement: Figure 2—source data 1. — Actin, ANP, BNP, and Calcineurin. [file elife-80949-fig2-data1.zip › Figure 2/alpha Skeletal Actin/alpha Skeletal Actin/DR aSK. Actin Blt52 2020.02.27_14.12.58_Ch.tif]

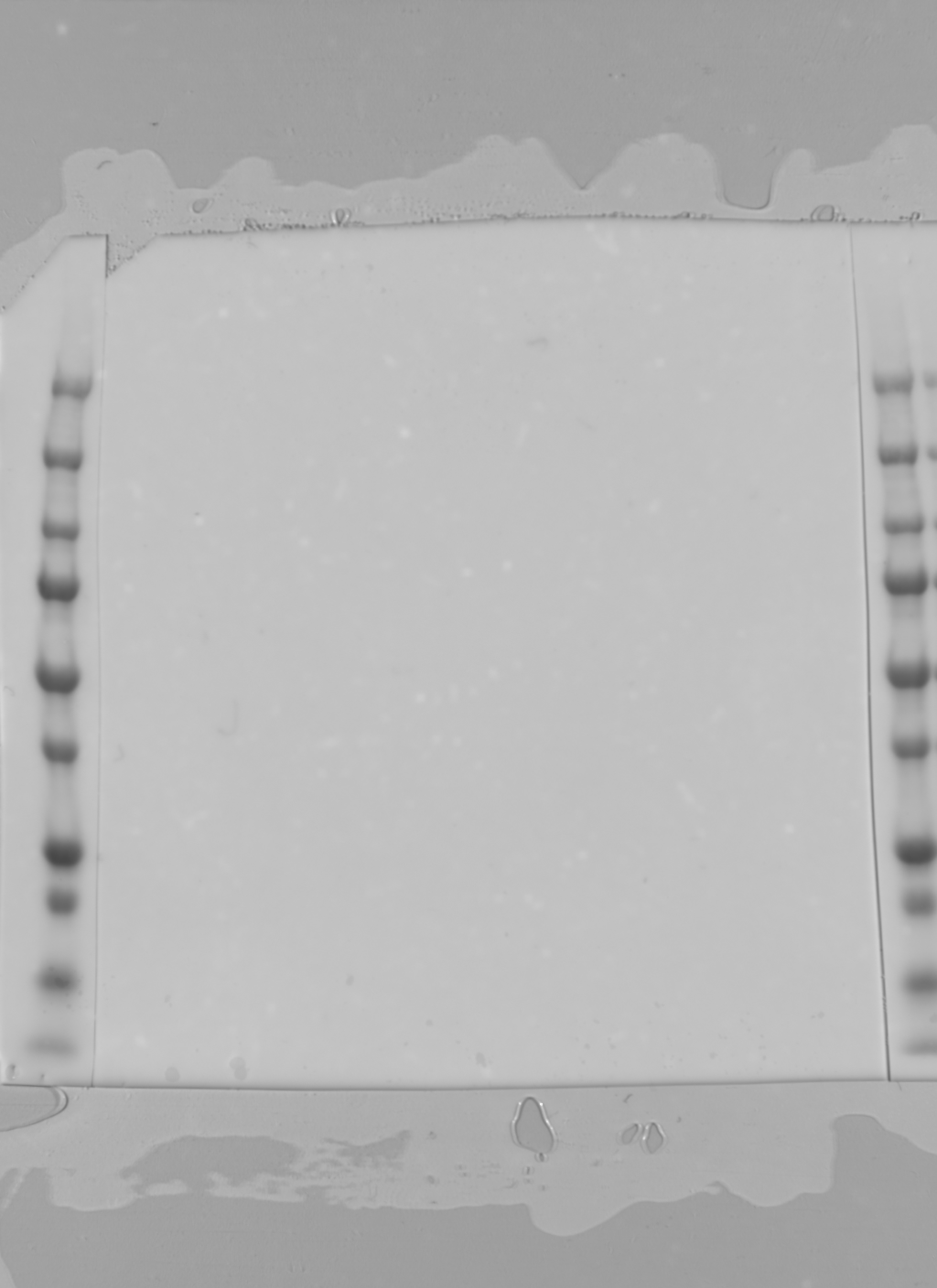

Supplement: Figure 2—source data 1. — Actin, ANP, BNP, and Calcineurin. [file elife-80949-fig2-data1.zip › Figure 2/alpha Skeletal Actin/alpha Skeletal Actin/DR aSK. Actin Blt52 2020.02.27_14.12.58_Ch-Marker.tif]

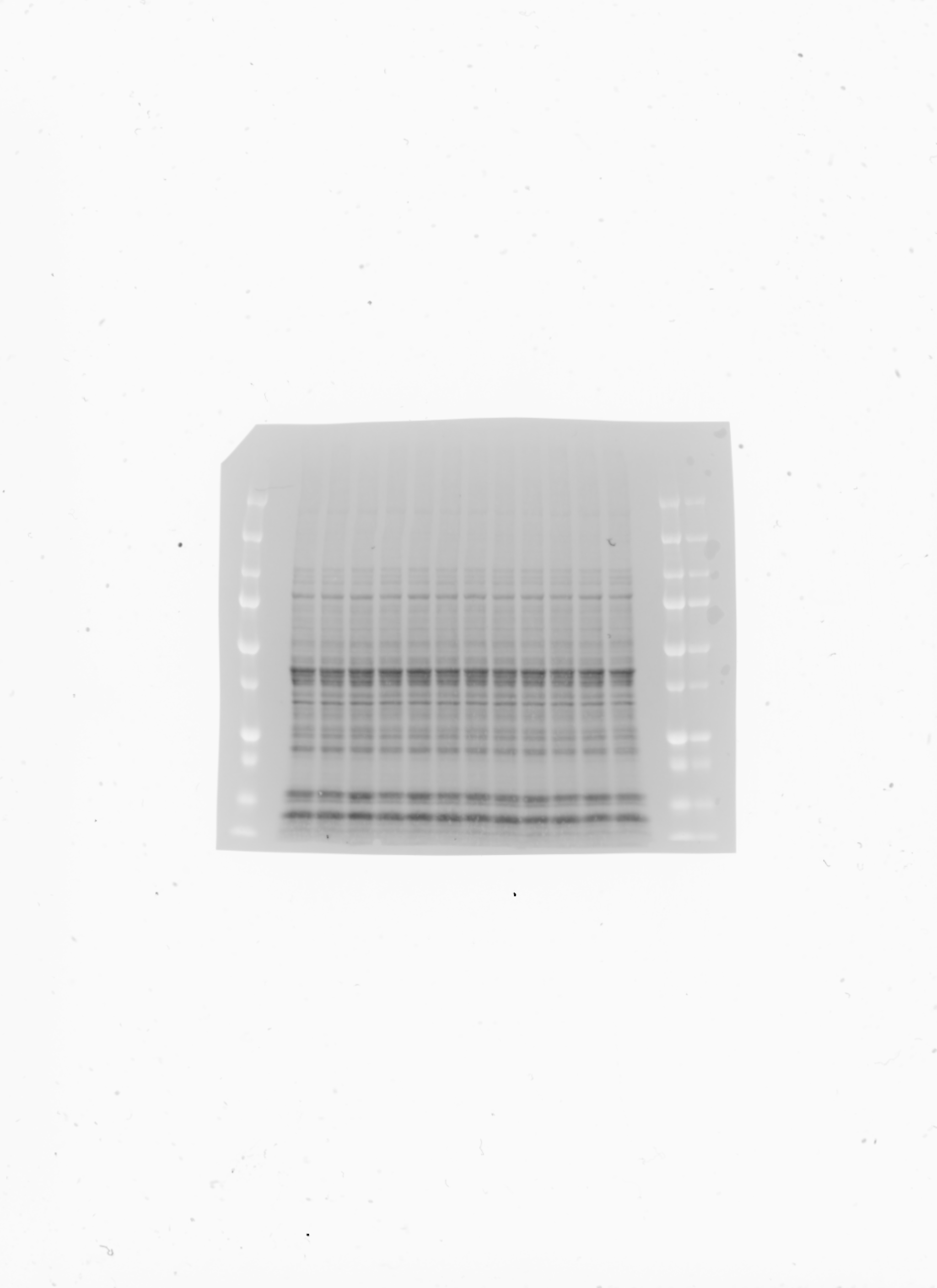

Supplement: Figure 2—source data 1. — Actin, ANP, BNP, and Calcineurin. [file elife-80949-fig2-data1.zip › Figure 2/alpha Skeletal Actin/Total Protein/DR T.Prot Blt 52 2020.01.08_13.32.46_Fl-UV.tif]

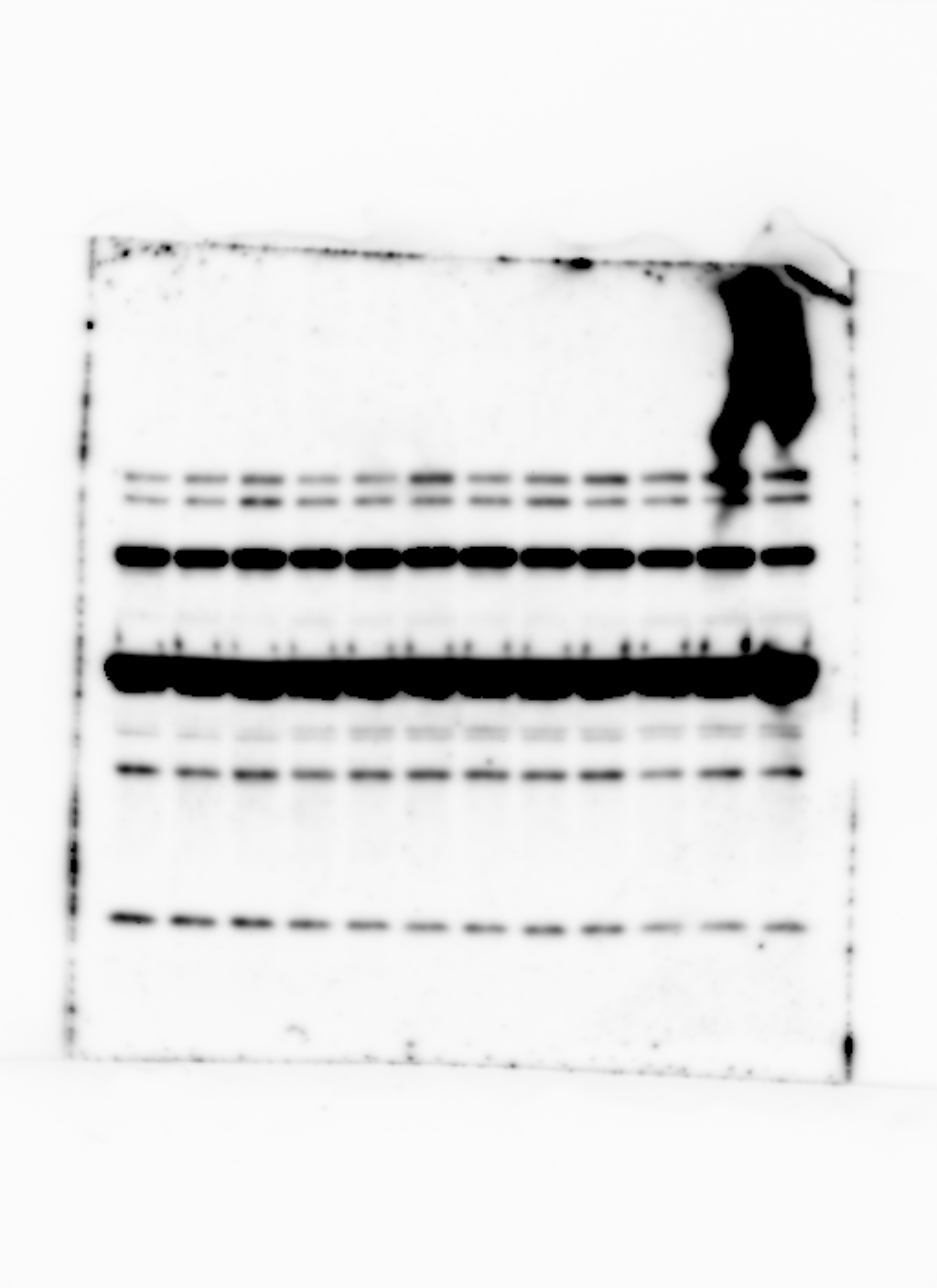

Supplement: Figure 2—source data 1. — Actin, ANP, BNP, and Calcineurin. [file elife-80949-fig2-data1.zip › Figure 2/ANP/ANP/DR ANP LV Blot53 2020.01.09_12.50.25_Ch.tif]

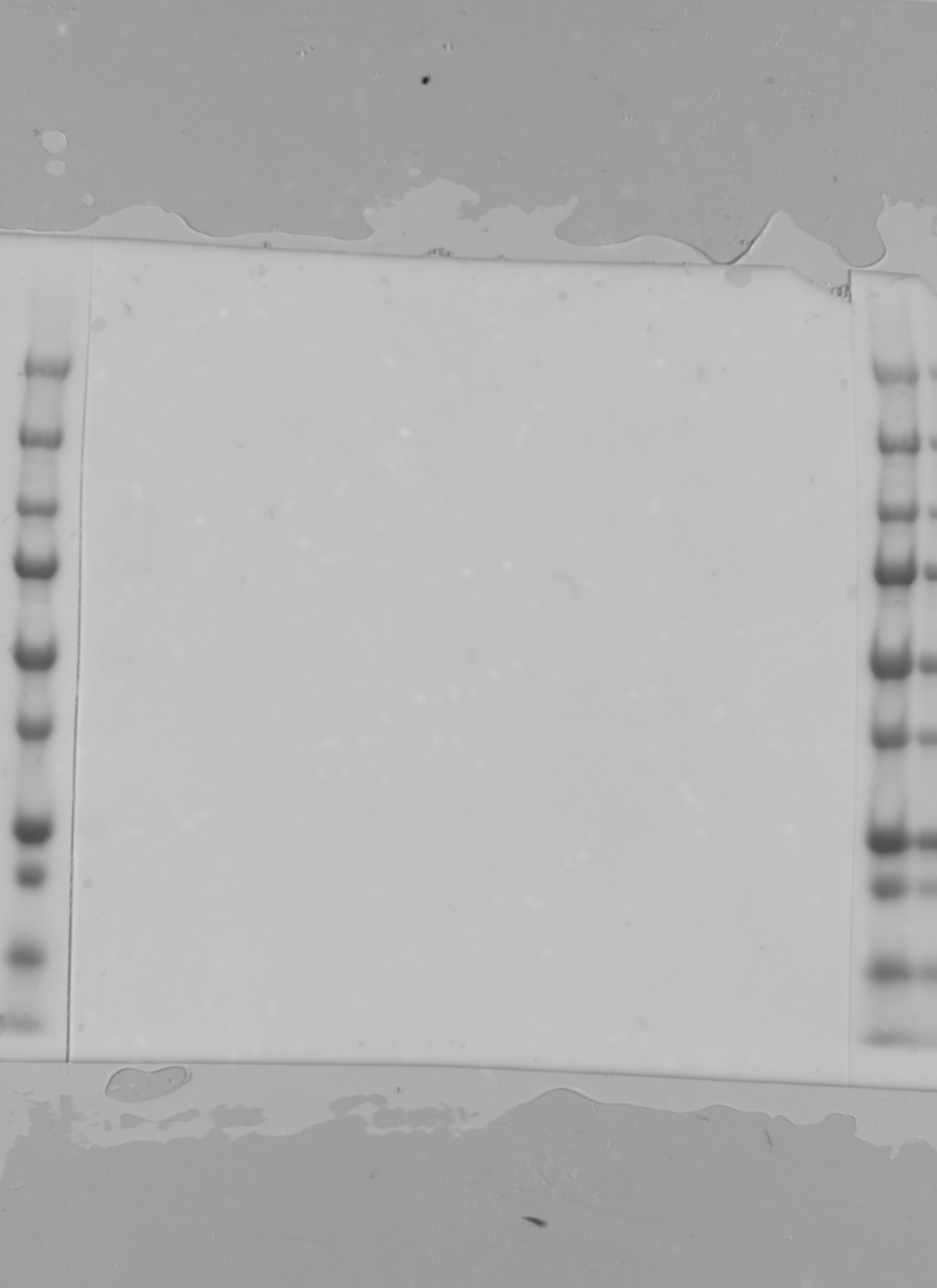

Supplement: Figure 2—source data 1. — Actin, ANP, BNP, and Calcineurin. [file elife-80949-fig2-data1.zip › Figure 2/ANP/ANP/DR ANP LV Blot53 2020.01.09_12.50.25_Ch-Marker.tif]

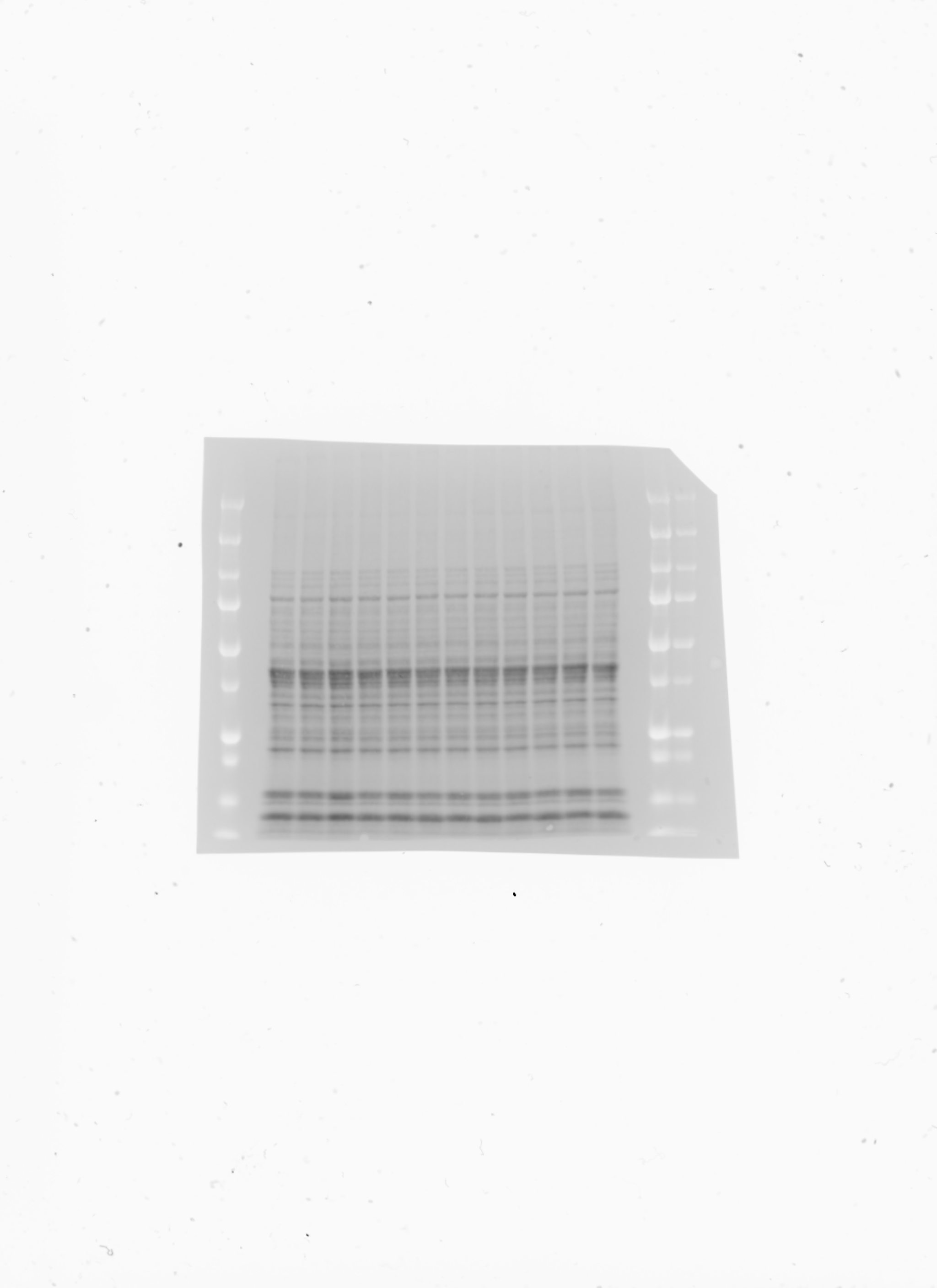

Supplement: Figure 2—source data 1. — Actin, ANP, BNP, and Calcineurin. [file elife-80949-fig2-data1.zip › Figure 2/ANP/Total Protein/DR T.Prot Blt 53 2020.01.08_13.35.13_Fl-UV.tif]

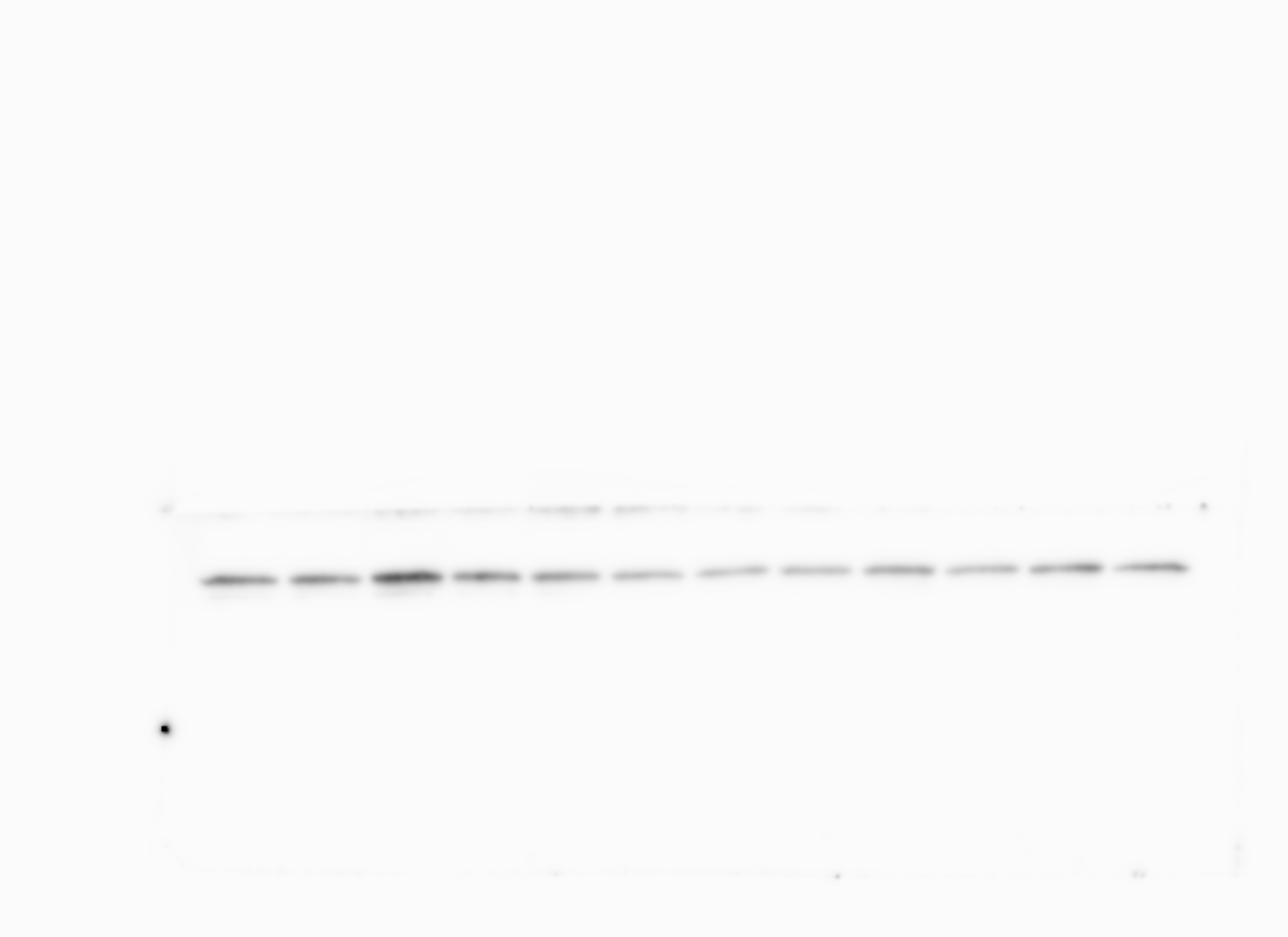

Supplement: Figure 2—source data 1. — Actin, ANP, BNP, and Calcineurin. [file elife-80949-fig2-data1.zip › Figure 2/BNP/BNP/DR BNP Blt60 2020.02.27_13.27.00_Ch.tif]

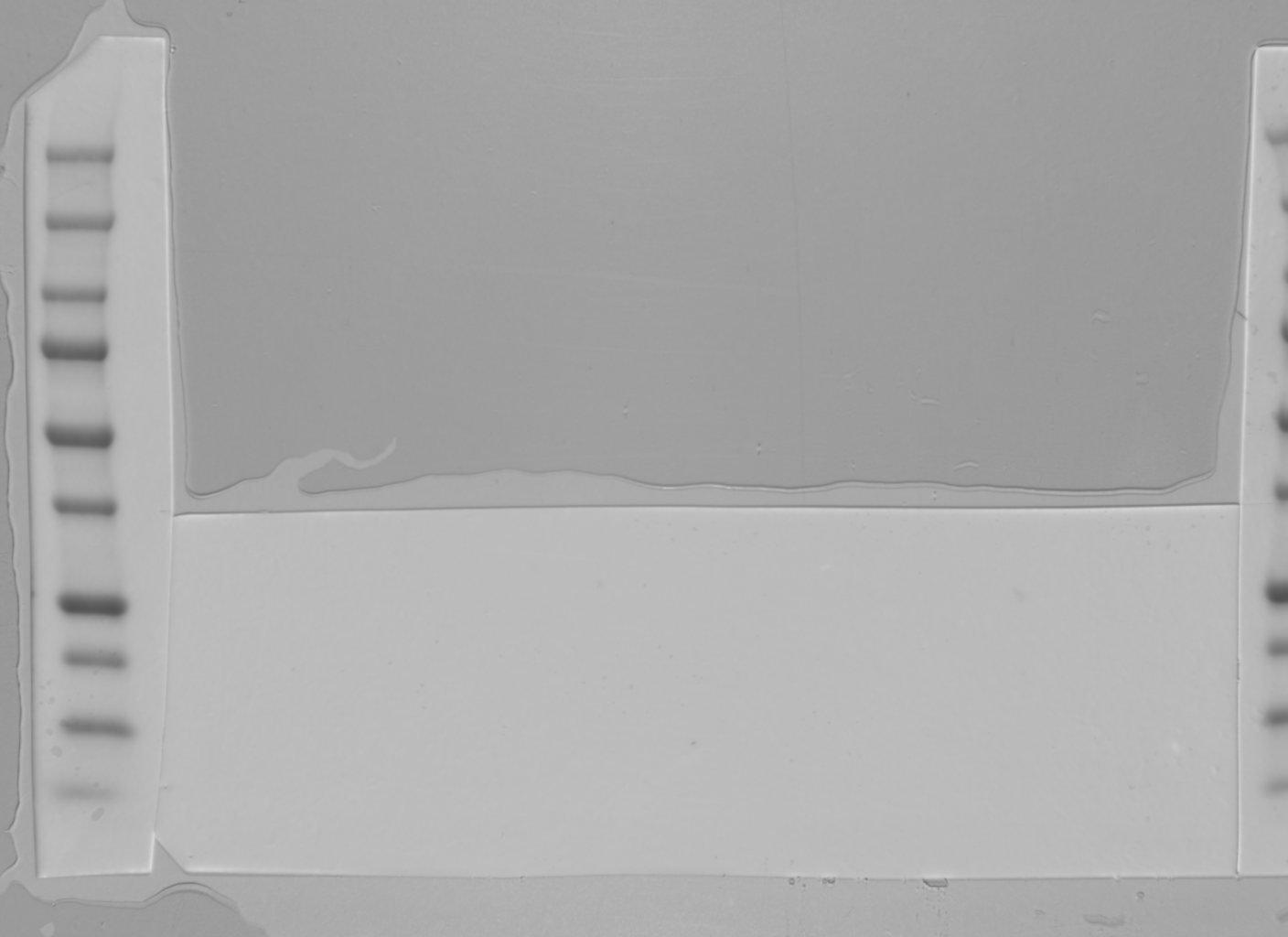

Supplement: Figure 2—source data 1. — Actin, ANP, BNP, and Calcineurin. [file elife-80949-fig2-data1.zip › Figure 2/BNP/BNP/DR BNP Blt60 2020.02.27_13.27.00_Ch-Marker.tif]

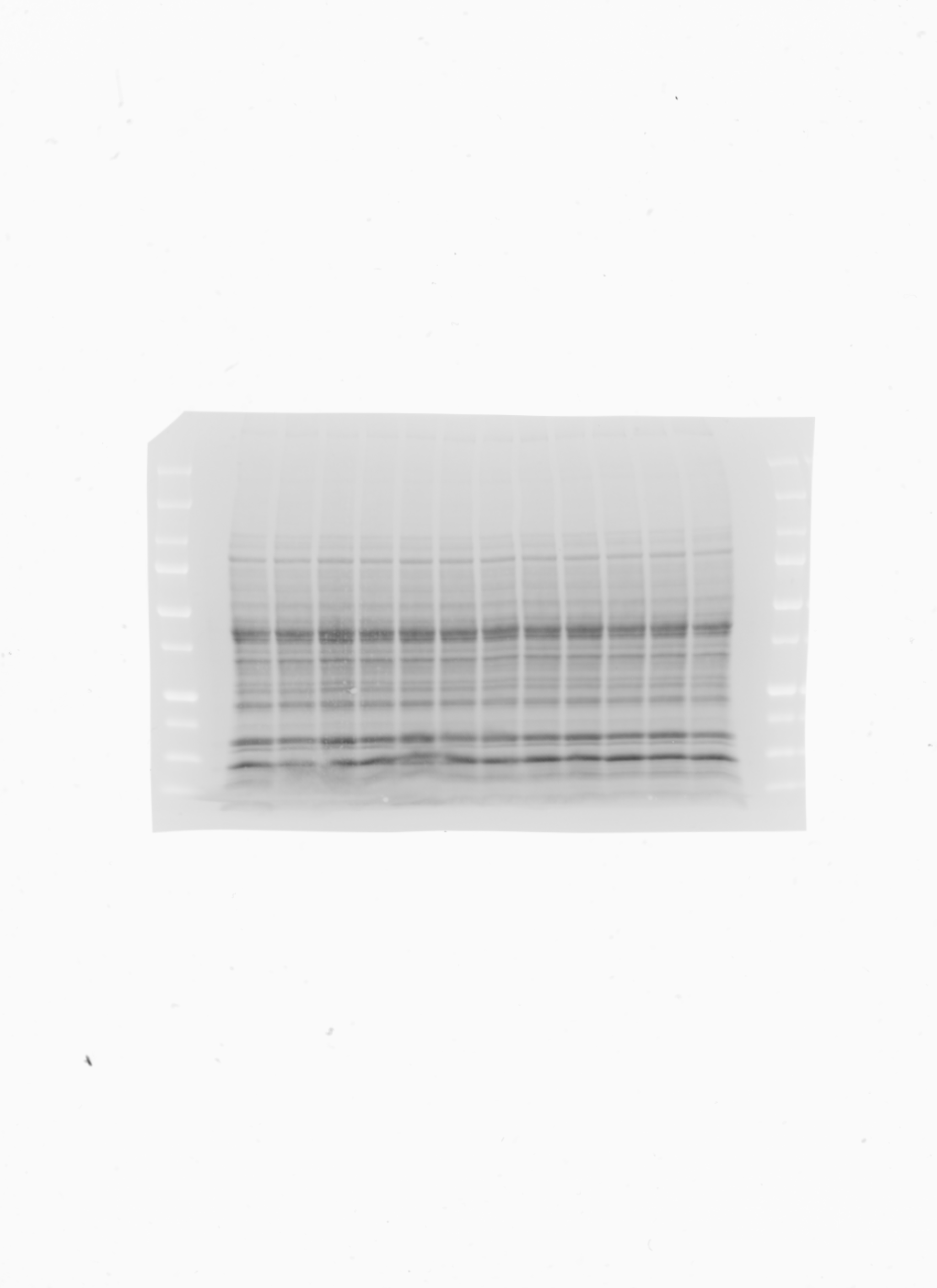

Supplement: Figure 2—source data 1. — Actin, ANP, BNP, and Calcineurin. [file elife-80949-fig2-data1.zip › Figure 2/BNP/Total Protein/DR T.Prot Blt60 2020.02.20_14.19.34_Fl-UV.tif]

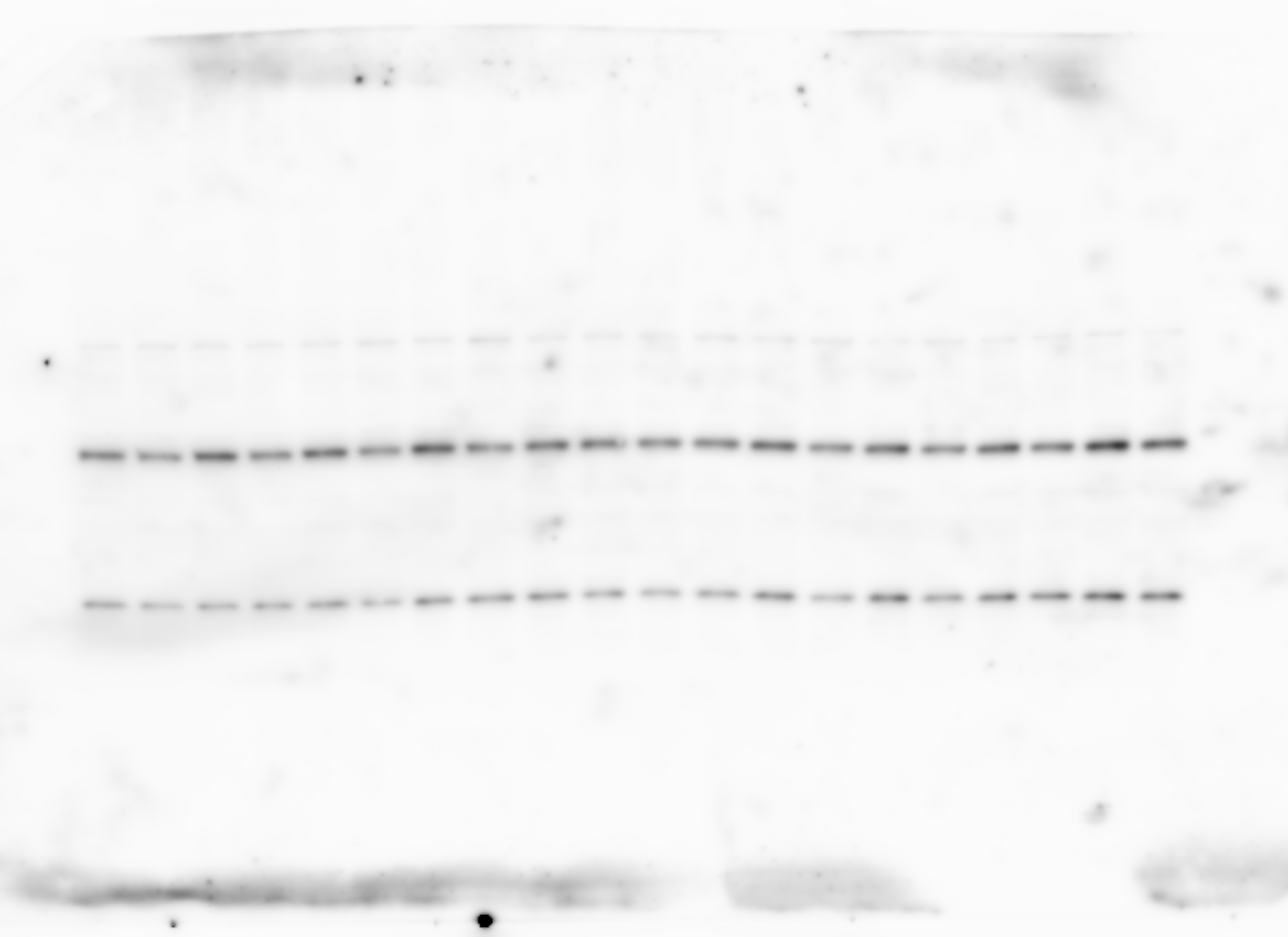

Supplement: Figure 2—source data 1. — Actin, ANP, BNP, and Calcineurin. [file elife-80949-fig2-data1.zip › Figure 2/Calcineurin/Calcineurin/DR PP2Ba Blt19 WPP 2018.04.24_10.46.06_Ch.tif]

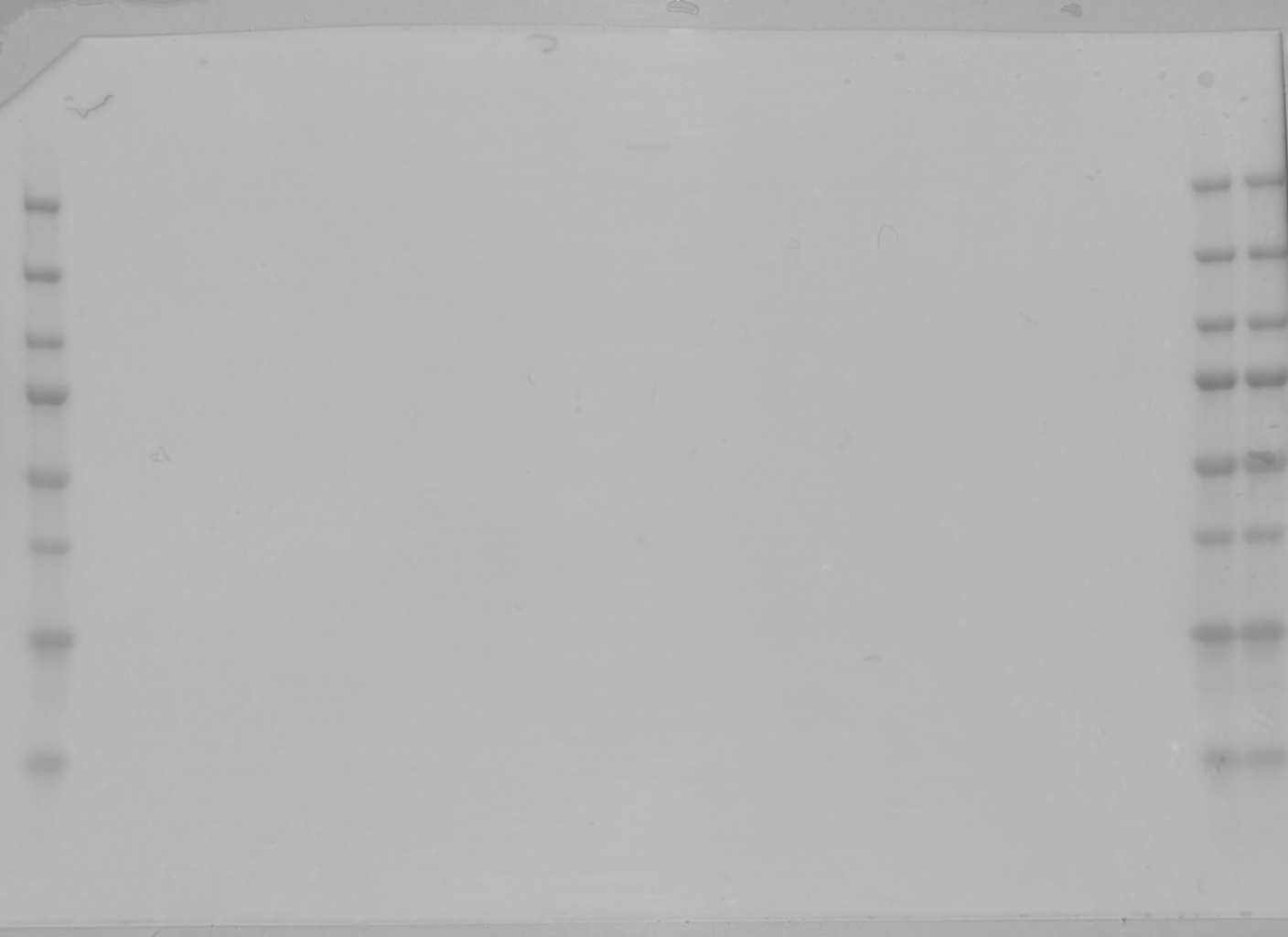

Supplement: Figure 2—source data 1. — Actin, ANP, BNP, and Calcineurin. [file elife-80949-fig2-data1.zip › Figure 2/Calcineurin/Calcineurin/DR PP2Ba Blt19 WPP 2018.04.24_10.46.06_Ch-Marker.tif]

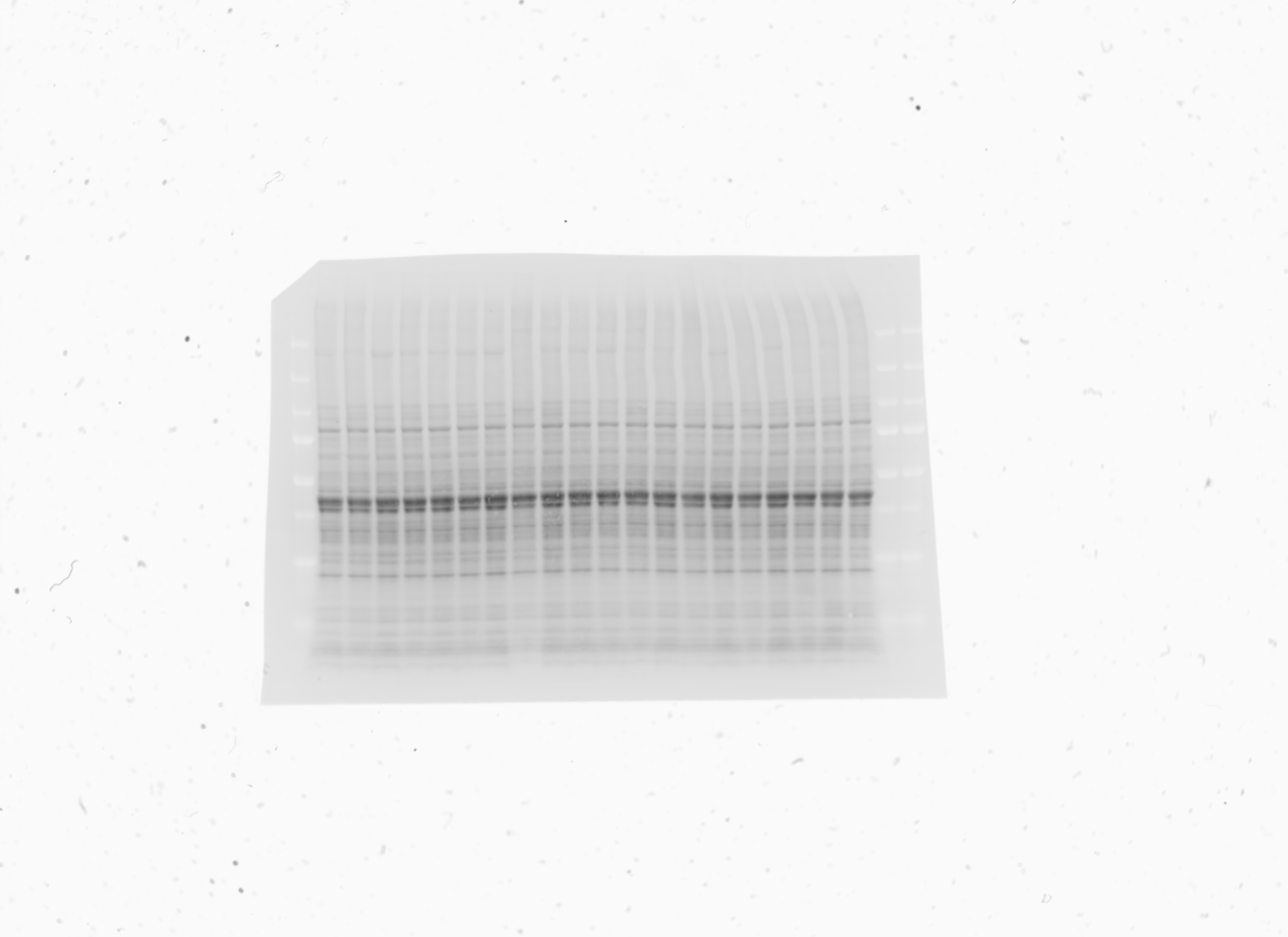

Supplement: Figure 2—source data 1. — Actin, ANP, BNP, and Calcineurin. [file elife-80949-fig2-data1.zip › Figure 2/Calcineurin/Total Protein/DR T.Prot. LV Blt 19 2018.04.19_13.48.44_Fl-UV.tif]

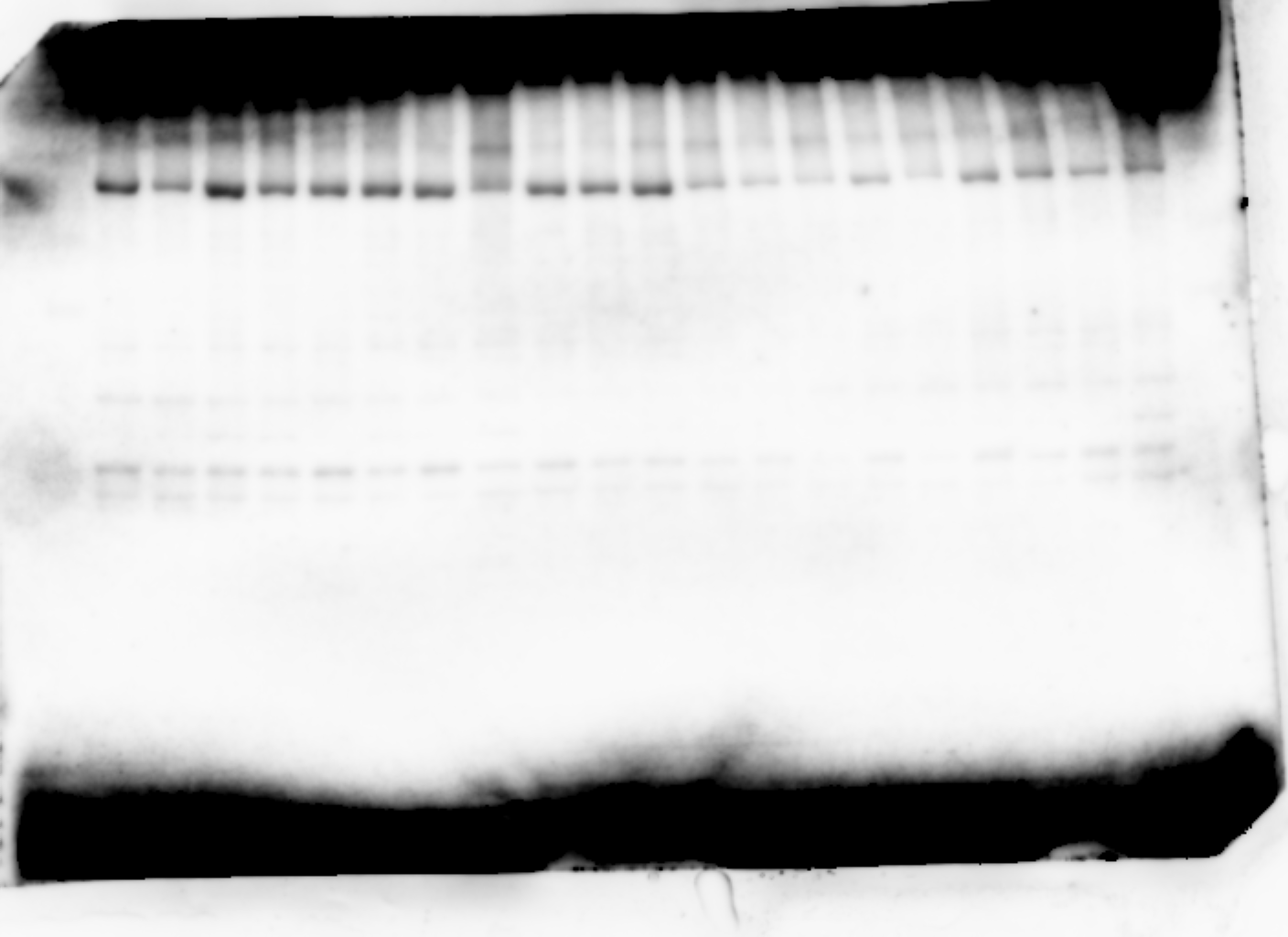

Supplement: Figure 2—source data 1. — Actin, ANP, BNP, and Calcineurin. [file elife-80949-fig2-data1.zip › Figure 2/MYH7/MYH7/DR MYH7 Blot46 2019.03.14_10.53.38_Ch.tif]

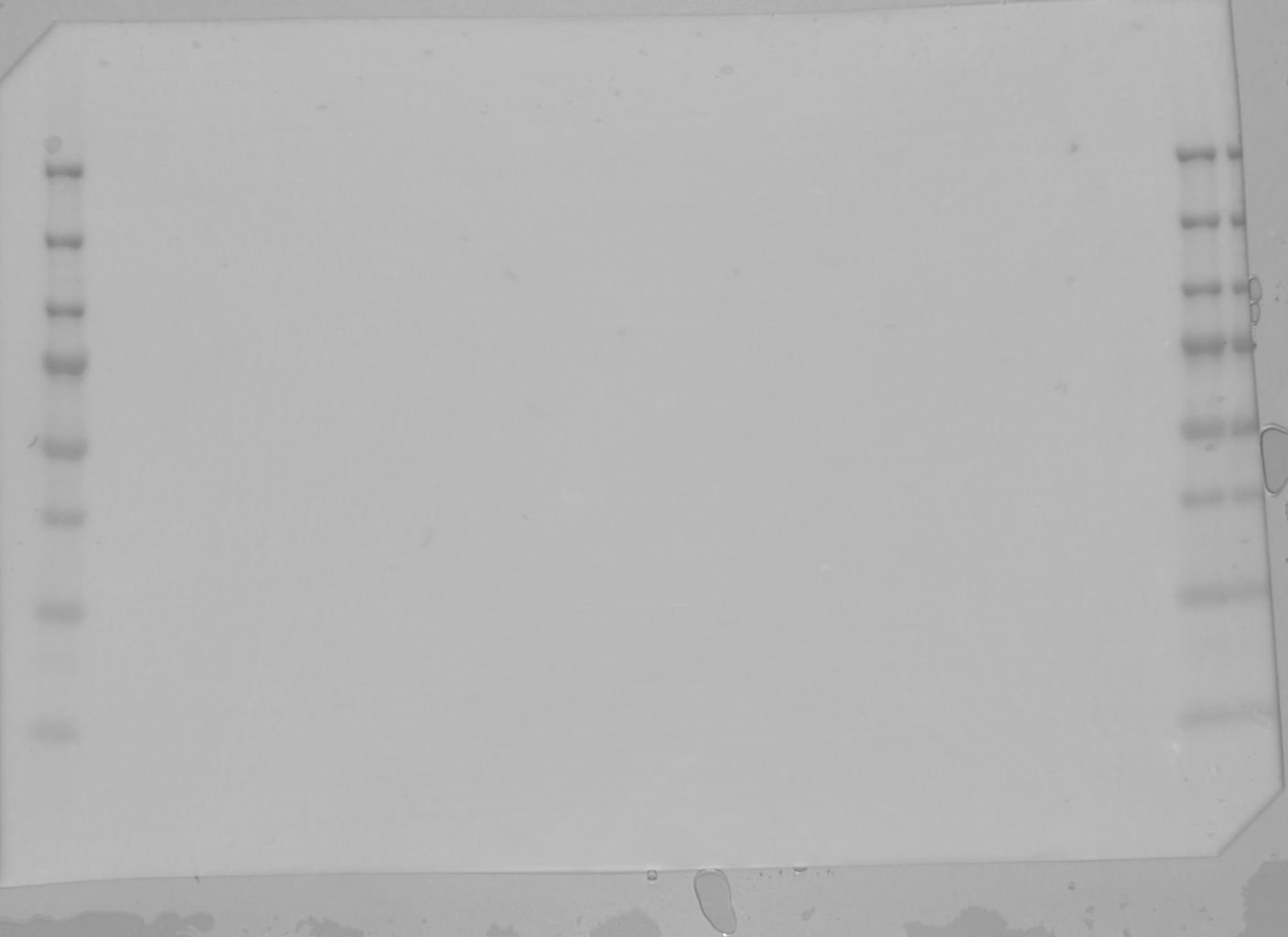

Supplement: Figure 2—source data 1. — Actin, ANP, BNP, and Calcineurin. [file elife-80949-fig2-data1.zip › Figure 2/MYH7/MYH7/DR MYH7 Blot46 2019.03.14_10.53.38_Ch-Marker.tif]

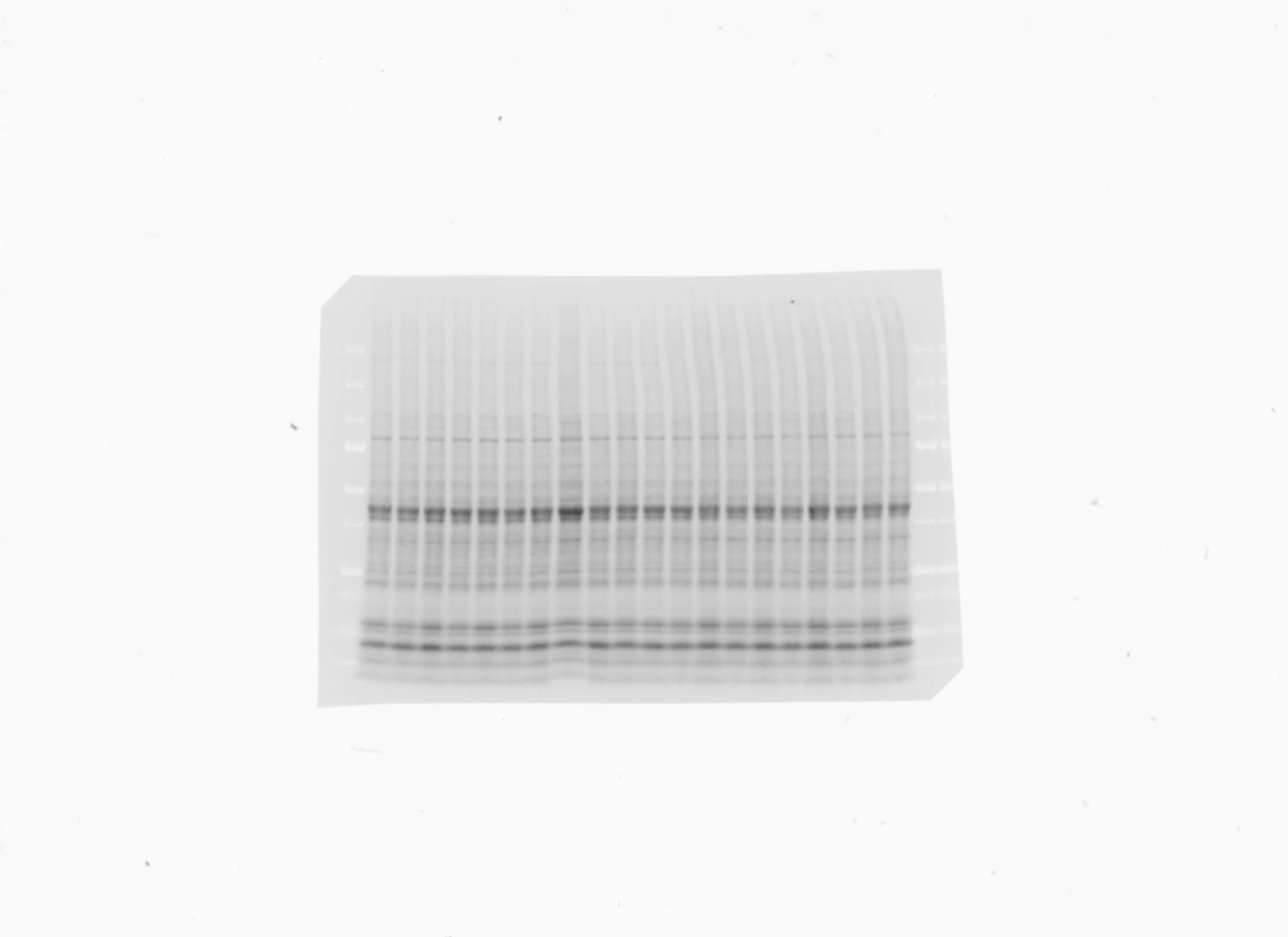

Supplement: Figure 2—source data 1. — Actin, ANP, BNP, and Calcineurin. [file elife-80949-fig2-data1.zip › Figure 2/MYH7/Total Protein/DR T.Prot LV blot46 2019.02.06_12.10.30_Fl-UV.tif]

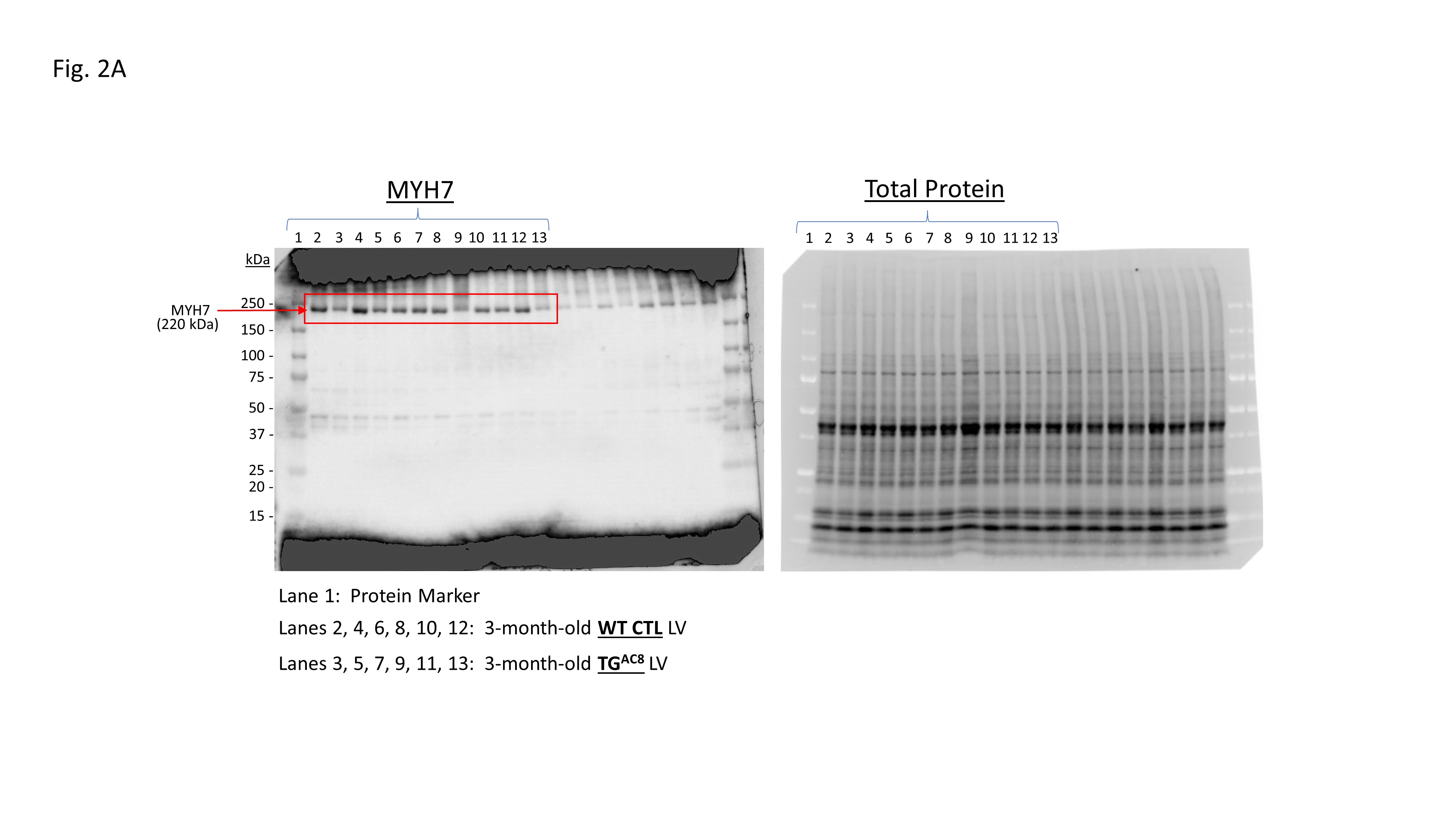

Supplement: Figure 2—source data 1. — Actin, ANP, BNP, and Calcineurin. [file elife-80949-fig2-data1.zip › Figure 2/Uncropped WB Images Fig2/Slide1.JPG]

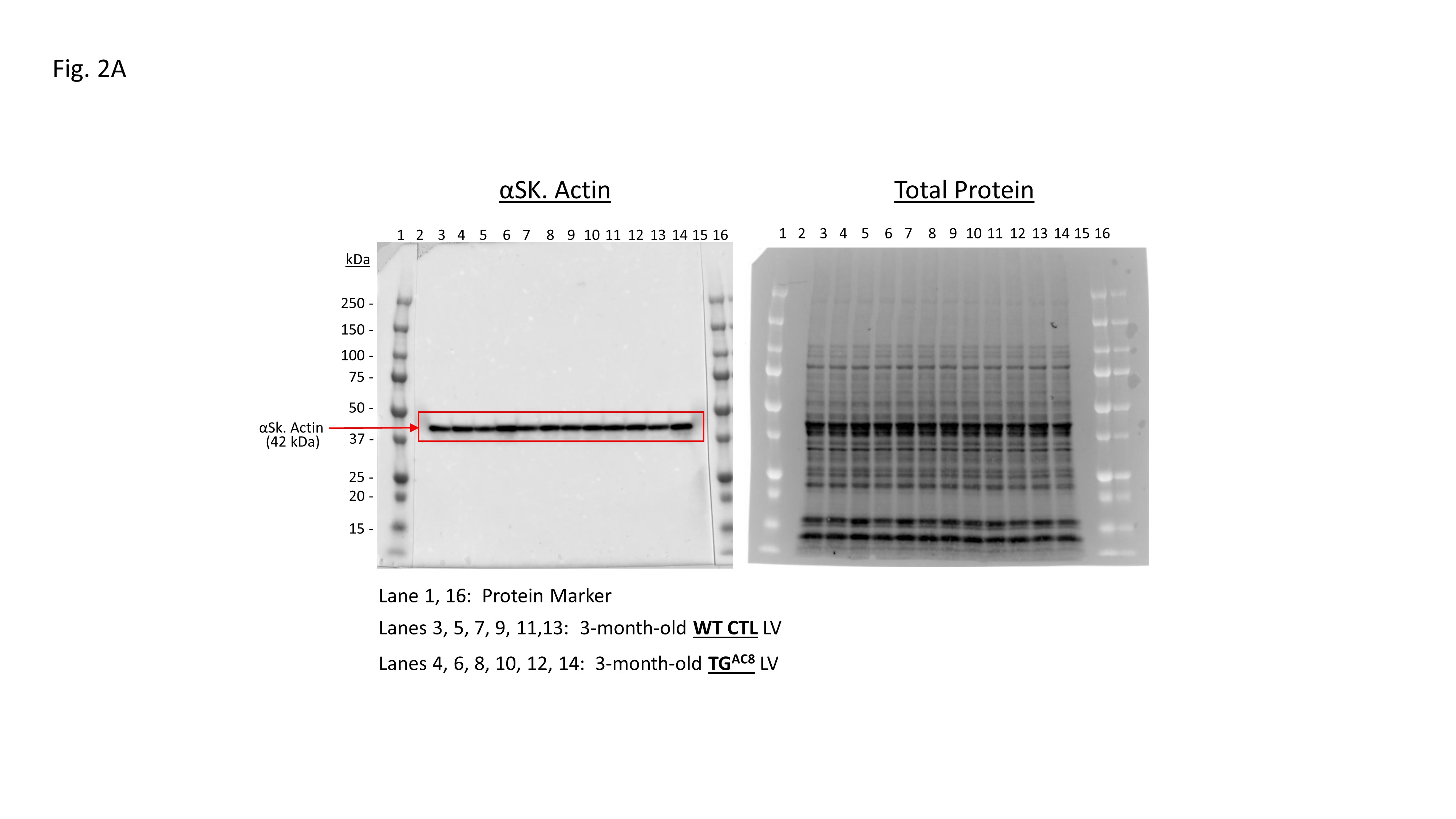

Supplement: Figure 2—source data 1. — Actin, ANP, BNP, and Calcineurin. [file elife-80949-fig2-data1.zip › Figure 2/Uncropped WB Images Fig2/Slide2.JPG]

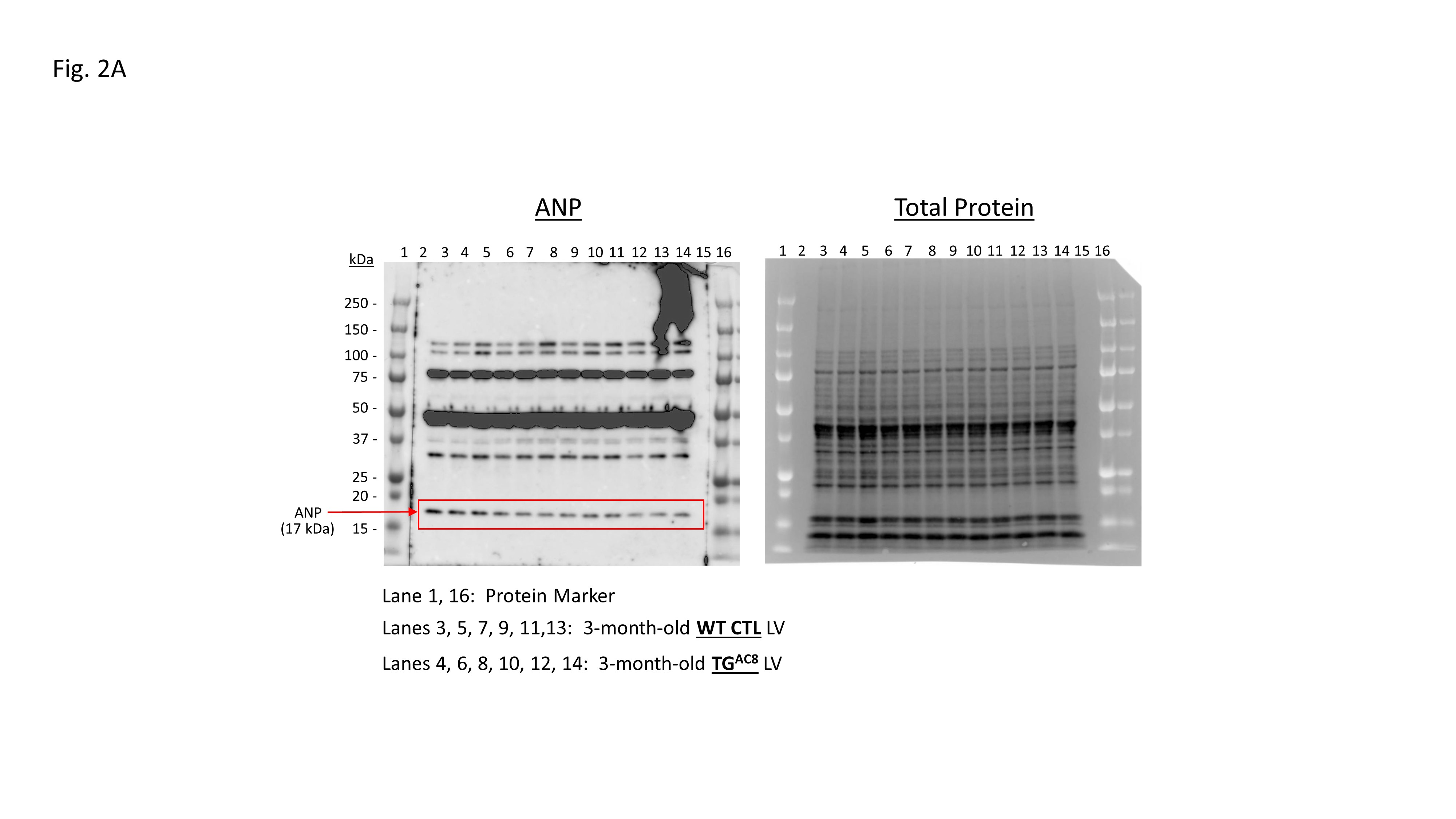

Supplement: Figure 2—source data 1. — Actin, ANP, BNP, and Calcineurin. [file elife-80949-fig2-data1.zip › Figure 2/Uncropped WB Images Fig2/Slide3.JPG]

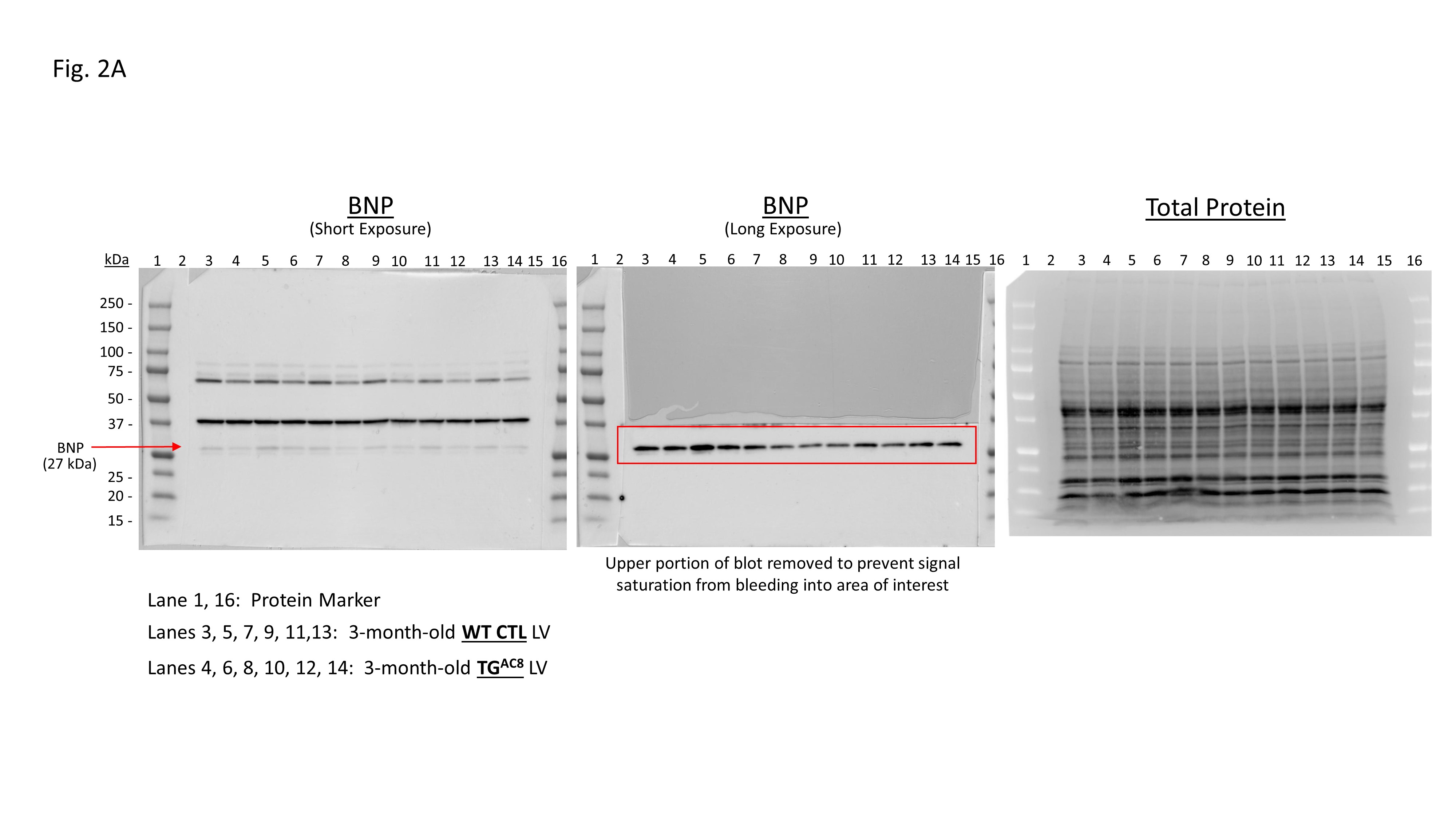

Supplement: Figure 2—source data 1. — Actin, ANP, BNP, and Calcineurin. [file elife-80949-fig2-data1.zip › Figure 2/Uncropped WB Images Fig2/Slide4.JPG]

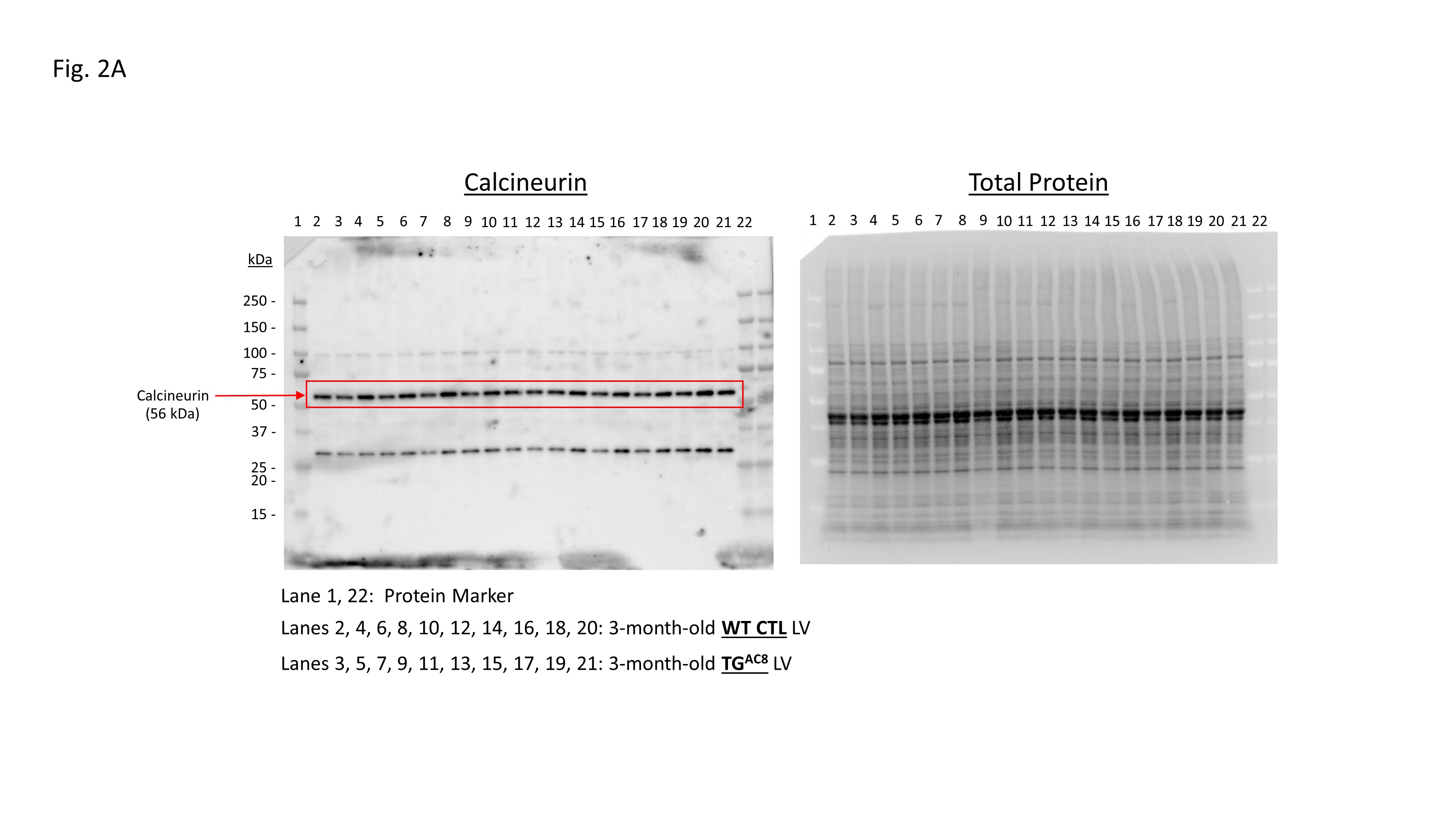

Supplement: Figure 2—source data 1. — Actin, ANP, BNP, and Calcineurin. [file elife-80949-fig2-data1.zip › Figure 2/Uncropped WB Images Fig2/Slide5.JPG]

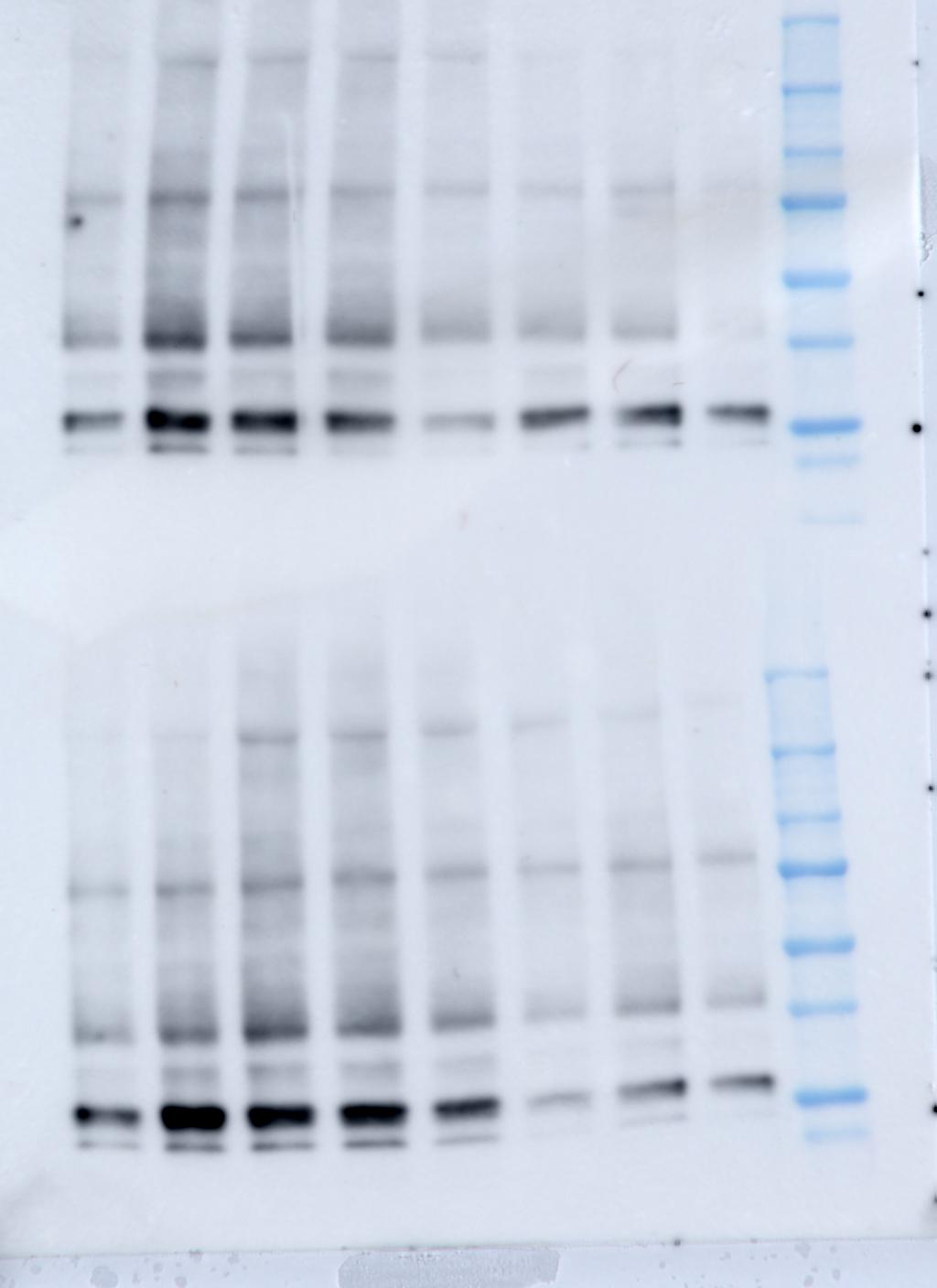

Supplement: Figure 4—source data 1. [file elife-80949-fig4-data1.zip › Figure 4/CamKII/CamKII 5min 2018.07.25_11.40.25_Ch+Marker.jpg]

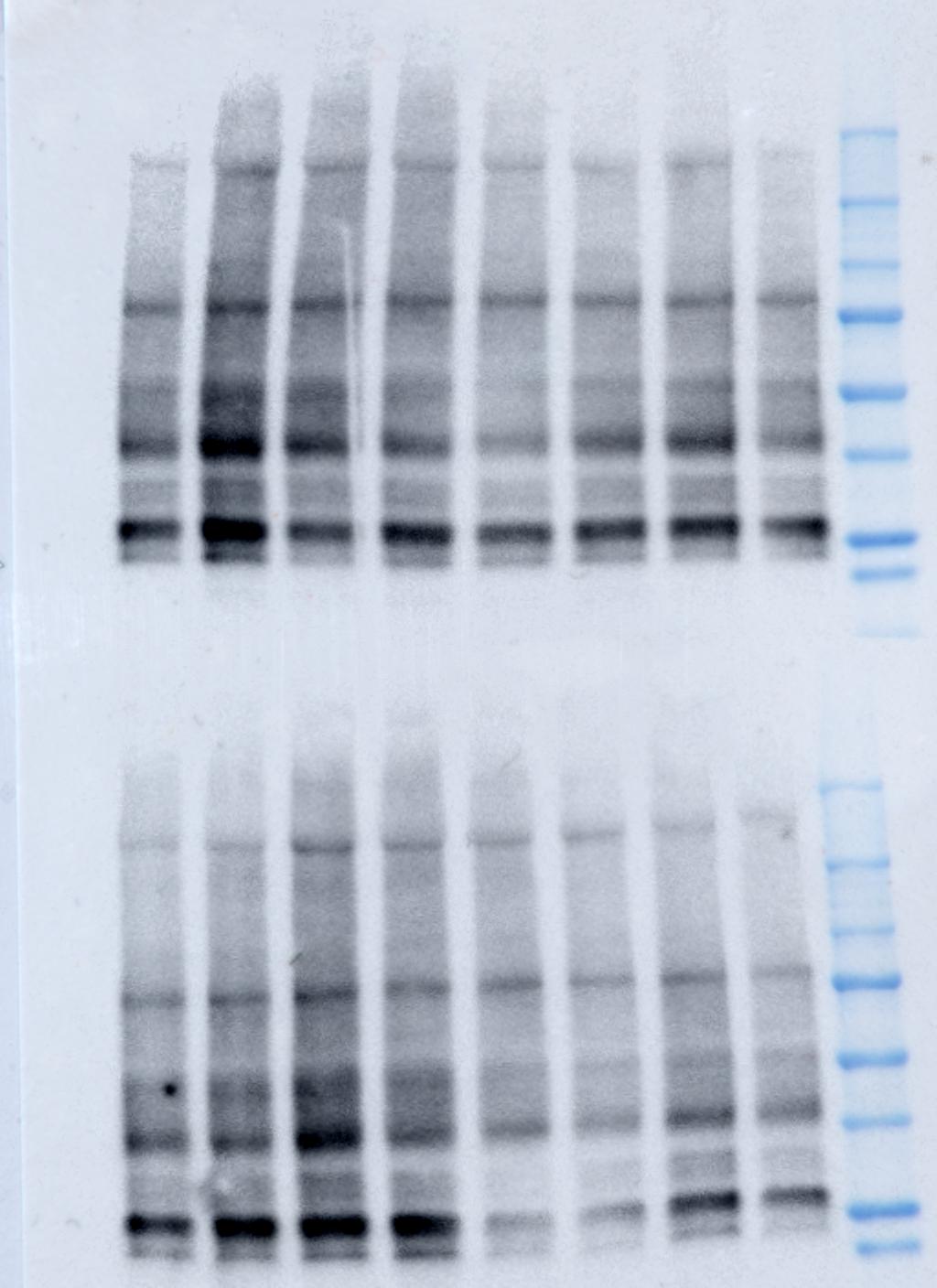

Supplement: Figure 4—source data 1. [file elife-80949-fig4-data1.zip › Figure 4/CamKII/p-CAMK2 5S 2018.07.24_11.43.41_Ch+Marker.jpg]

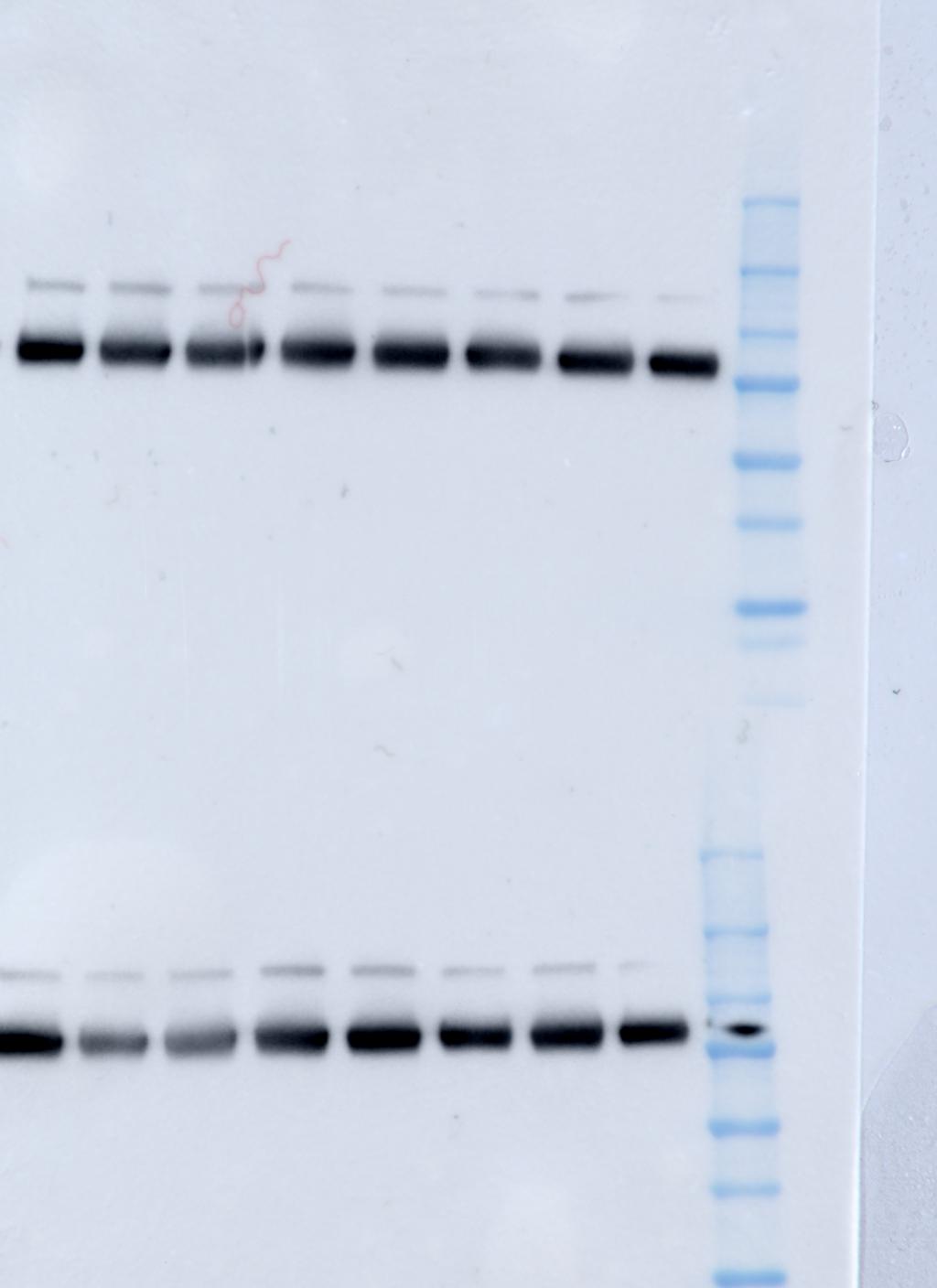

Supplement: Figure 4—source data 1. [file elife-80949-fig4-data1.zip › Figure 4/CamKII/Vinculin for CamKii 2018.07.26_11.15.04_Ch+Marker.jpg]

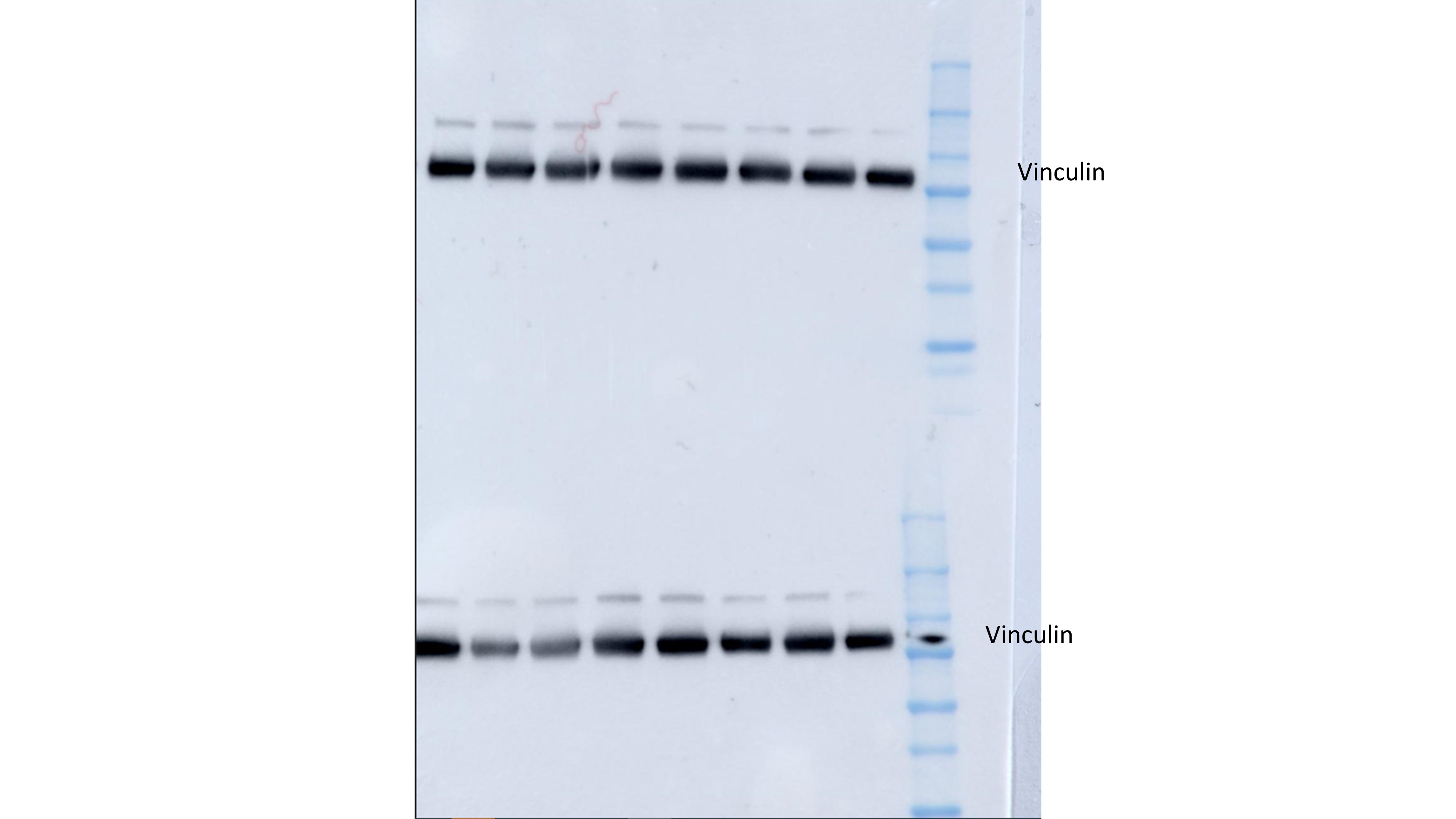

Supplement: Figure 4—source data 1. [file elife-80949-fig4-data1.zip › Figure 4/Figure 4 Uncropped WB Images/CamKII Vinklulin.JPG]

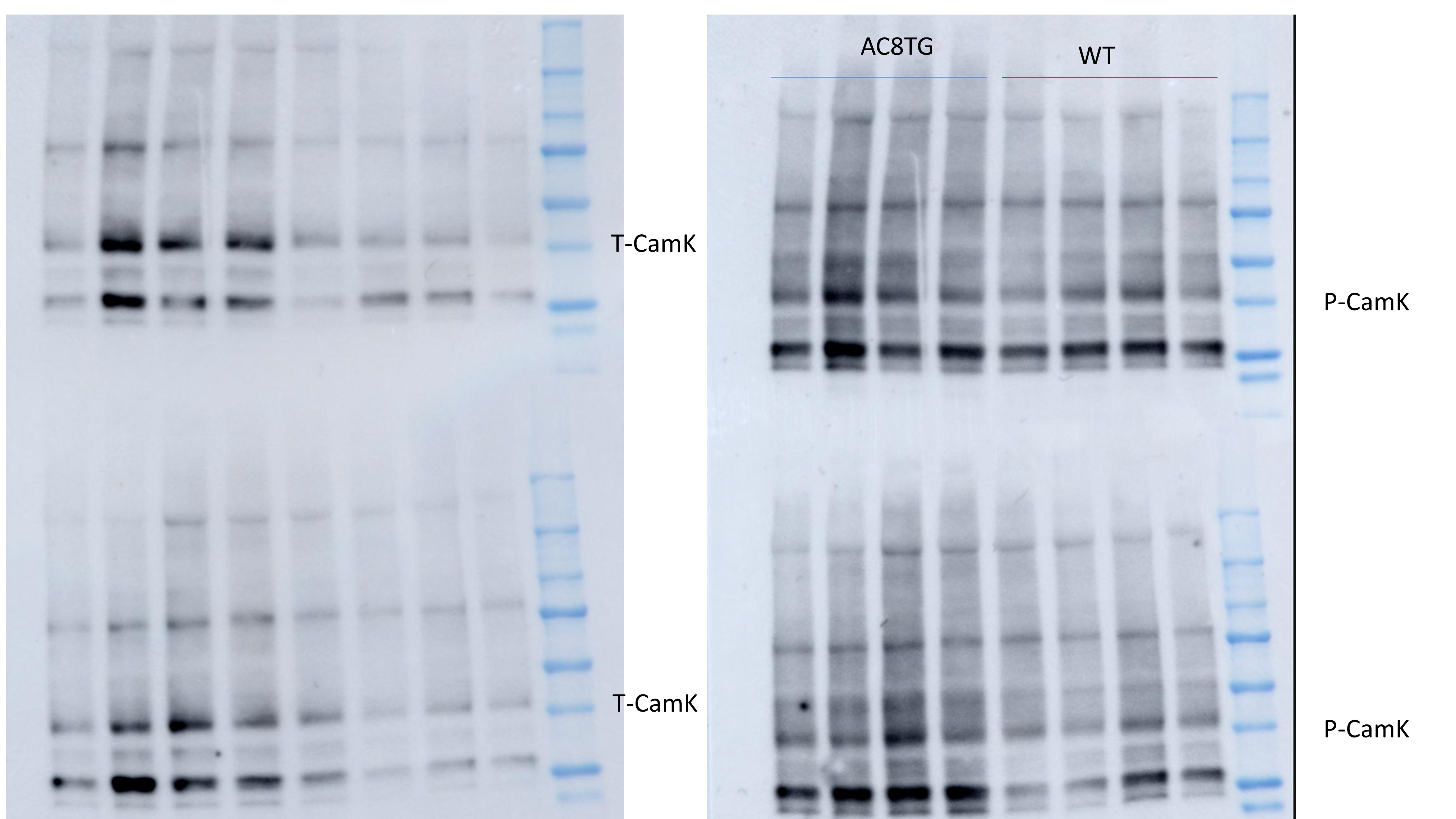

Supplement: Figure 4—source data 1. [file elife-80949-fig4-data1.zip › Figure 4/Figure 4 Uncropped WB Images/CamkII.JPG]

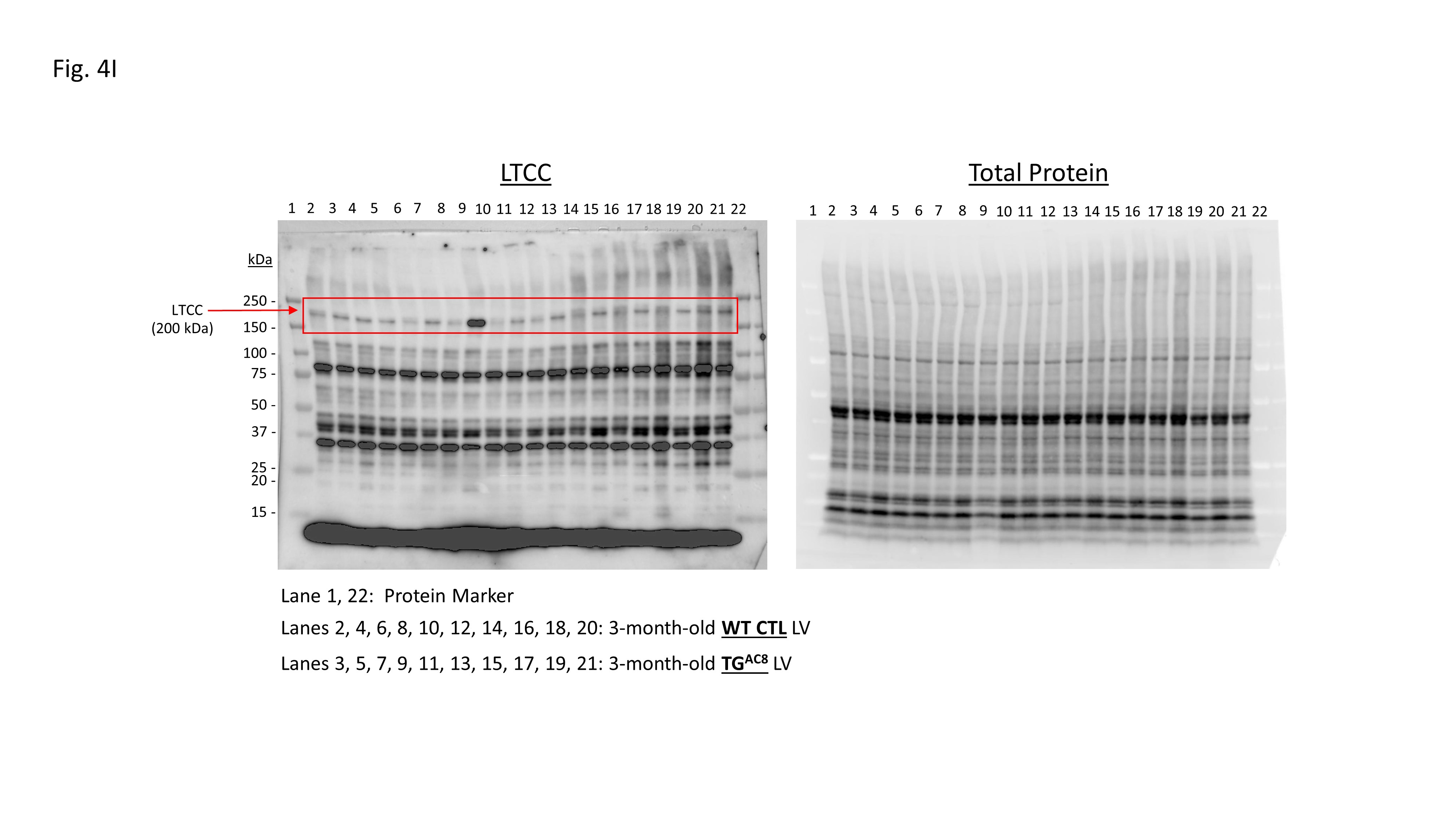

Supplement: Figure 4—source data 1. [file elife-80949-fig4-data1.zip › Figure 4/Figure 4 Uncropped WB Images/Slide10.JPG]

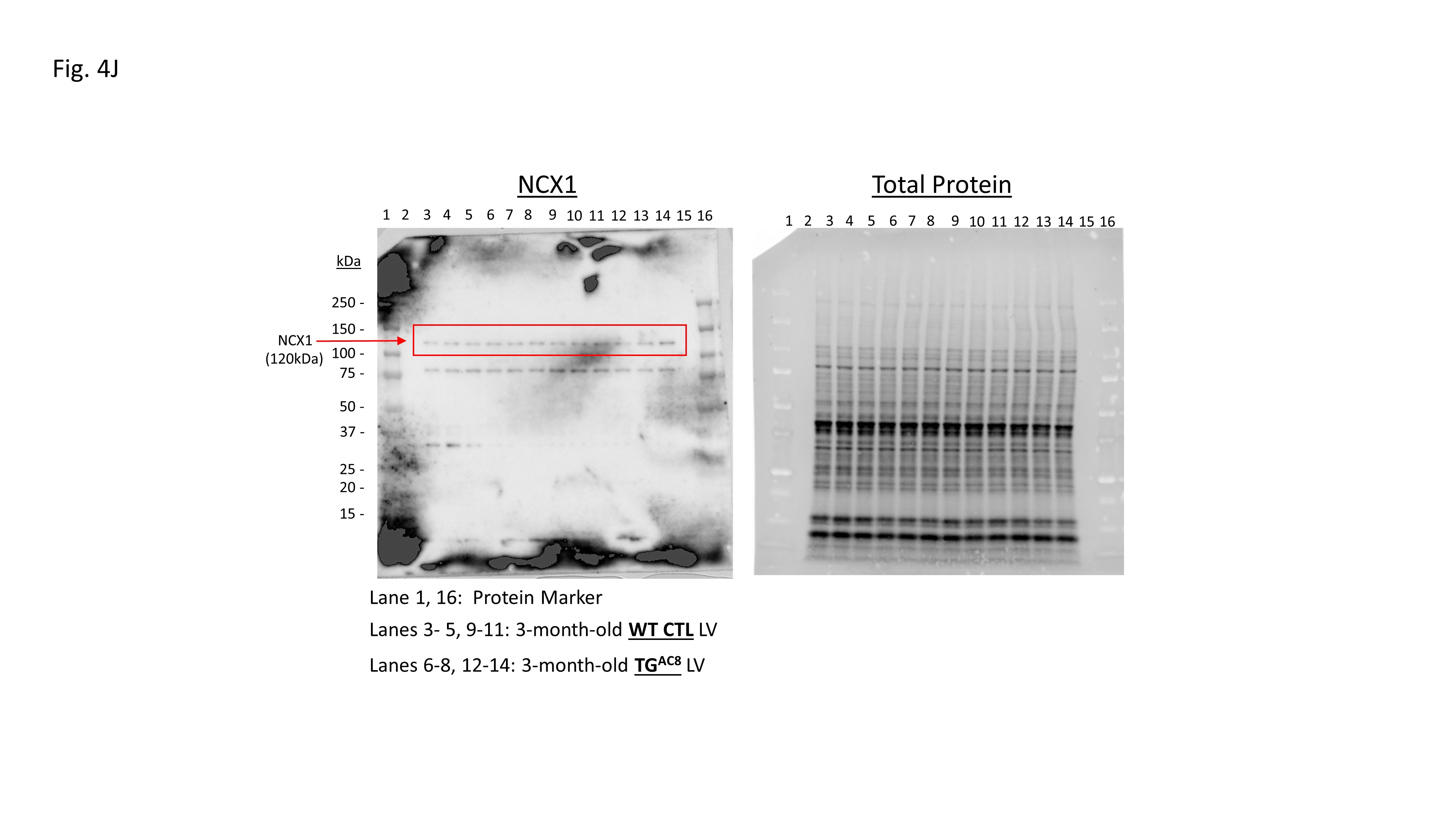

Supplement: Figure 4—source data 1. [file elife-80949-fig4-data1.zip › Figure 4/Figure 4 Uncropped WB Images/Slide11.JPG]

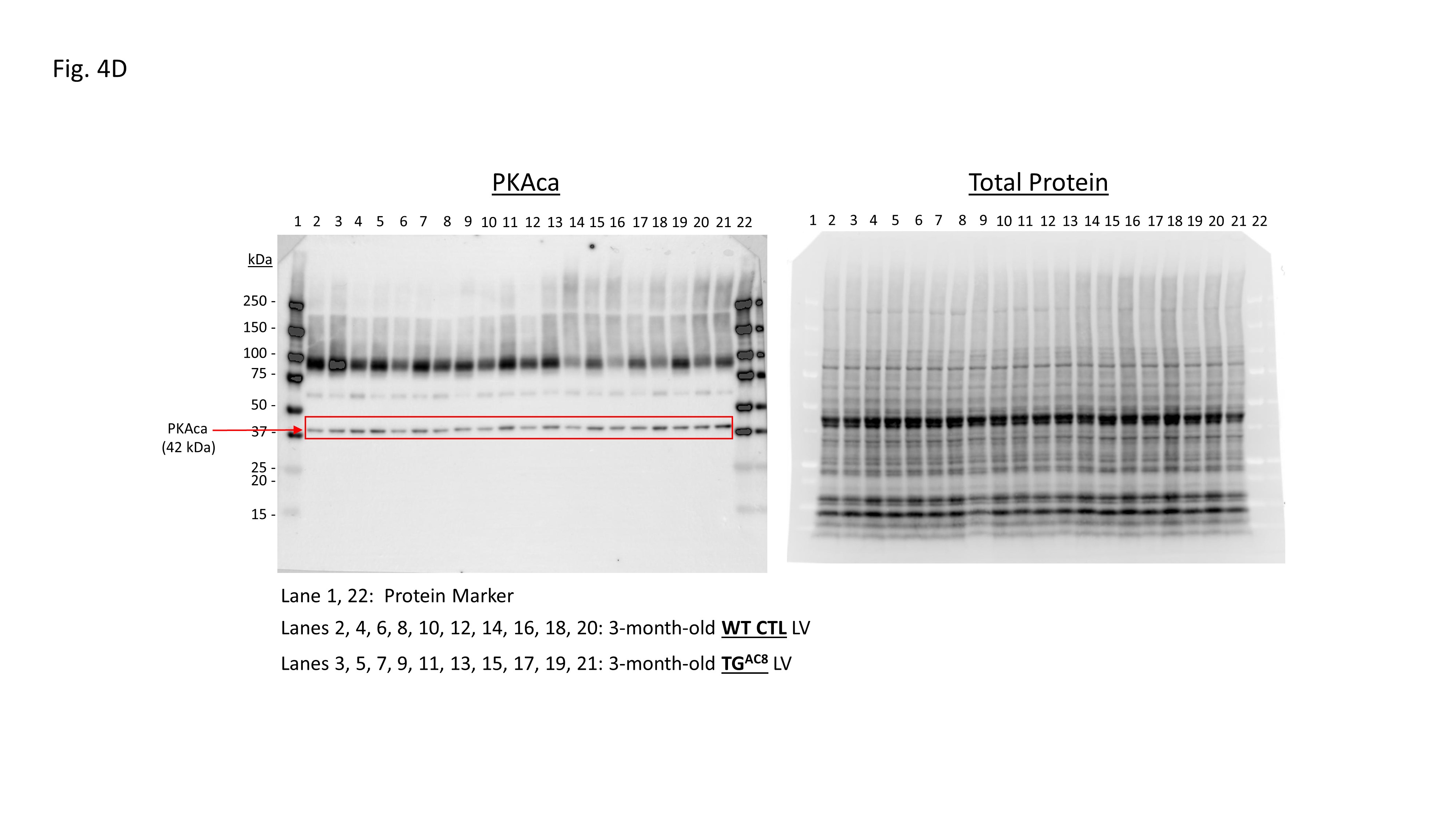

Supplement: Figure 4—source data 1. [file elife-80949-fig4-data1.zip › Figure 4/Figure 4 Uncropped WB Images/Slide6.JPG]

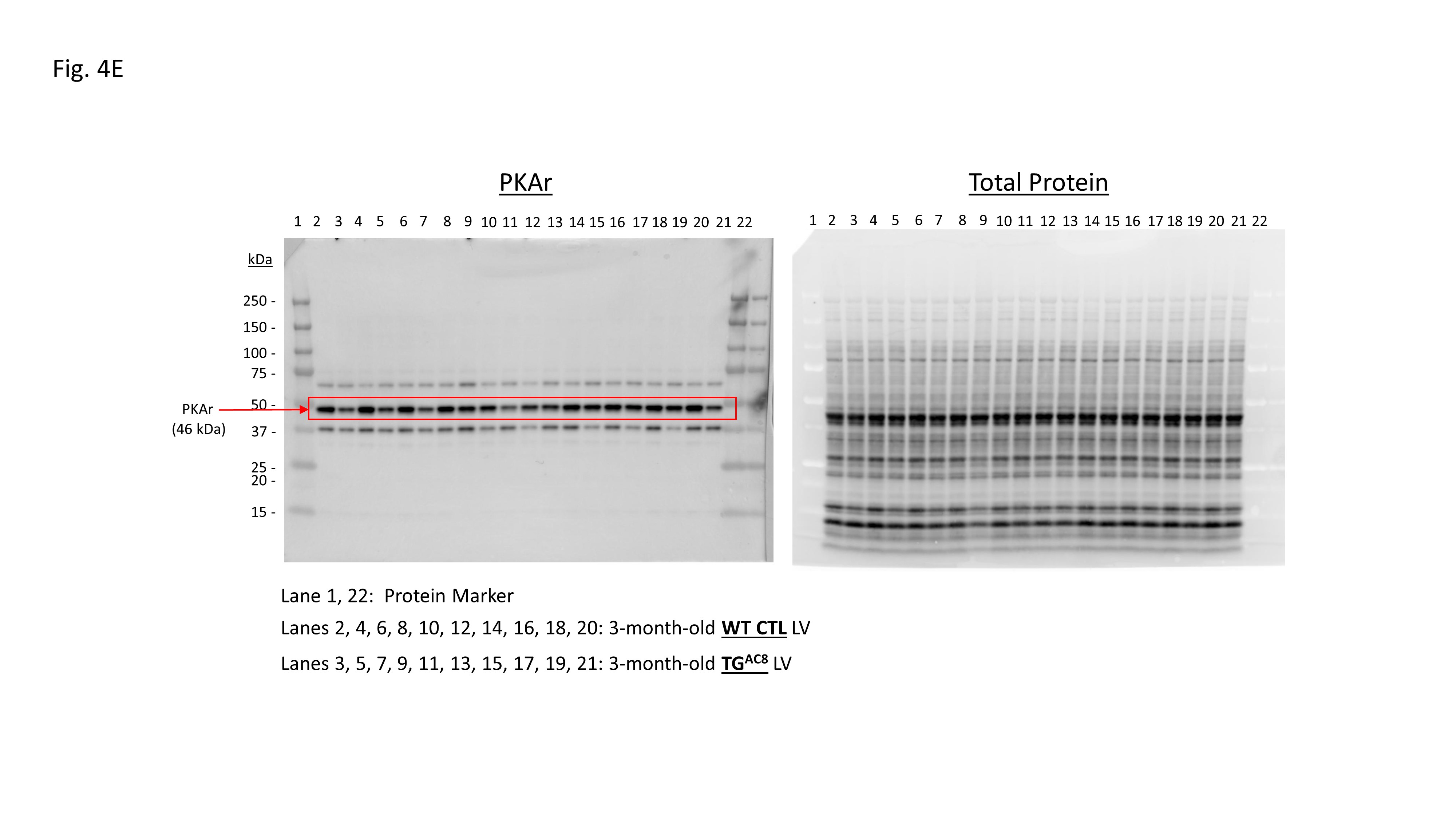

Supplement: Figure 4—source data 1. [file elife-80949-fig4-data1.zip › Figure 4/Figure 4 Uncropped WB Images/Slide7.JPG]

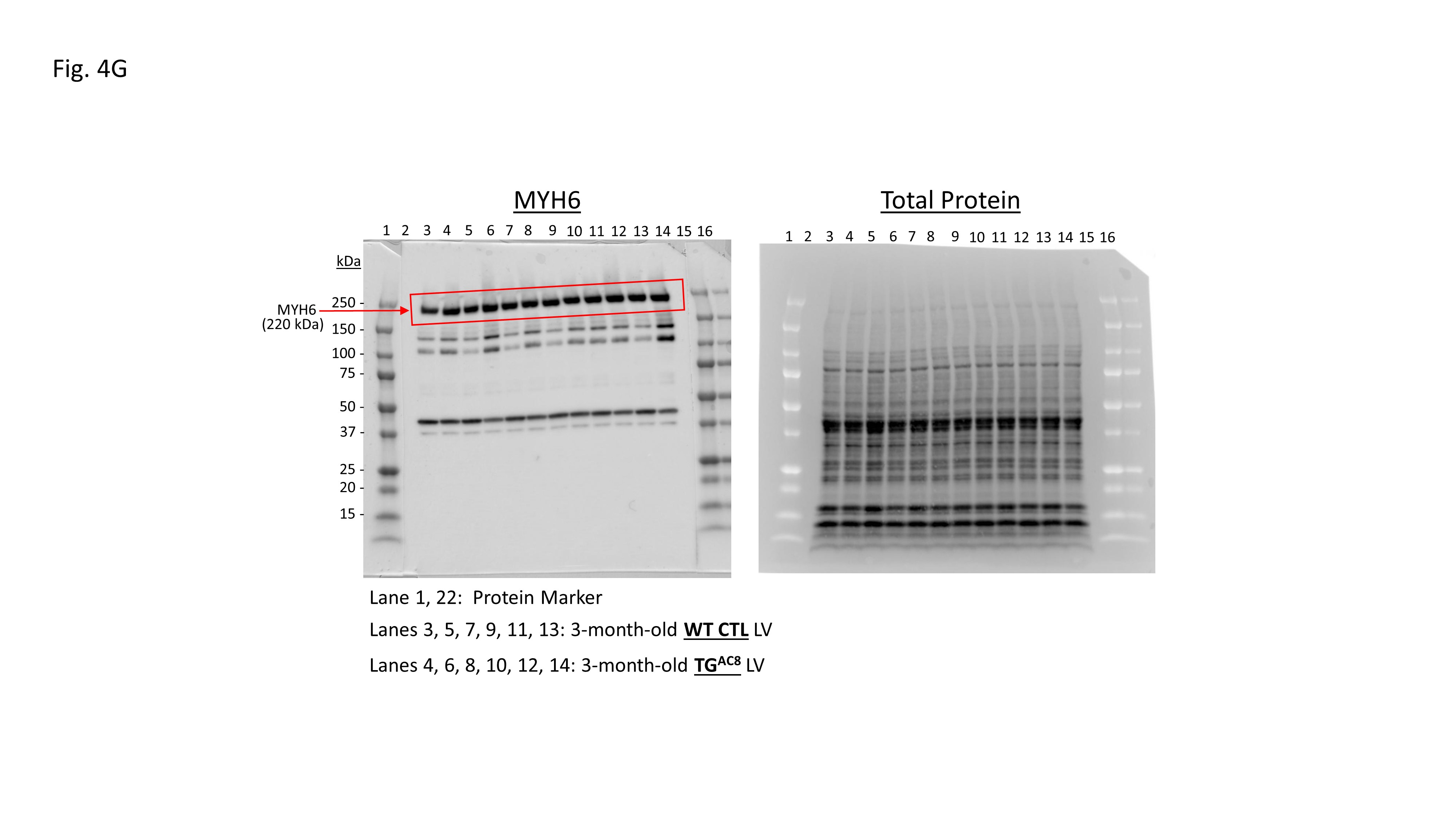

Supplement: Figure 4—source data 1. [file elife-80949-fig4-data1.zip › Figure 4/Figure 4 Uncropped WB Images/Slide8.JPG]

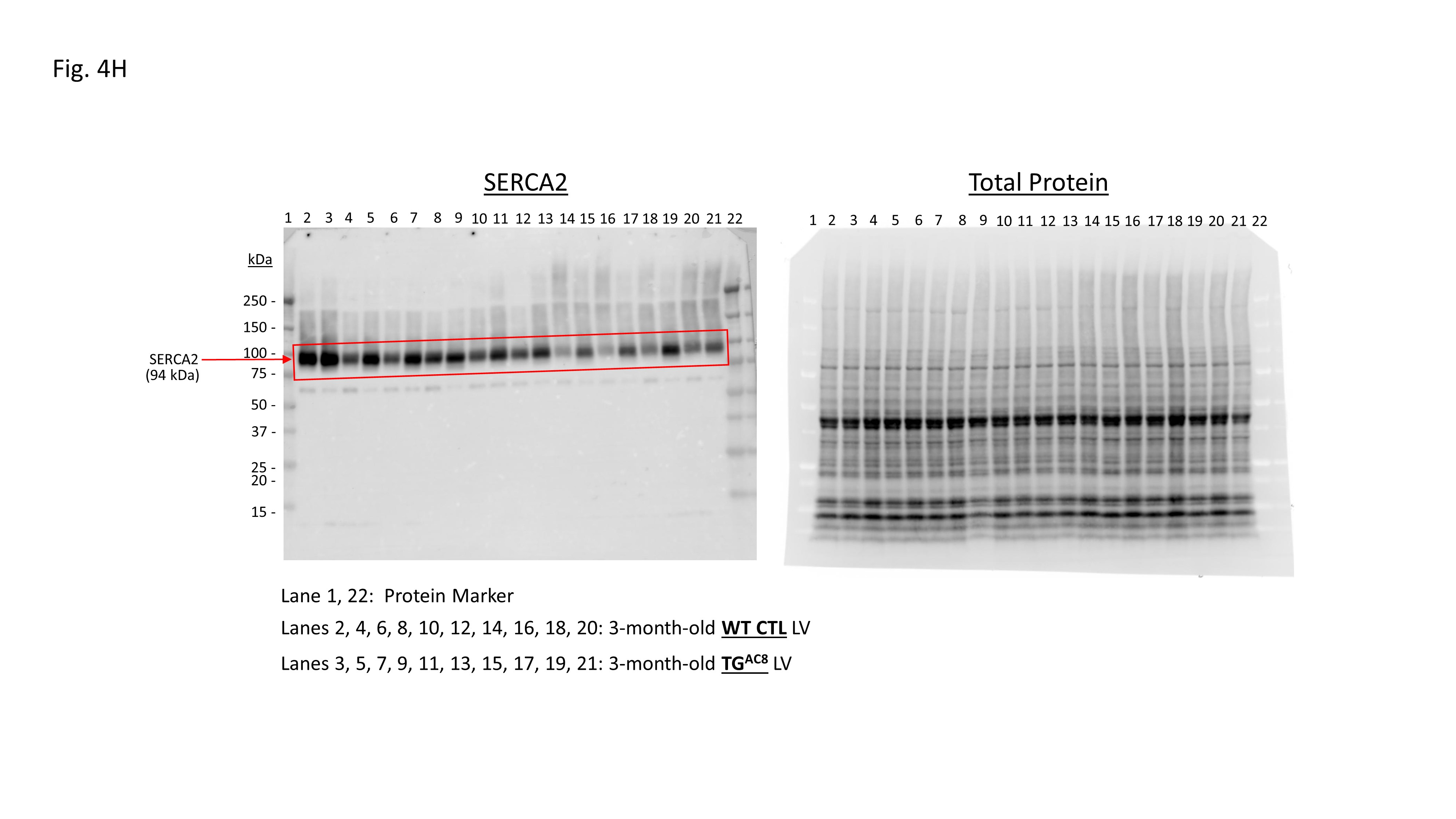

Supplement: Figure 4—source data 1. [file elife-80949-fig4-data1.zip › Figure 4/Figure 4 Uncropped WB Images/Slide9.JPG]

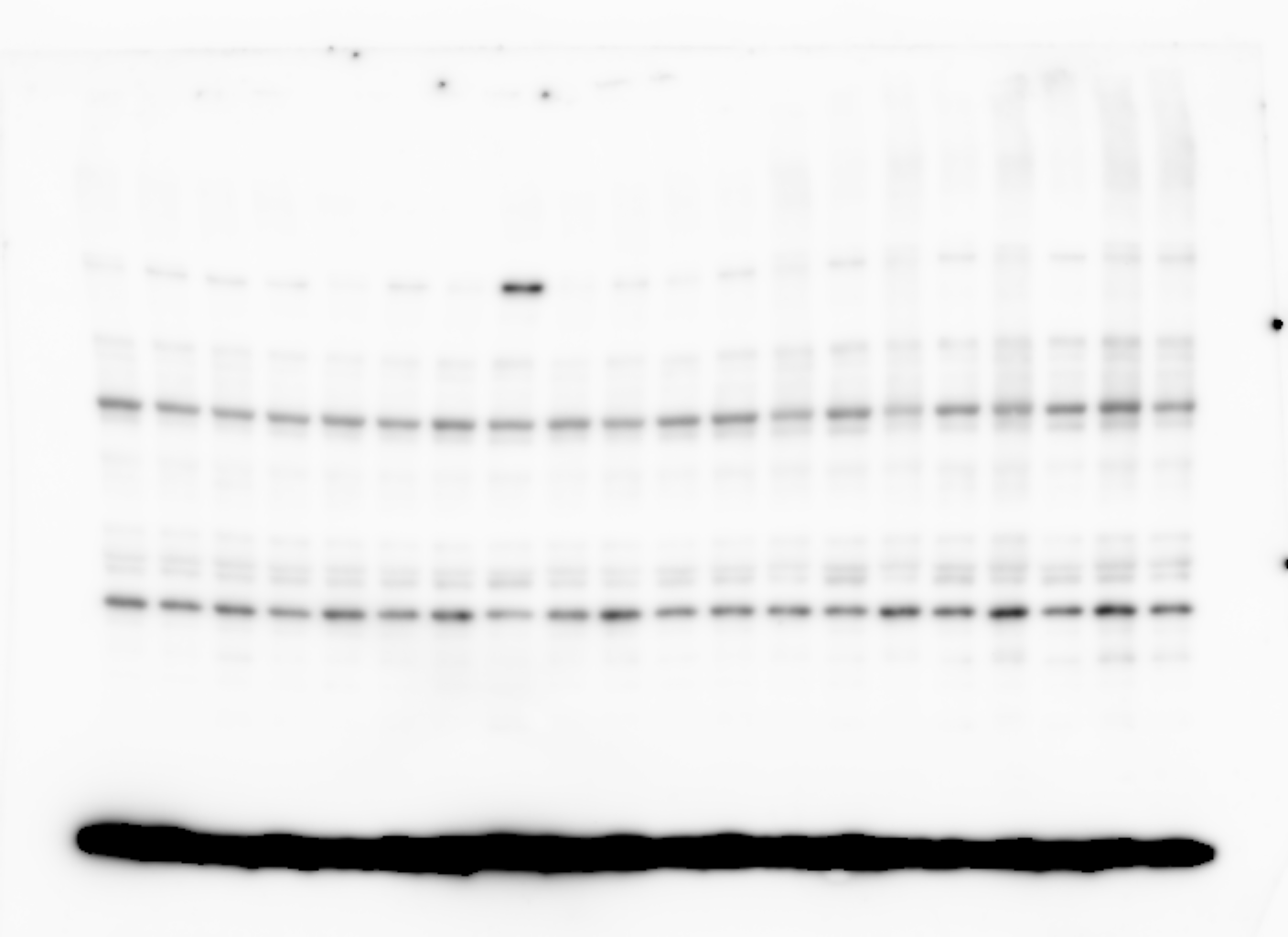

Supplement: Figure 4—source data 1. [file elife-80949-fig4-data1.zip › Figure 4/LTCC/LTCC/DR LTCC LV Blot35 2018.09.12_11.52.24_Ch.tif]

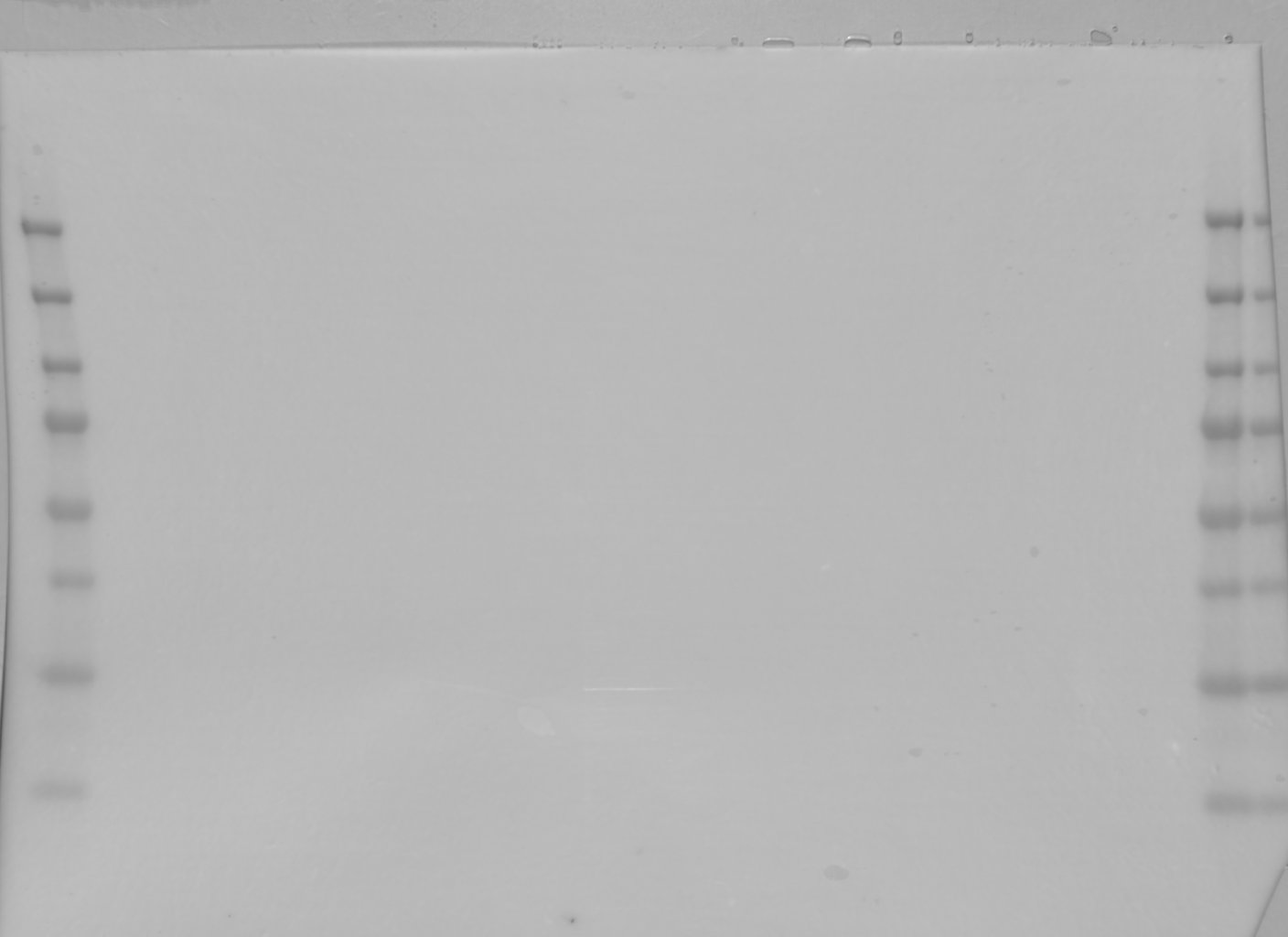

Supplement: Figure 4—source data 1. [file elife-80949-fig4-data1.zip › Figure 4/LTCC/LTCC/DR LTCC LV Blot35 2018.09.12_11.52.24_Ch-Marker.tif]

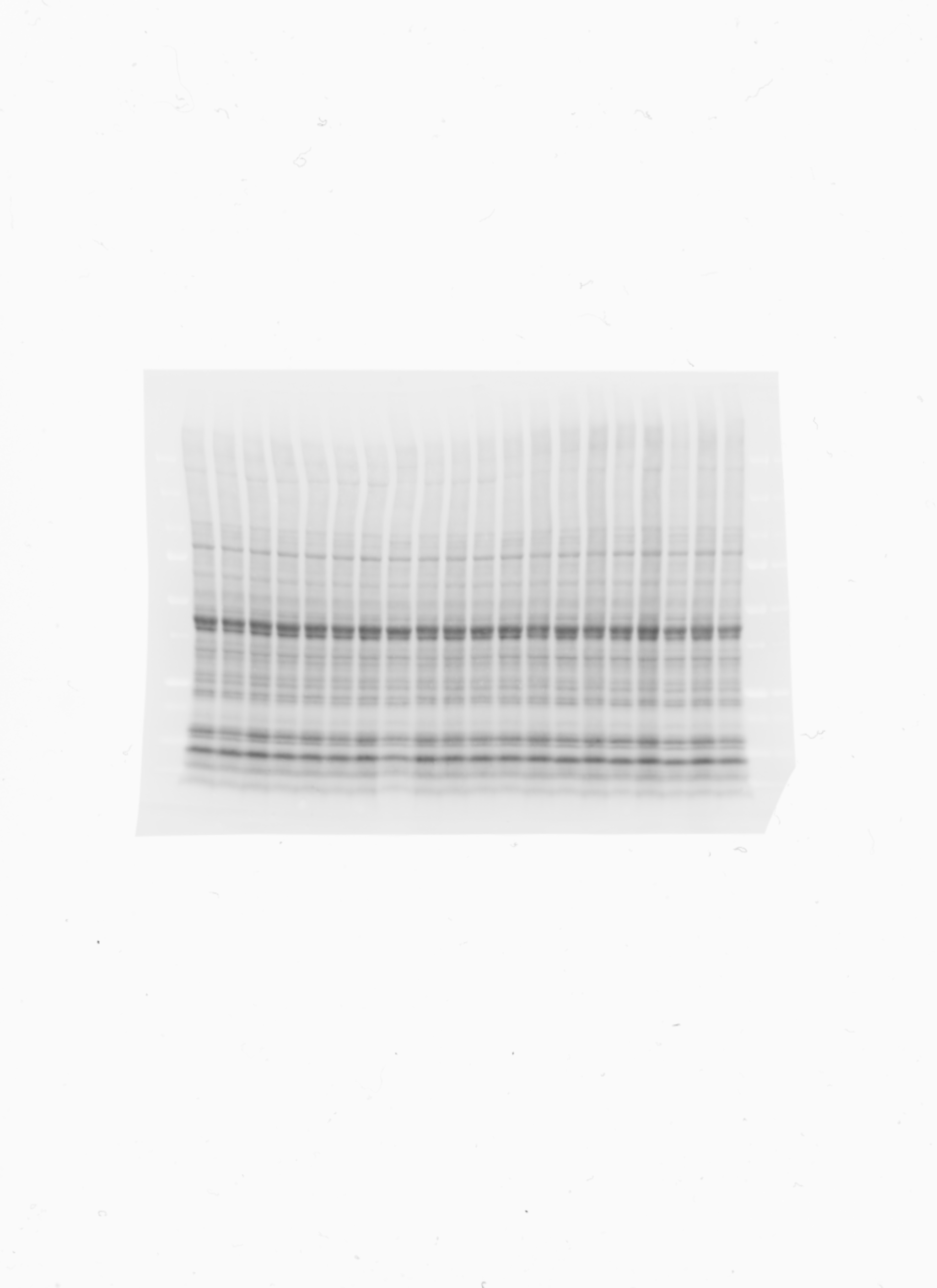

Supplement: Figure 4—source data 1. [file elife-80949-fig4-data1.zip › Figure 4/LTCC/Total Protein/DR T.Prot LV Blot35 2018.09.06_13.42.58_Fl-UV.tif]

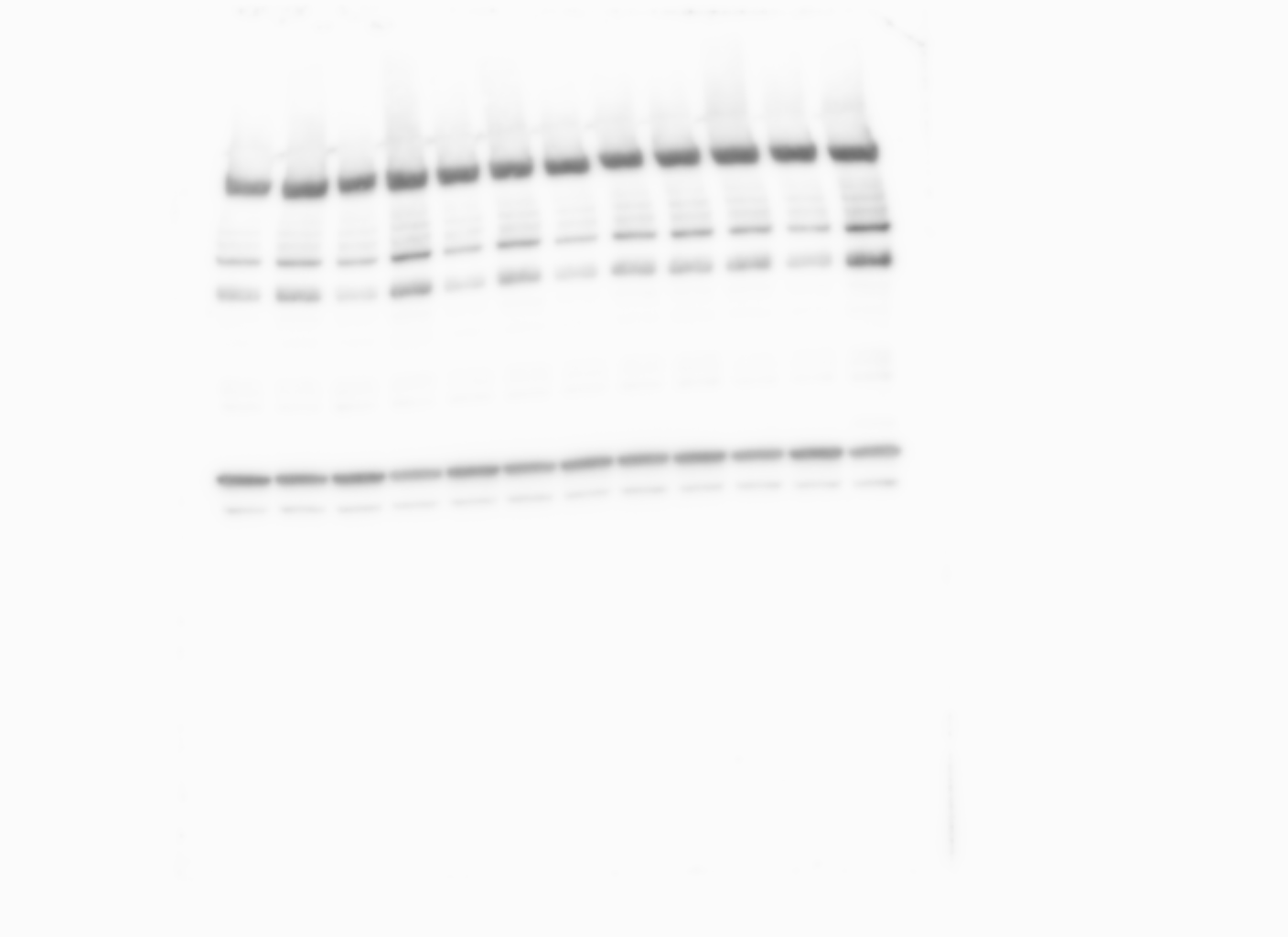

Supplement: Figure 4—source data 1. [file elife-80949-fig4-data1.zip › Figure 4/MYH6/MYH6/DR MYH6 LV blt49 2019.06.20_10.49.48_Ch.tif]

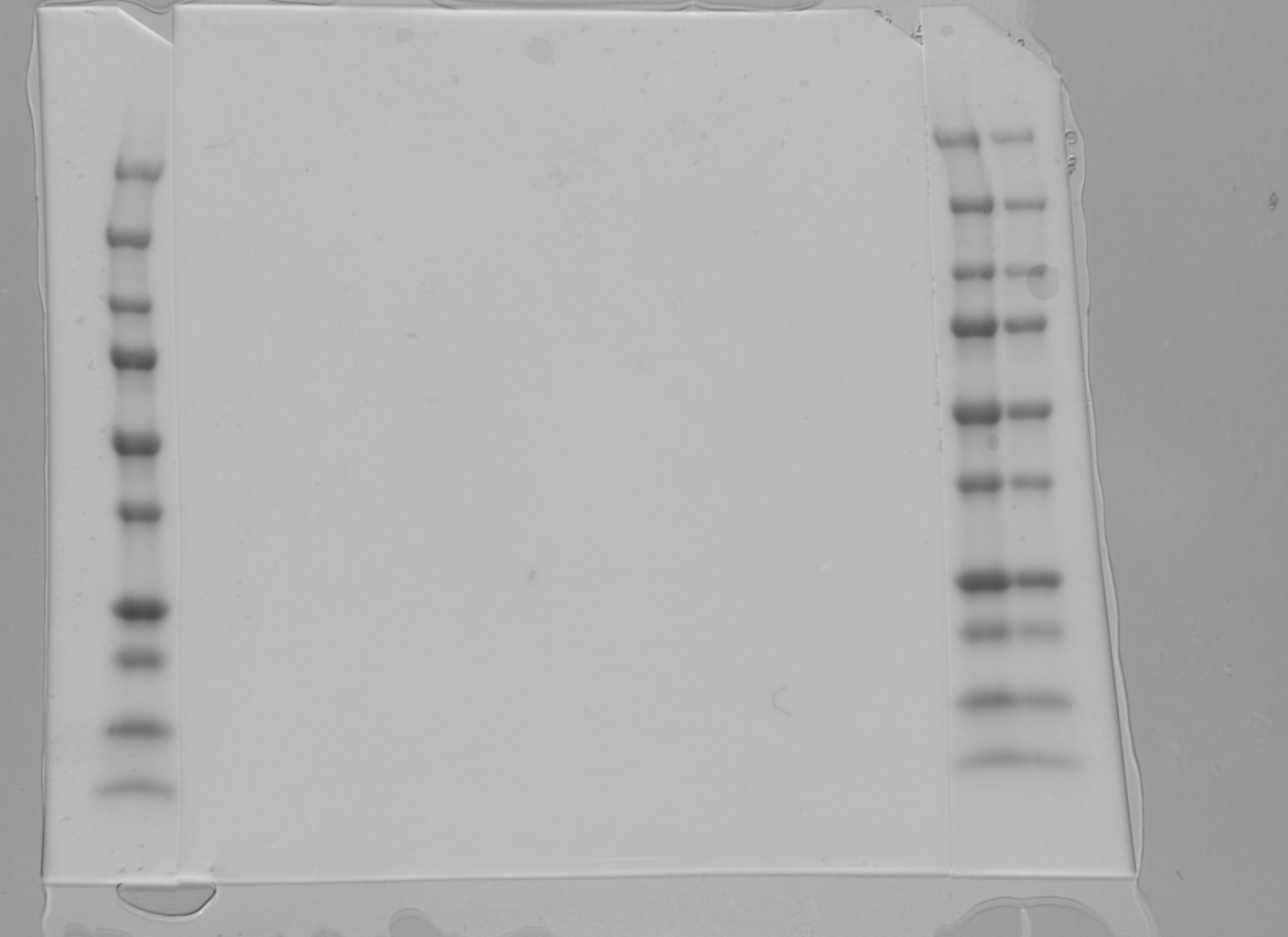

Supplement: Figure 4—source data 1. [file elife-80949-fig4-data1.zip › Figure 4/MYH6/MYH6/DR MYH6 LV blt49 2019.06.20_10.49.48_Ch-Marker.tif]

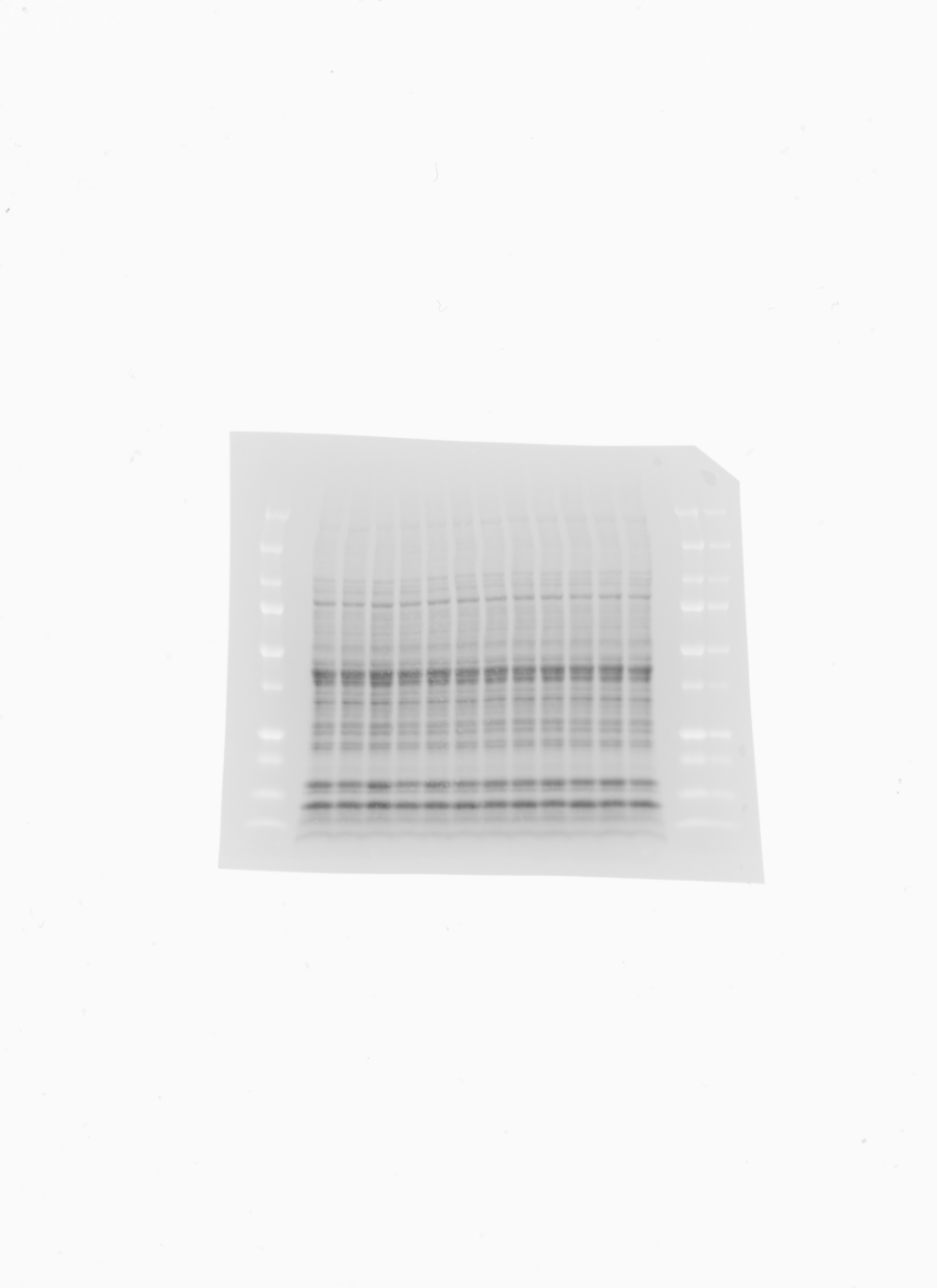

Supplement: Figure 4—source data 1. [file elife-80949-fig4-data1.zip › Figure 4/MYH6/Total Protein/DR TProt. Blt49 2019.06.18_12.18.39_Fl-UV.tif]

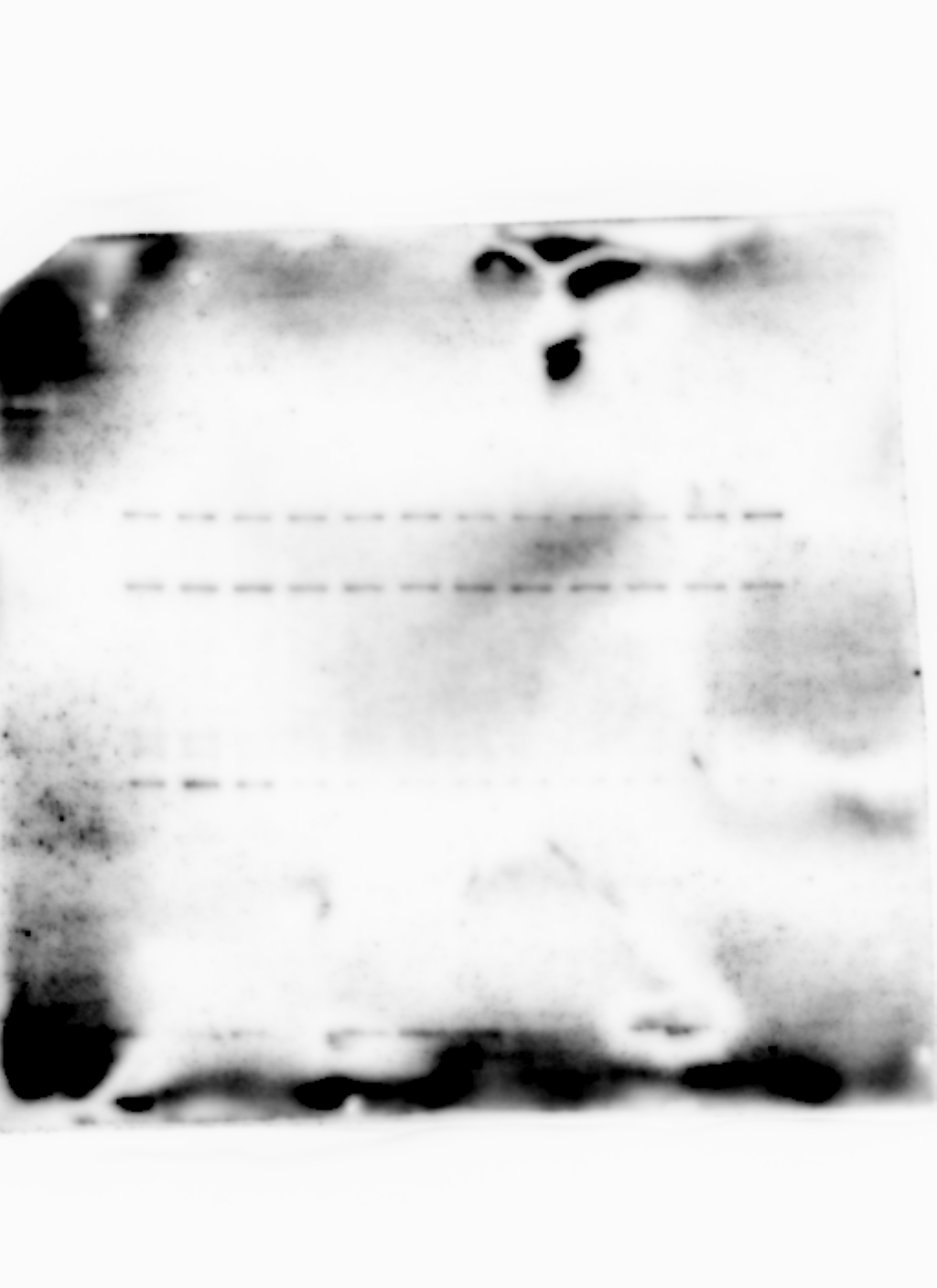

Supplement: Figure 4—source data 1. [file elife-80949-fig4-data1.zip › Figure 4/NCX1/NCX1/DR NCX1 LV Blt78 2020.11.24_12.12.01_Ch.tif]

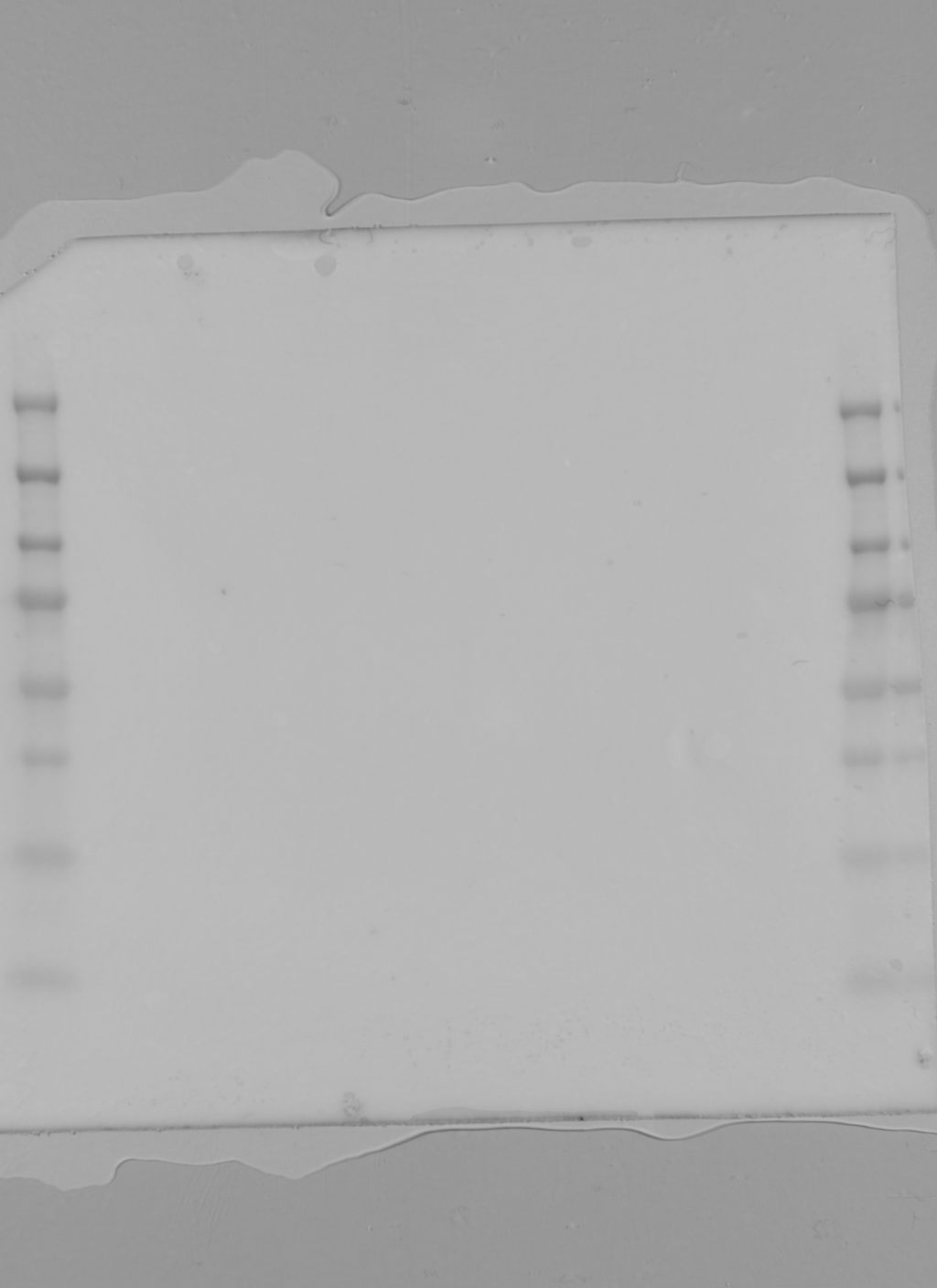

Supplement: Figure 4—source data 1. [file elife-80949-fig4-data1.zip › Figure 4/NCX1/NCX1/DR NCX1 LV Blt78 2020.11.24_12.12.01_Ch-Marker.tif]

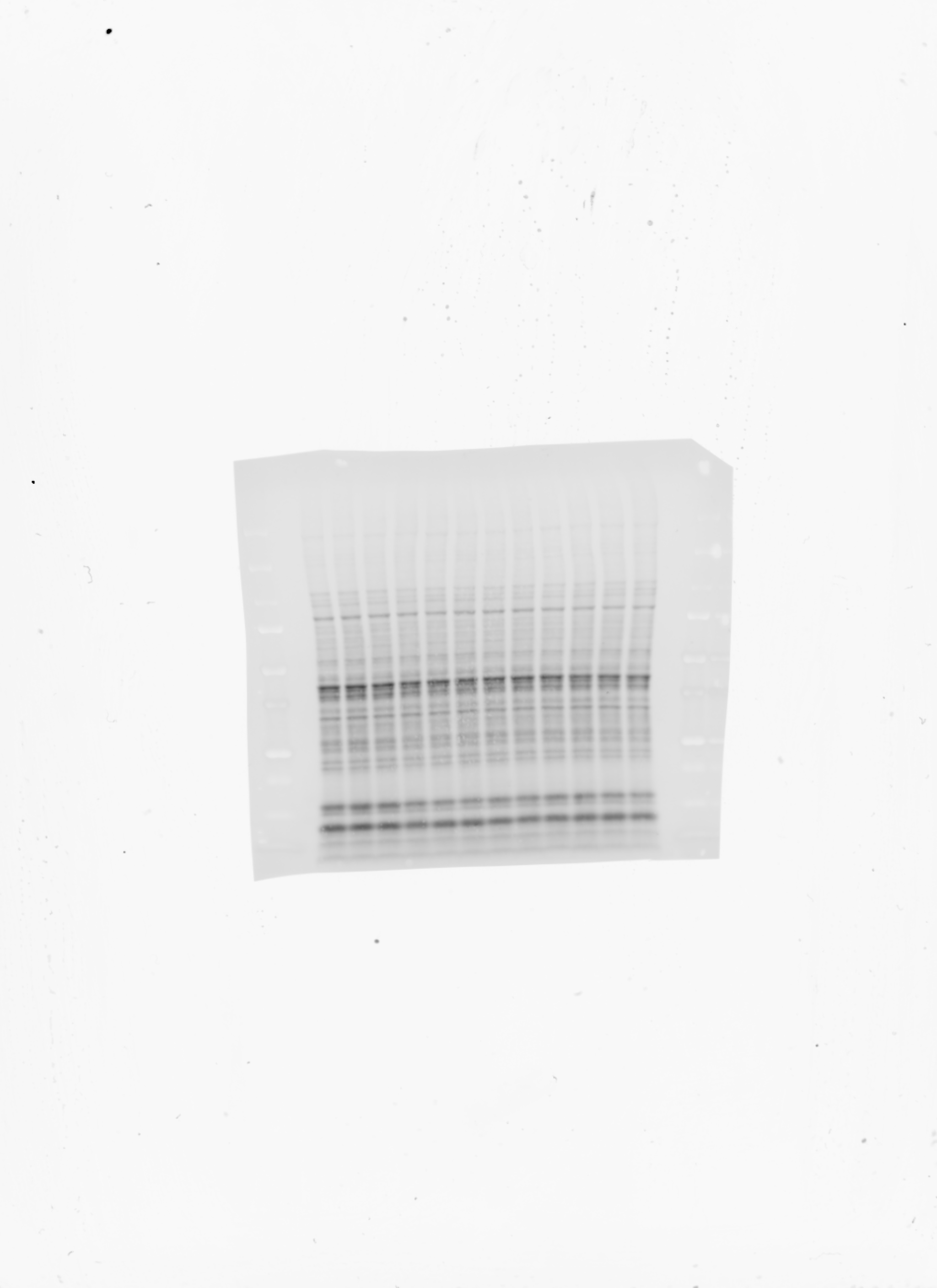

Supplement: Figure 4—source data 1. [file elife-80949-fig4-data1.zip › Figure 4/NCX1/Total Protein/DR T.Prot. LV Blot78 2020.11.03_13.44.28_Fl-UV.tif]

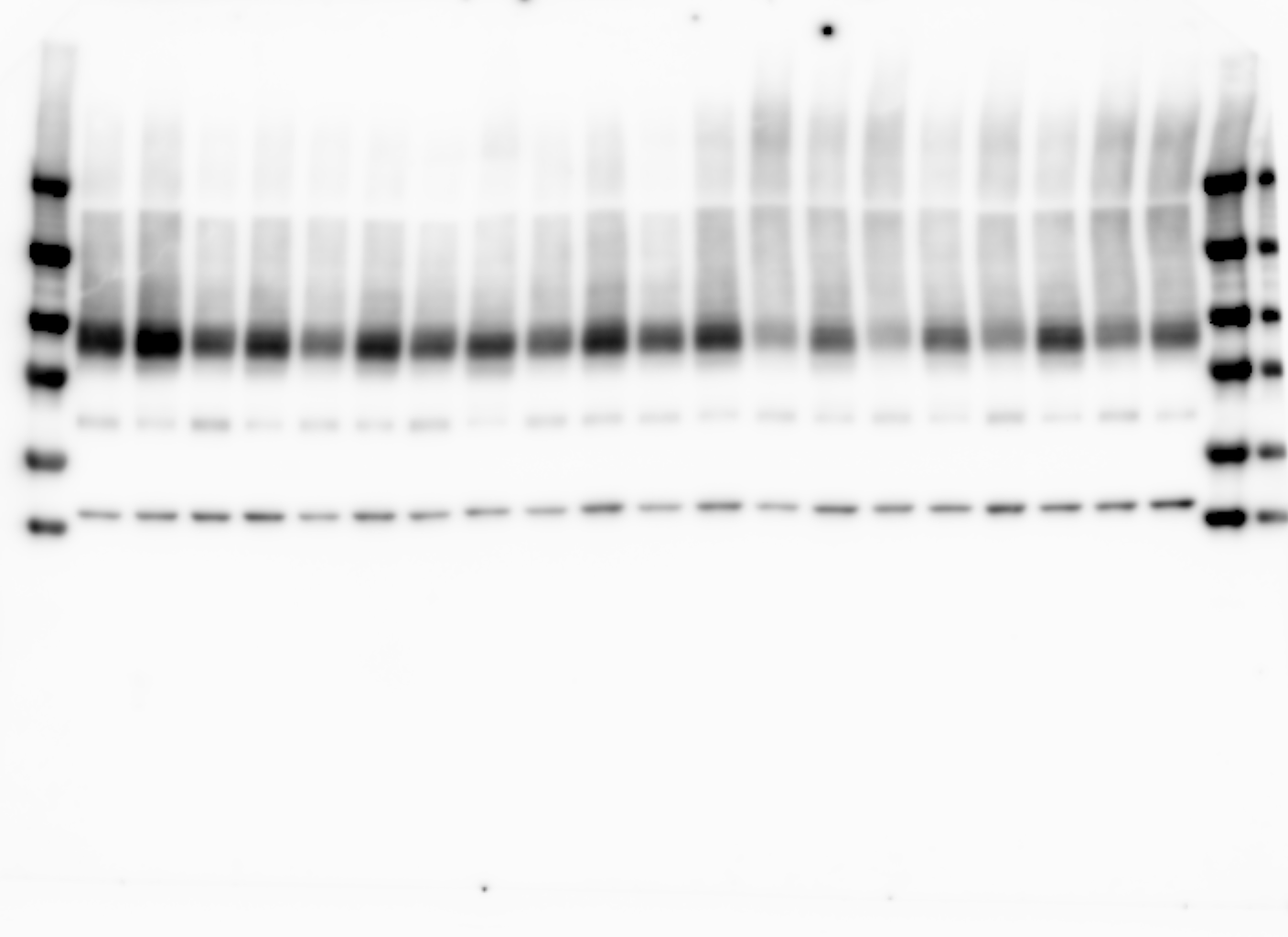

Supplement: Figure 4—source data 1. [file elife-80949-fig4-data1.zip › Figure 4/PKAca/PKAca/DR PKAc LV Blot36 2018.09.18_11.12.58_Ch.tif]

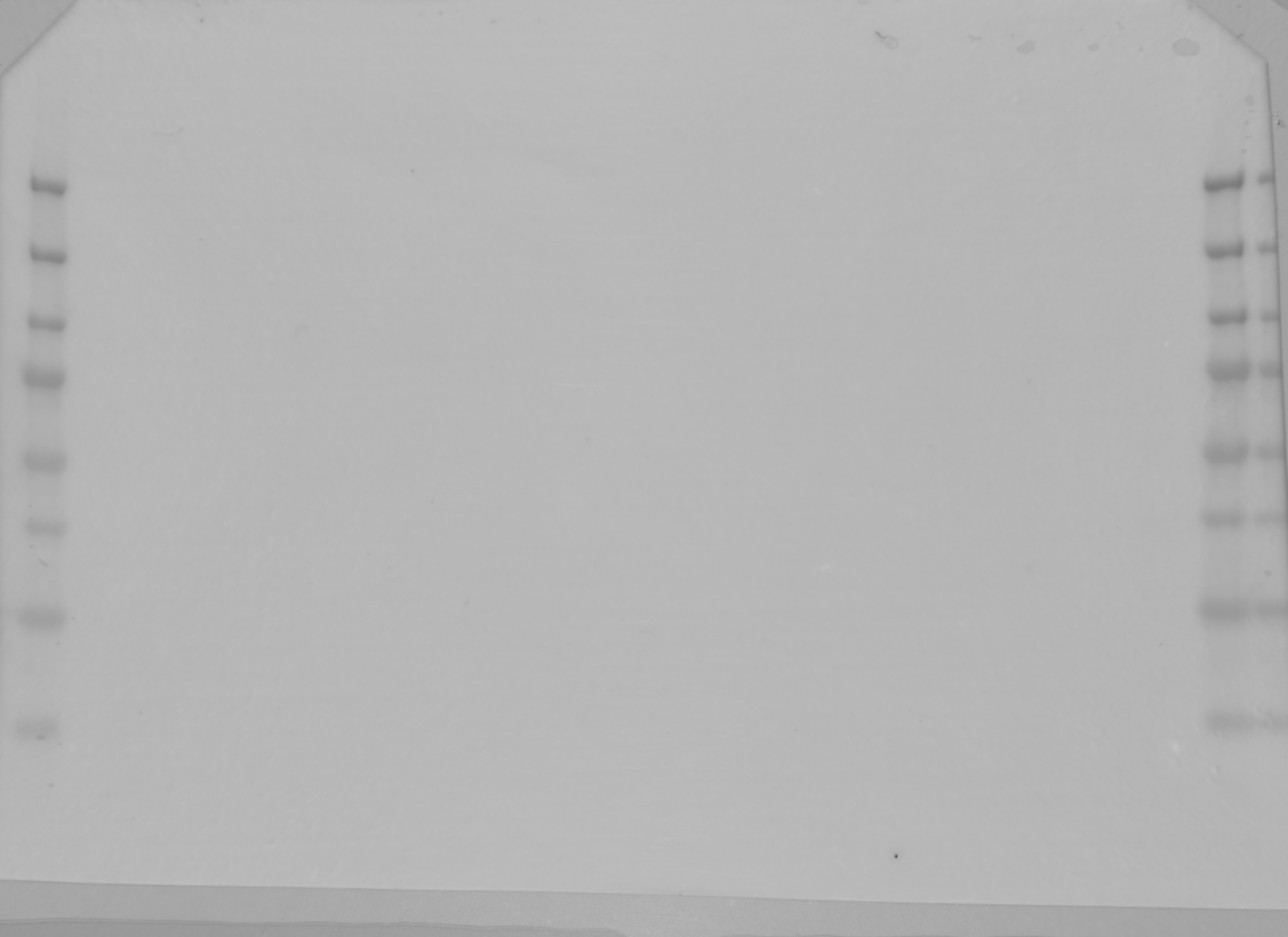

Supplement: Figure 4—source data 1. [file elife-80949-fig4-data1.zip › Figure 4/PKAca/PKAca/DR PKAc LV Blot36 2018.09.18_11.12.58_Ch-Marker.tif]

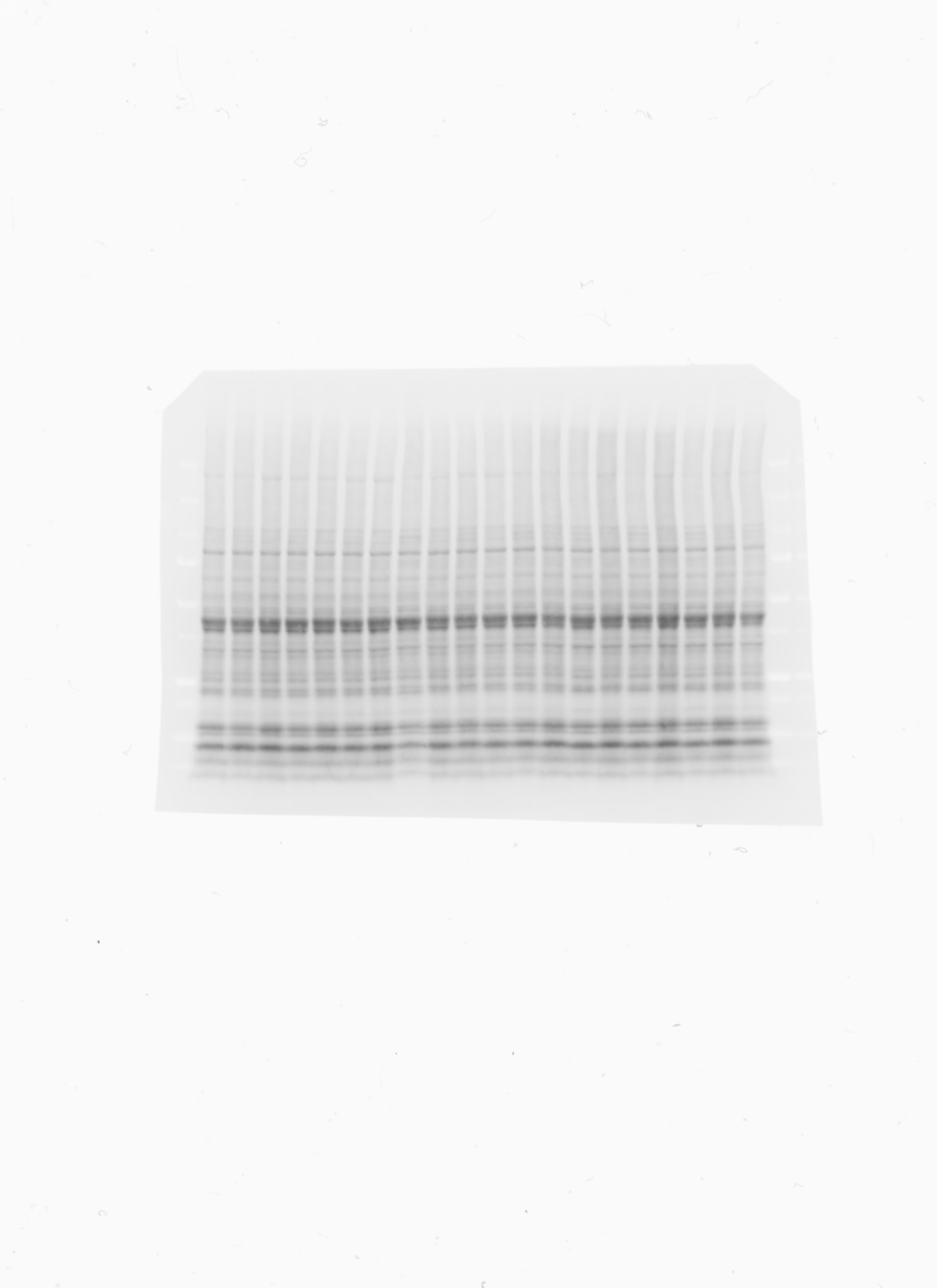

Supplement: Figure 4—source data 1. [file elife-80949-fig4-data1.zip › Figure 4/PKAca/Total Protein/DR T.Prot LV Blot36 2018.09.06_13.44.49_Fl-UV.tif]

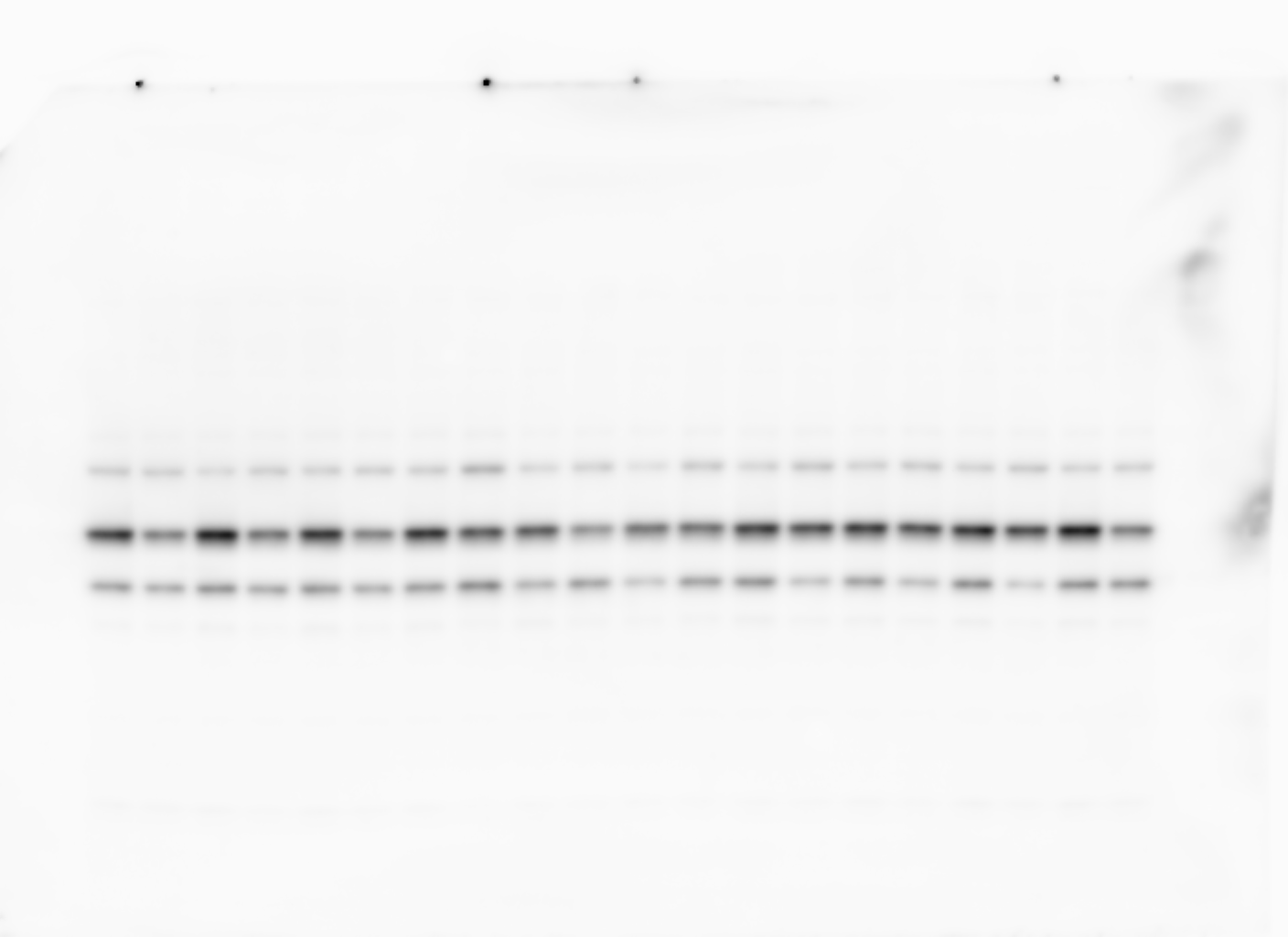

Supplement: Figure 4—source data 1. [file elife-80949-fig4-data1.zip › Figure 4/PKAr/PKAr/DR PKA r1alpha WPP 2018.02.22_11.21.33_Ch.tif]

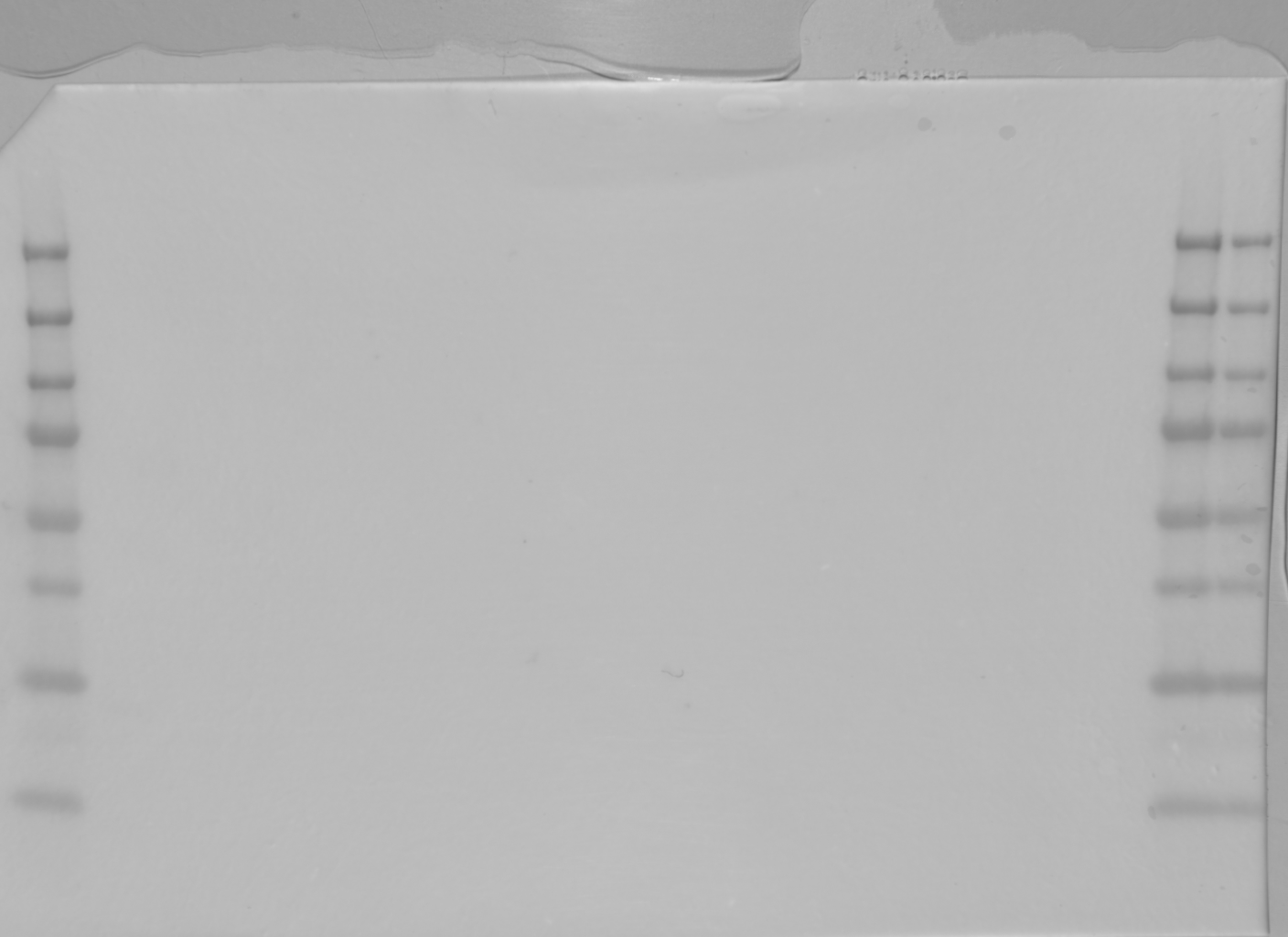

Supplement: Figure 4—source data 1. [file elife-80949-fig4-data1.zip › Figure 4/PKAr/PKAr/DR PKA r1alpha WPP 2018.02.22_11.21.33_Ch-Marker.tif]

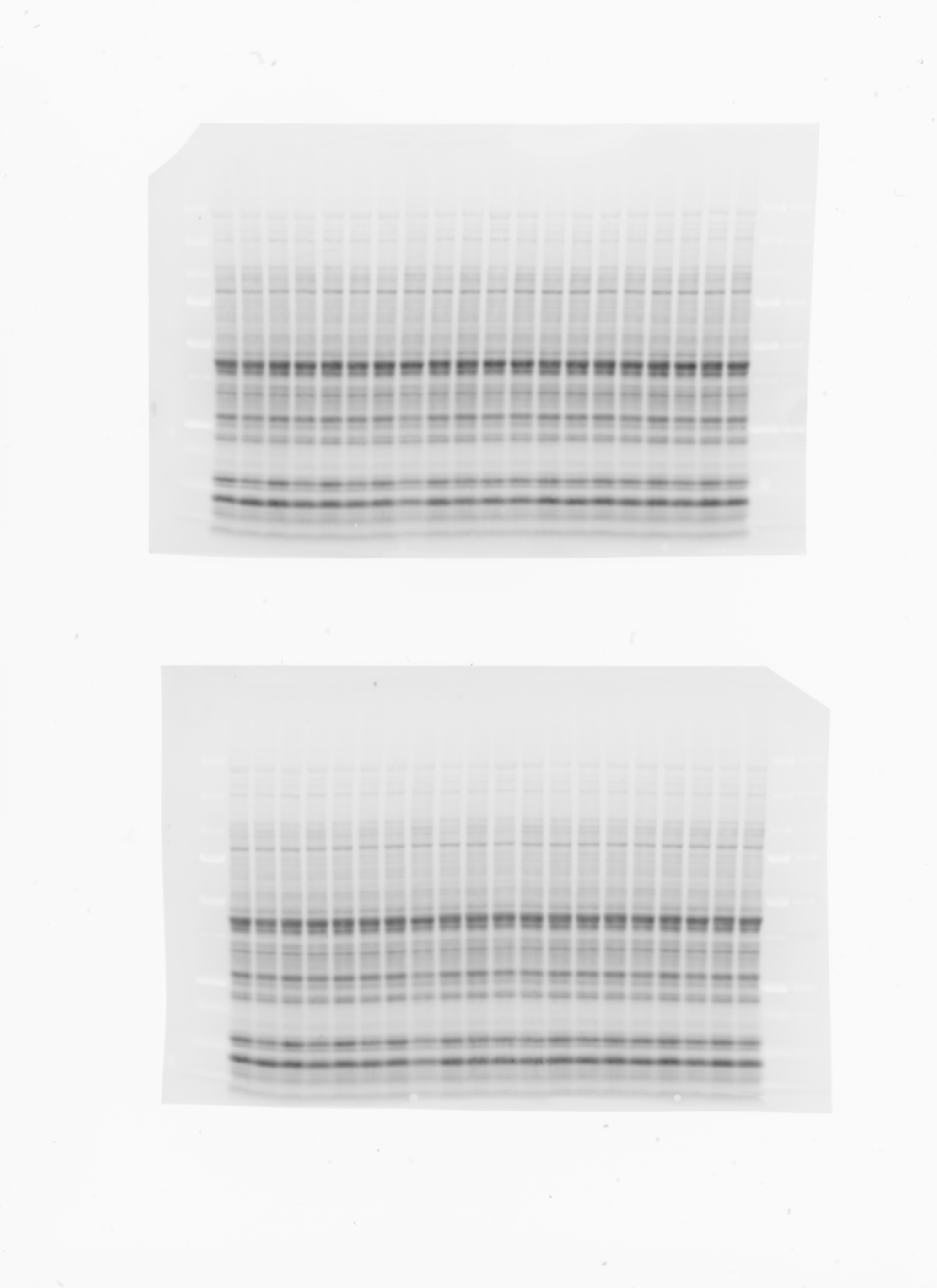

Supplement: Figure 4—source data 1. [file elife-80949-fig4-data1.zip › Figure 4/PKAr/Total Protein/DR Tot.Prot. Blt 5,6 2018.02.14_13.38.46_Fl-UV.tif]

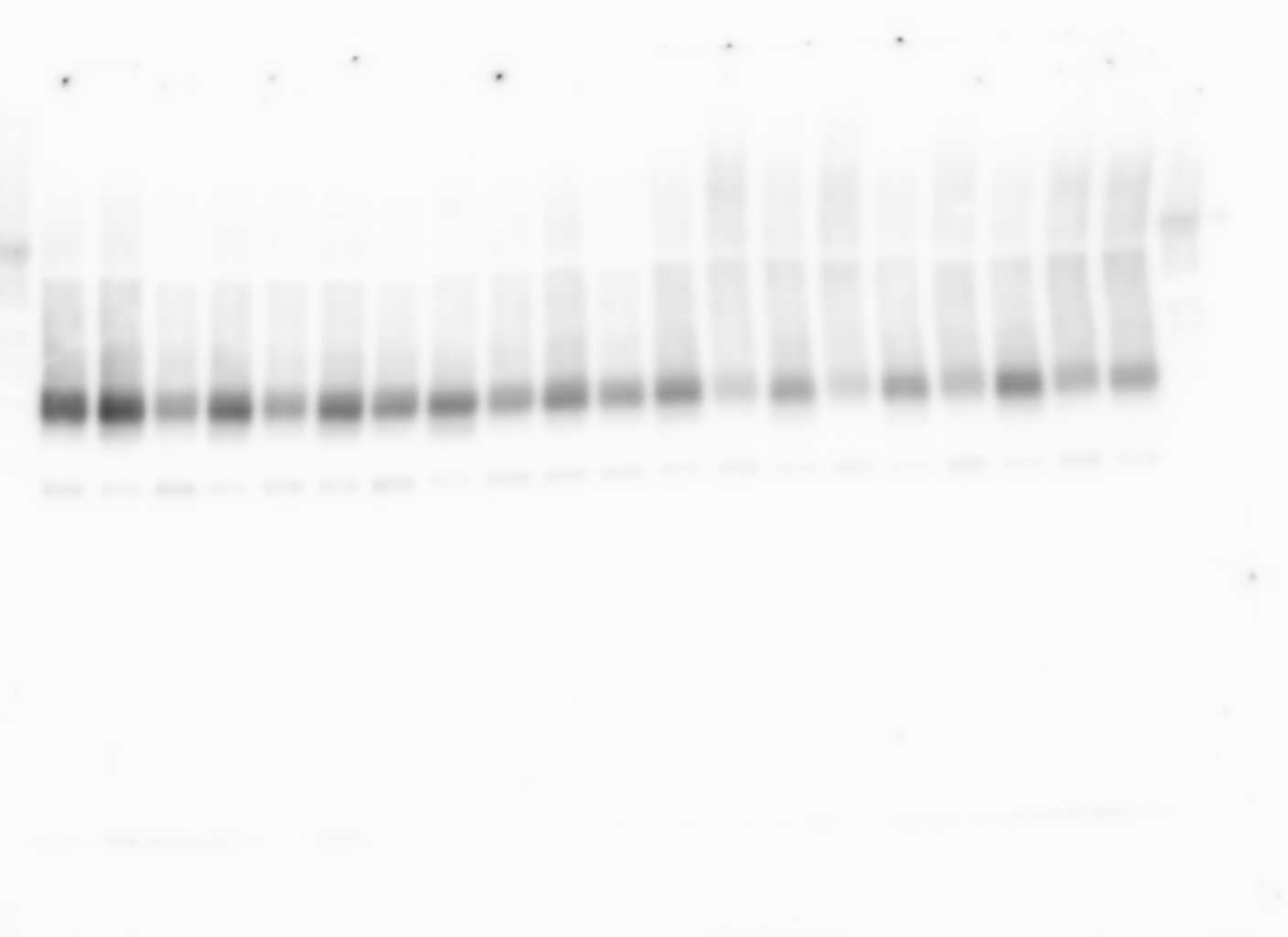

Supplement: Figure 4—source data 1. [file elife-80949-fig4-data1.zip › Figure 4/SERCA2/SERCA2/DR SERCA LV blt36 2018.09.14_12.55.14_Ch.tif]

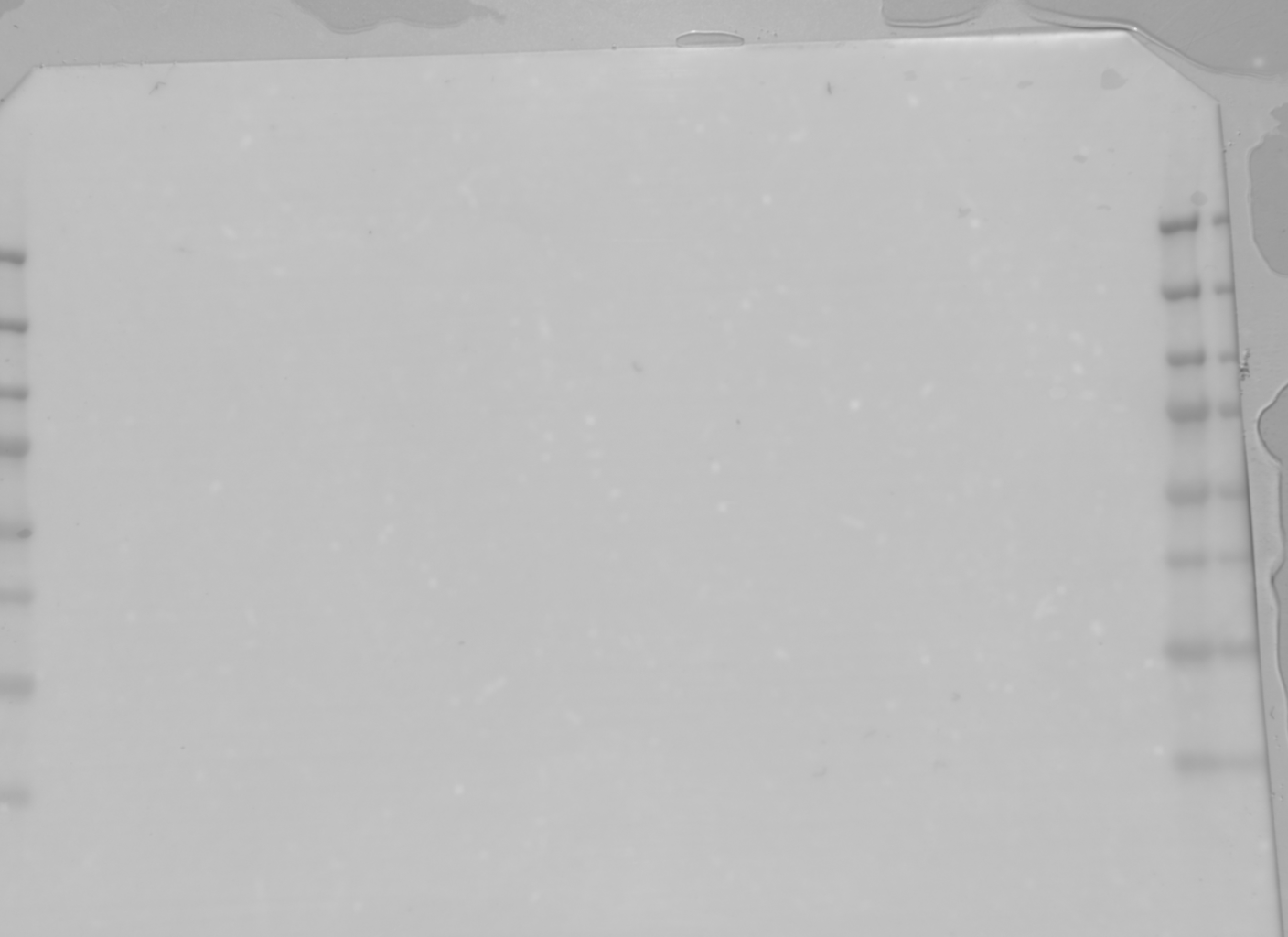

Supplement: Figure 4—source data 1. [file elife-80949-fig4-data1.zip › Figure 4/SERCA2/SERCA2/DR SERCA LV blt36 2018.09.14_12.55.14_Ch-Marker.tif]

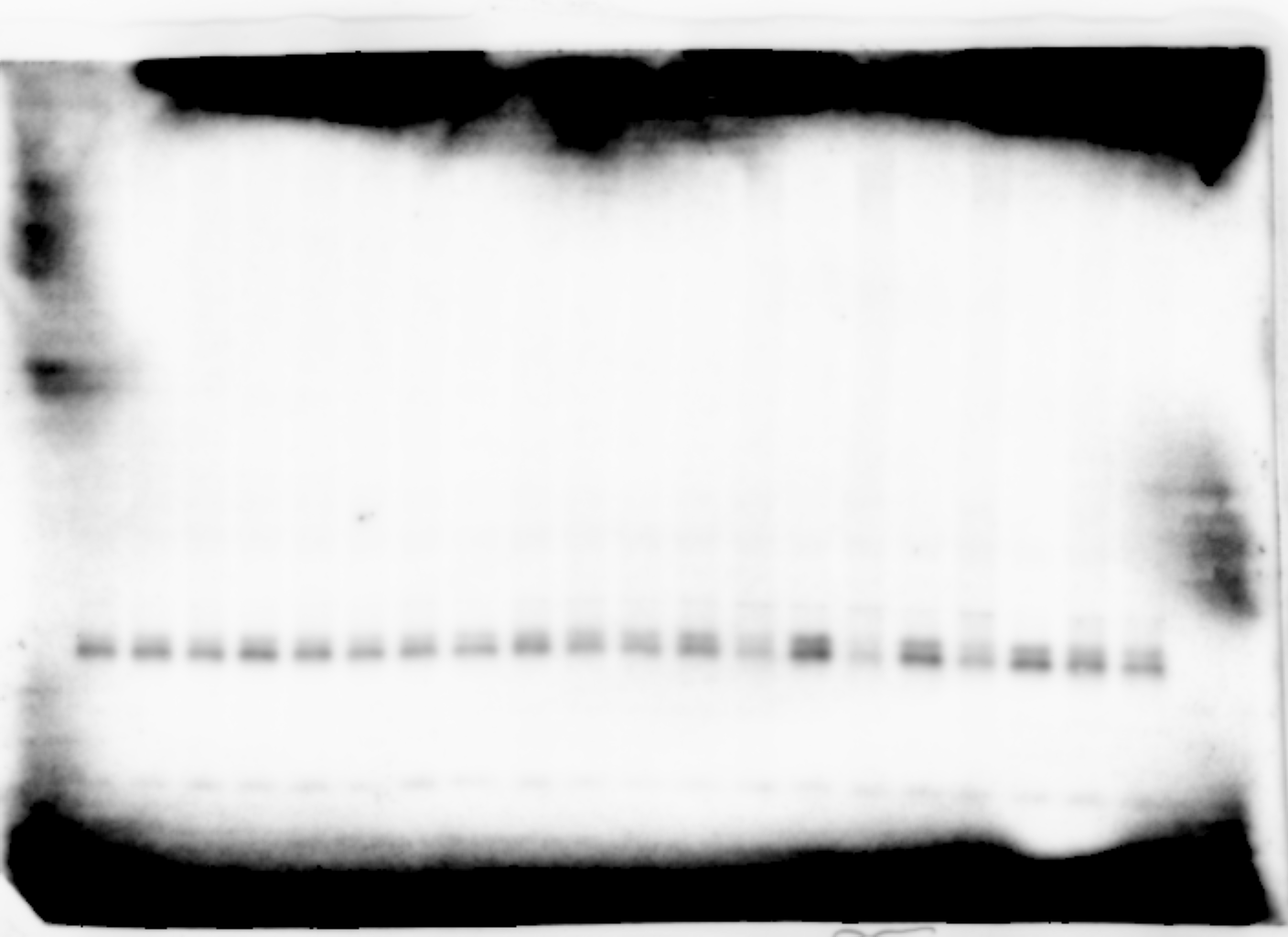

Supplement: Figure 5—source data 1. [file elife-80949-fig5-data1.zip › Figure 5 Source data/CITED4/CITED4/CITED4 LV BLot34 2018.09.07_11.22.12_Ch.tif]

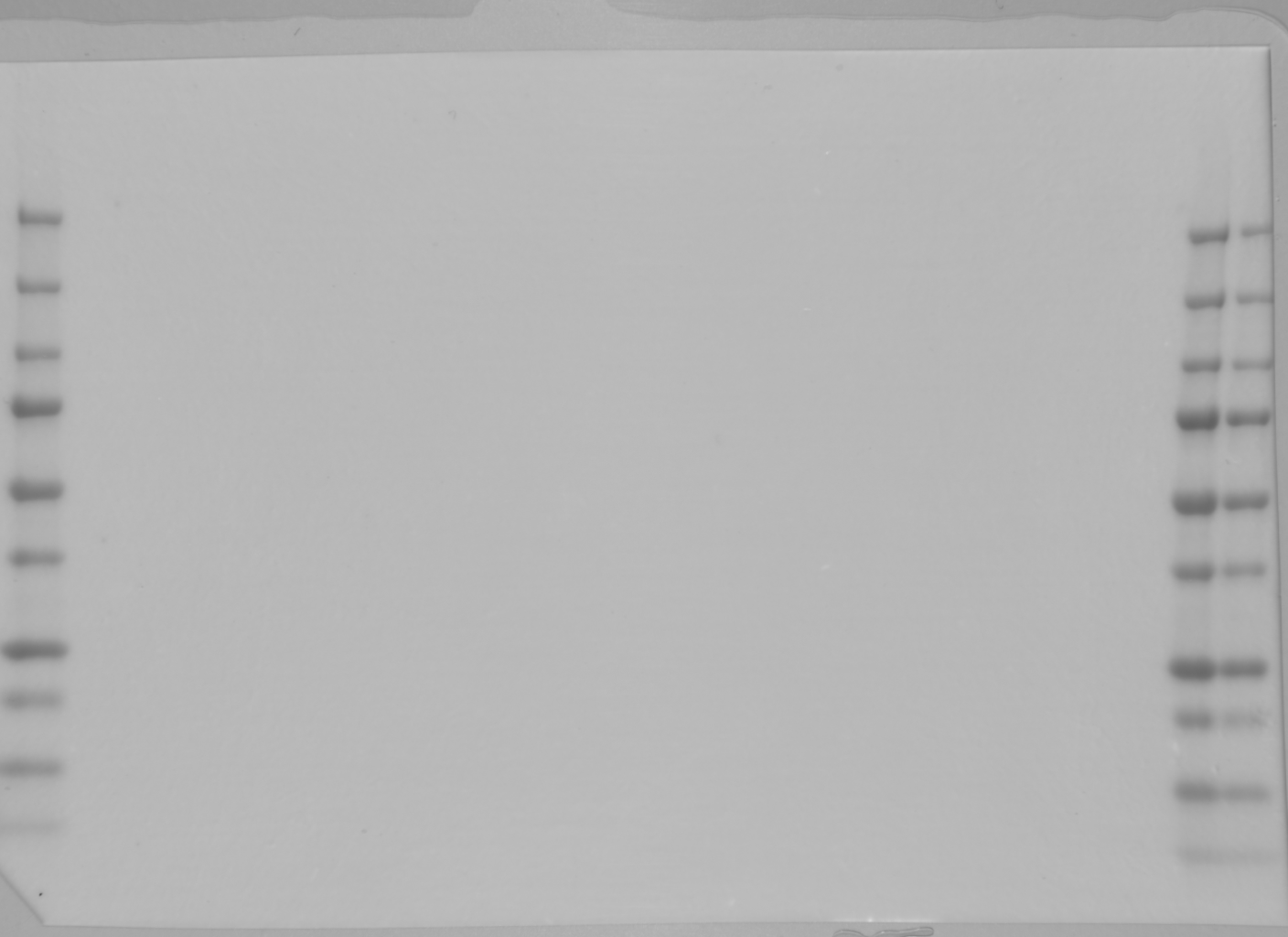

Supplement: Figure 5—source data 1. [file elife-80949-fig5-data1.zip › Figure 5 Source data/CITED4/CITED4/CITED4 LV BLot34 2018.09.07_11.22.12_Ch-Marker.tif]

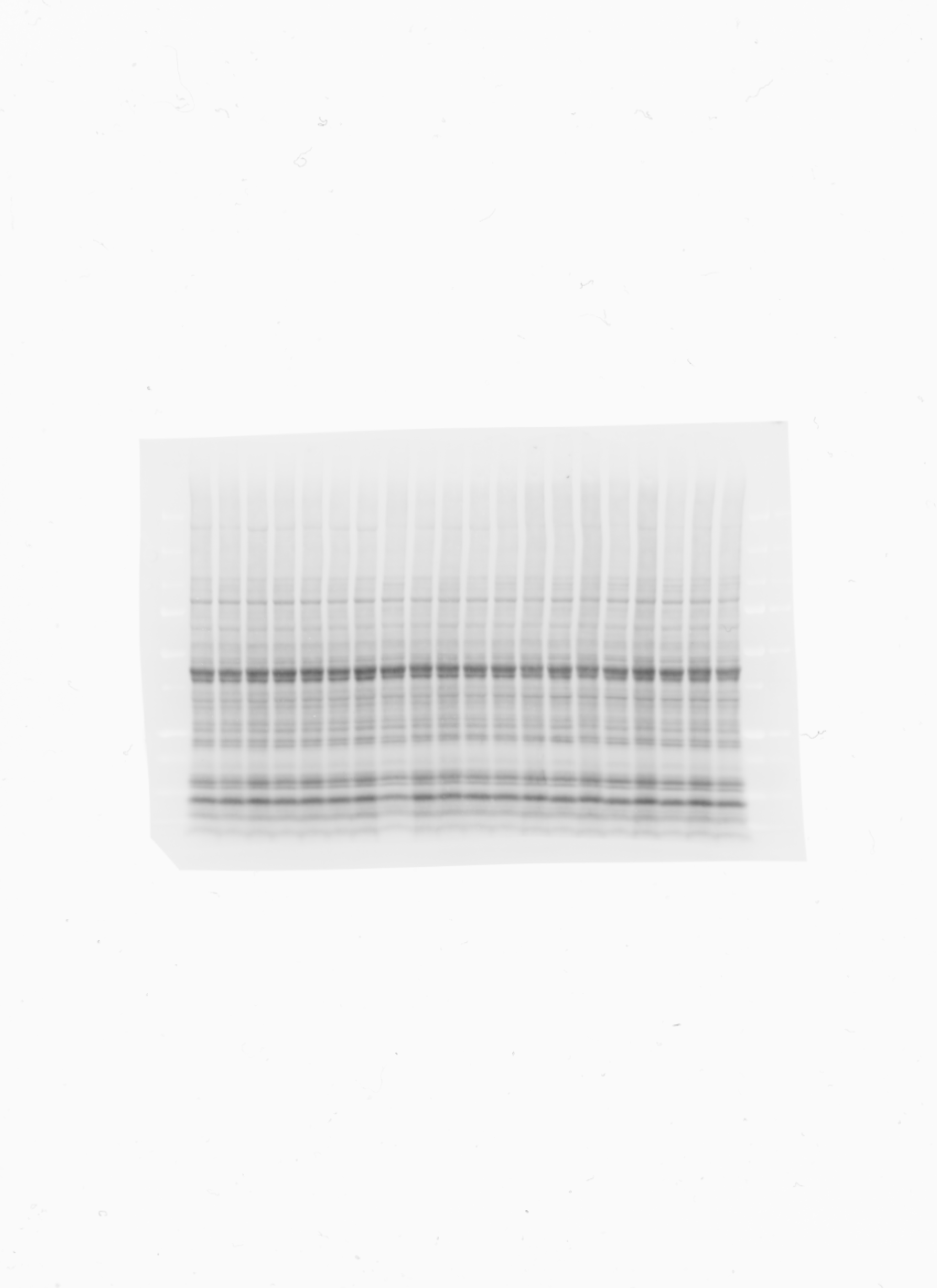

Supplement: Figure 5—source data 1. [file elife-80949-fig5-data1.zip › Figure 5 Source data/CITED4/Total Protein/DR T.Prot LV Blot34 2018.09.06_13.40.47_Fl-UV.tif]

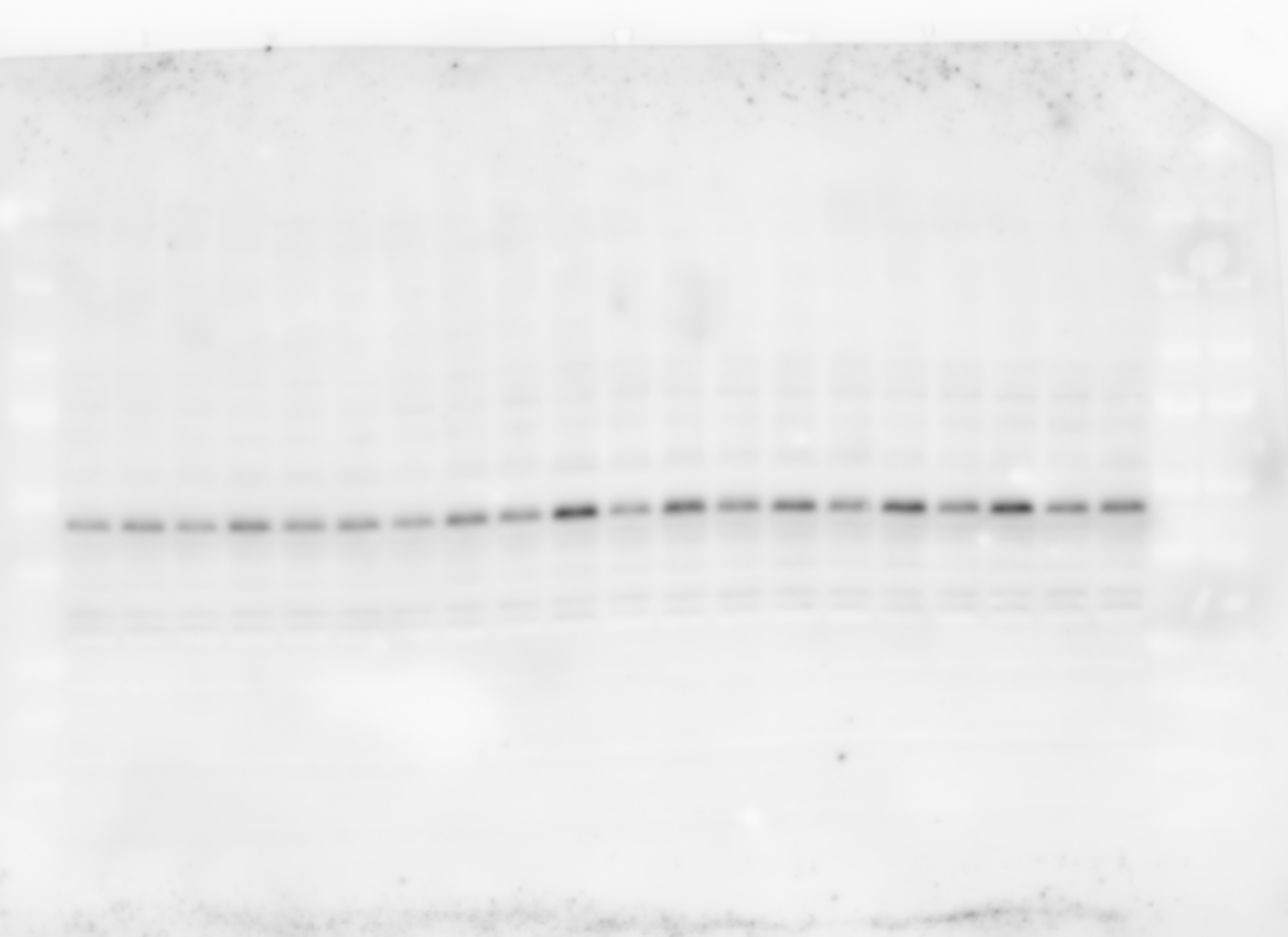

Supplement: Figure 5—source data 1. [file elife-80949-fig5-data1.zip › Figure 5 Source data/CREB1/CREB1/DR CREB1 Blot20 WPP 2018.04.20_12.23.30_Ch.tif]

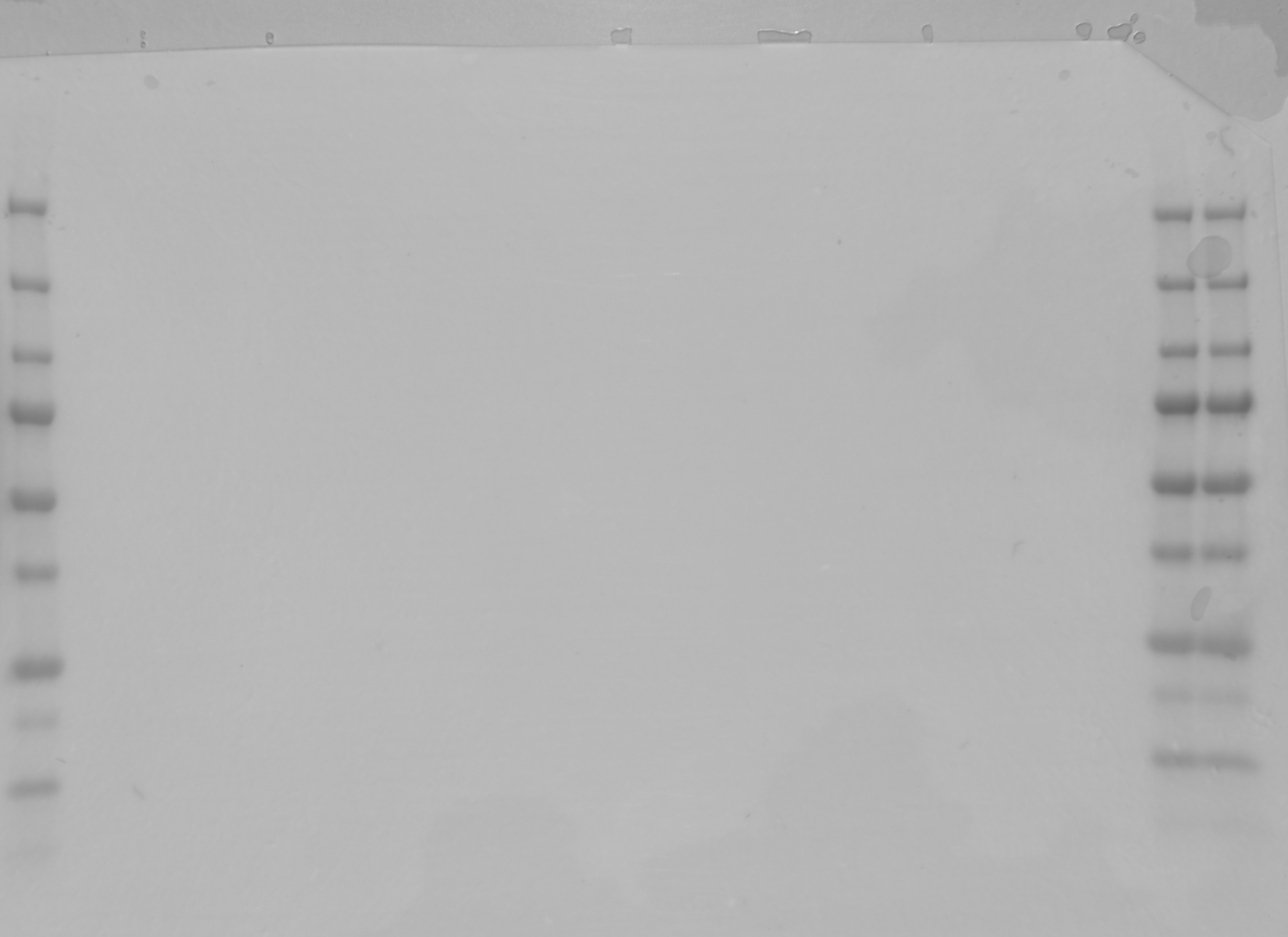

Supplement: Figure 5—source data 1. [file elife-80949-fig5-data1.zip › Figure 5 Source data/CREB1/CREB1/DR CREB1 Blot20 WPP 2018.04.20_12.23.30_Ch-Marker.tif]

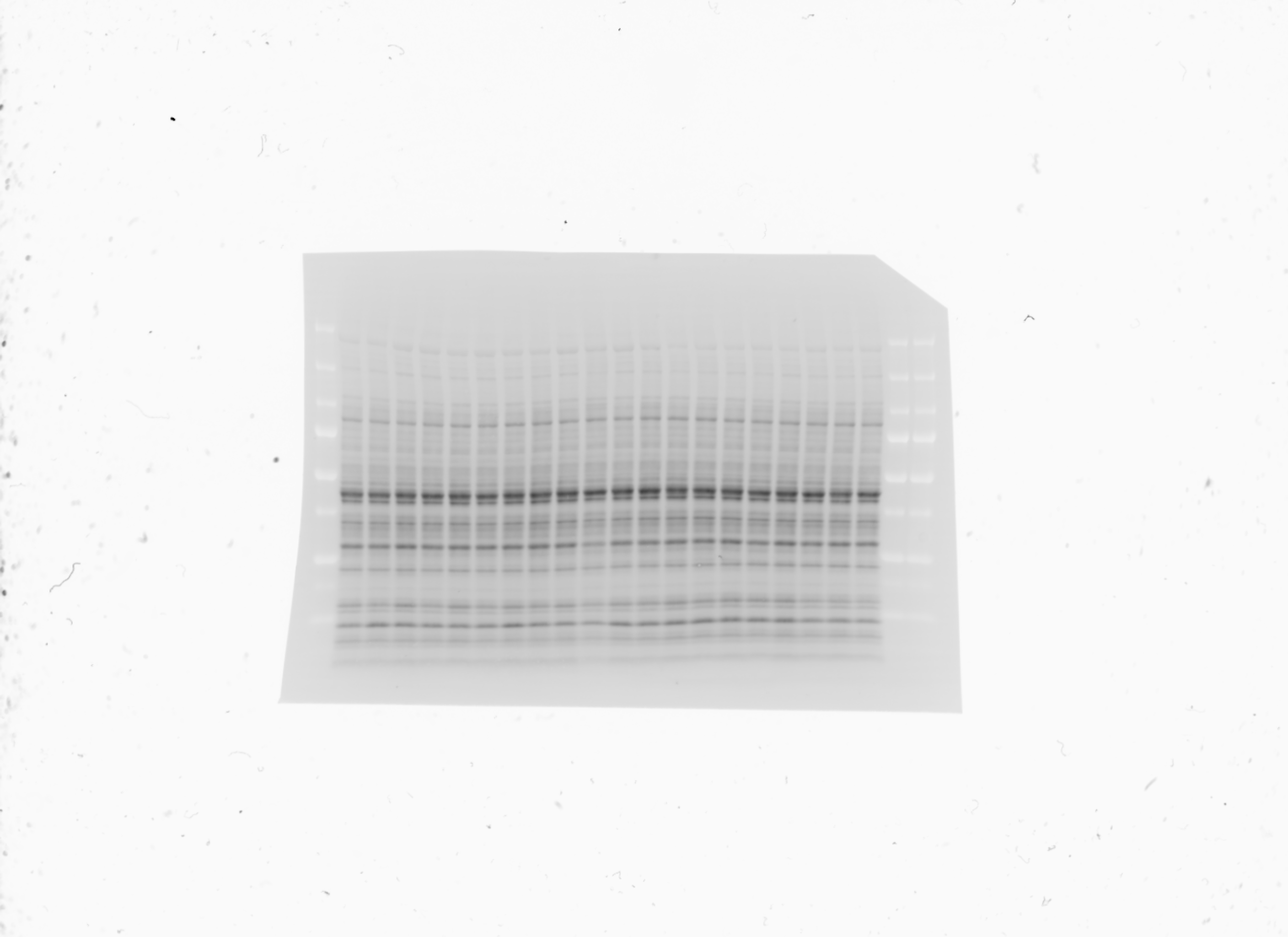

Supplement: Figure 5—source data 1. [file elife-80949-fig5-data1.zip › Figure 5 Source data/CREB1/Total Protein/DR T.Prot. LV Blt 20 2018.04.19_13.56.58_Fl-UV.tif]

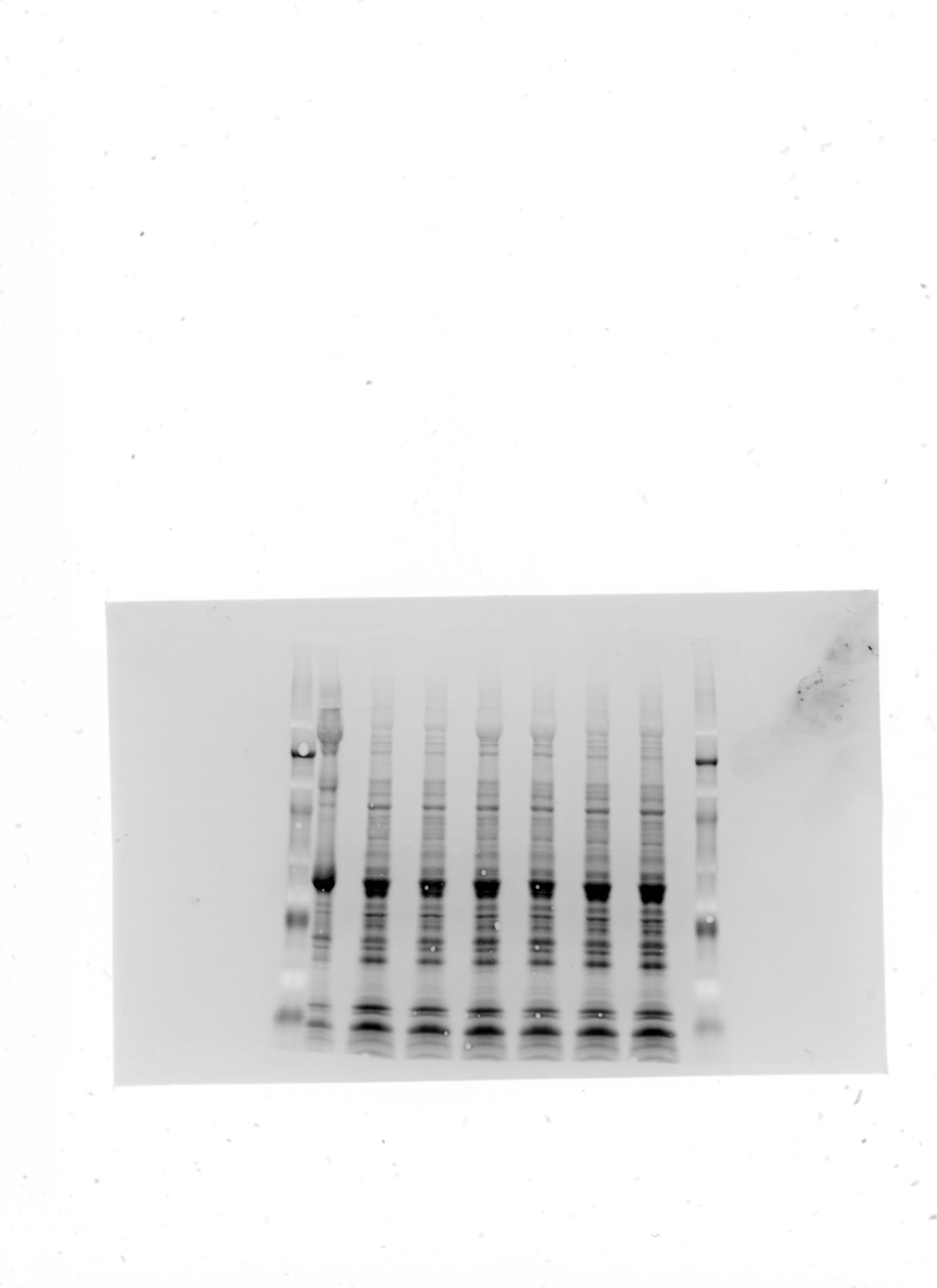

Supplement: Figure 5—source data 1. [file elife-80949-fig5-data1.zip › Figure 5 Source data/Fig5A - Puromycin/MGP_Puromycin Total Protein/MGP_Puromycin Total Protein.jpg]

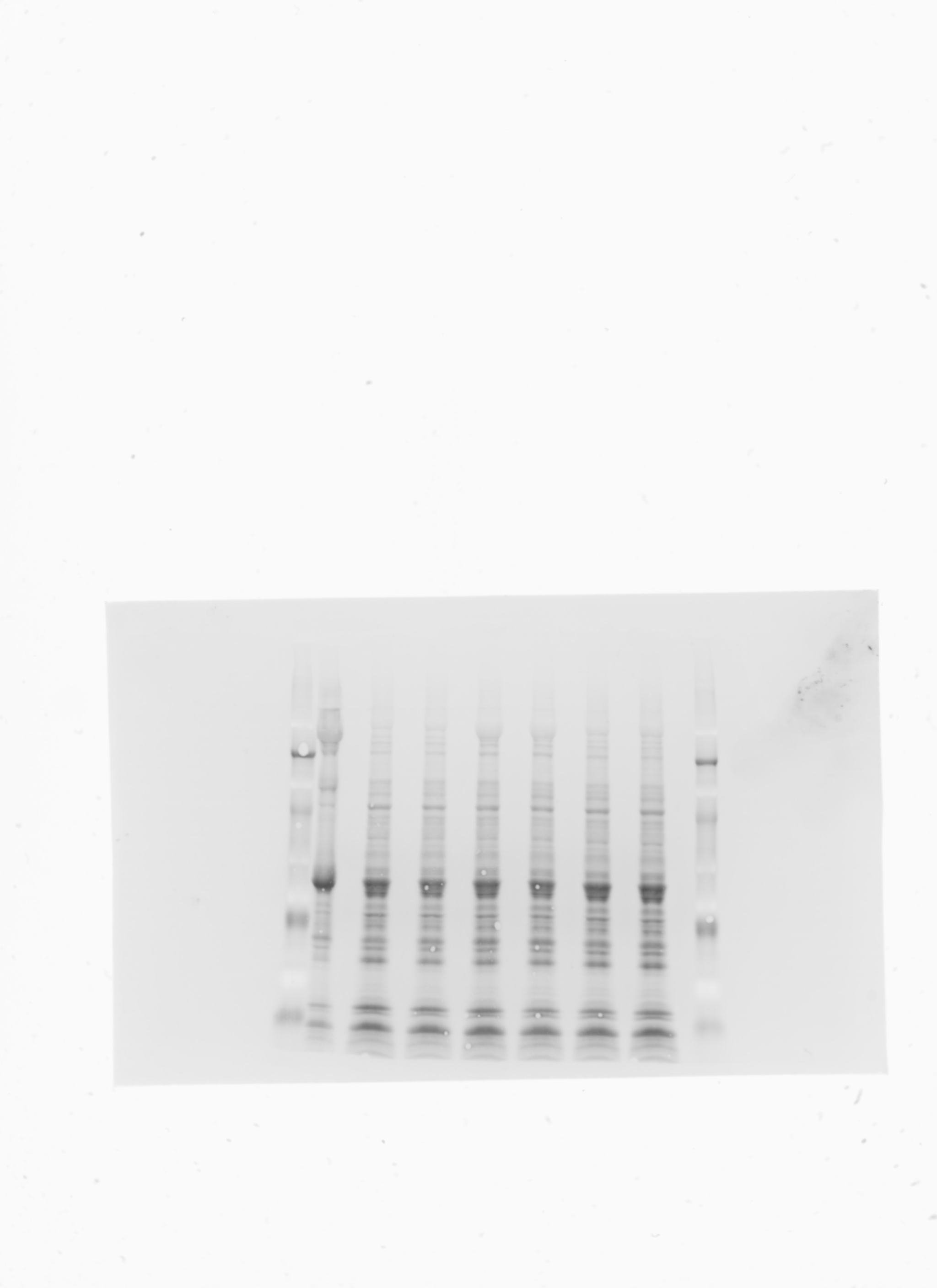

Supplement: Figure 5—source data 1. [file elife-80949-fig5-data1.zip › Figure 5 Source data/Fig5A - Puromycin/MGP_Puromycin Total Protein/MGP_Puromycin Total Protein.tif]

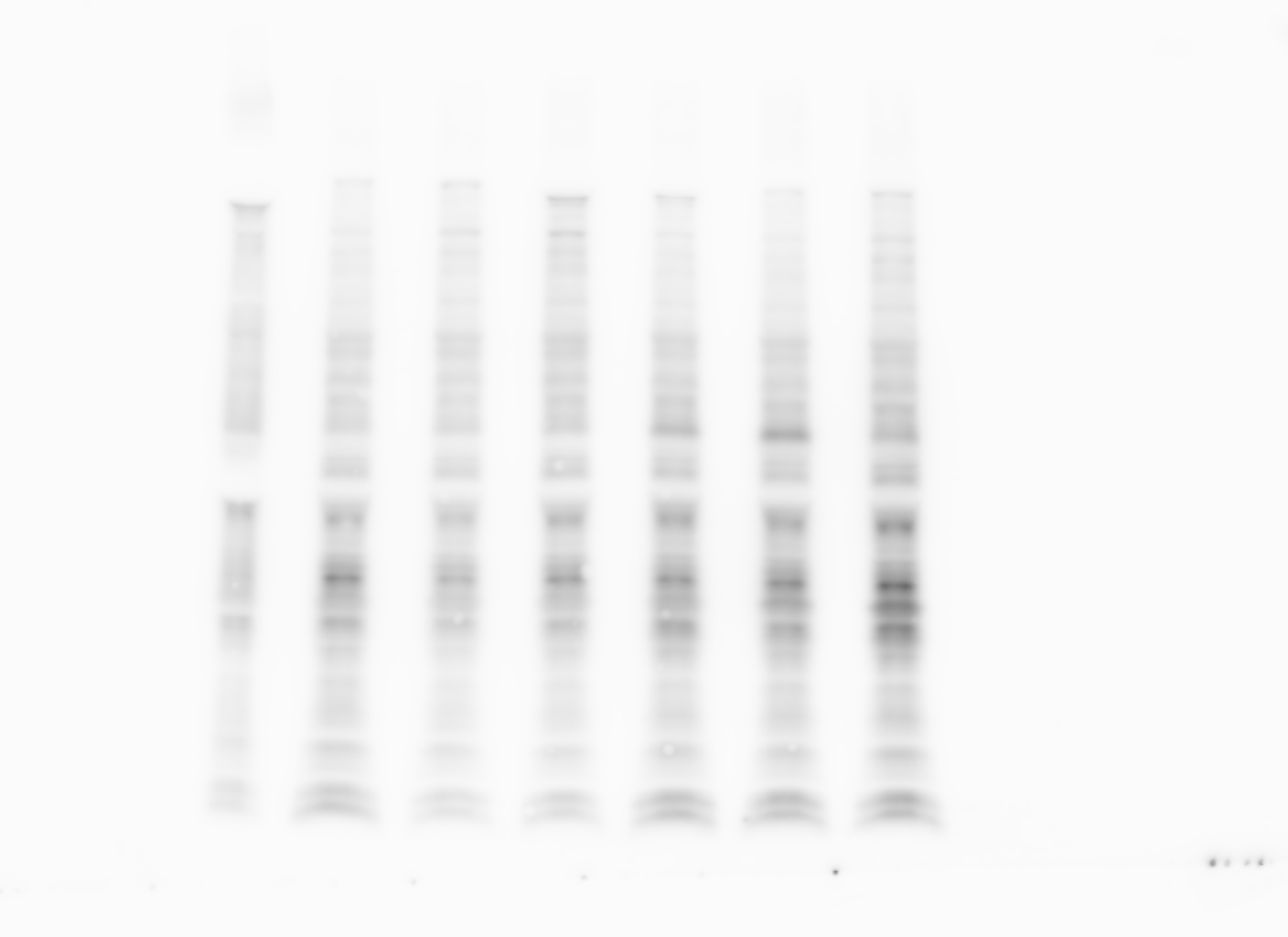

Supplement: Figure 5—source data 1. [file elife-80949-fig5-data1.zip › Figure 5 Source data/Fig5A - Puromycin/MGP_Puromycin/MGP_Puromycin_Ch.tif]

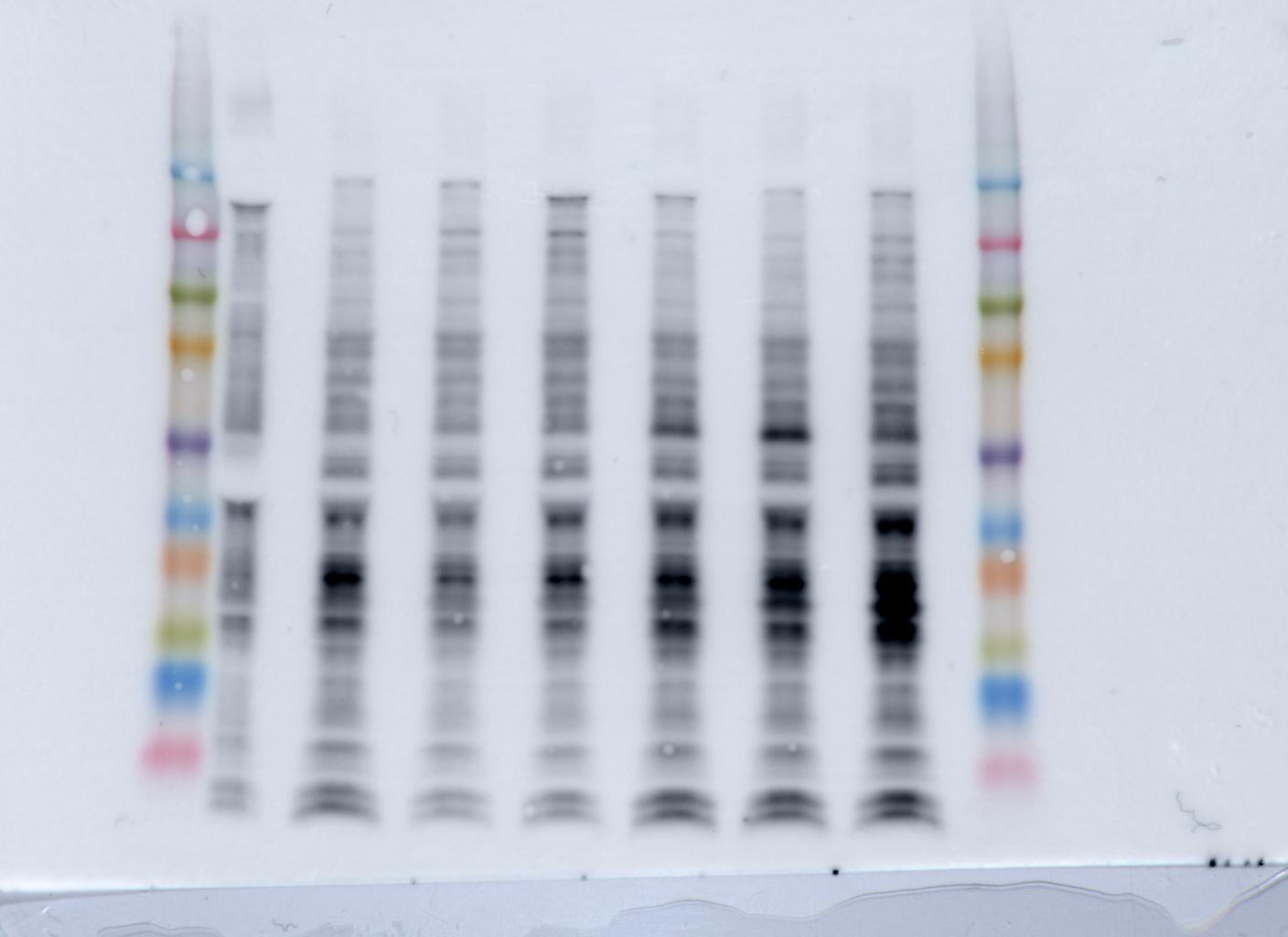

Supplement: Figure 5—source data 1. [file elife-80949-fig5-data1.zip › Figure 5 Source data/Fig5A - Puromycin/MGP_Puromycin/MGP_Puromycin_Ch+Marker.jpg]

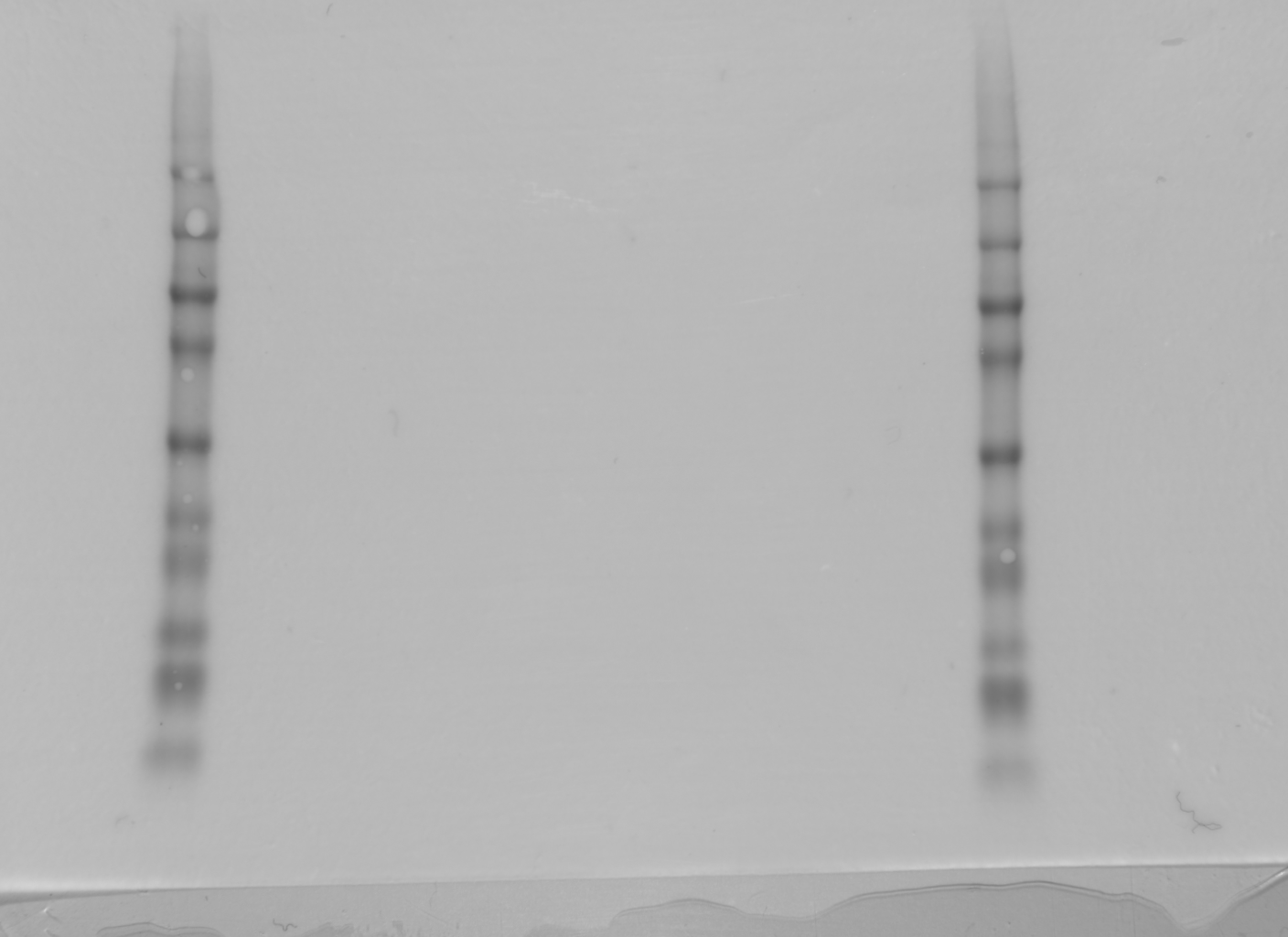

Supplement: Figure 5—source data 1. [file elife-80949-fig5-data1.zip › Figure 5 Source data/Fig5A - Puromycin/MGP_Puromycin/MGP_Puromycin_Marker.tif]

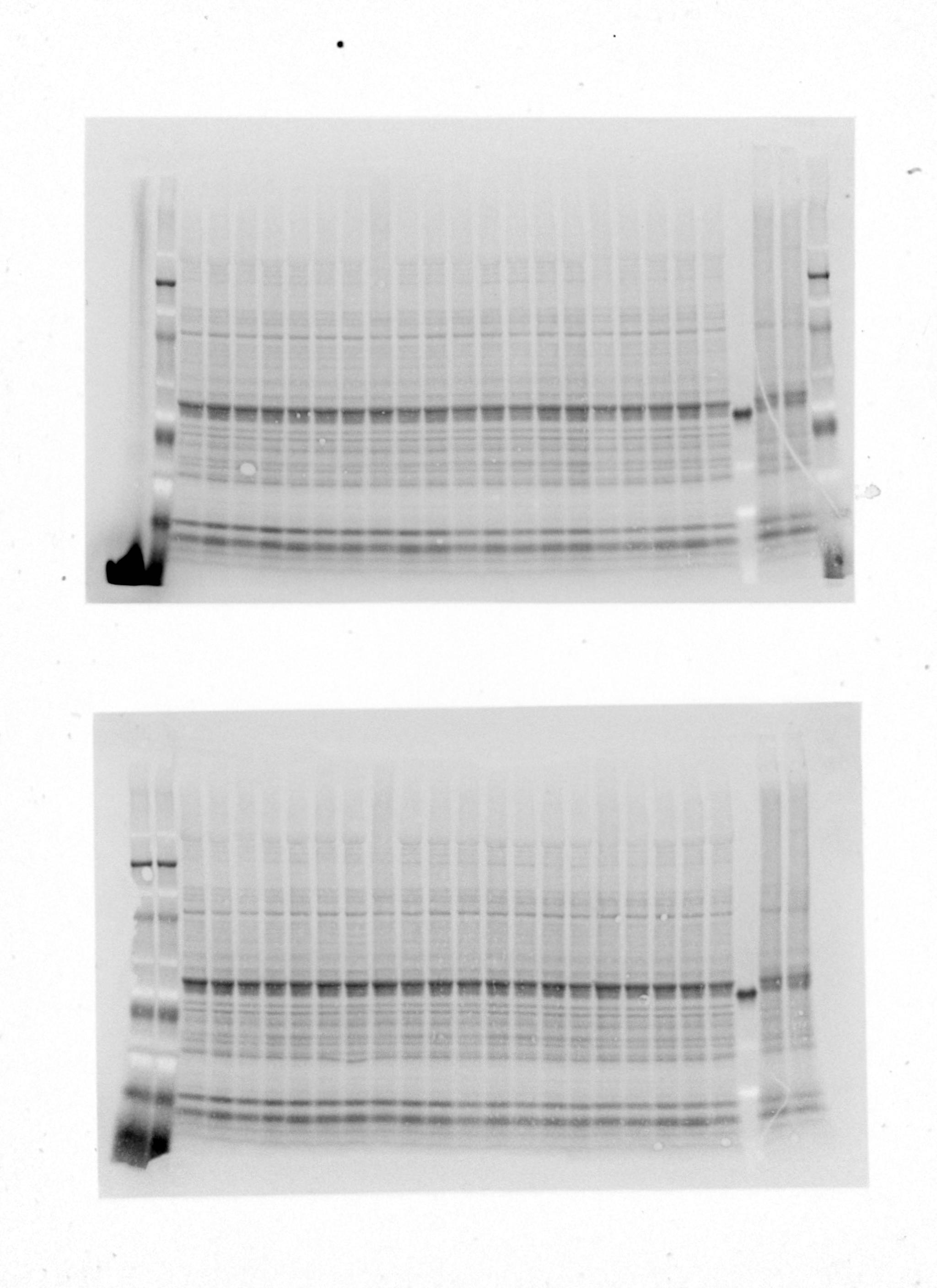

Supplement: Figure 5—source data 1. [file elife-80949-fig5-data1.zip › Figure 5 Source data/Fig5F - MNK1/MGP_MNK1 Total Protein/MGP_MNK1 Total Protein.jpg]

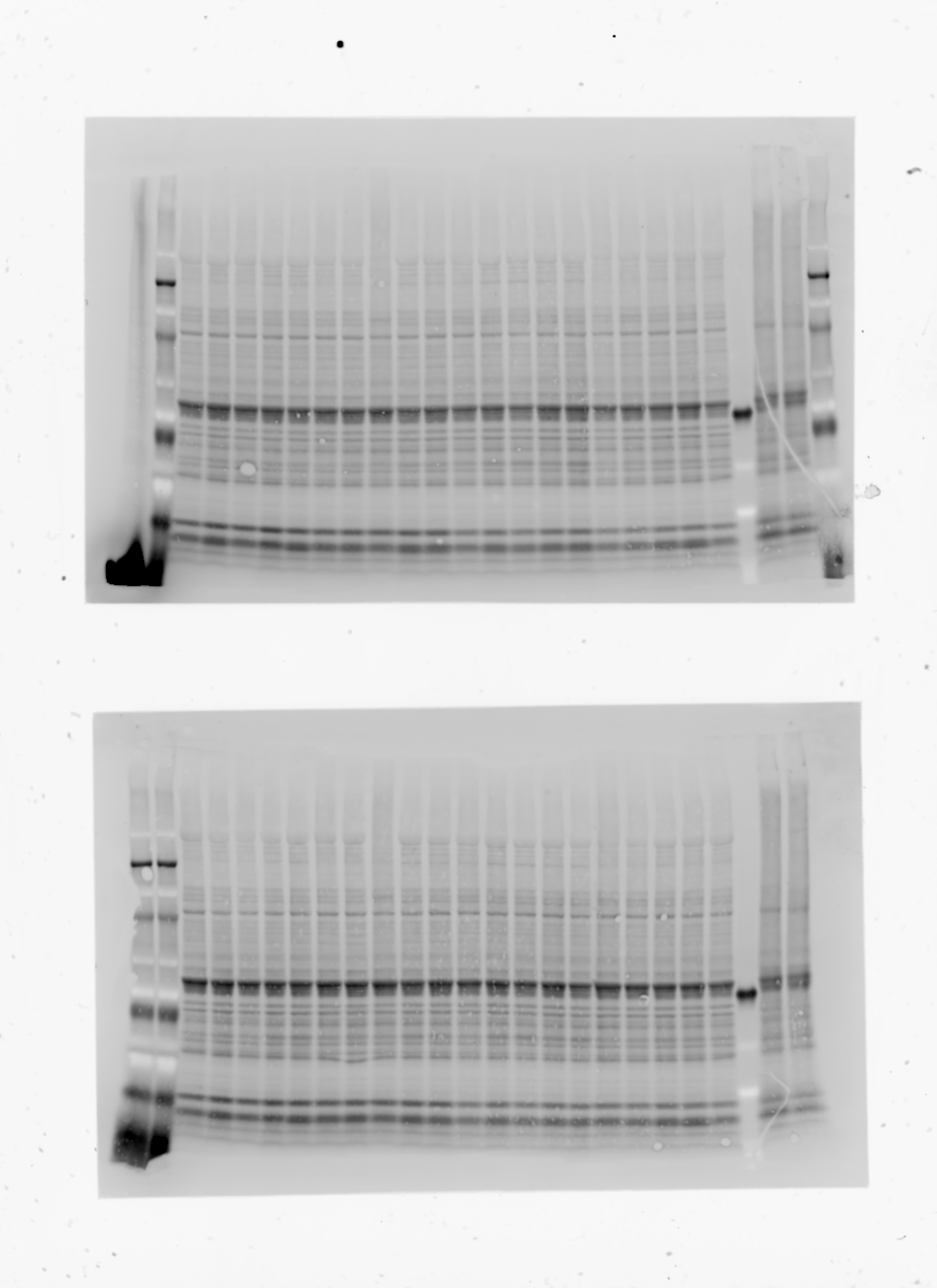

Supplement: Figure 5—source data 1. [file elife-80949-fig5-data1.zip › Figure 5 Source data/Fig5F - MNK1/MGP_MNK1 Total Protein/MGP_MNK1 Total Protein.tif]

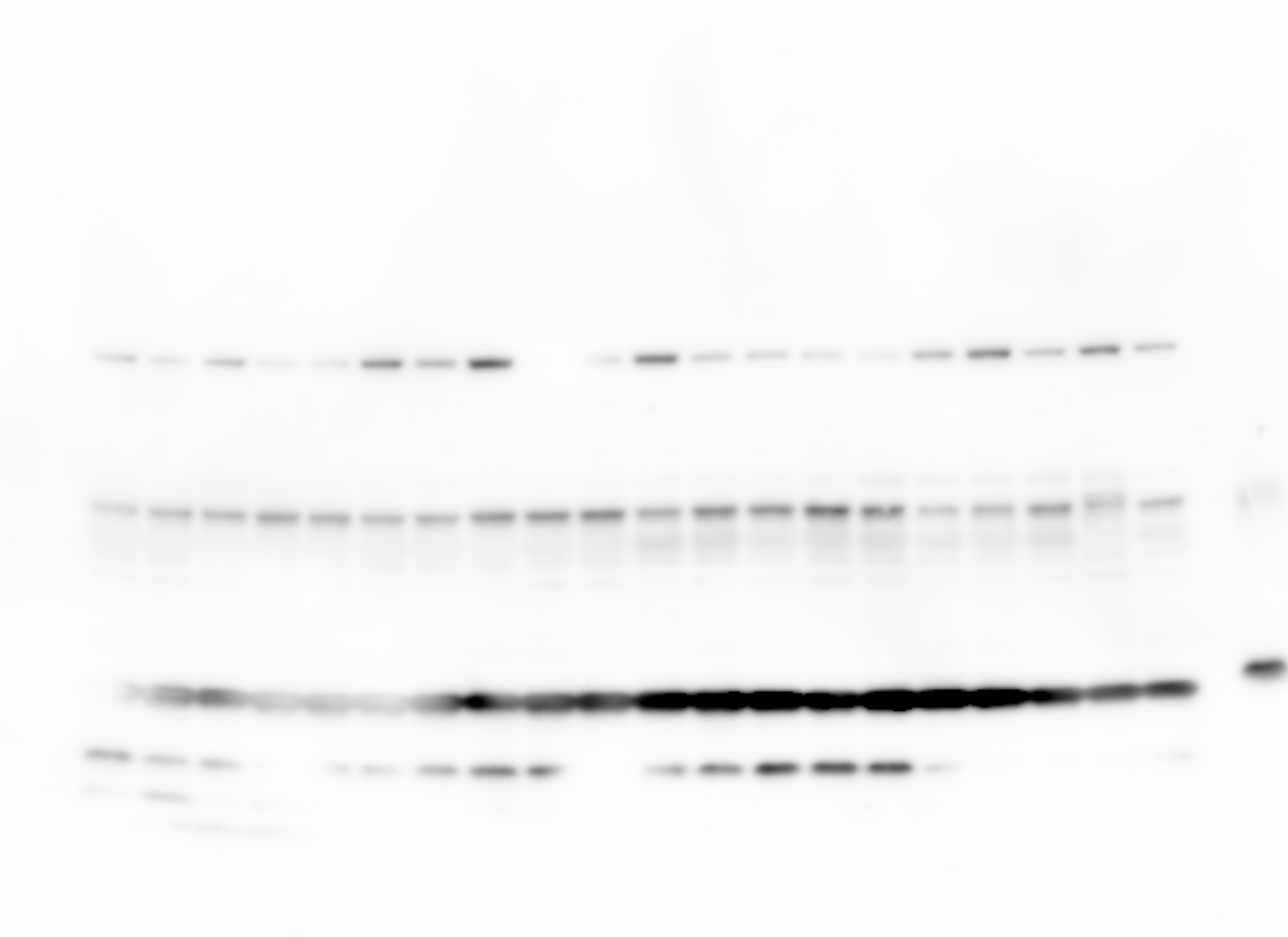

Supplement: Figure 5—source data 1. [file elife-80949-fig5-data1.zip › Figure 5 Source data/Fig5F - MNK1/MGP_MNK1/MGP_MNK1_Ch.tif]

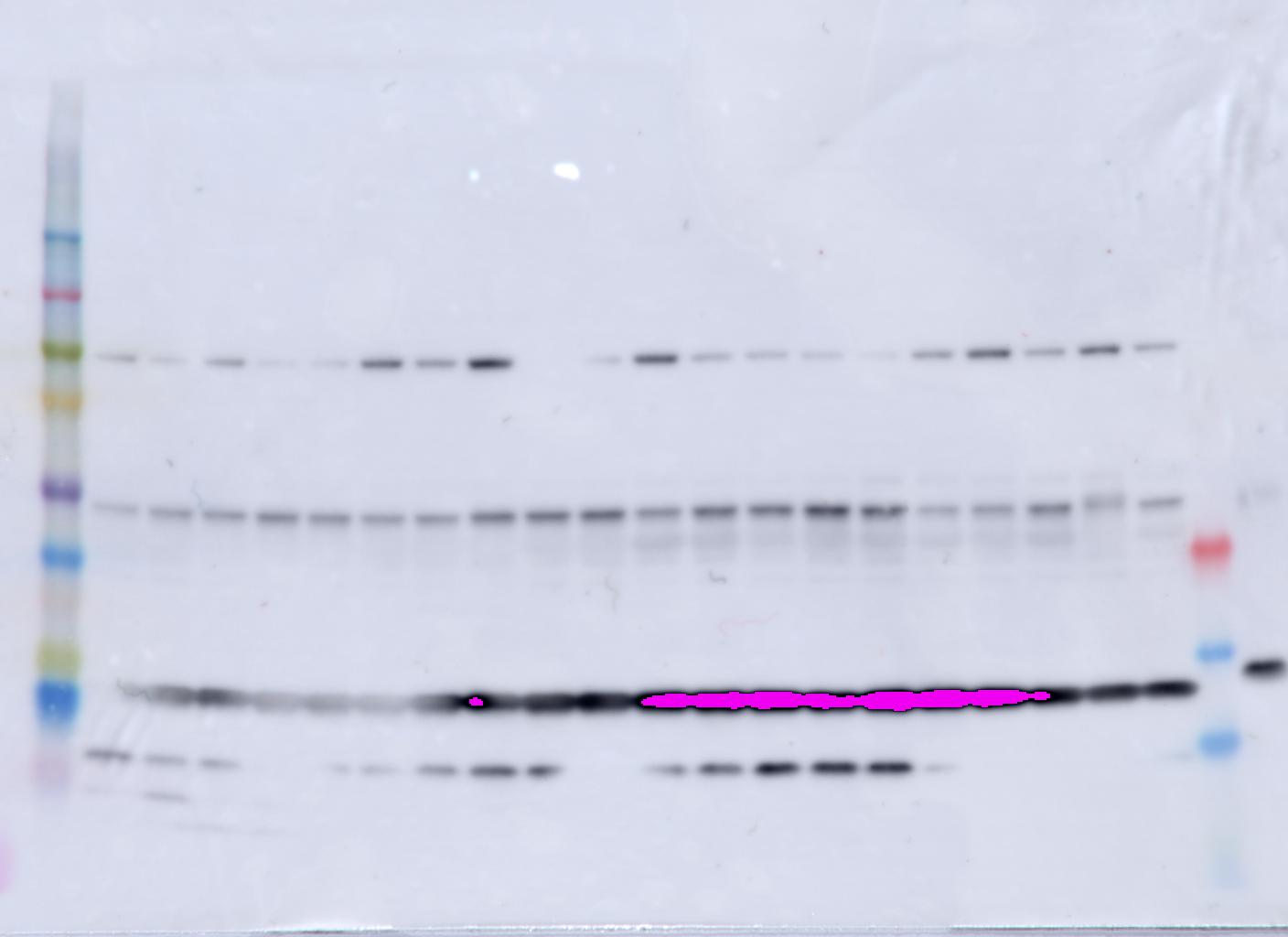

Supplement: Figure 5—source data 1. [file elife-80949-fig5-data1.zip › Figure 5 Source data/Fig5F - MNK1/MGP_MNK1/MGP_MNK1_Ch+Marker.jpg]

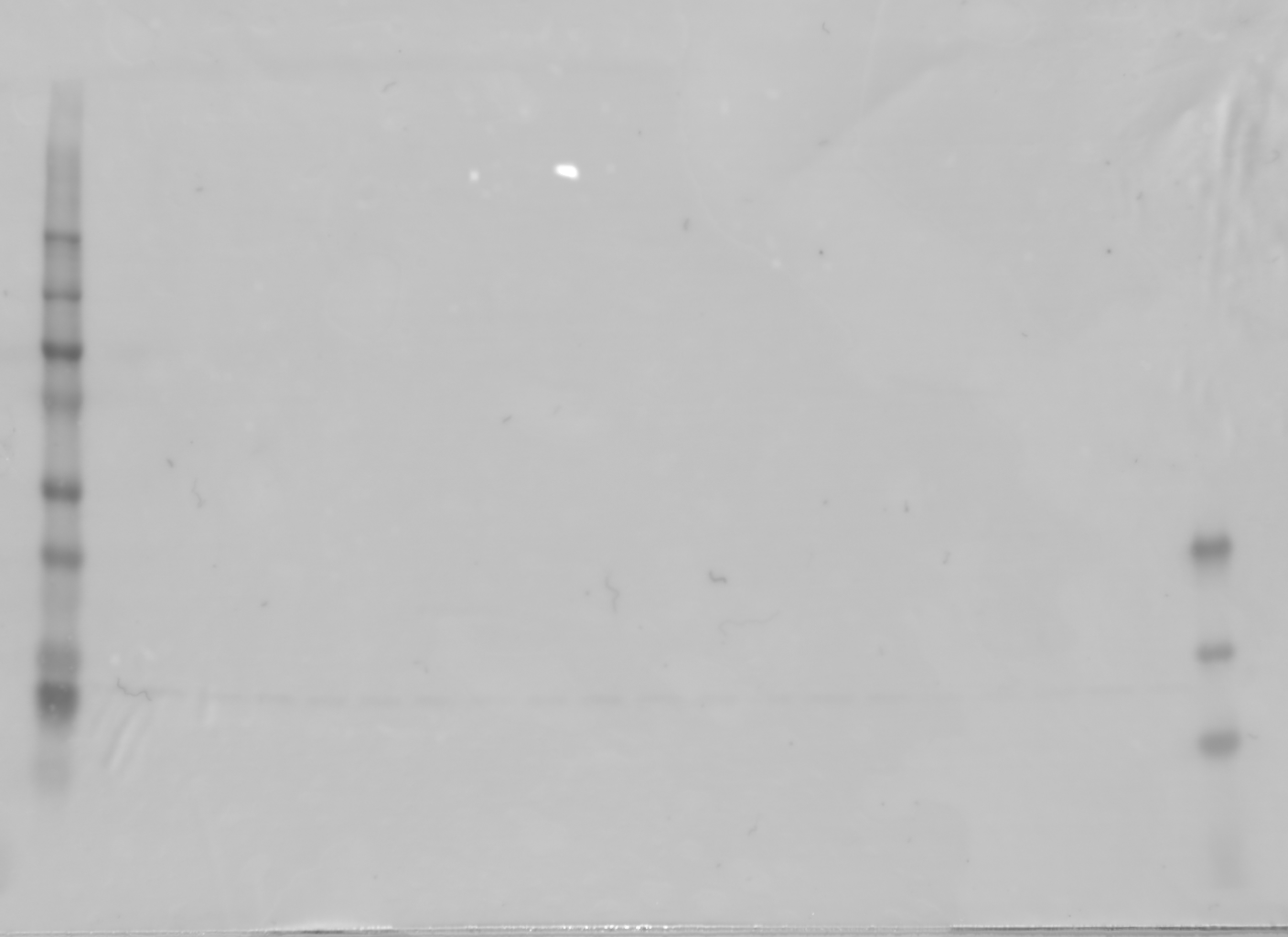

Supplement: Figure 5—source data 1. [file elife-80949-fig5-data1.zip › Figure 5 Source data/Fig5F - MNK1/MGP_MNK1/MGP_MNK1_Marker.tif]

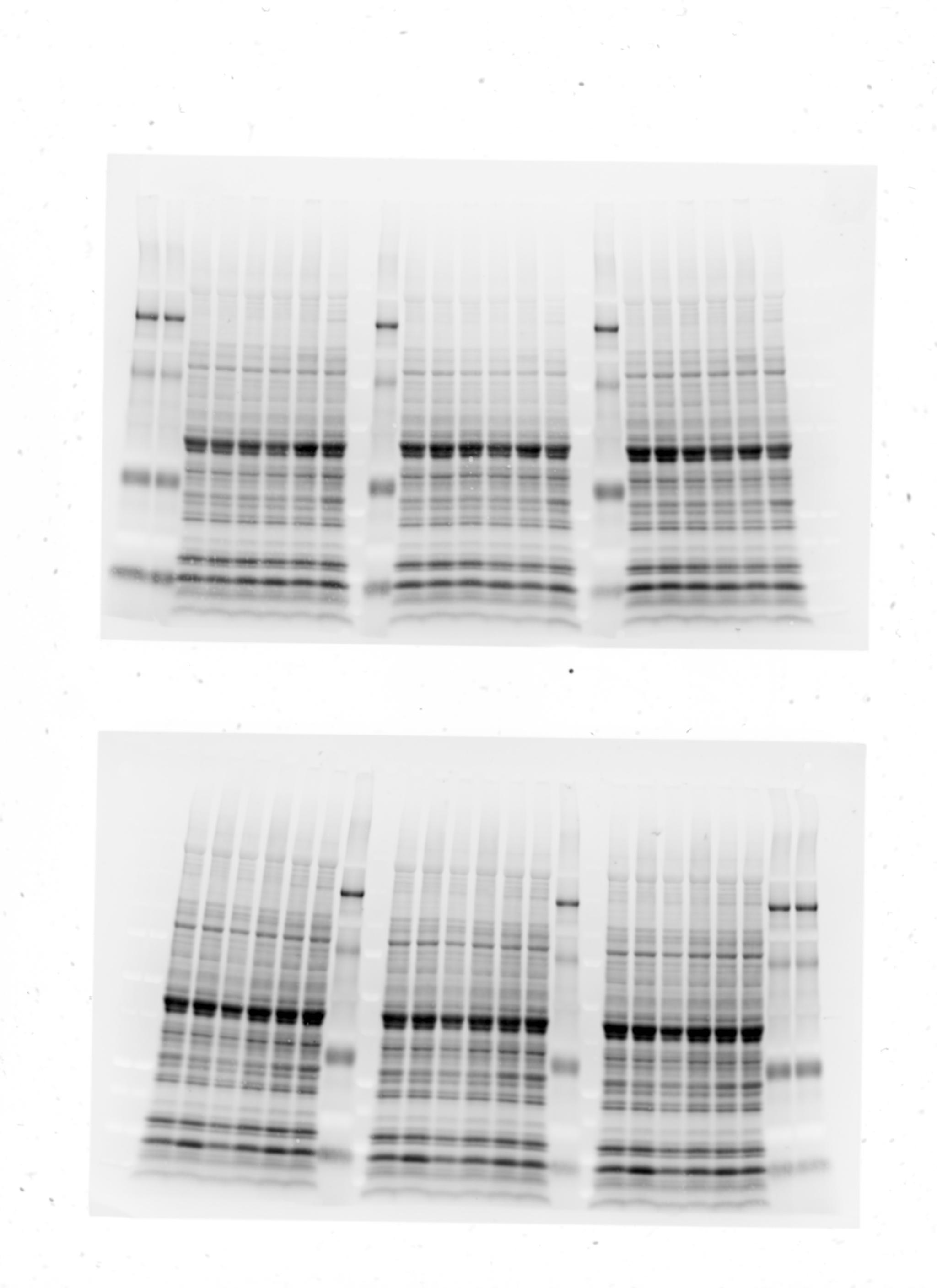

Supplement: Figure 5—source data 1. [file elife-80949-fig5-data1.zip › Figure 5 Source data/Fig5G - MSK1/MGP_MSK1 Total Protein/MGP_MSK1 Total Protein.jpg]

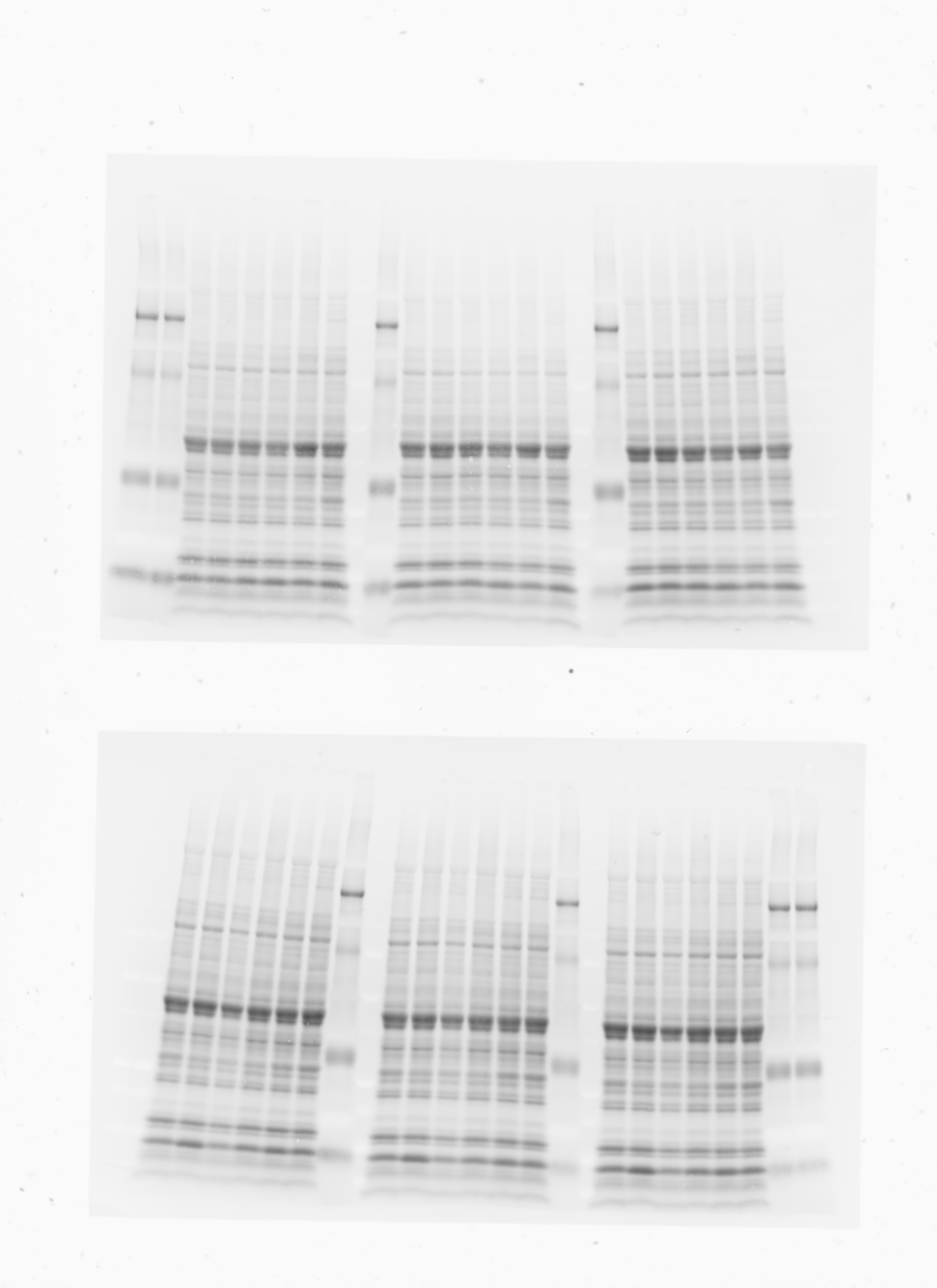

Supplement: Figure 5—source data 1. [file elife-80949-fig5-data1.zip › Figure 5 Source data/Fig5G - MSK1/MGP_MSK1 Total Protein/MGP_MSK1 Total Protein.tif]

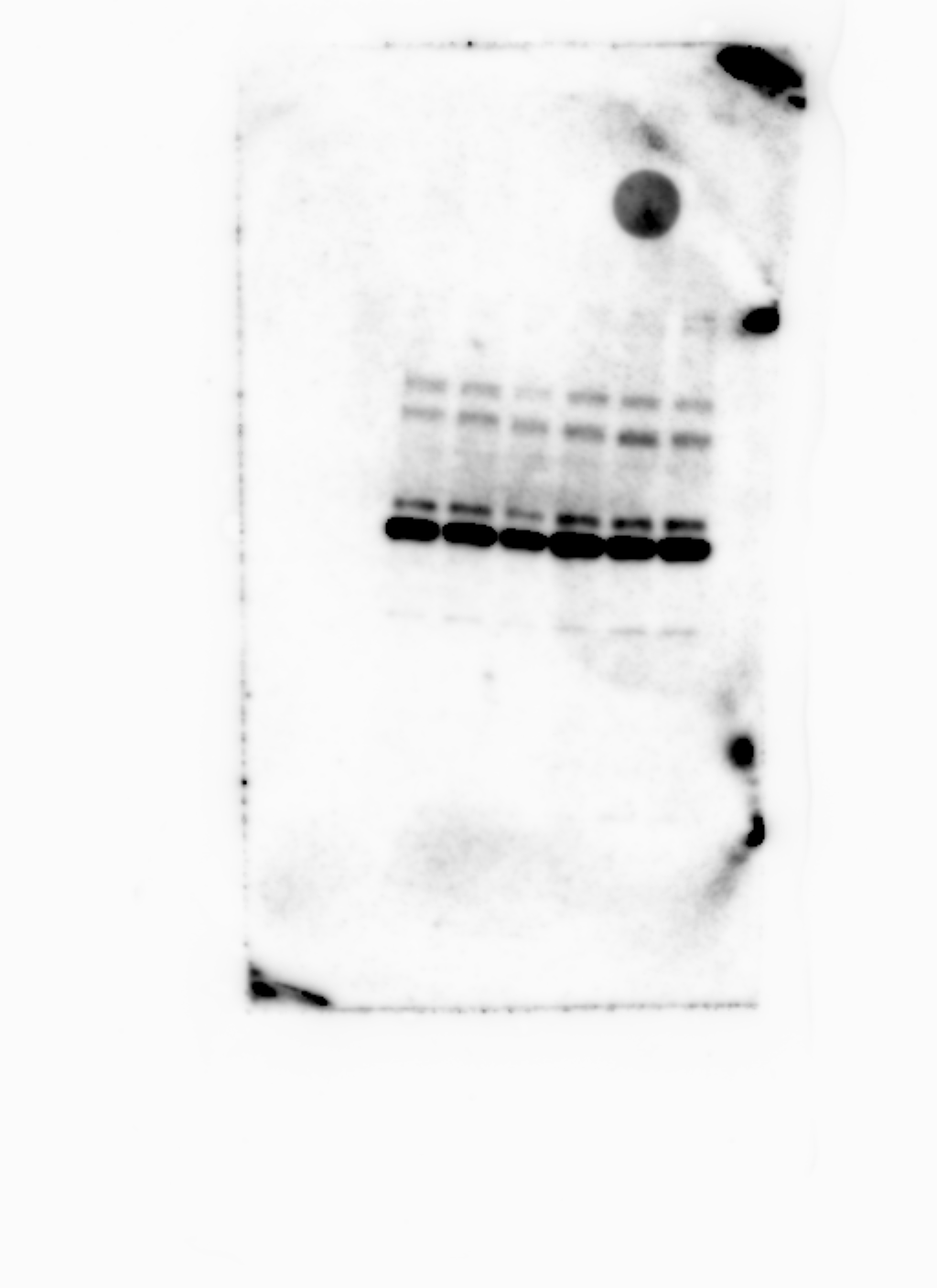

Supplement: Figure 5—source data 1. [file elife-80949-fig5-data1.zip › Figure 5 Source data/Fig5G - MSK1/MGP_MSK1/MGP_MSK1_Ch.tif]

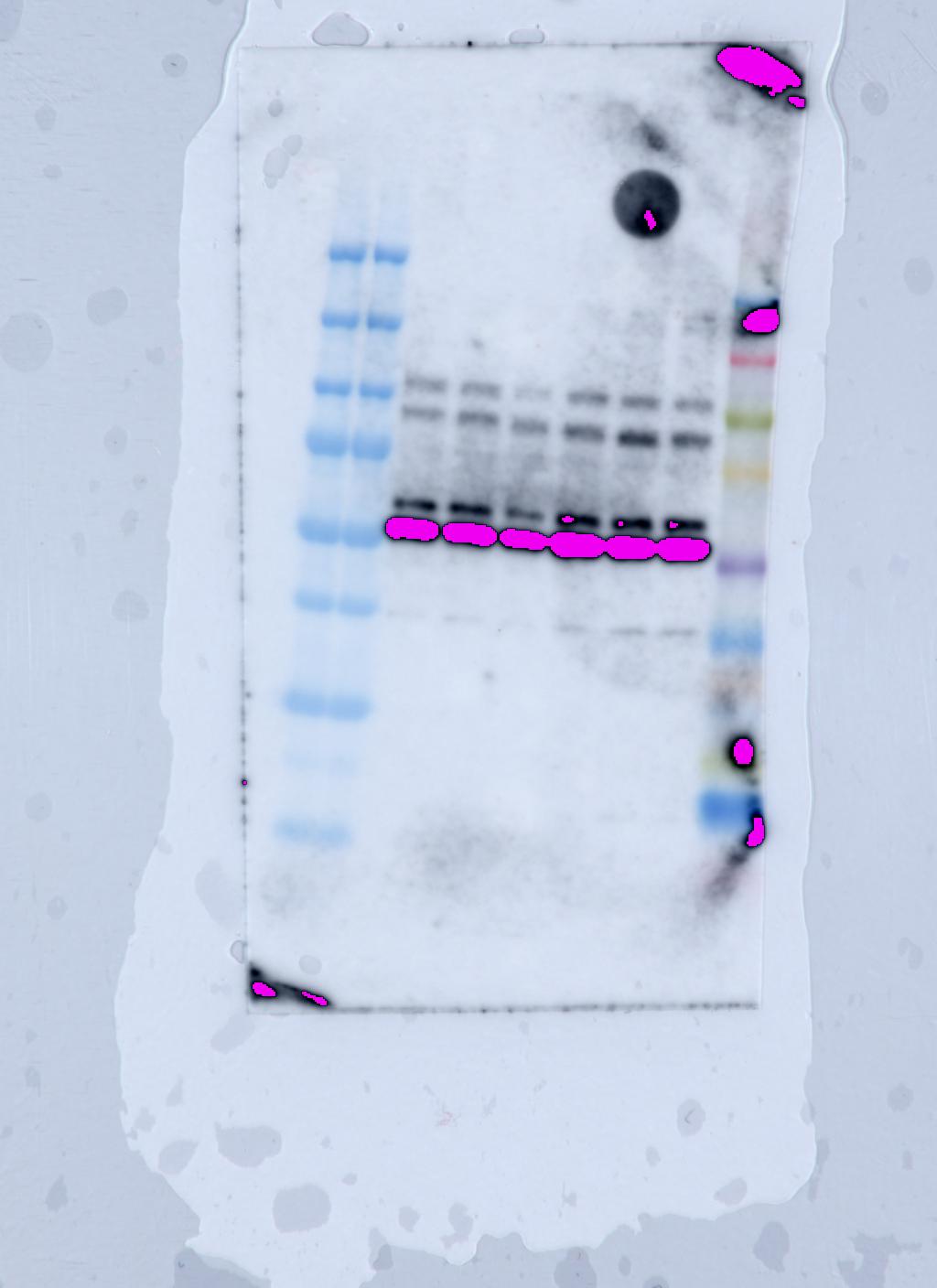

Supplement: Figure 5—source data 1. [file elife-80949-fig5-data1.zip › Figure 5 Source data/Fig5G - MSK1/MGP_MSK1/MGP_MSK1_Ch+Marker.jpg]

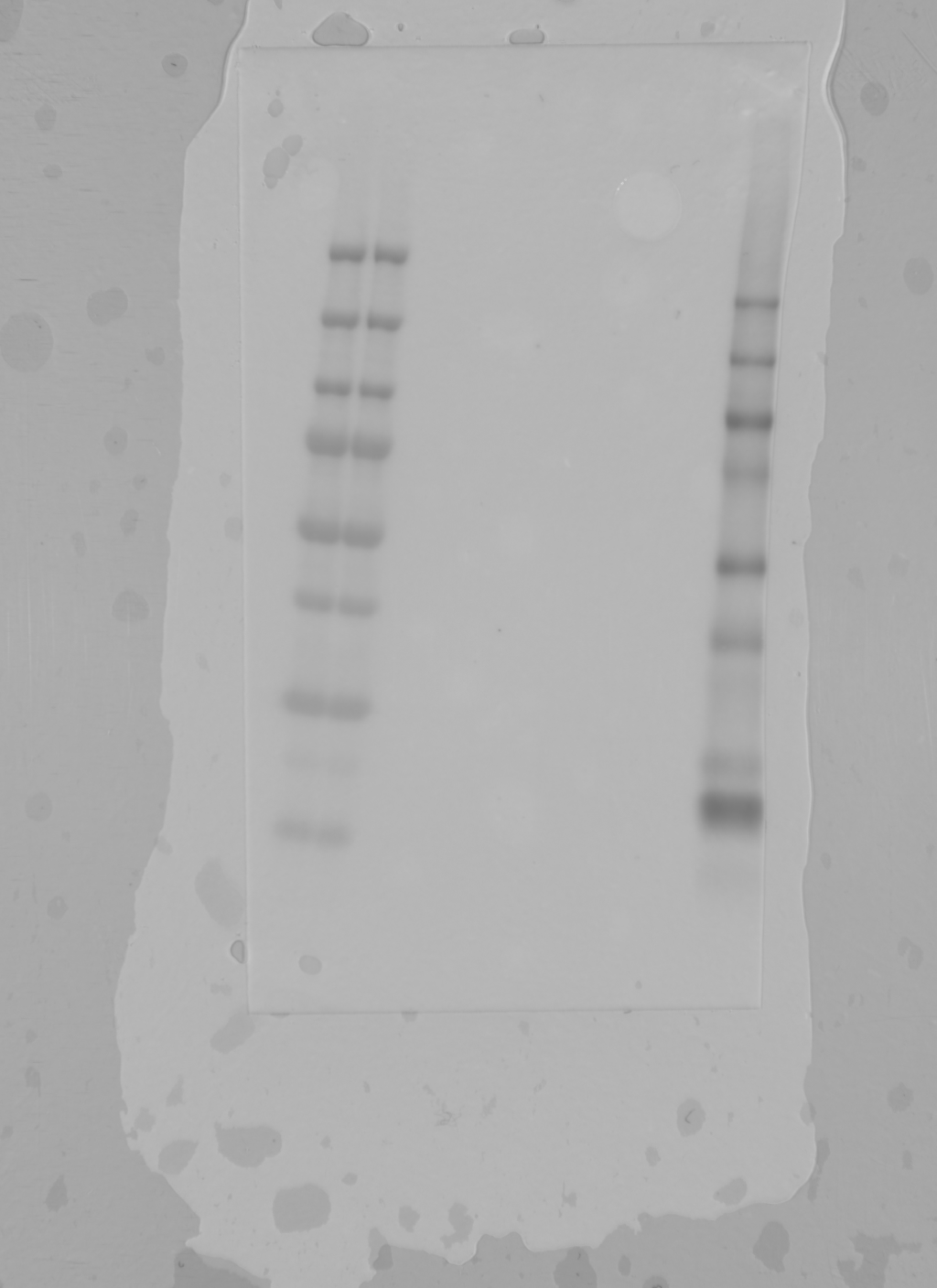

Supplement: Figure 5—source data 1. [file elife-80949-fig5-data1.zip › Figure 5 Source data/Fig5G - MSK1/MGP_MSK1/MGP_MSK1_Marker.tif]

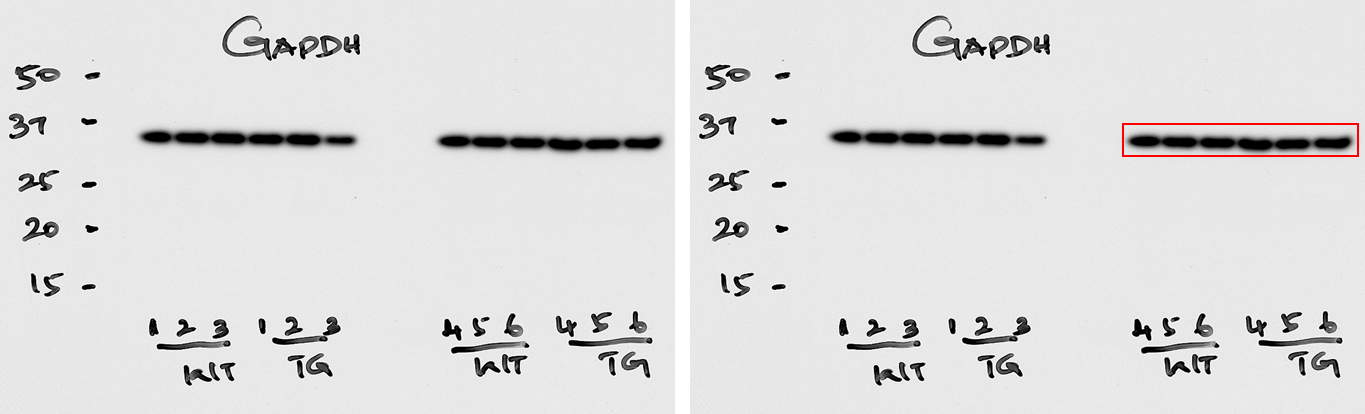

Supplement: Figure 5—source data 1. [file elife-80949-fig5-data1.zip › Figure 5 Source data/Figure 5B_GAPDH.tif]

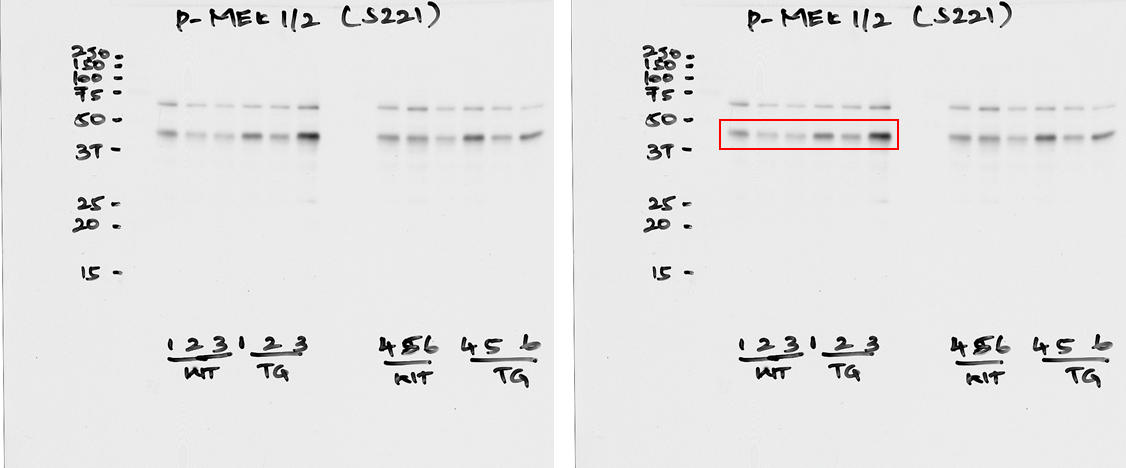

Supplement: Figure 5—source data 1. [file elife-80949-fig5-data1.zip › Figure 5 Source data/Figure 5B_p_MEK1_2_Ser221.tif]

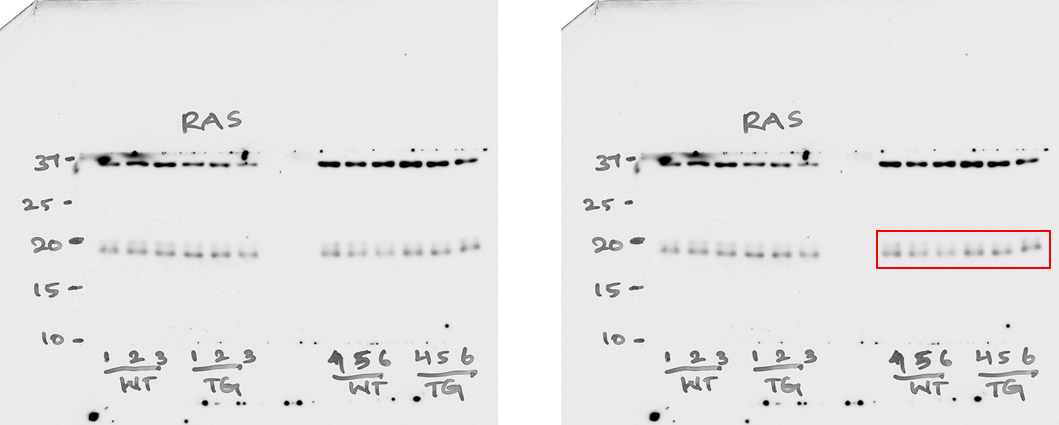

Supplement: Figure 5—source data 1. [file elife-80949-fig5-data1.zip › Figure 5 Source data/Figure 5B_P21 Ras.tif]

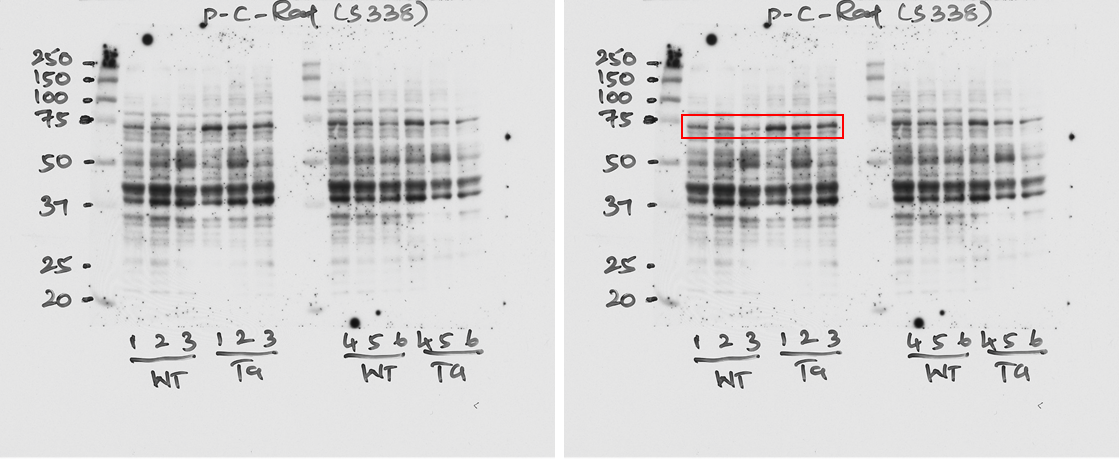

Supplement: Figure 5—source data 1. [file elife-80949-fig5-data1.zip › Figure 5 Source data/Figure 5B_p-c-Raf_Ser338.tif]

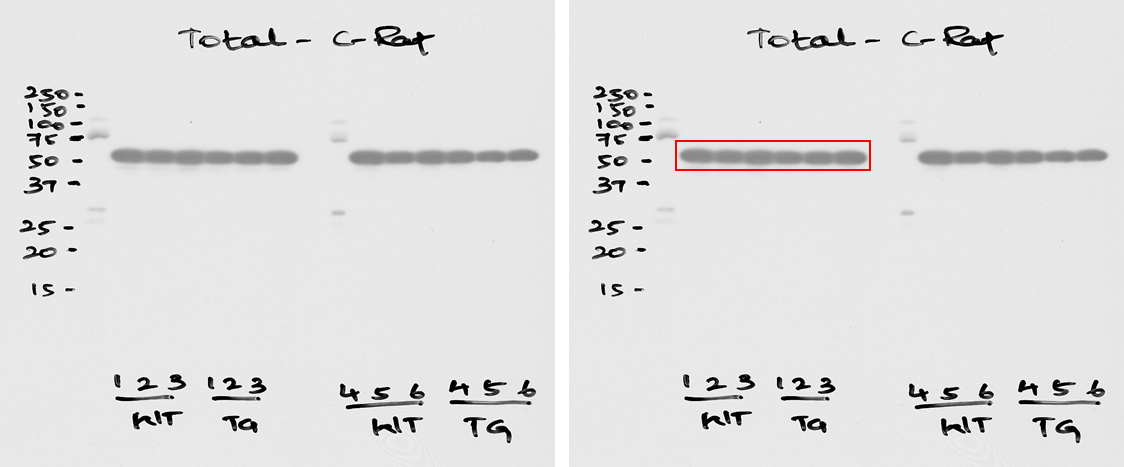

Supplement: Figure 5—source data 1. [file elife-80949-fig5-data1.zip › Figure 5 Source data/Figure 5B_Total_c-Raf.tif]

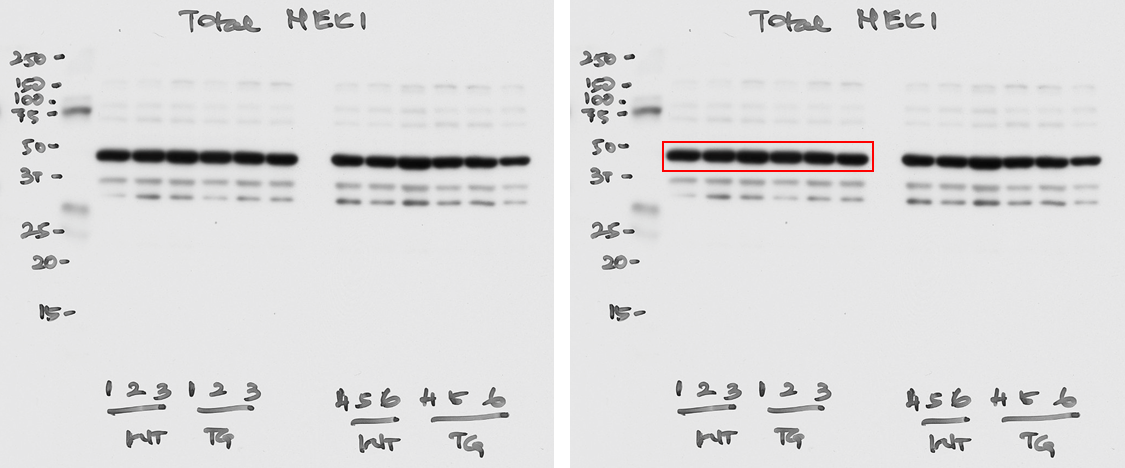

Supplement: Figure 5—source data 1. [file elife-80949-fig5-data1.zip › Figure 5 Source data/Figure 5B_Total_MEK1_2.tif]

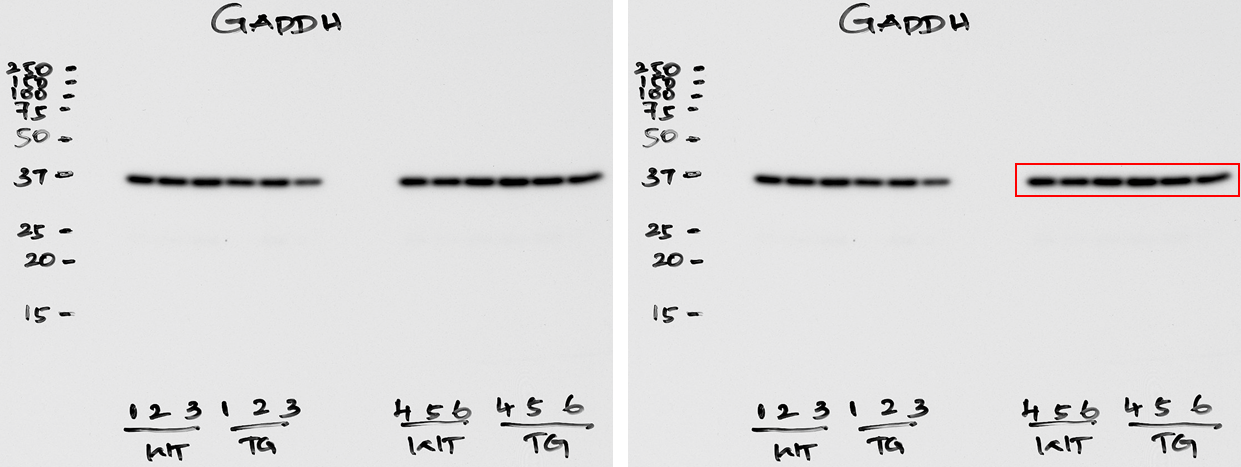

Supplement: Figure 5—source data 1. [file elife-80949-fig5-data1.zip › Figure 5 Source data/Figure 5C_GAPDH.tif]

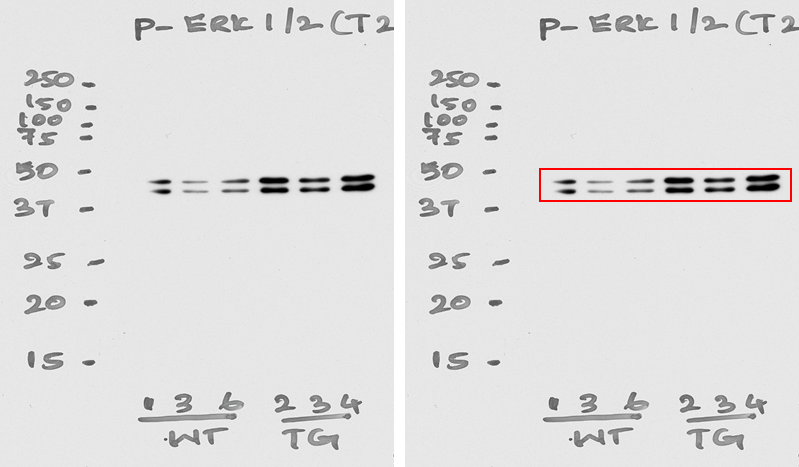

Supplement: Figure 5—source data 1. [file elife-80949-fig5-data1.zip › Figure 5 Source data/Figure 5C_p_ERK1_2_Thr202_Tyr204.tif]

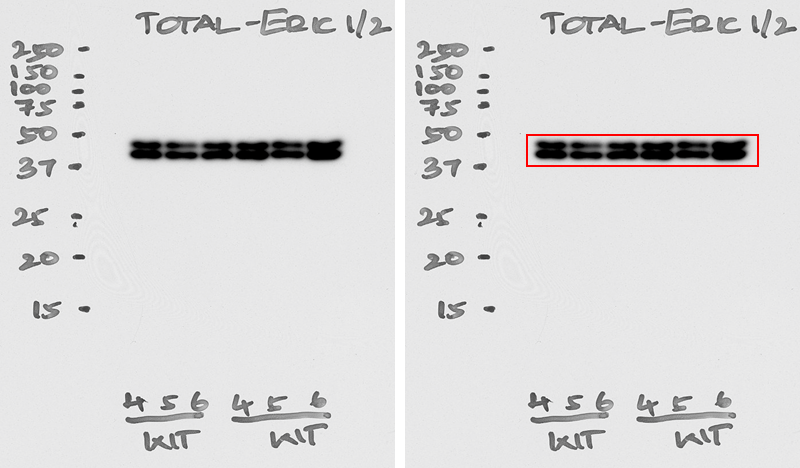

Supplement: Figure 5—source data 1. [file elife-80949-fig5-data1.zip › Figure 5 Source data/Figure 5C_Total ERK1_2.tif]

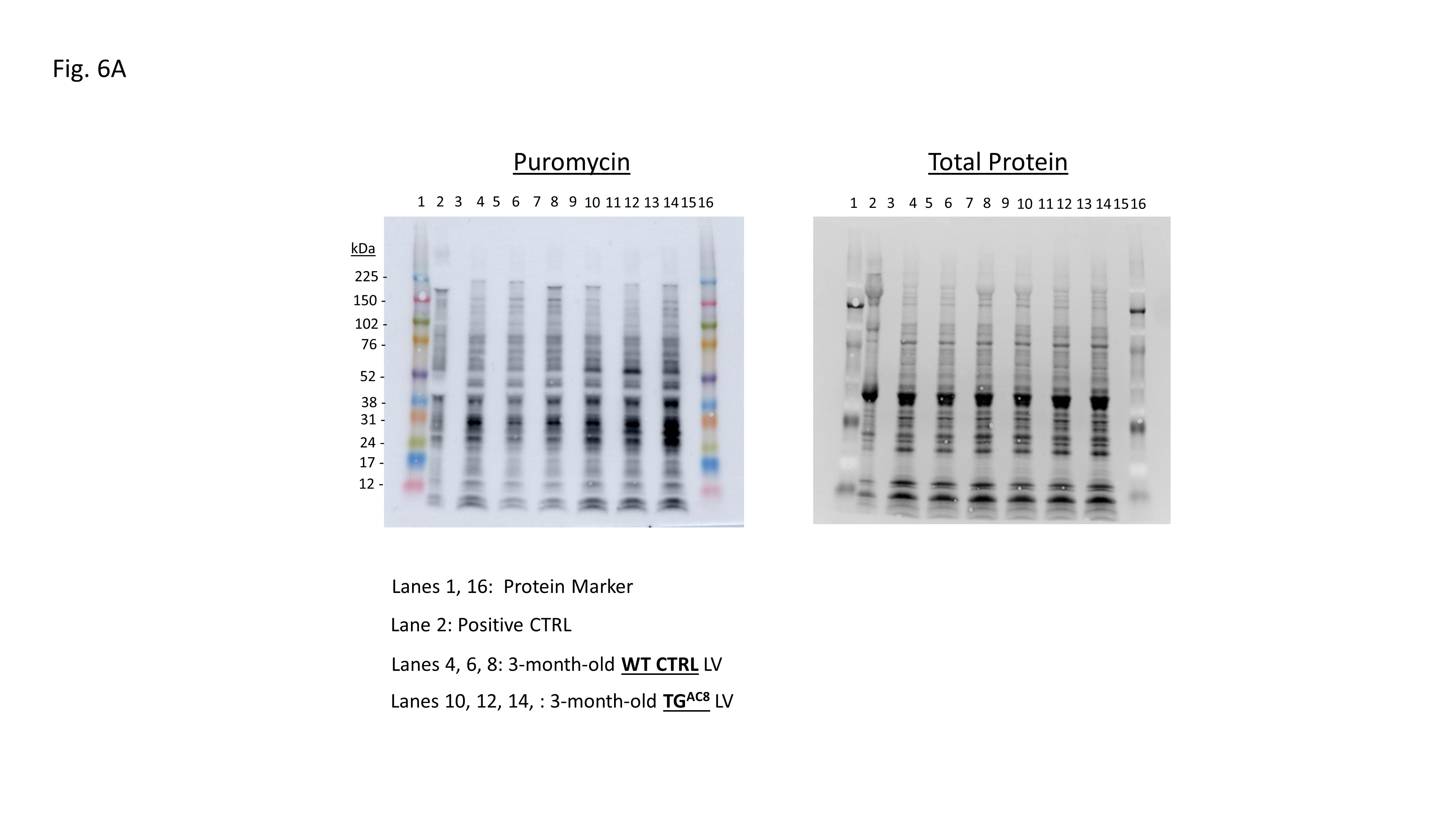

Supplement: Figure 5—source data 1. [file elife-80949-fig5-data1.zip › Figure 5 Source data/Uncropped images/5a.JPG]

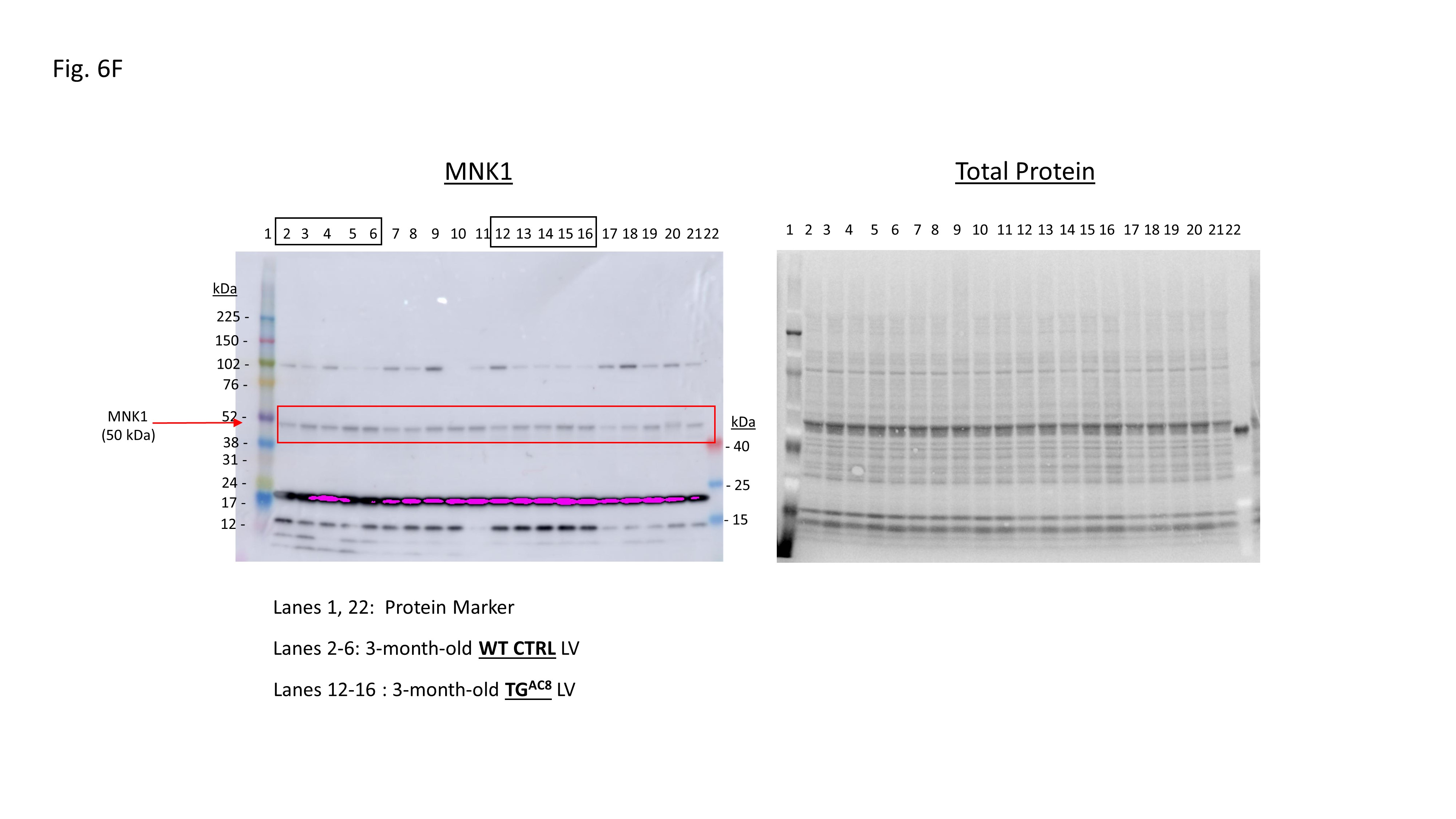

Supplement: Figure 5—source data 1. [file elife-80949-fig5-data1.zip › Figure 5 Source data/Uncropped images/5f.JPG]

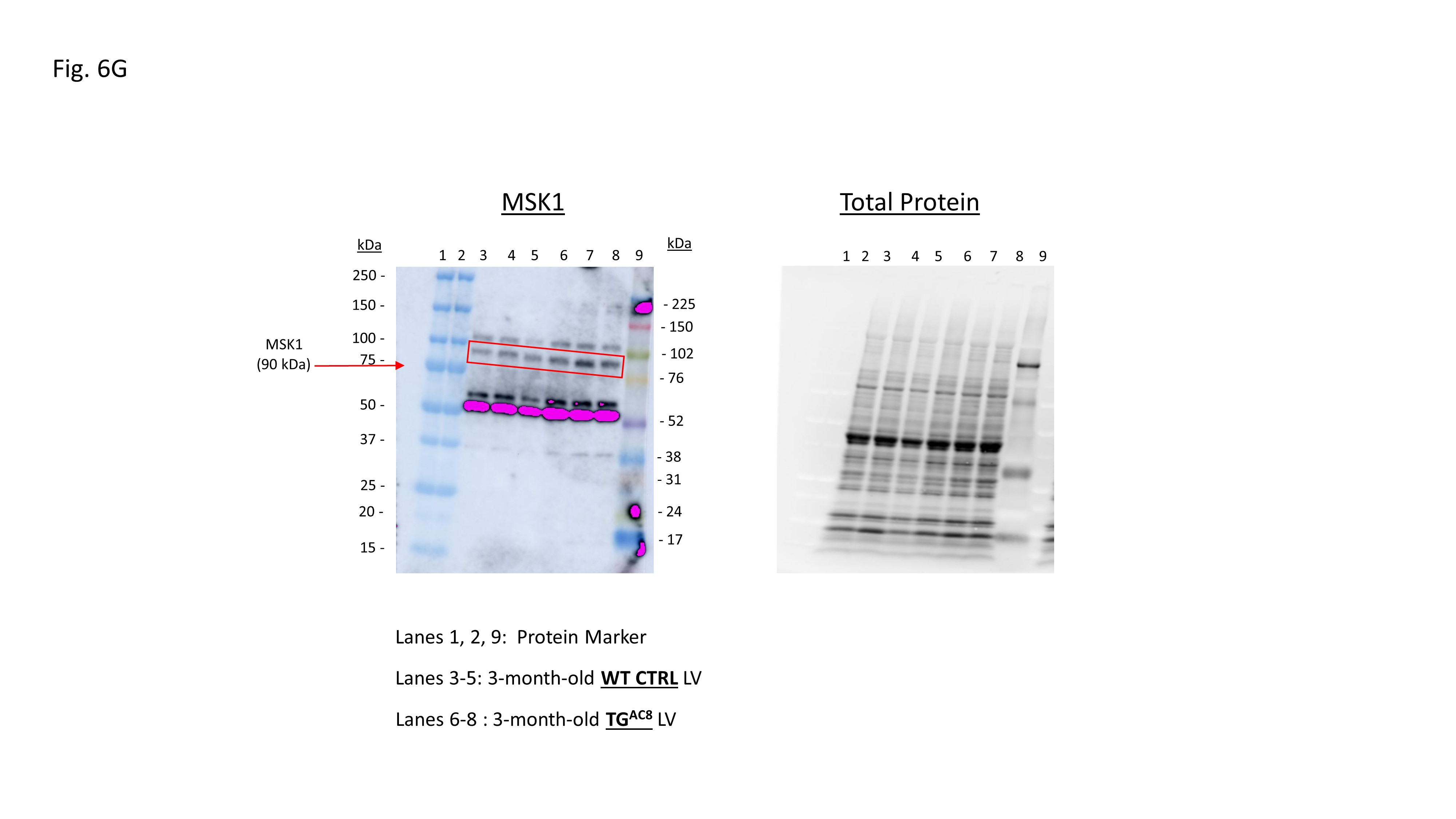

Supplement: Figure 5—source data 1. [file elife-80949-fig5-data1.zip › Figure 5 Source data/Uncropped images/5g.JPG]

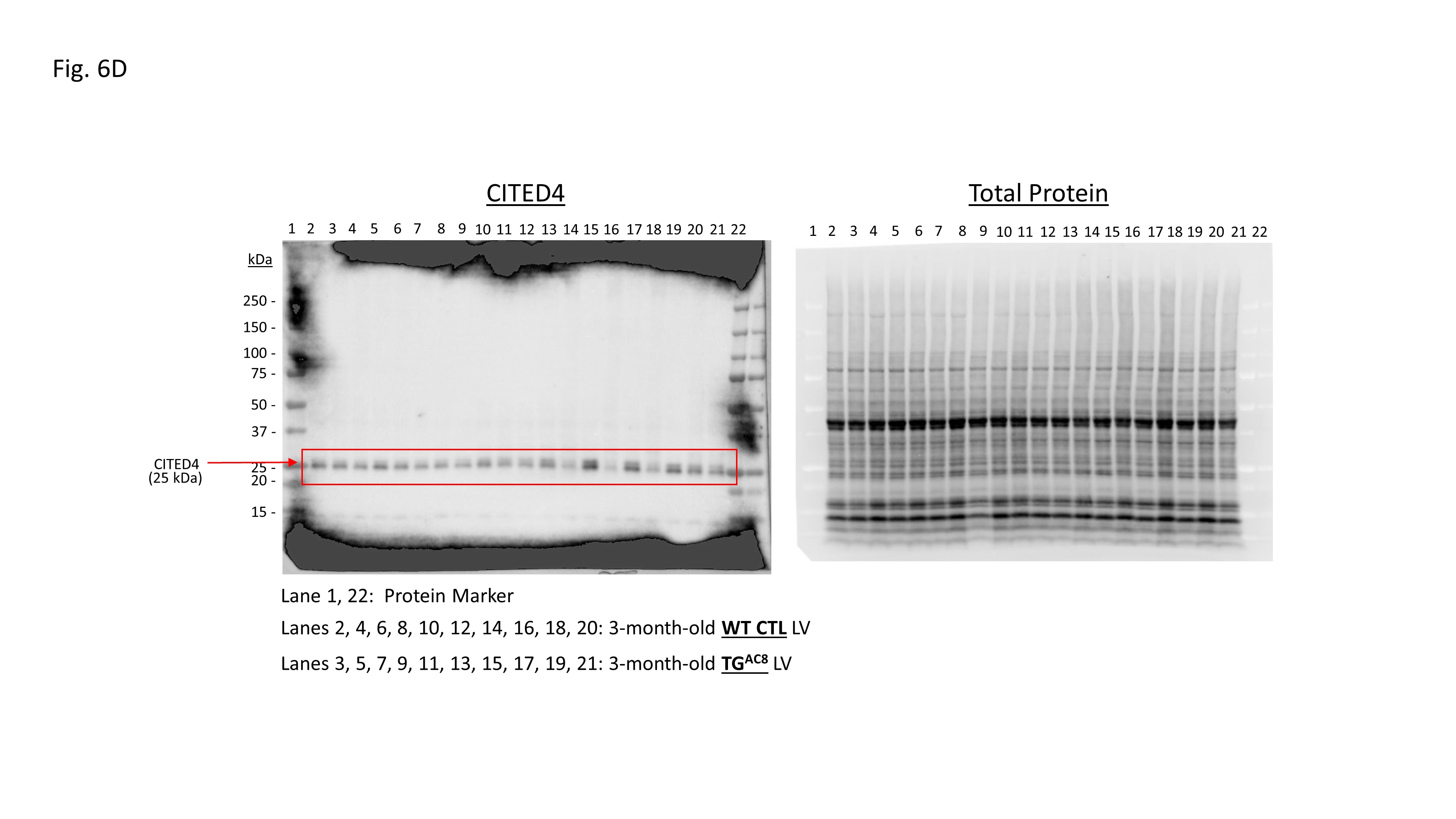

Supplement: Figure 5—source data 1. [file elife-80949-fig5-data1.zip › Figure 5 Source data/Uncropped images/CITED4.JPG]

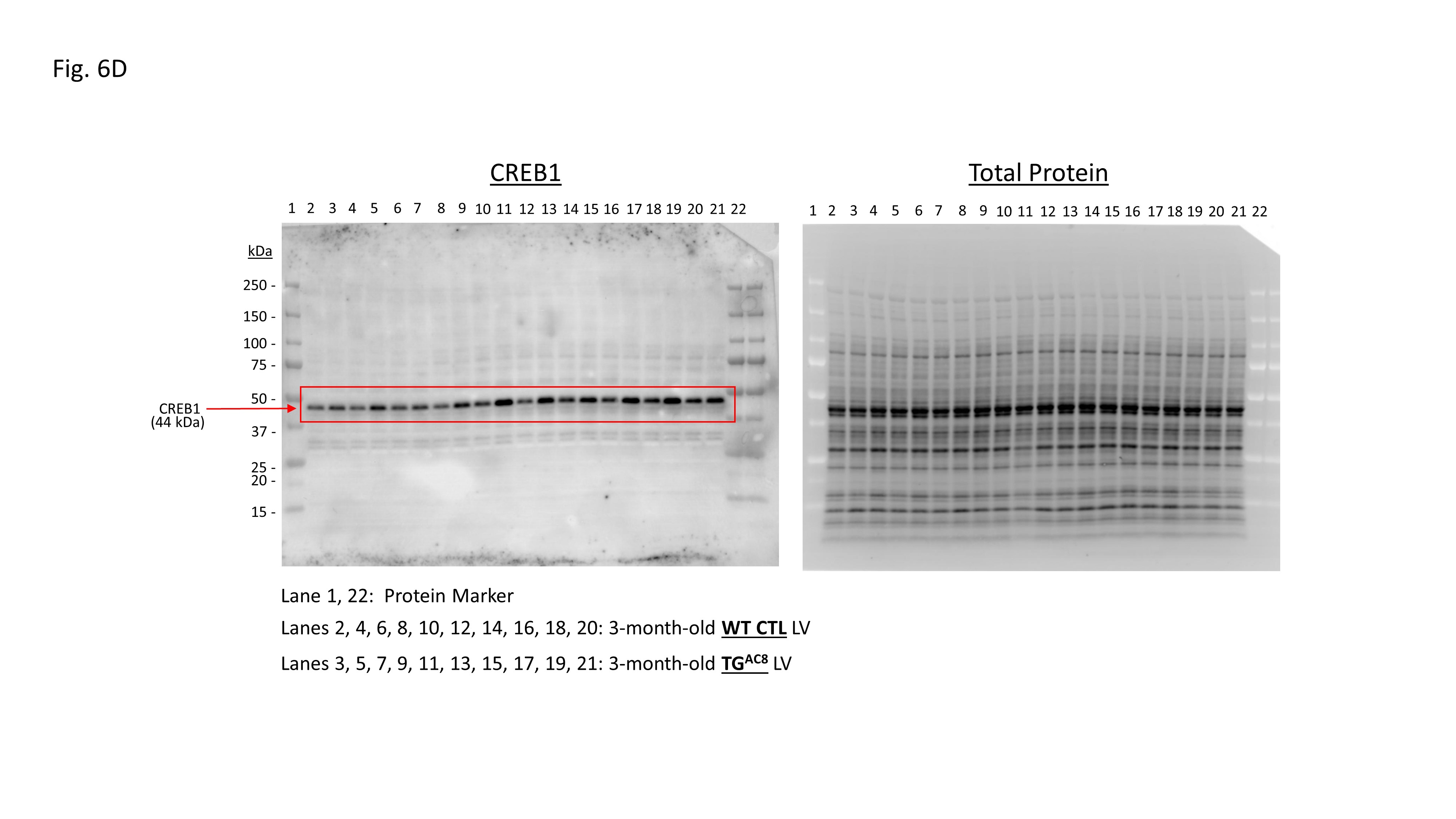

Supplement: Figure 5—source data 1. [file elife-80949-fig5-data1.zip › Figure 5 Source data/Uncropped images/CREB1.JPG]

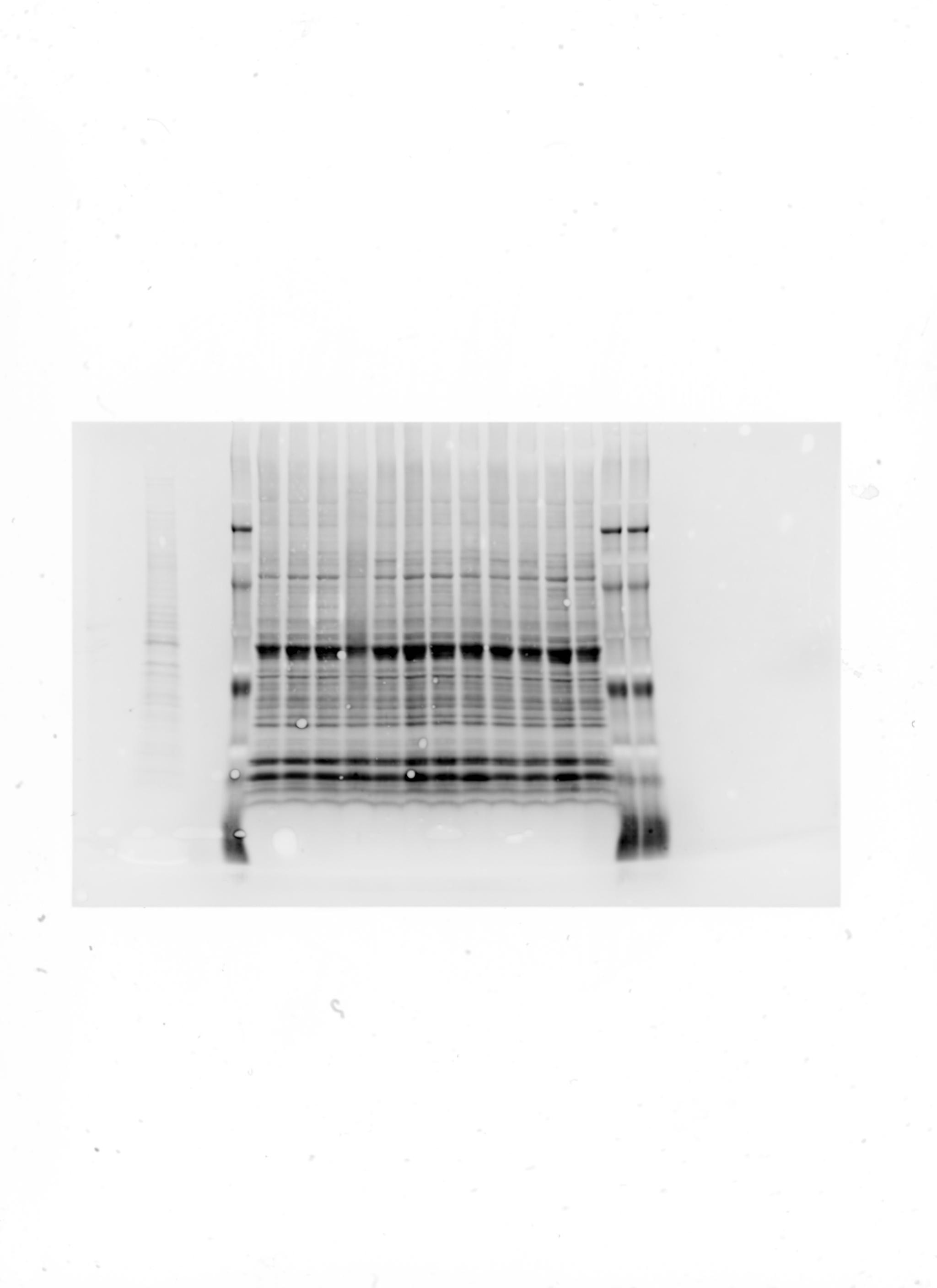

Supplement: Figure 6—source data 1. [file elife-80949-fig6-data1.zip › Figure 6 source data/Fig6D - HSP90/CRC_HSP90 Total Protein/CRC_HSP90 Total Protein.jpg]

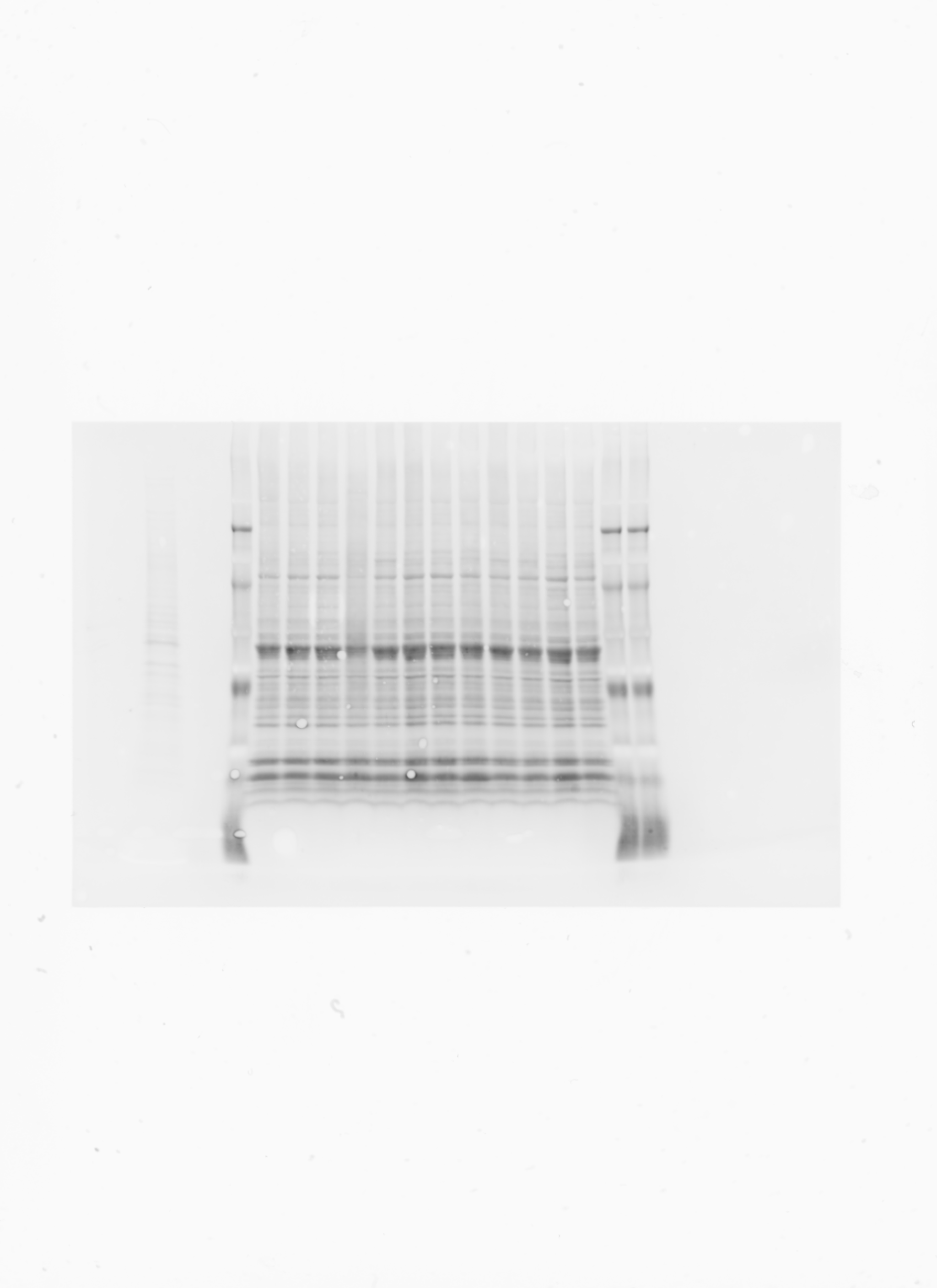

Supplement: Figure 6—source data 1. [file elife-80949-fig6-data1.zip › Figure 6 source data/Fig6D - HSP90/CRC_HSP90 Total Protein/CRC_HSP90 Total Protein.tif]

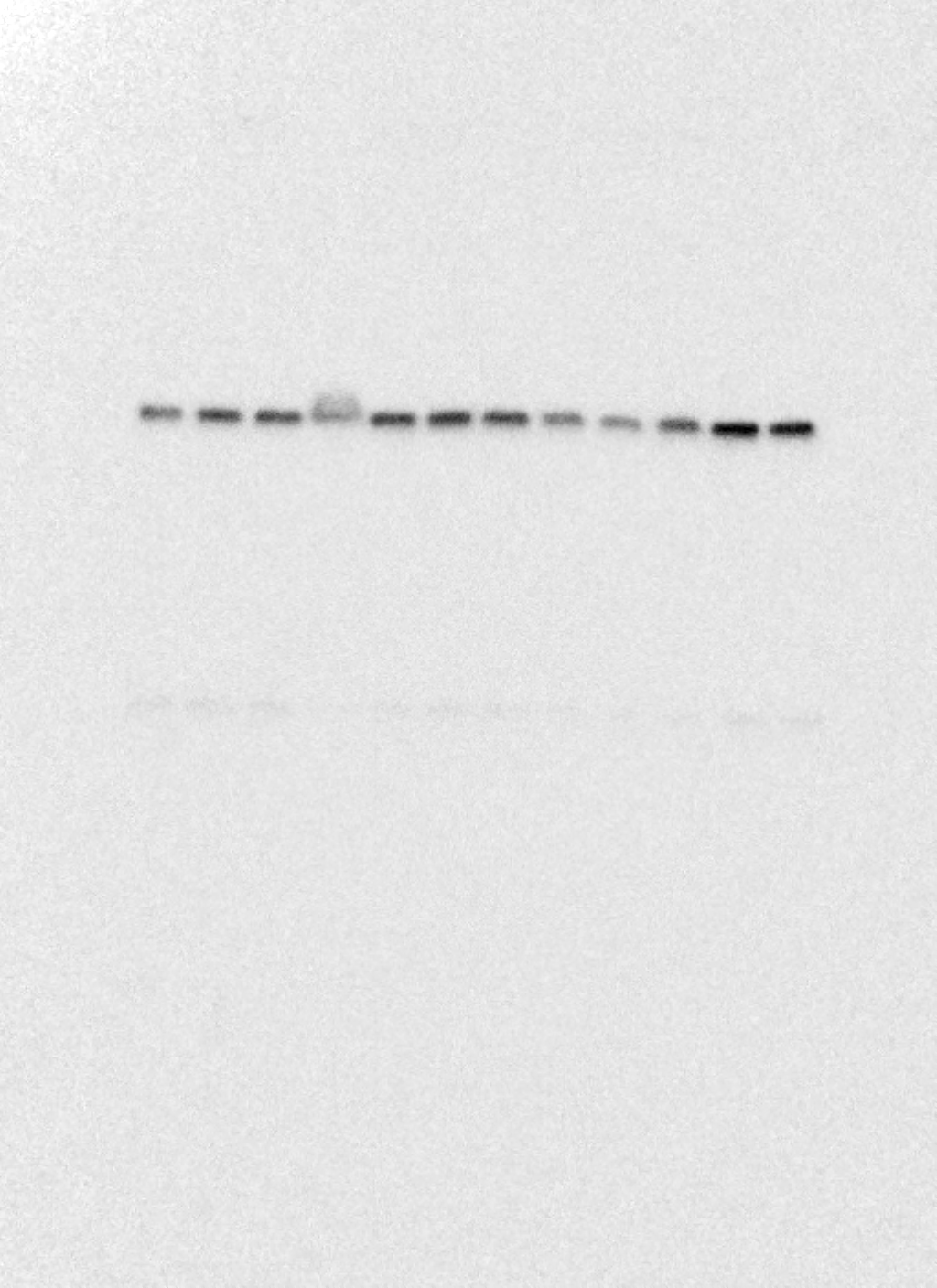

Supplement: Figure 6—source data 1. [file elife-80949-fig6-data1.zip › Figure 6 source data/Fig6D - HSP90/CRC_HSP90/CRC_HSP90a_Ch.tif]

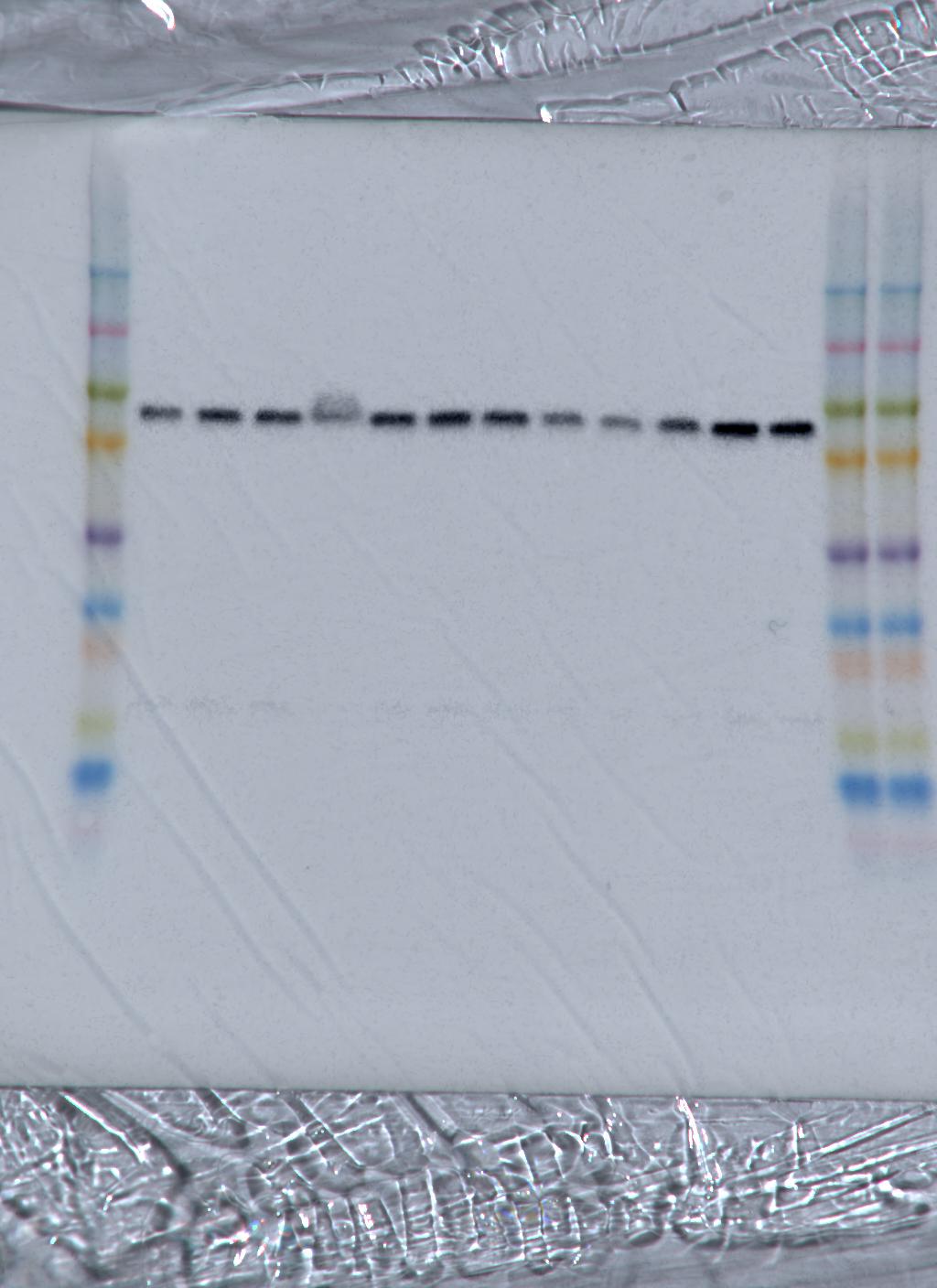

Supplement: Figure 6—source data 1. [file elife-80949-fig6-data1.zip › Figure 6 source data/Fig6D - HSP90/CRC_HSP90/CRC_HSP90a_Ch+Marker.jpg]

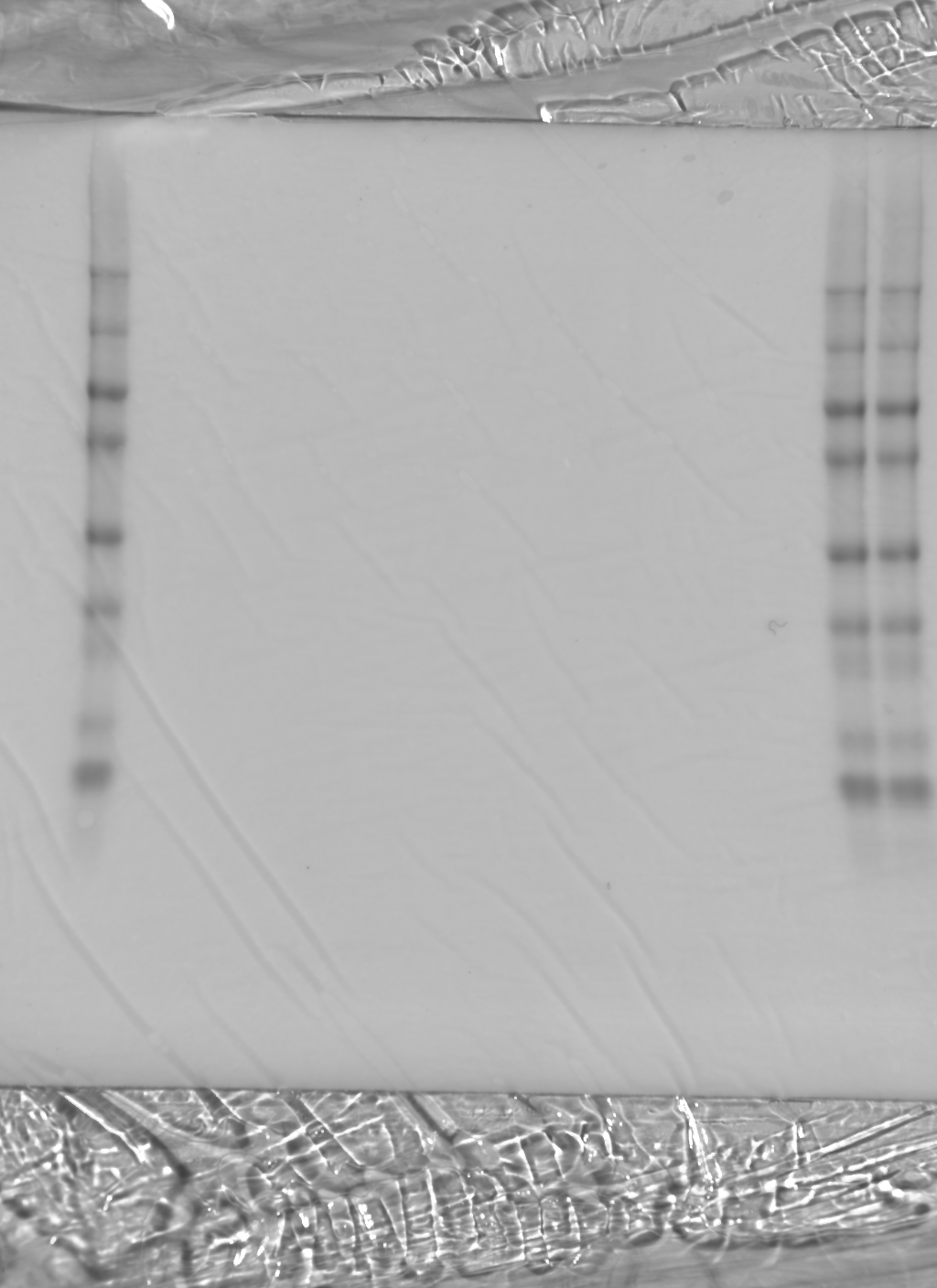

Supplement: Figure 6—source data 1. [file elife-80949-fig6-data1.zip › Figure 6 source data/Fig6D - HSP90/CRC_HSP90/CRC_HSP90a_Marker.tif]

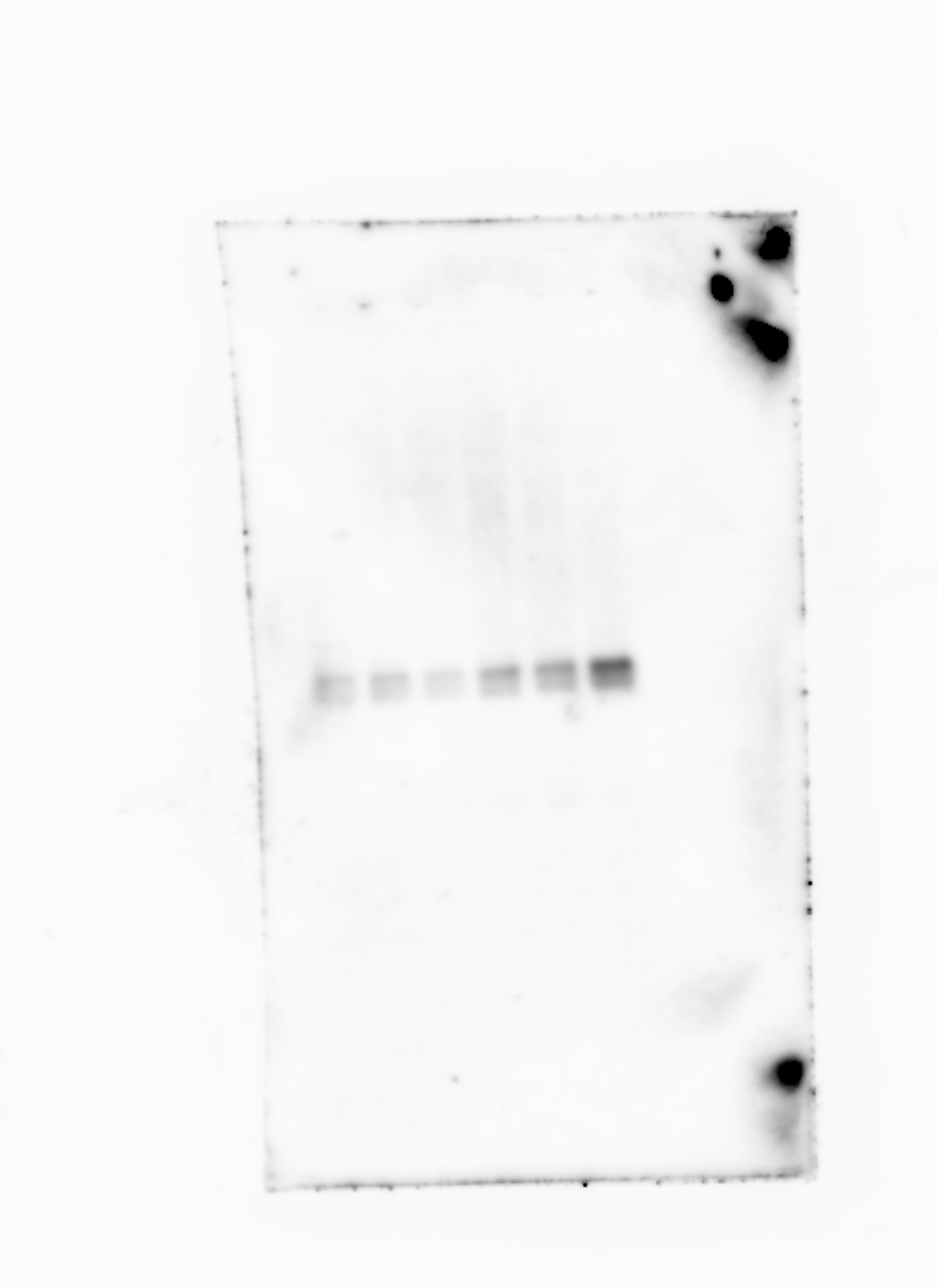

Supplement: Figure 6—source data 1. [file elife-80949-fig6-data1.zip › Figure 6 source data/Fig6E - ATG13/MGP_ATG13/MGP_ATG13_Ch.tif]

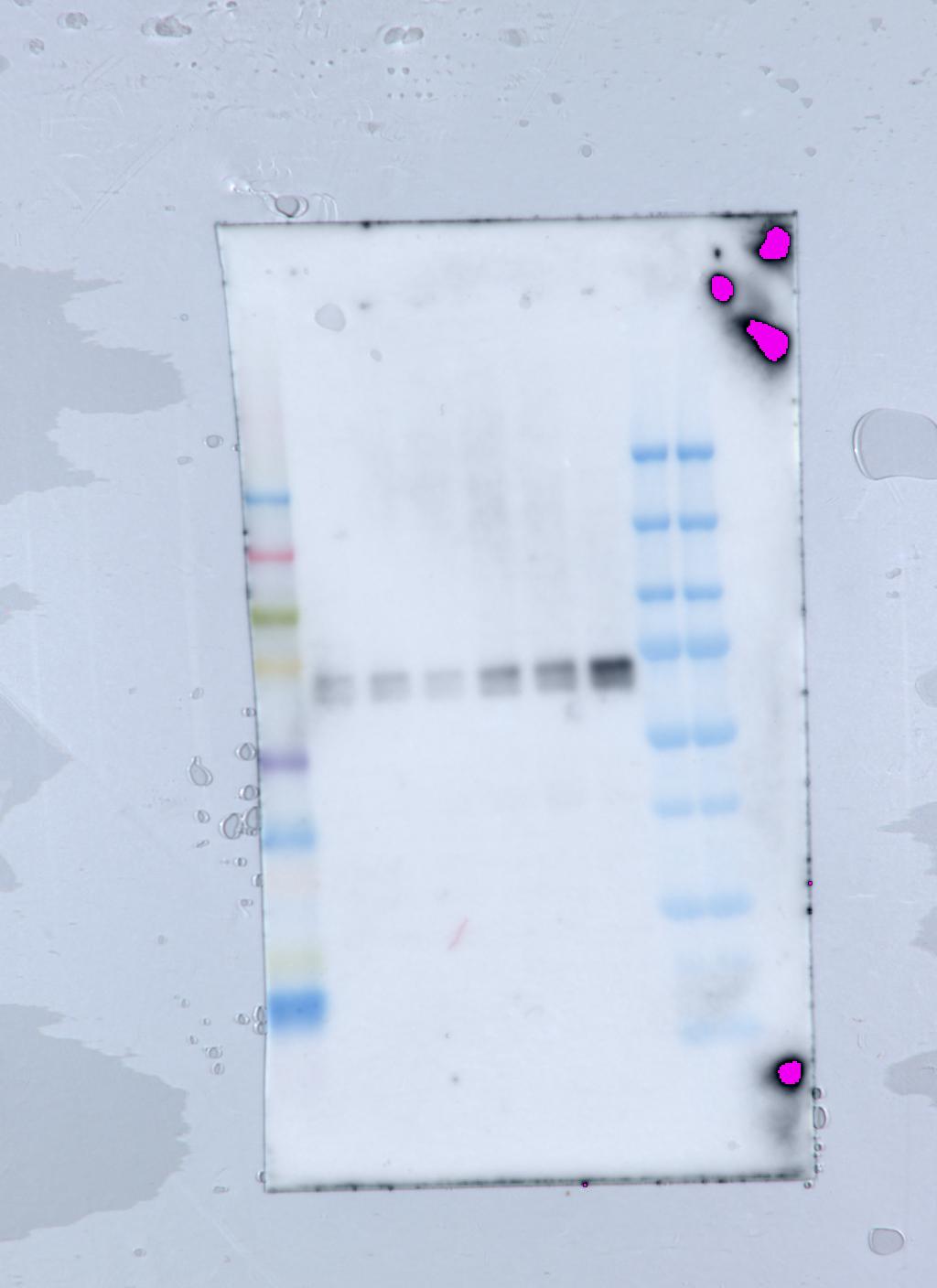

Supplement: Figure 6—source data 1. [file elife-80949-fig6-data1.zip › Figure 6 source data/Fig6E - ATG13/MGP_ATG13/MGP_ATG13_Ch+Marker.jpg]

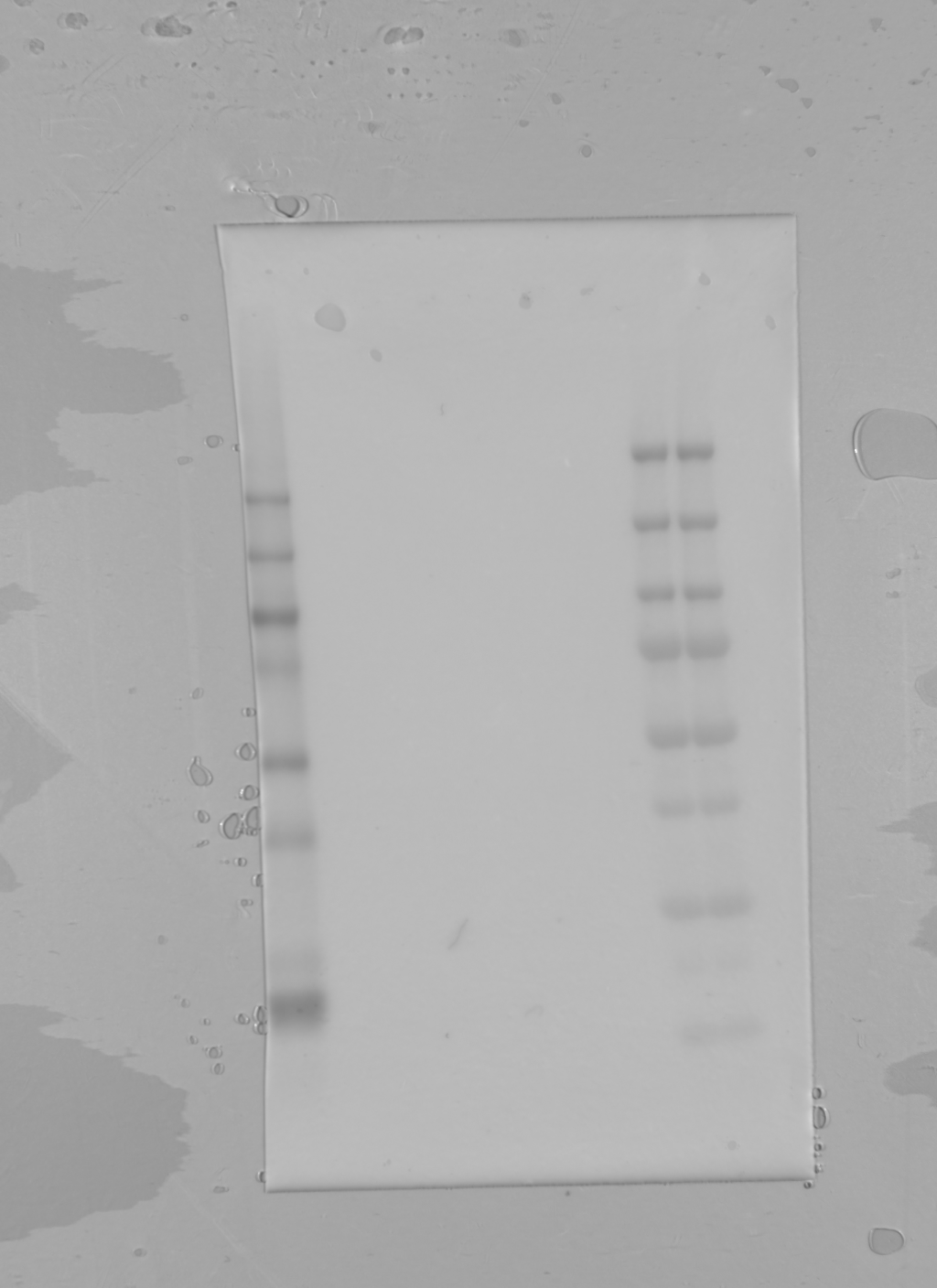

Supplement: Figure 6—source data 1. [file elife-80949-fig6-data1.zip › Figure 6 source data/Fig6E - ATG13/MGP_ATG13/MGP_ATG13_Marker.tif]

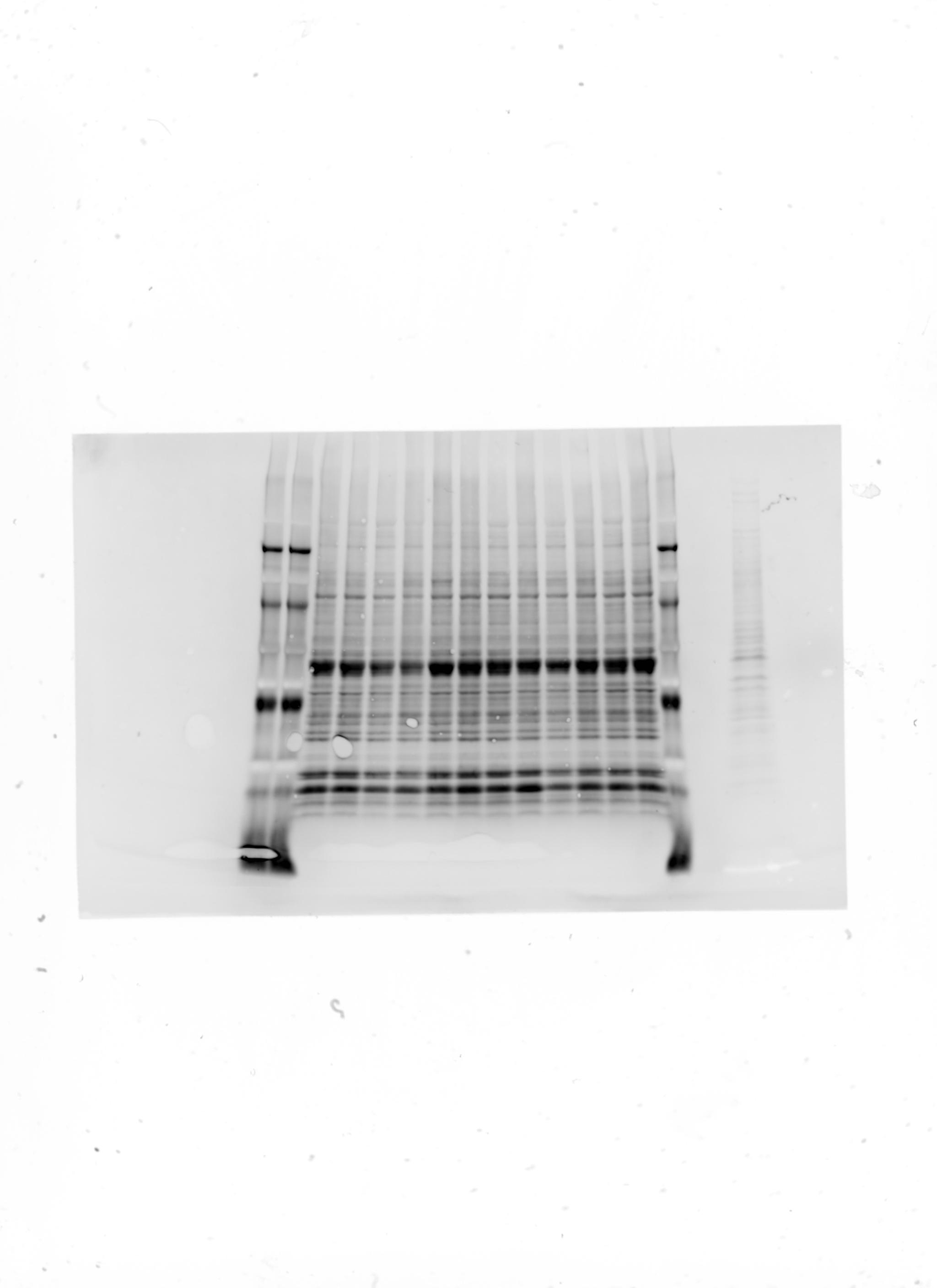

Supplement: Figure 6—source data 1. [file elife-80949-fig6-data1.zip › Figure 6 source data/Fig6F - PARKIN/CRC_PARKIN Total Protein/CRC_PARKIN Total Protein.jpg]

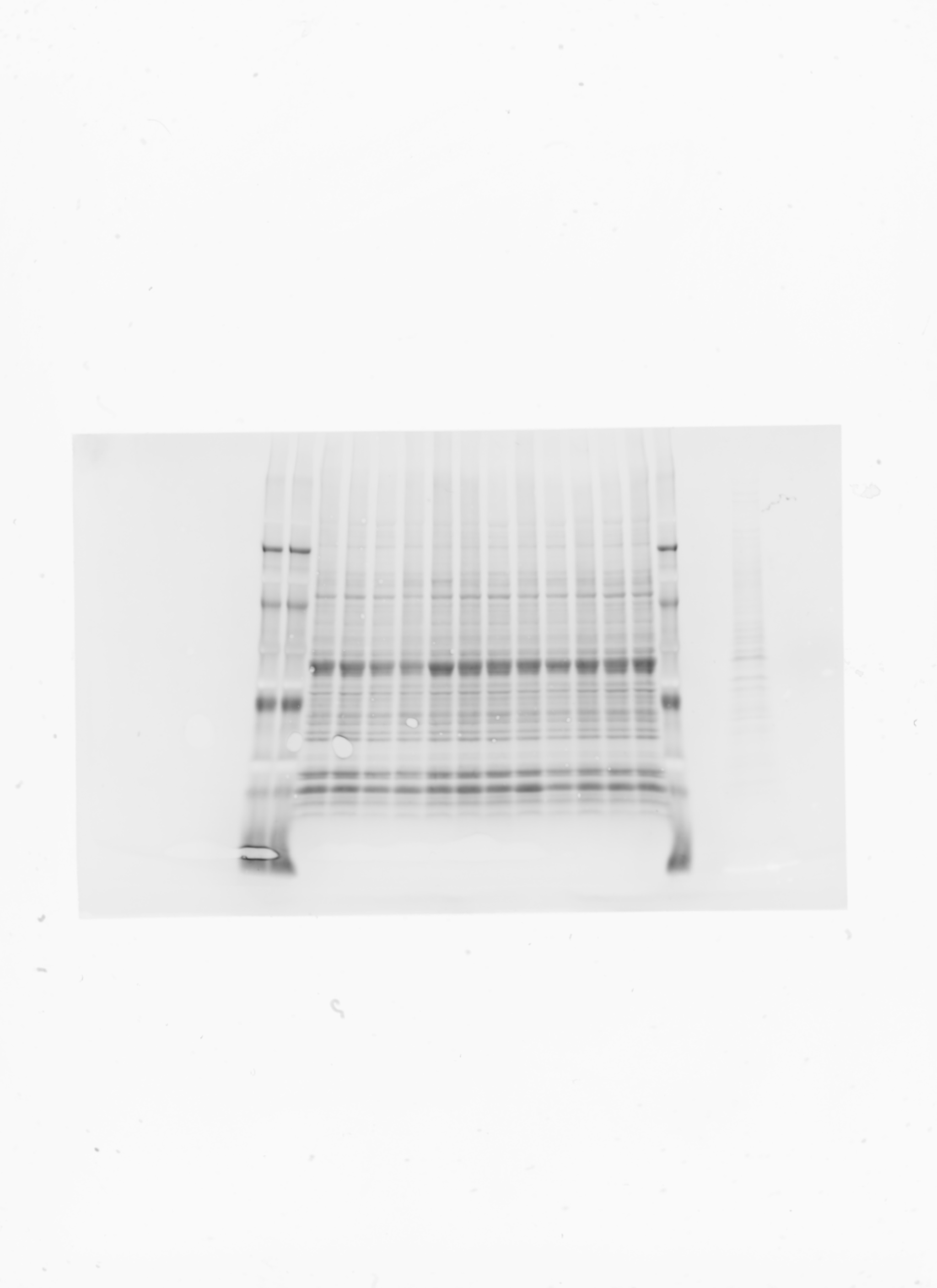

Supplement: Figure 6—source data 1. [file elife-80949-fig6-data1.zip › Figure 6 source data/Fig6F - PARKIN/CRC_PARKIN Total Protein/CRC_PARKIN Total Protein.tif]

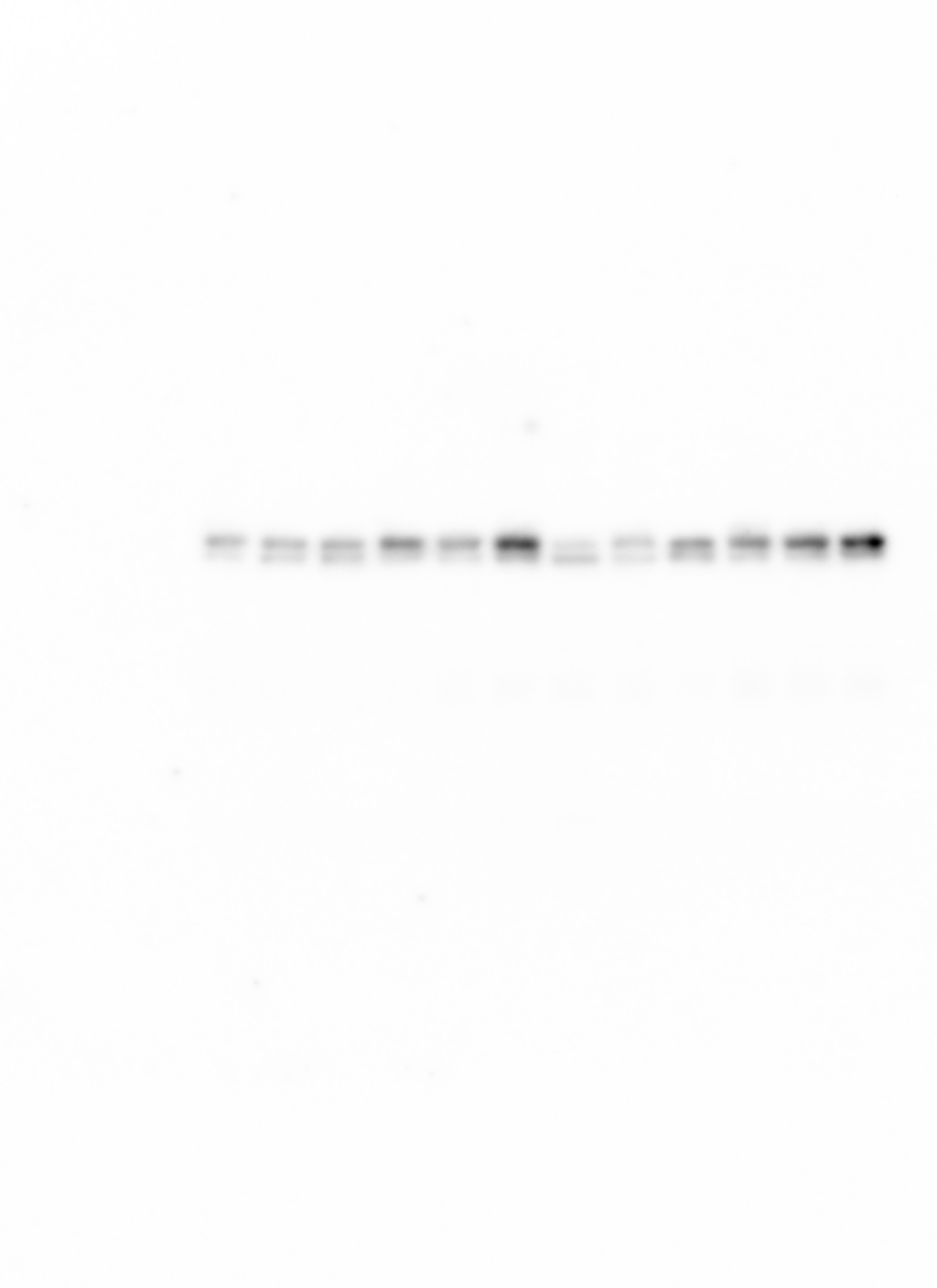

Supplement: Figure 6—source data 1. [file elife-80949-fig6-data1.zip › Figure 6 source data/Fig6F - PARKIN/CRC_PARKIN/CRC_PARKIN_Ch.tif]

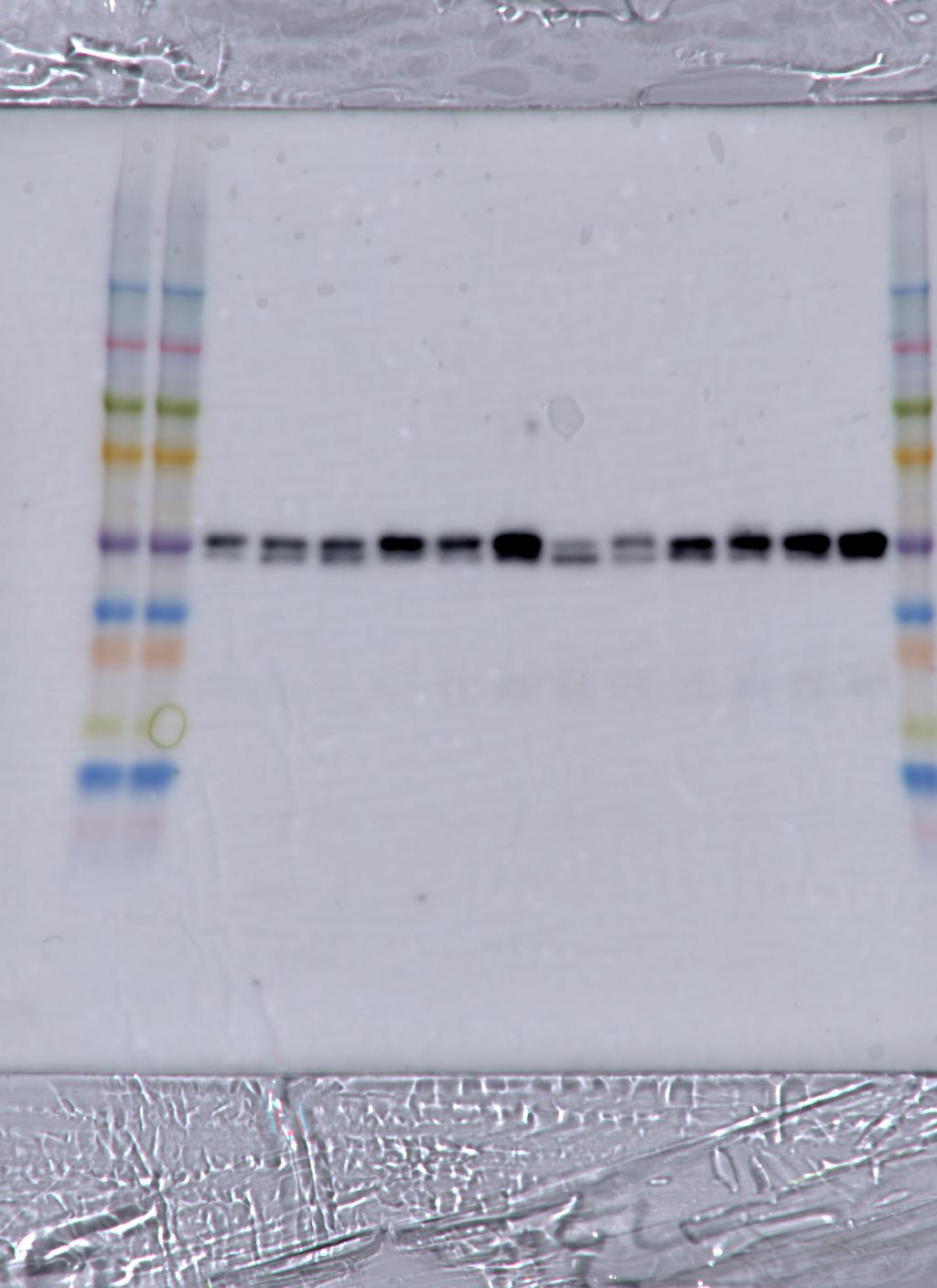

Supplement: Figure 6—source data 1. [file elife-80949-fig6-data1.zip › Figure 6 source data/Fig6F - PARKIN/CRC_PARKIN/CRC_PARKIN_Ch+Marker.jpg]

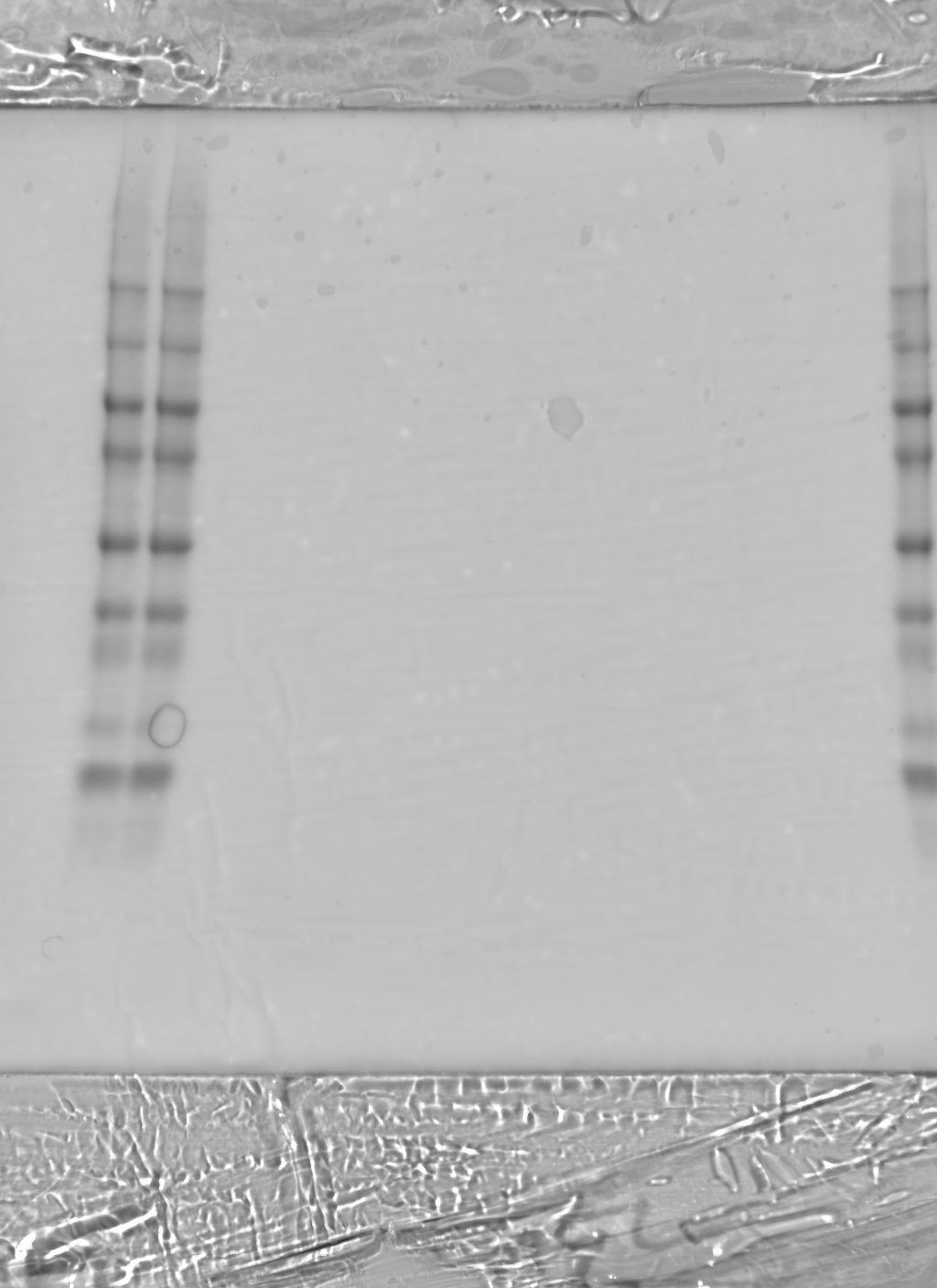

Supplement: Figure 6—source data 1. [file elife-80949-fig6-data1.zip › Figure 6 source data/Fig6F - PARKIN/CRC_PARKIN/CRC_PARKIN_Marker.tif]

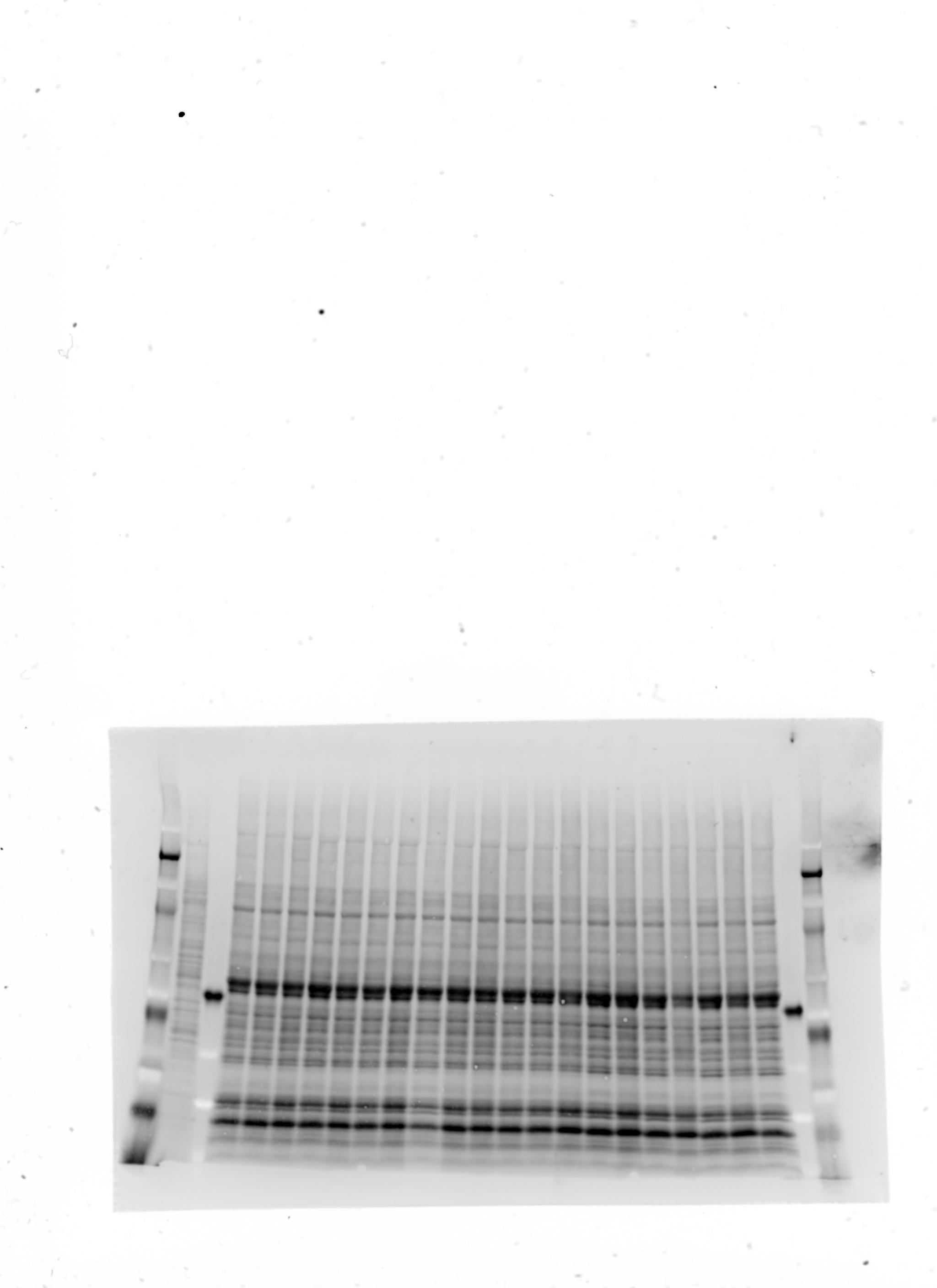

Supplement: Figure 6—source data 1. [file elife-80949-fig6-data1.zip › Figure 6 source data/Fig6G - ATG4B/MGP_ATG4B Total Protein/MGP_ATG4B Total Protein.jpg]

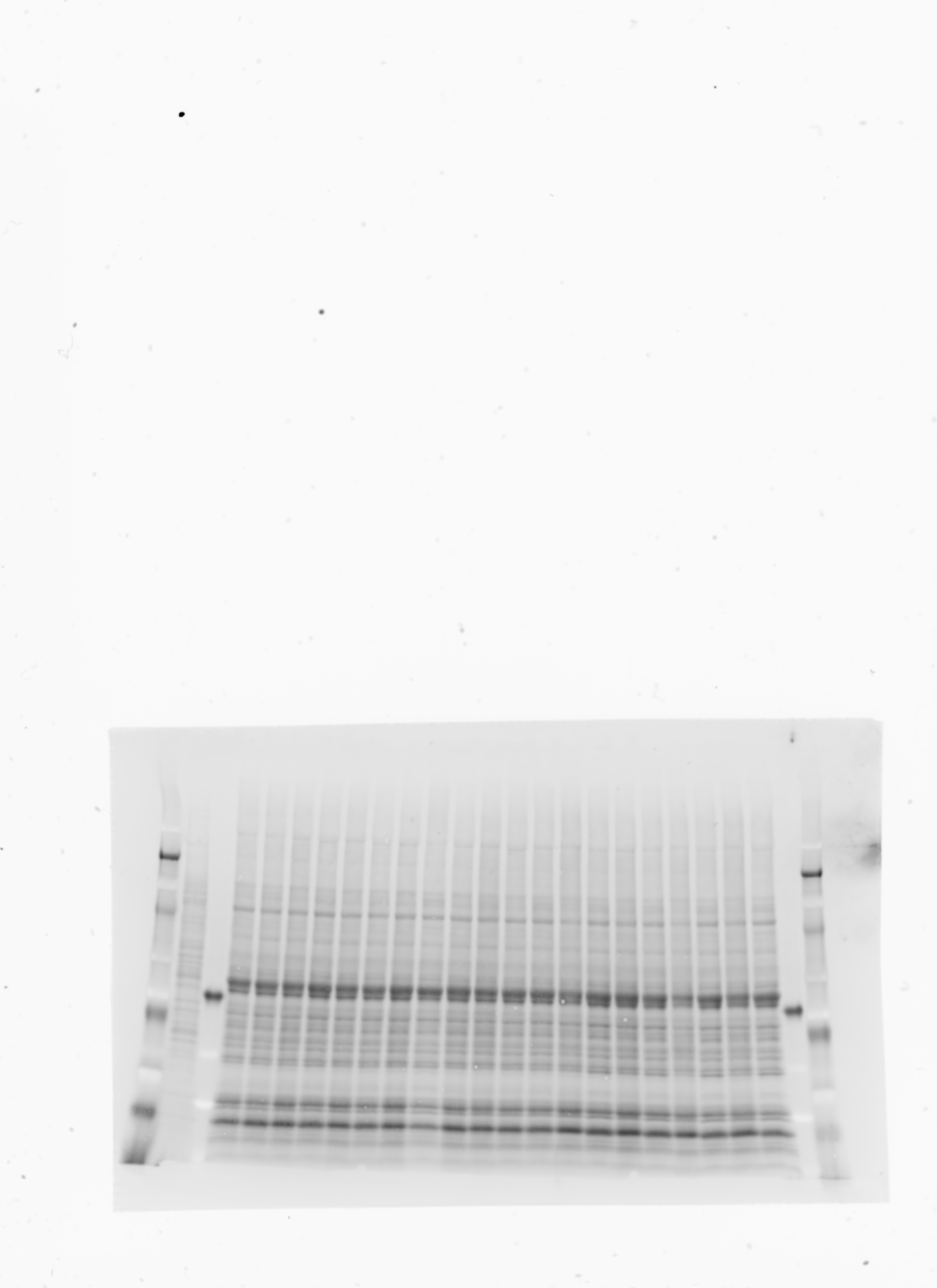

Supplement: Figure 6—source data 1. [file elife-80949-fig6-data1.zip › Figure 6 source data/Fig6G - ATG4B/MGP_ATG4B Total Protein/MGP_ATG4B Total Protein.tif]

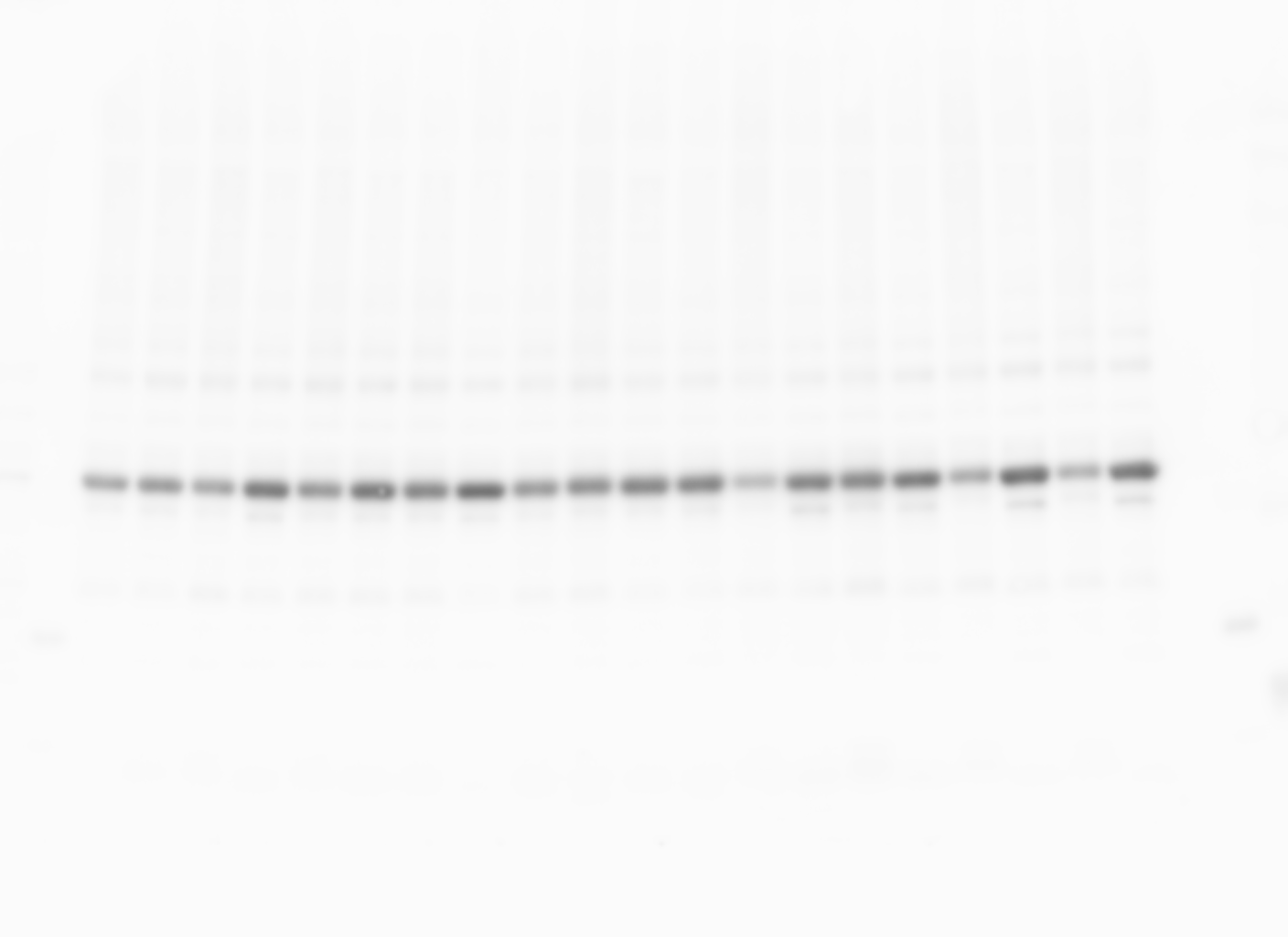

Supplement: Figure 6—source data 1. [file elife-80949-fig6-data1.zip › Figure 6 source data/Fig6G - ATG4B/MGP_ATG4B/MGP_ATG4B_Ch.tif]

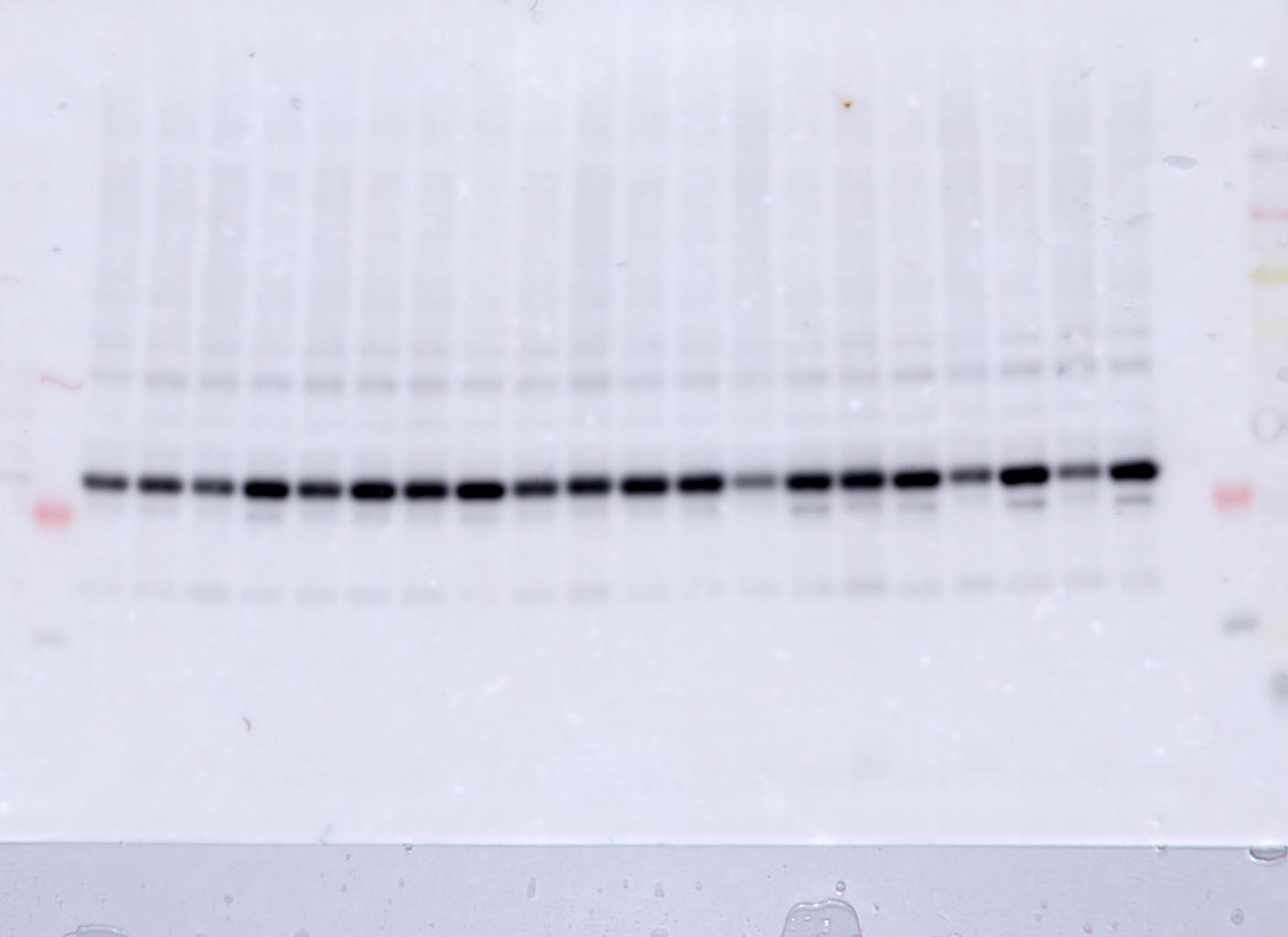

Supplement: Figure 6—source data 1. [file elife-80949-fig6-data1.zip › Figure 6 source data/Fig6G - ATG4B/MGP_ATG4B/MGP_ATG4B_Ch+Marker.jpg]
