# Supplementary figures and images for: A remarkable adaptive paradigm of heart performance and protection emerges in response to marked cardiac-specific overexpression of ADCY8 (part 2 of 3)
Source: eLife. 2022 Dec 14;11:e80949. doi: 10.7554/eLife.80949 (PMC9822292; doi:10.7554/eLife.80949)

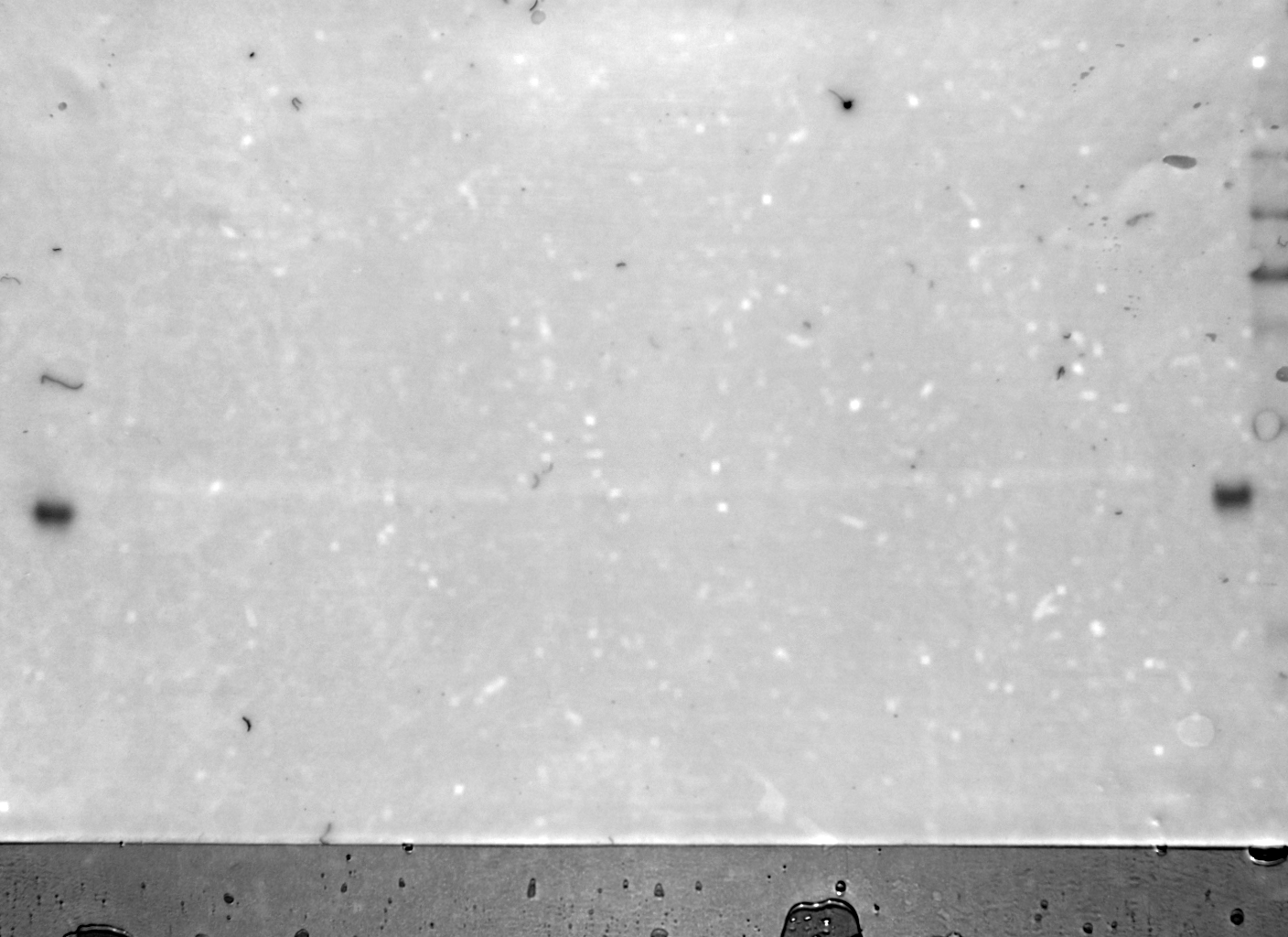

Supplement: Figure 6—source data 1. [file elife-80949-fig6-data1.zip › Figure 6 source data/Fig6G - ATG4B/MGP_ATG4B/MGP_ATG4B_Marker.tif]

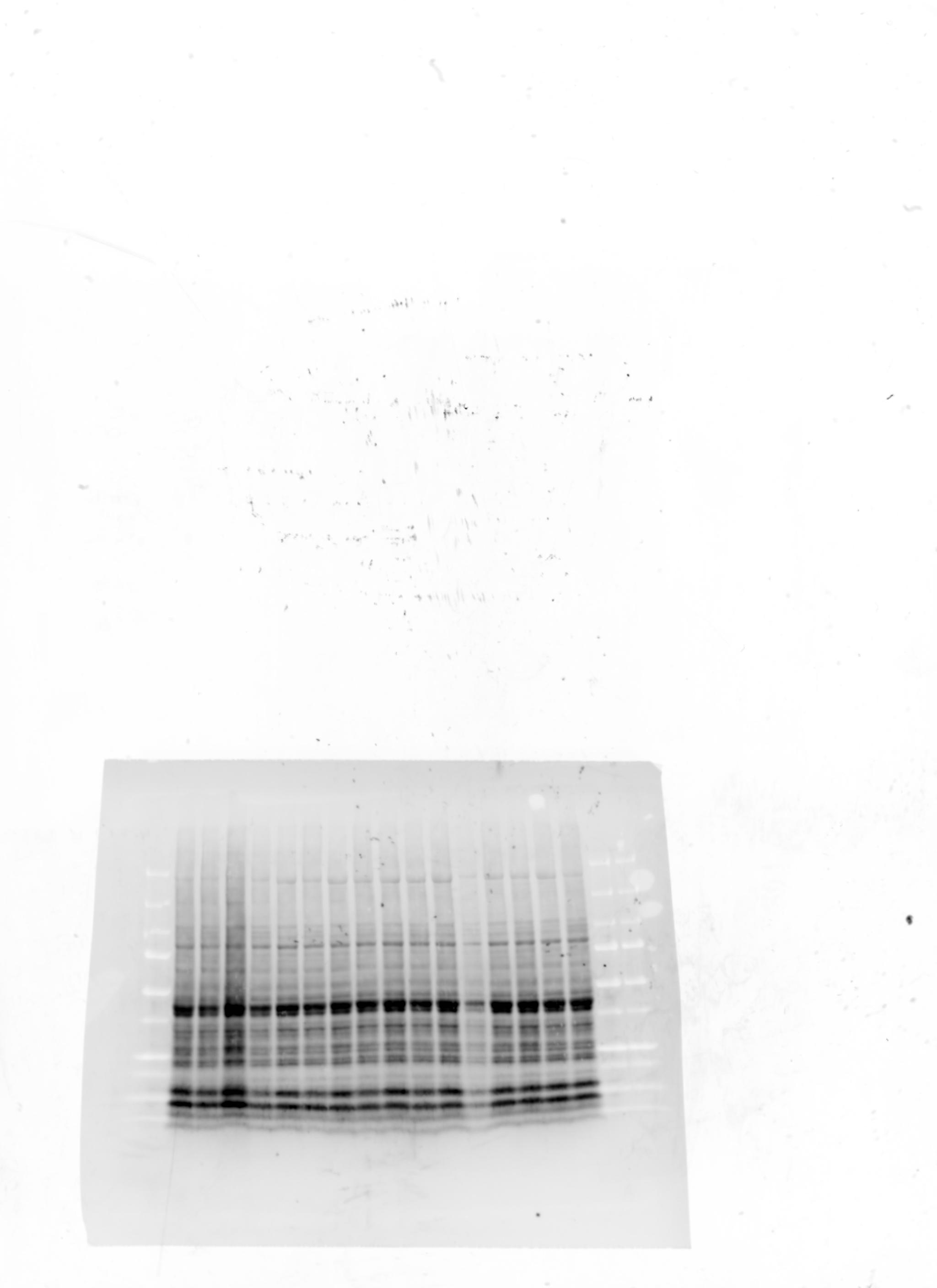

Supplement: Figure 6—source data 1. [file elife-80949-fig6-data1.zip › Figure 6 source data/Fig6H - PI3Kc3/MGP_PI3Kc3 Total Protein/MGP_PI3Kc3 Total Protein.jpg]

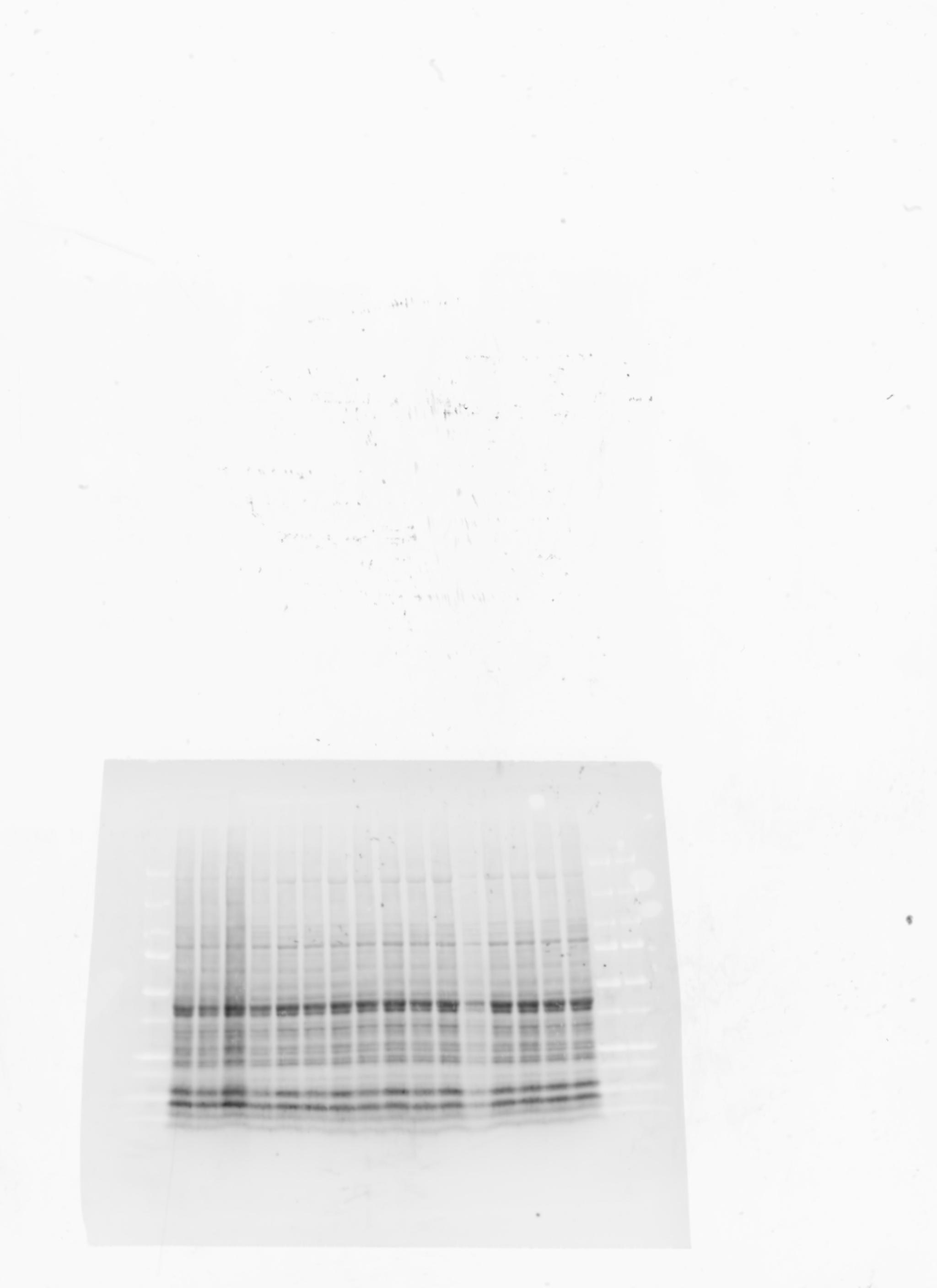

Supplement: Figure 6—source data 1. [file elife-80949-fig6-data1.zip › Figure 6 source data/Fig6H - PI3Kc3/MGP_PI3Kc3 Total Protein/MGP_PI3Kc3 Total Protein.tif]

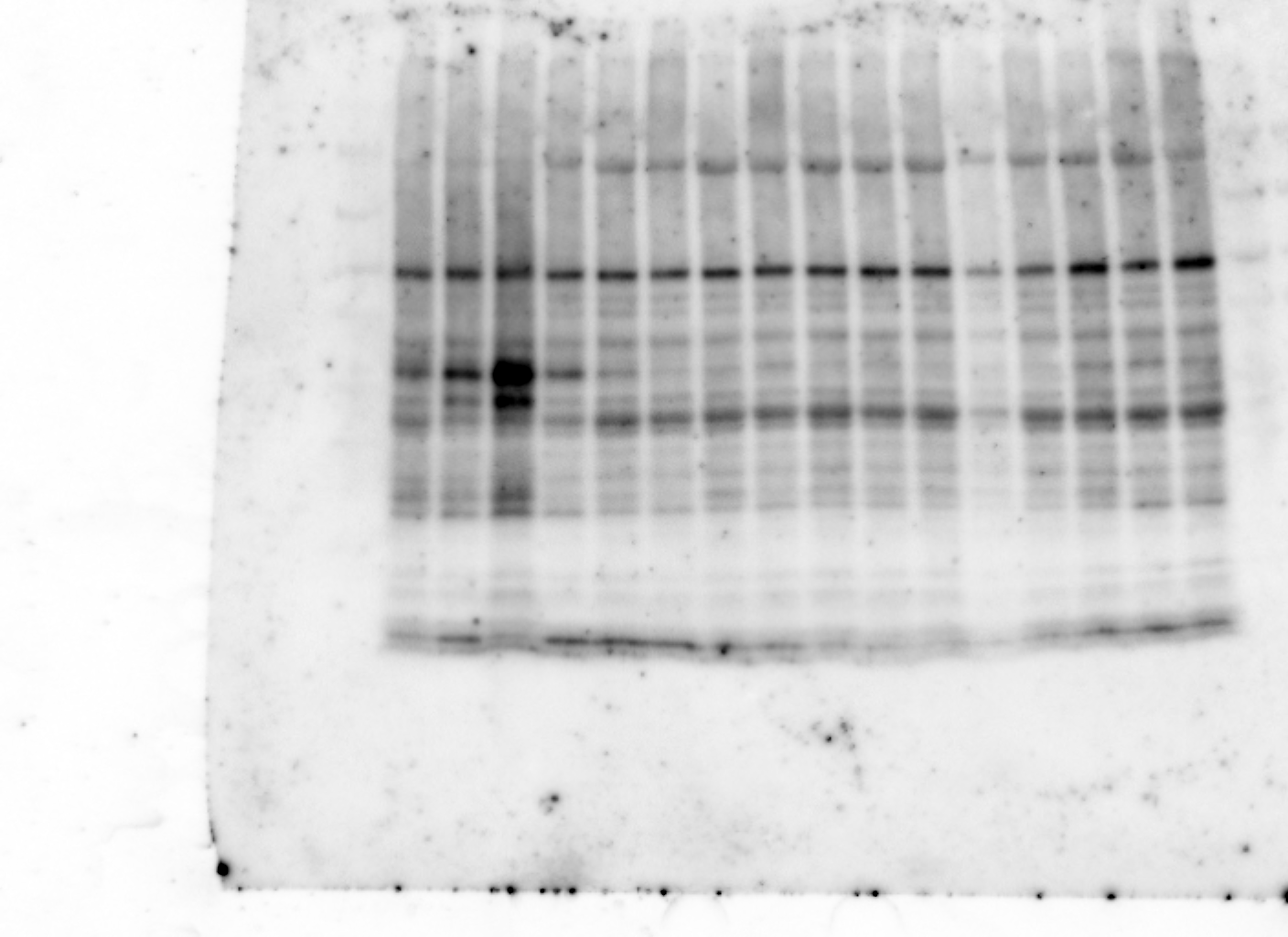

Supplement: Figure 6—source data 1. [file elife-80949-fig6-data1.zip › Figure 6 source data/Fig6H - PI3Kc3/MGP_PI3Kc3/MGP_PI3Kc3_Ch.tif]

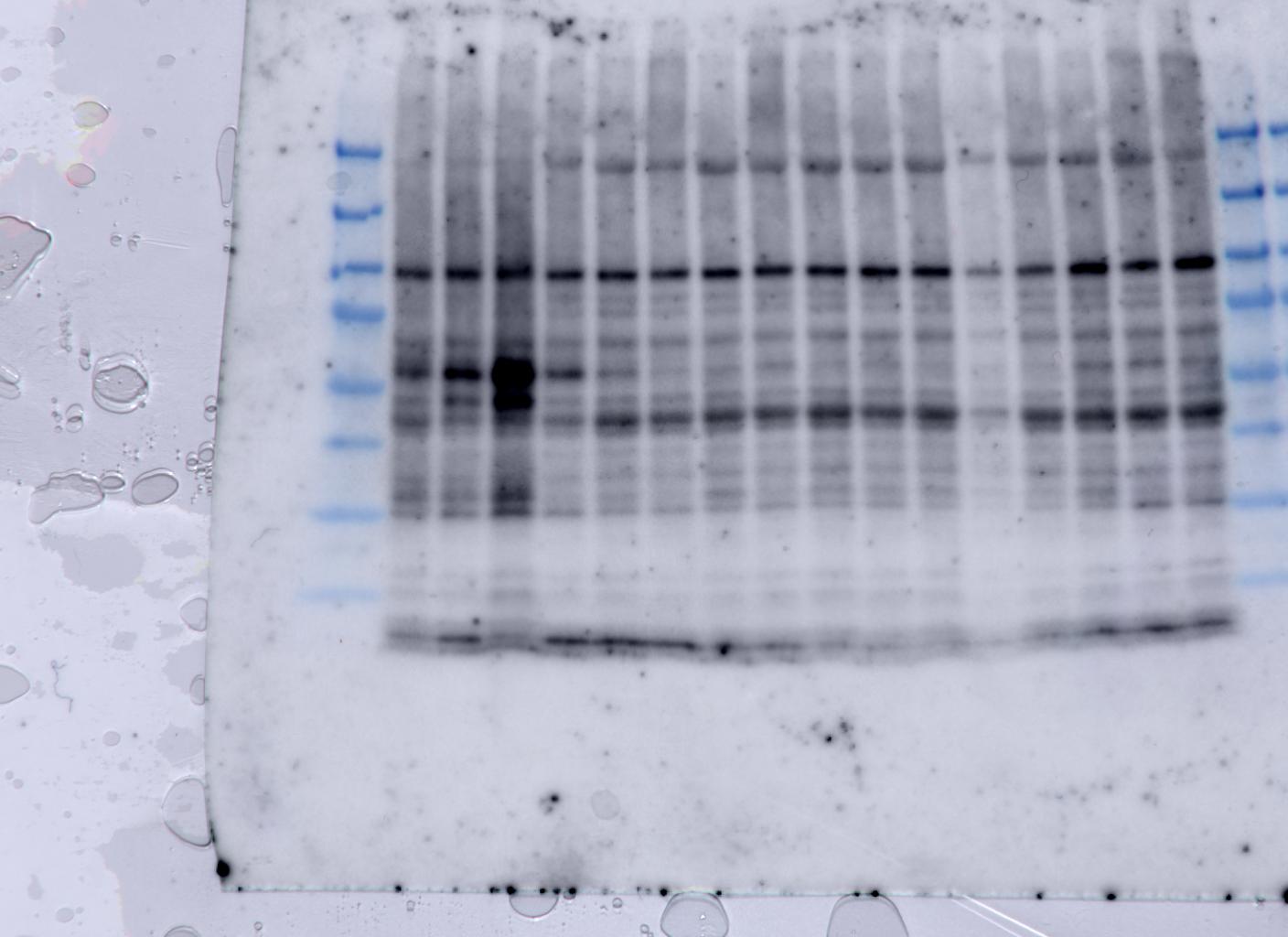

Supplement: Figure 6—source data 1. [file elife-80949-fig6-data1.zip › Figure 6 source data/Fig6H - PI3Kc3/MGP_PI3Kc3/MGP_PI3Kc3_Ch+Marker.jpg]

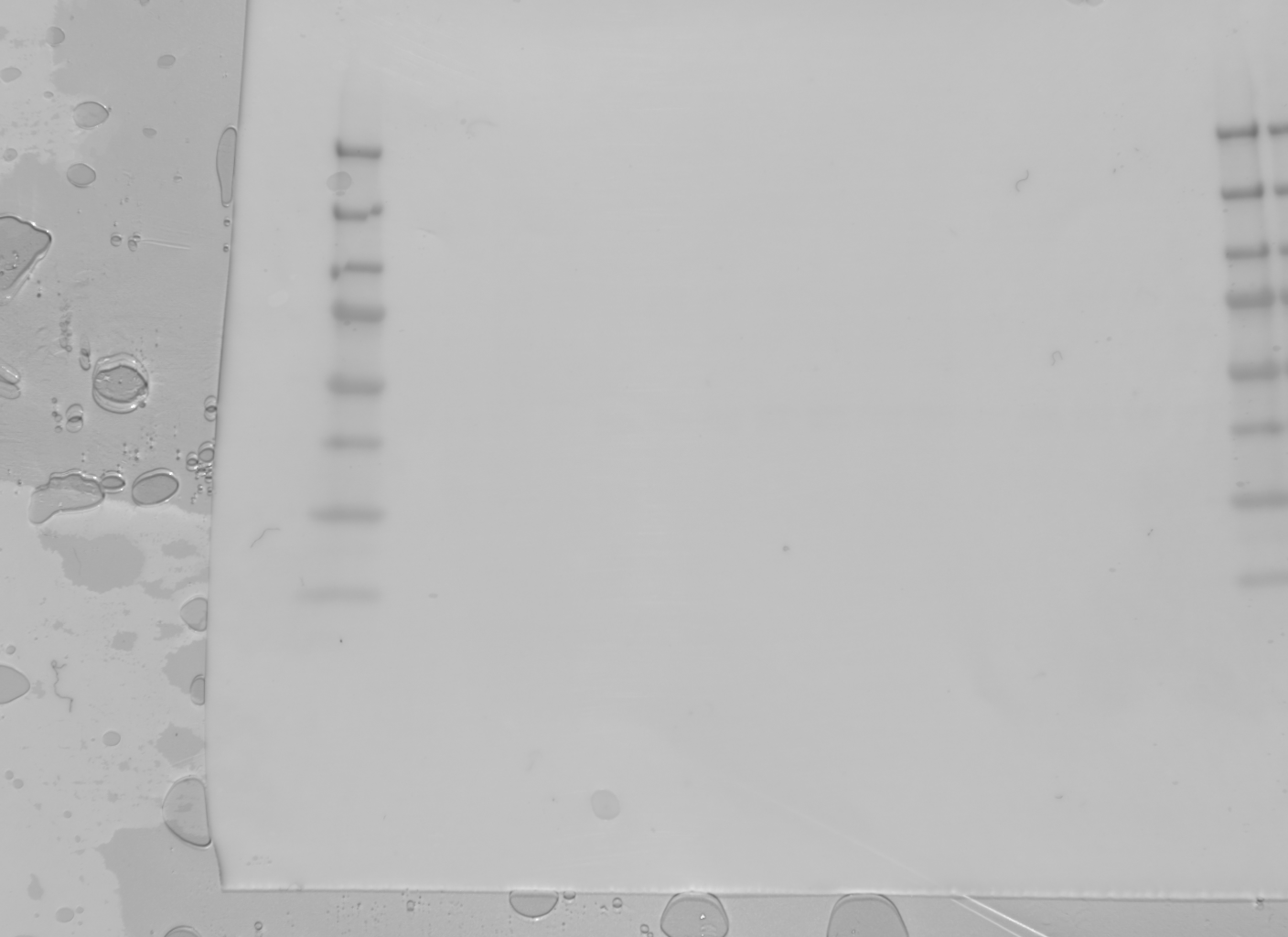

Supplement: Figure 6—source data 1. [file elife-80949-fig6-data1.zip › Figure 6 source data/Fig6H - PI3Kc3/MGP_PI3Kc3/MGP_PI3Kc3_Marker.tif]

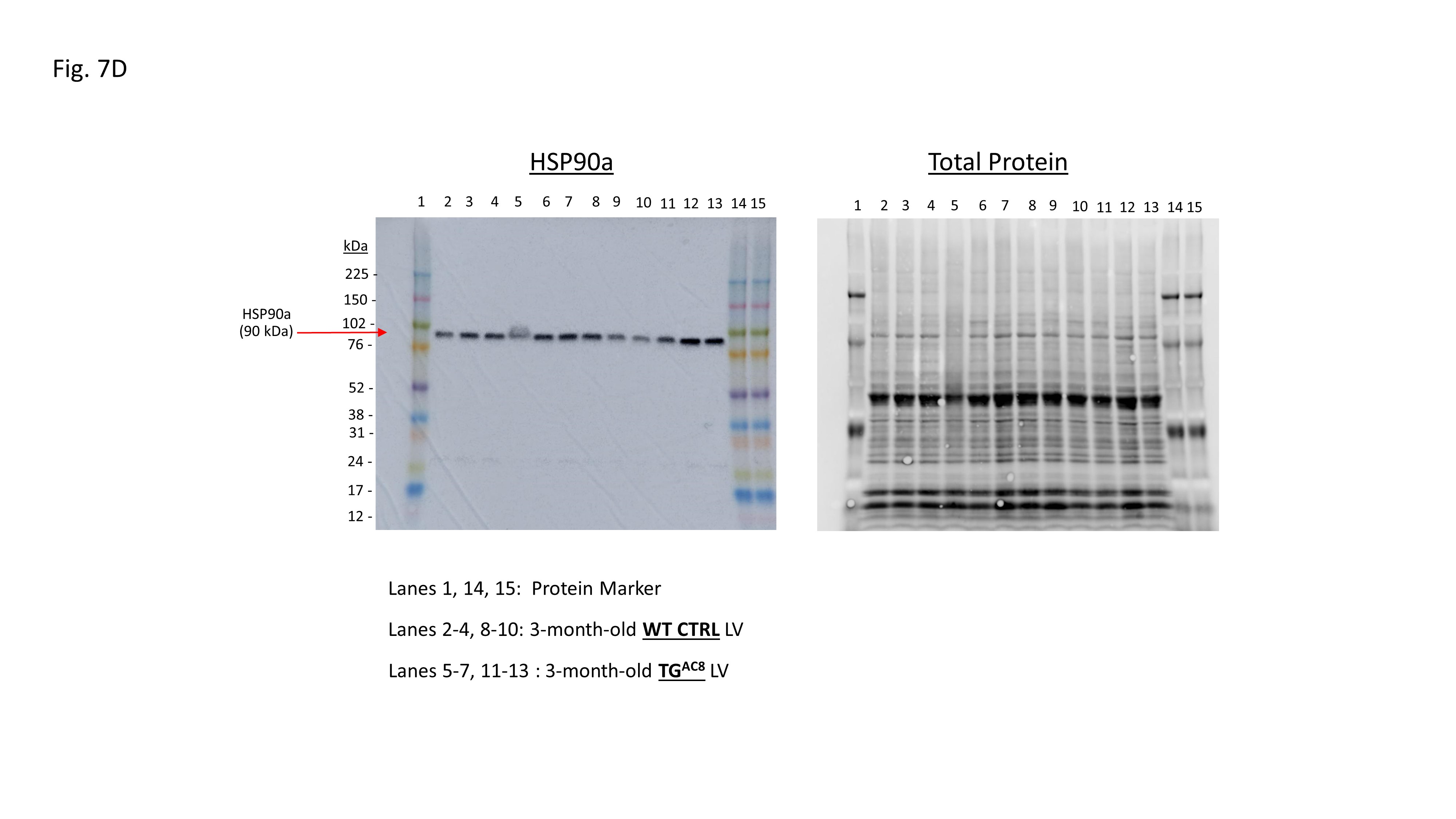

Supplement: Figure 6—source data 1. [file elife-80949-fig6-data1.zip › Figure 6 source data/Uncropped Images/6d.JPG]

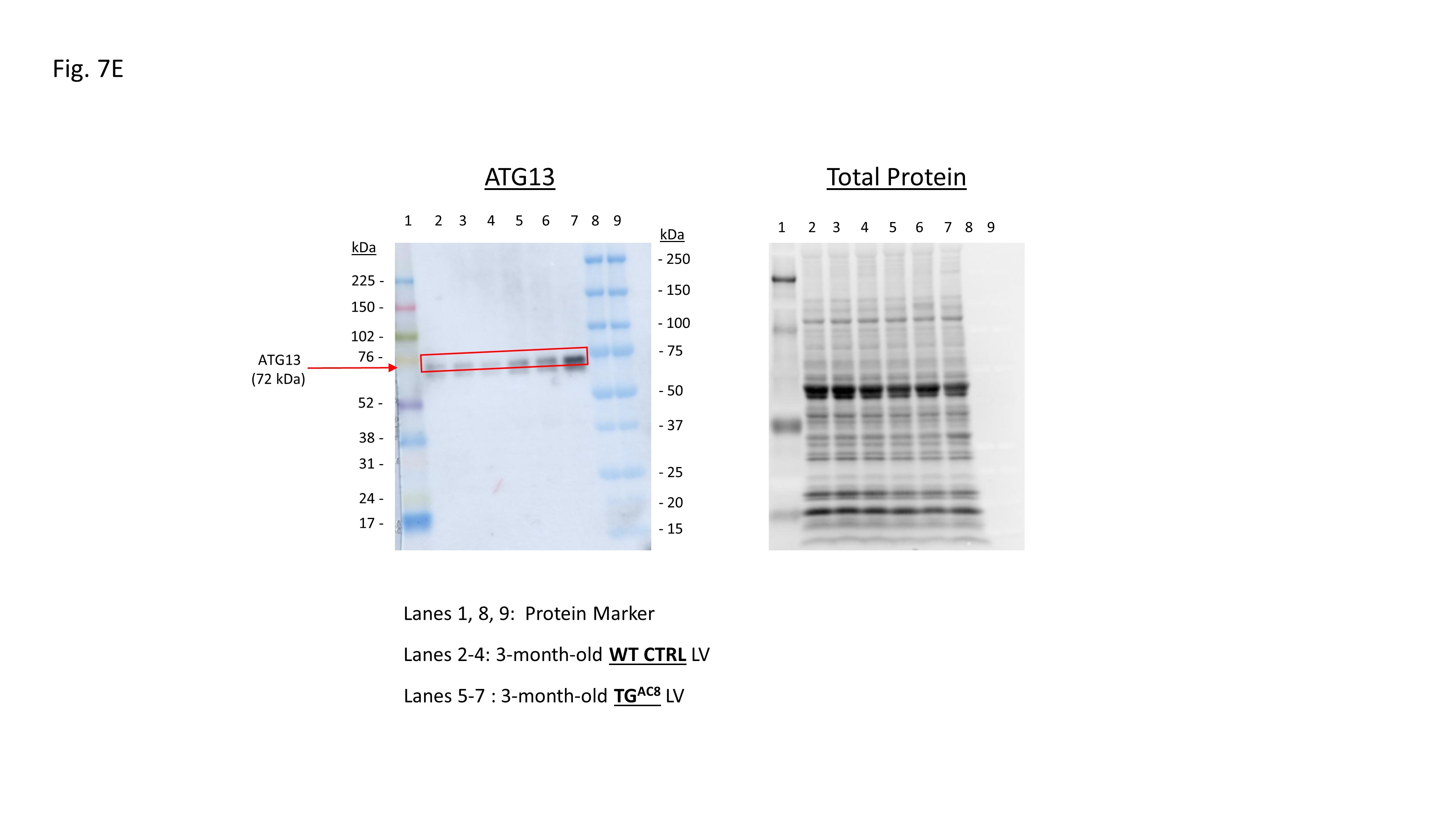

Supplement: Figure 6—source data 1. [file elife-80949-fig6-data1.zip › Figure 6 source data/Uncropped Images/6e.JPG]

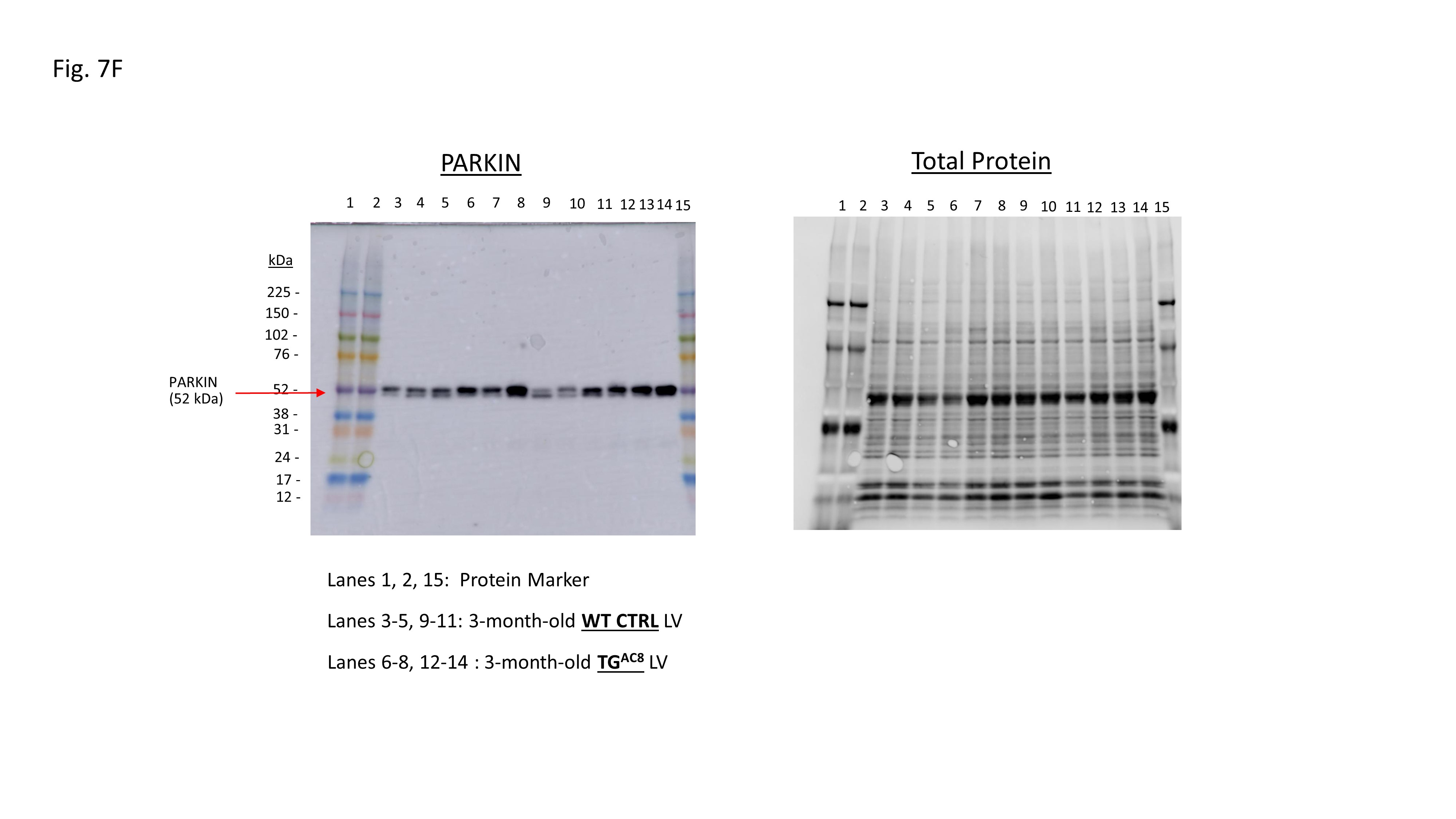

Supplement: Figure 6—source data 1. [file elife-80949-fig6-data1.zip › Figure 6 source data/Uncropped Images/6f.JPG]

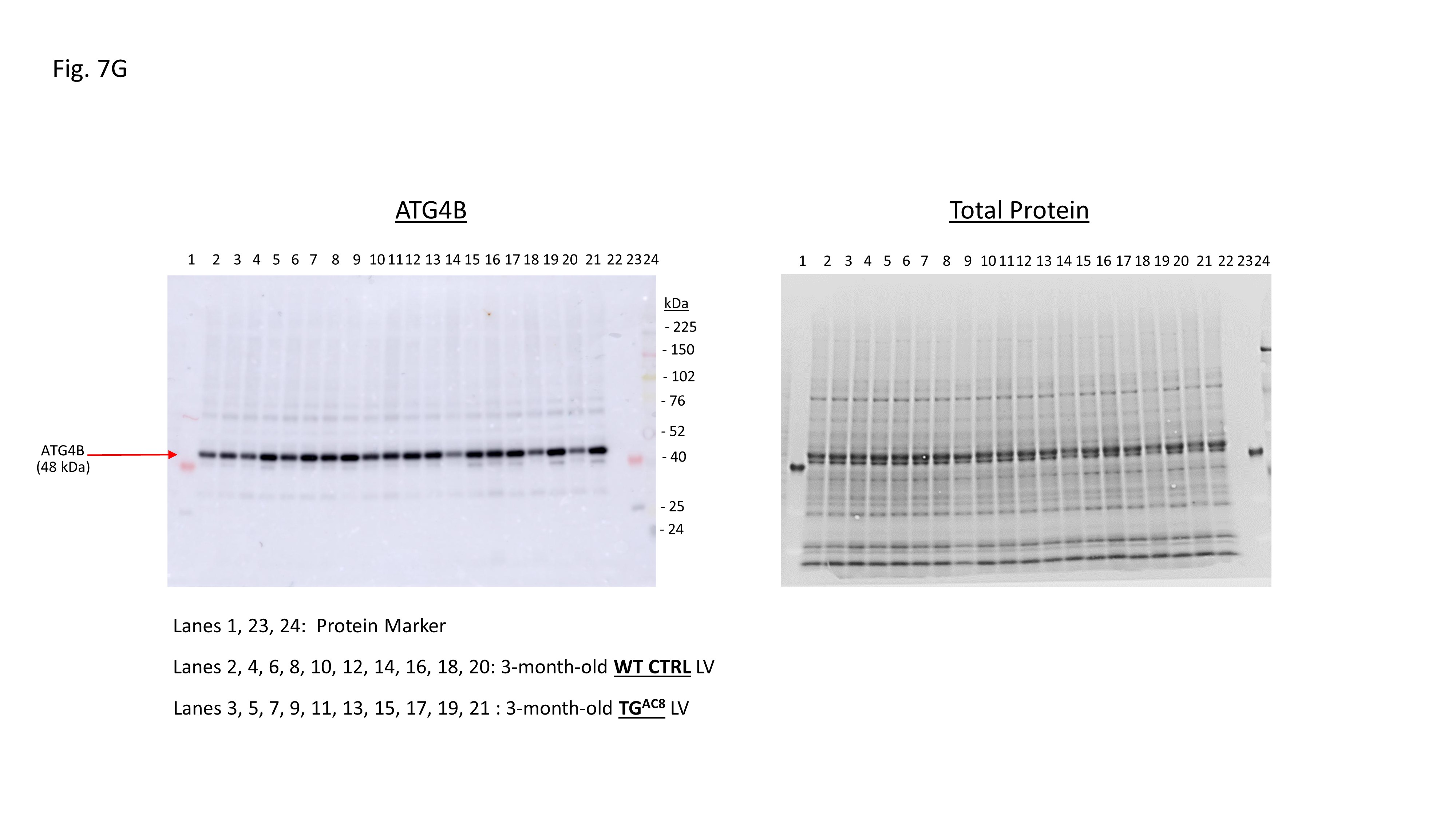

Supplement: Figure 6—source data 1. [file elife-80949-fig6-data1.zip › Figure 6 source data/Uncropped Images/6g.JPG]

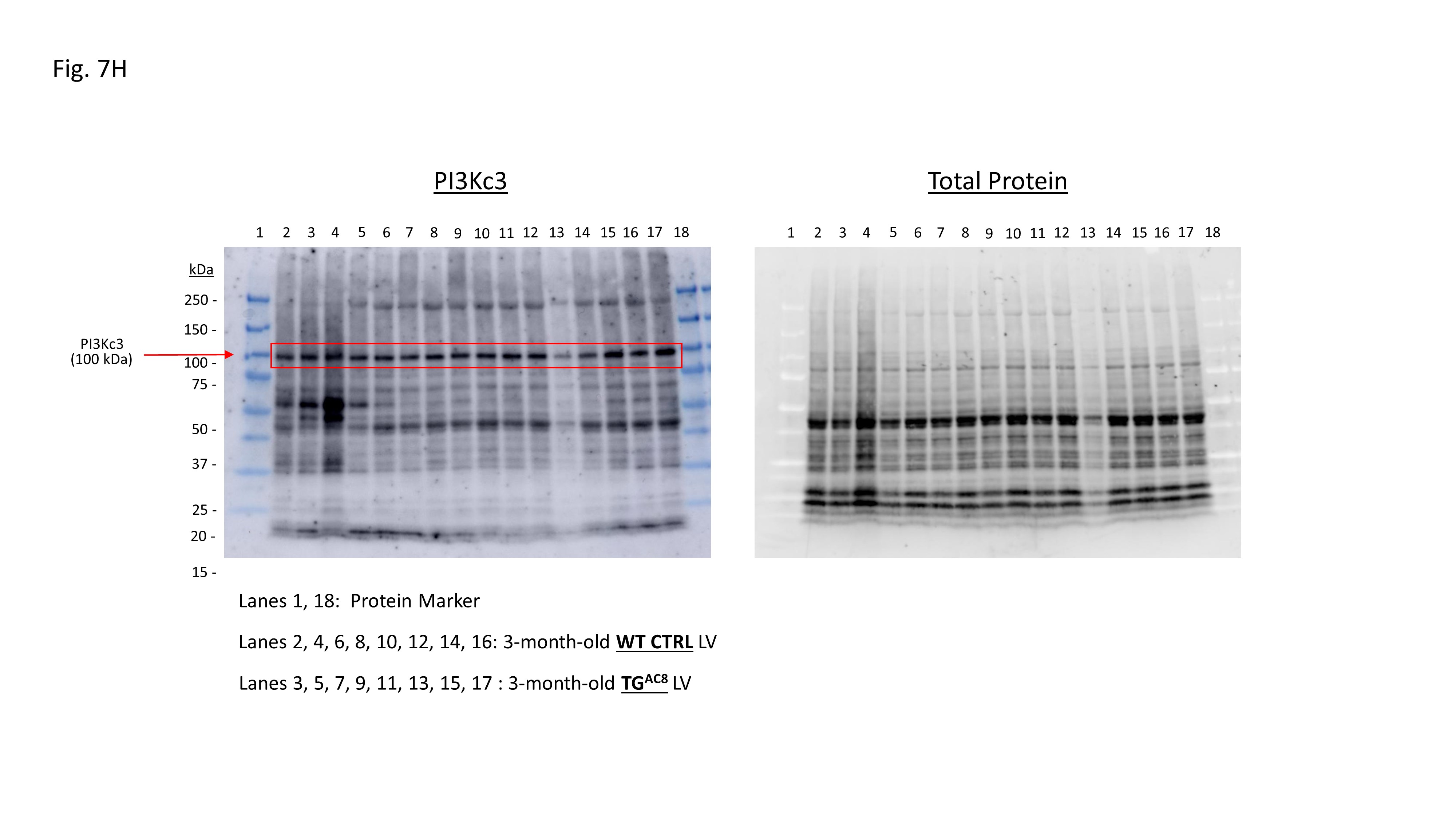

Supplement: Figure 6—source data 1. [file elife-80949-fig6-data1.zip › Figure 6 source data/Uncropped Images/6h.JPG]

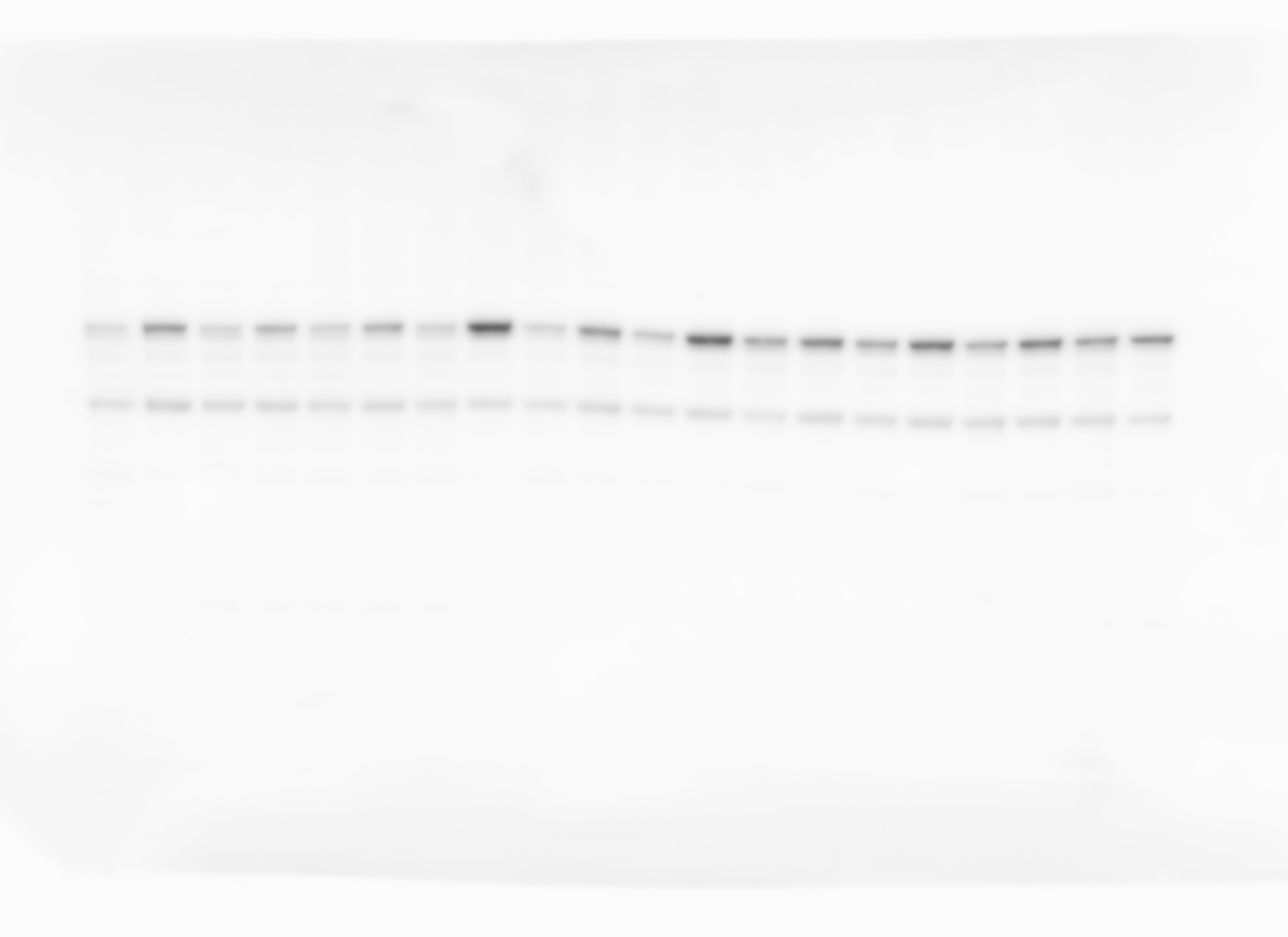

Supplement: Figure 8—source data 1. [file elife-80949-fig8-data1.zip › Figure 8 Source data/NRF2/NRF2/DR NRF2 Blt15 WPP 2018.03.30_12.15.22_Ch.tif]

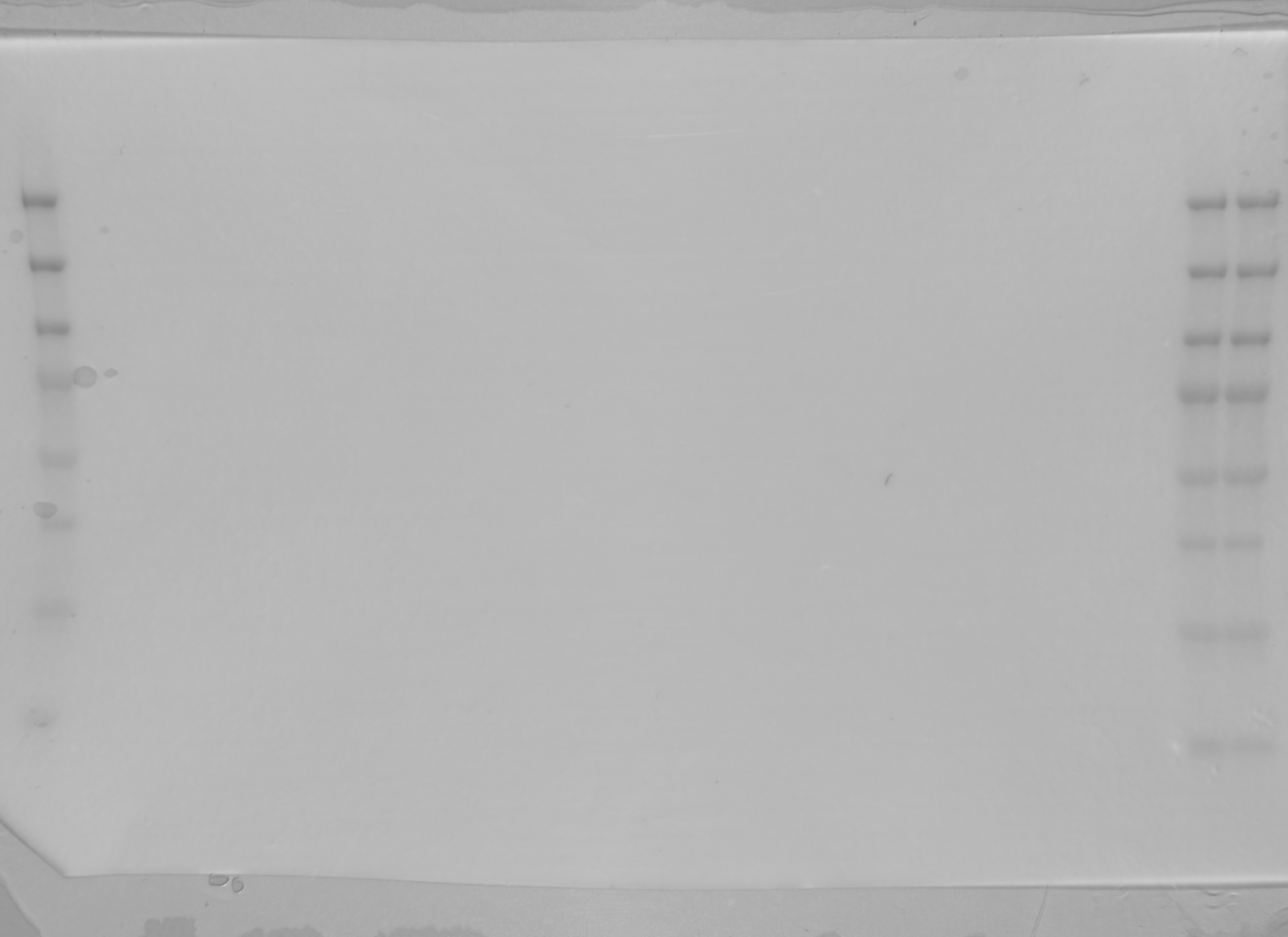

Supplement: Figure 8—source data 1. [file elife-80949-fig8-data1.zip › Figure 8 Source data/NRF2/NRF2/DR NRF2 Blt15 WPP 2018.03.30_12.15.22_Ch-Marker.tif]

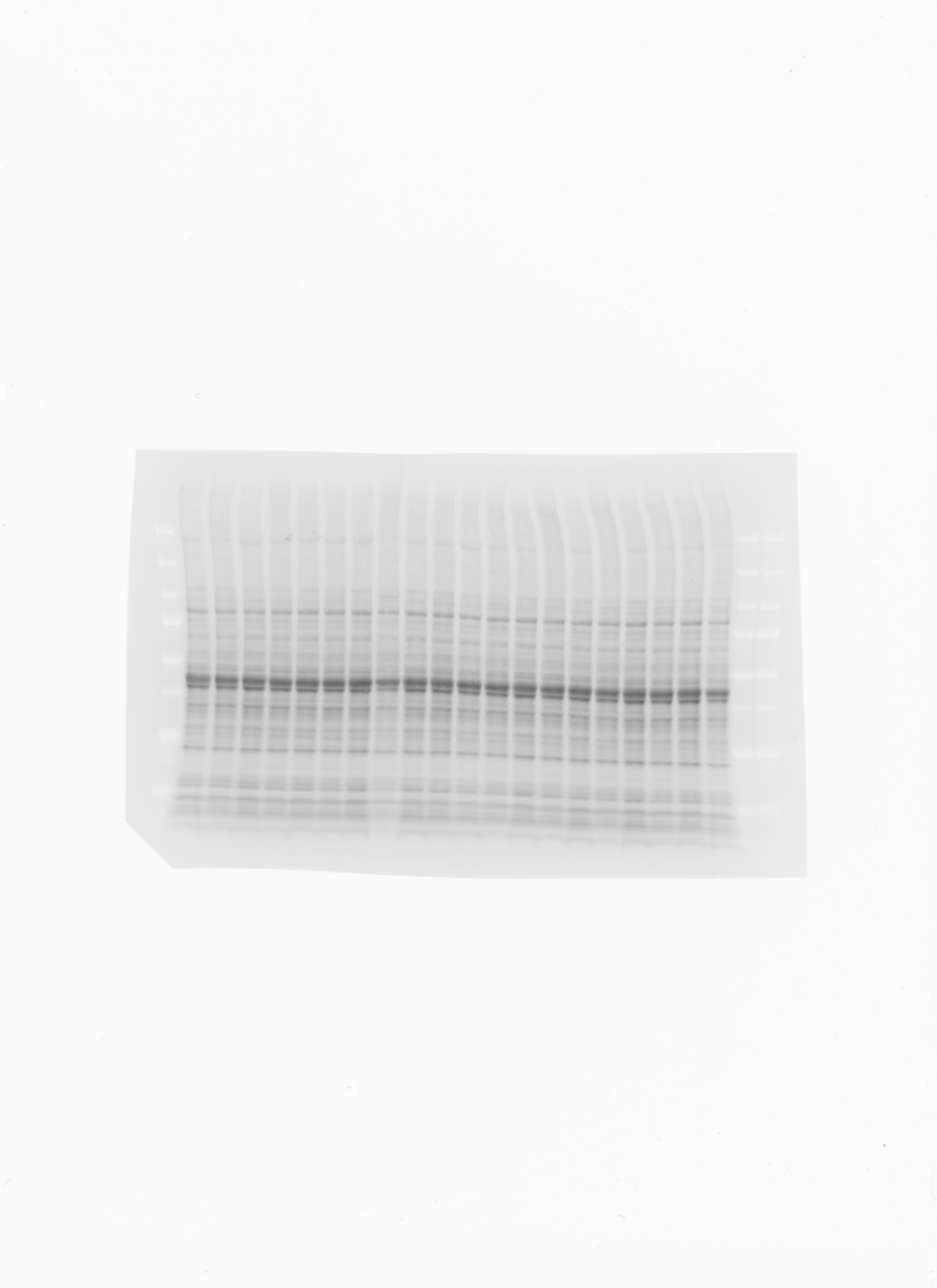

Supplement: Figure 8—source data 1. [file elife-80949-fig8-data1.zip › Figure 8 Source data/NRF2/Total Protein/DR TProt. LV Blot15 2018.03.20_11.46.56_Fl-UV.tif]

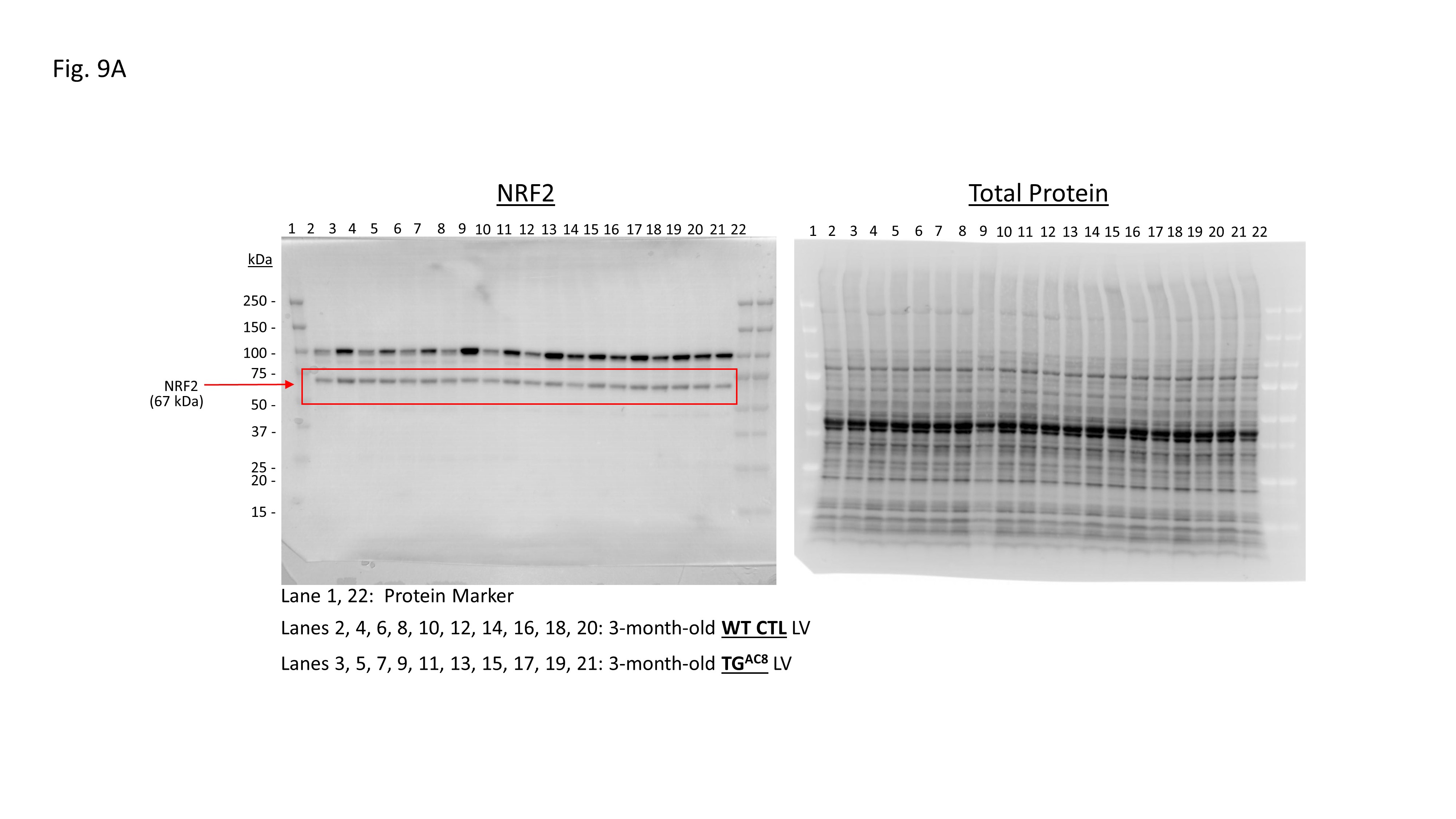

Supplement: Figure 8—source data 1. [file elife-80949-fig8-data1.zip › Figure 8 Source data/NRF2_Uncropped.JPG]

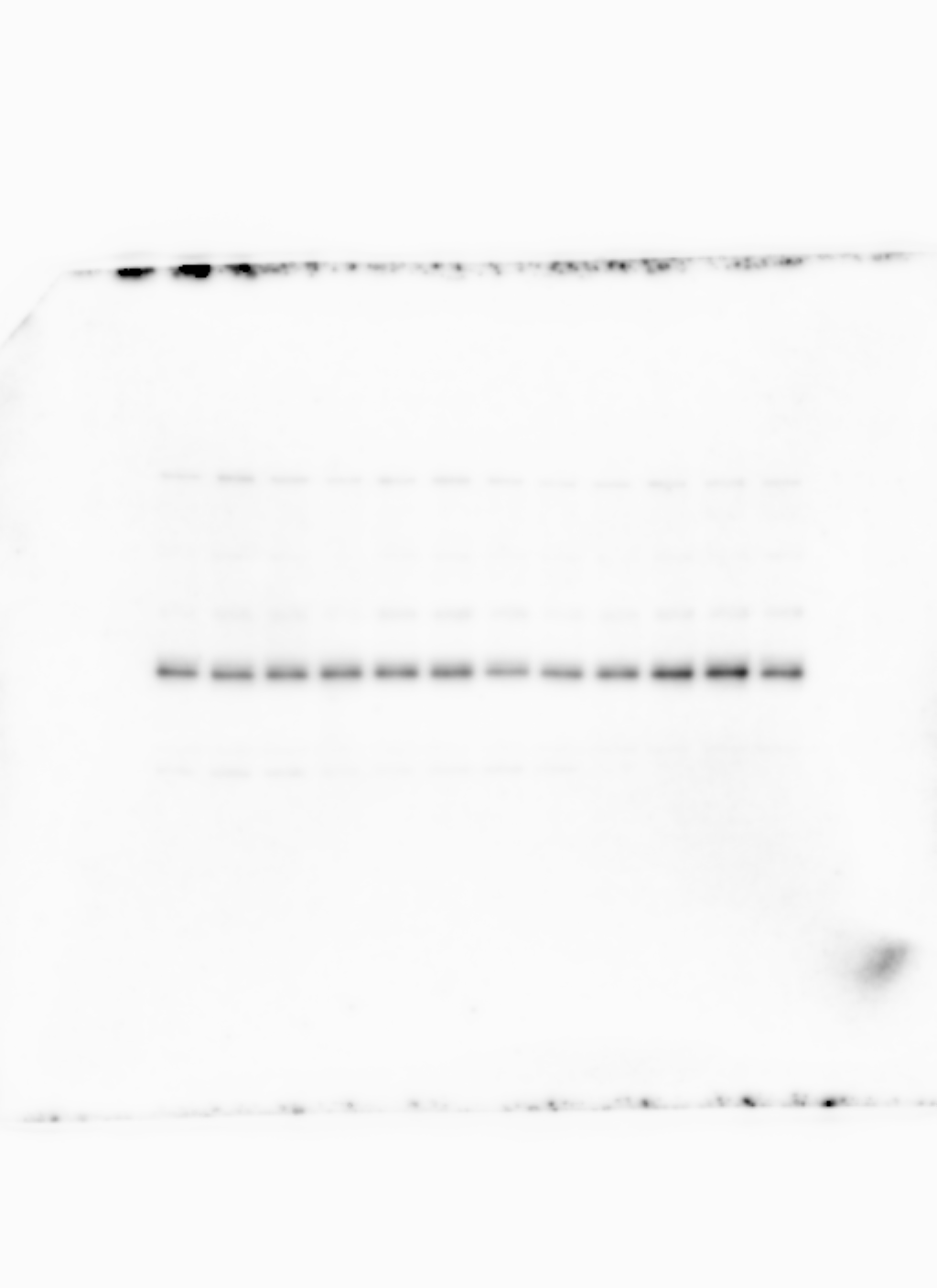

Supplement: Figure 12—figure supplement 1—source data 1. [file elife-80949-fig12-figsupp1-data1.zip › Figure 12-supplement 1 source data 1/AKT2/AKT2/DR AKT2 Blt82 2020.11.20_10.04.36_Ch.tif]

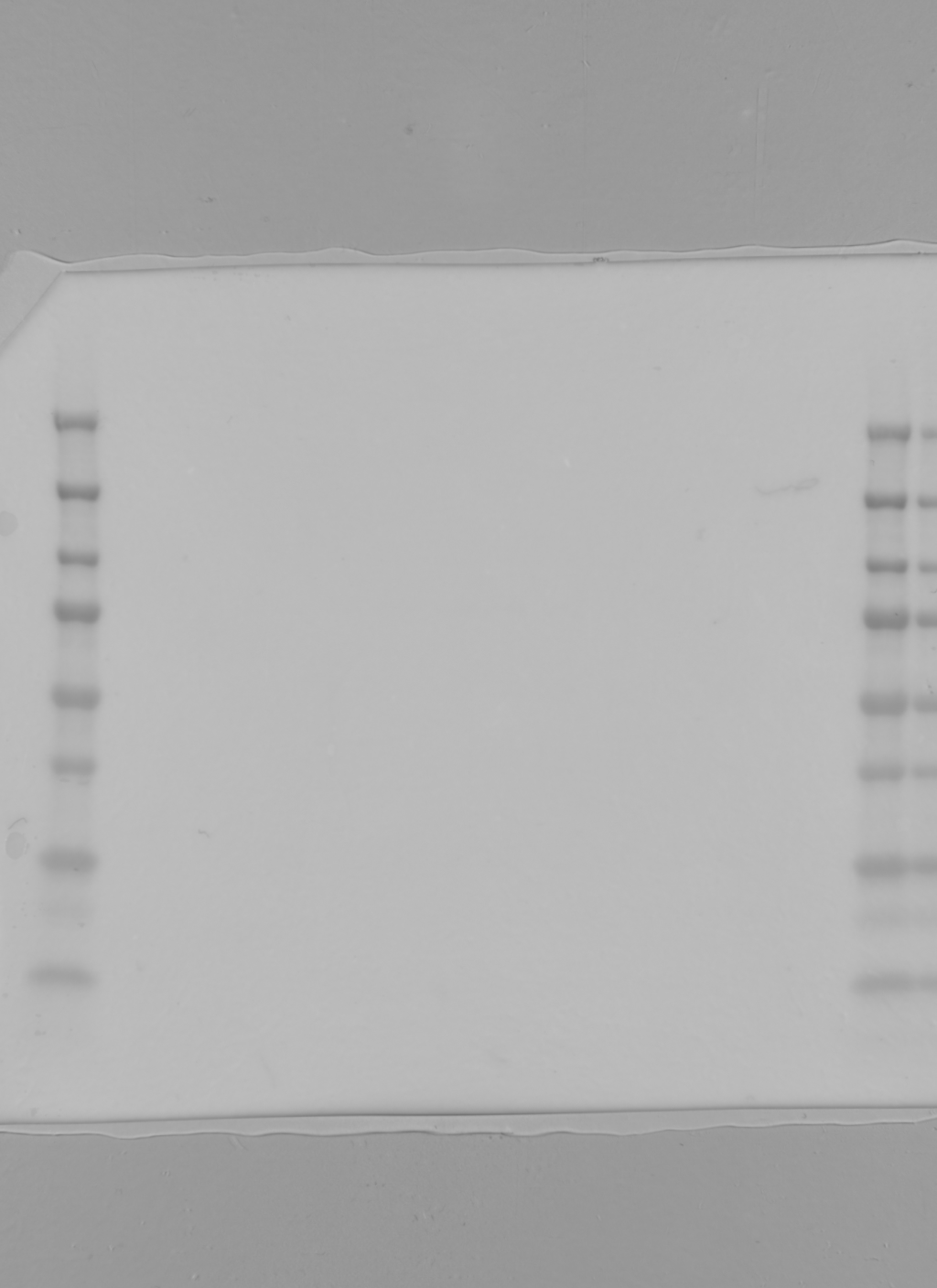

Supplement: Figure 12—figure supplement 1—source data 1. [file elife-80949-fig12-figsupp1-data1.zip › Figure 12-supplement 1 source data 1/AKT2/AKT2/DR AKT2 Blt82 2020.11.20_10.04.36_Ch-Marker.tif]

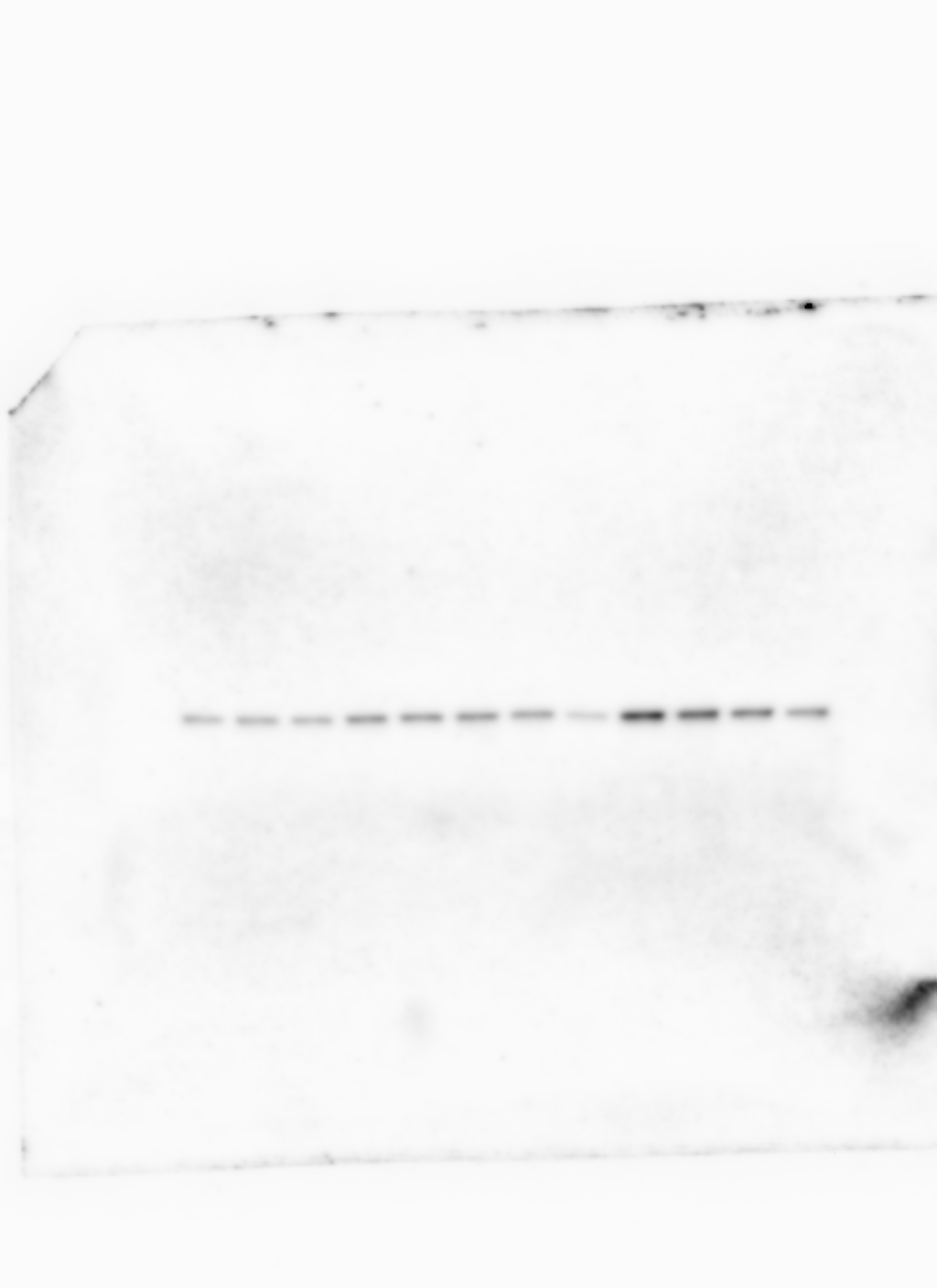

Supplement: Figure 12—figure supplement 1—source data 1. [file elife-80949-fig12-figsupp1-data1.zip › Figure 12-supplement 1 source data 1/AKT2/p-AKT2 Ser473/DR pAKT2 S473 blt82 2020.11.19_11.28.26_Ch.tif]

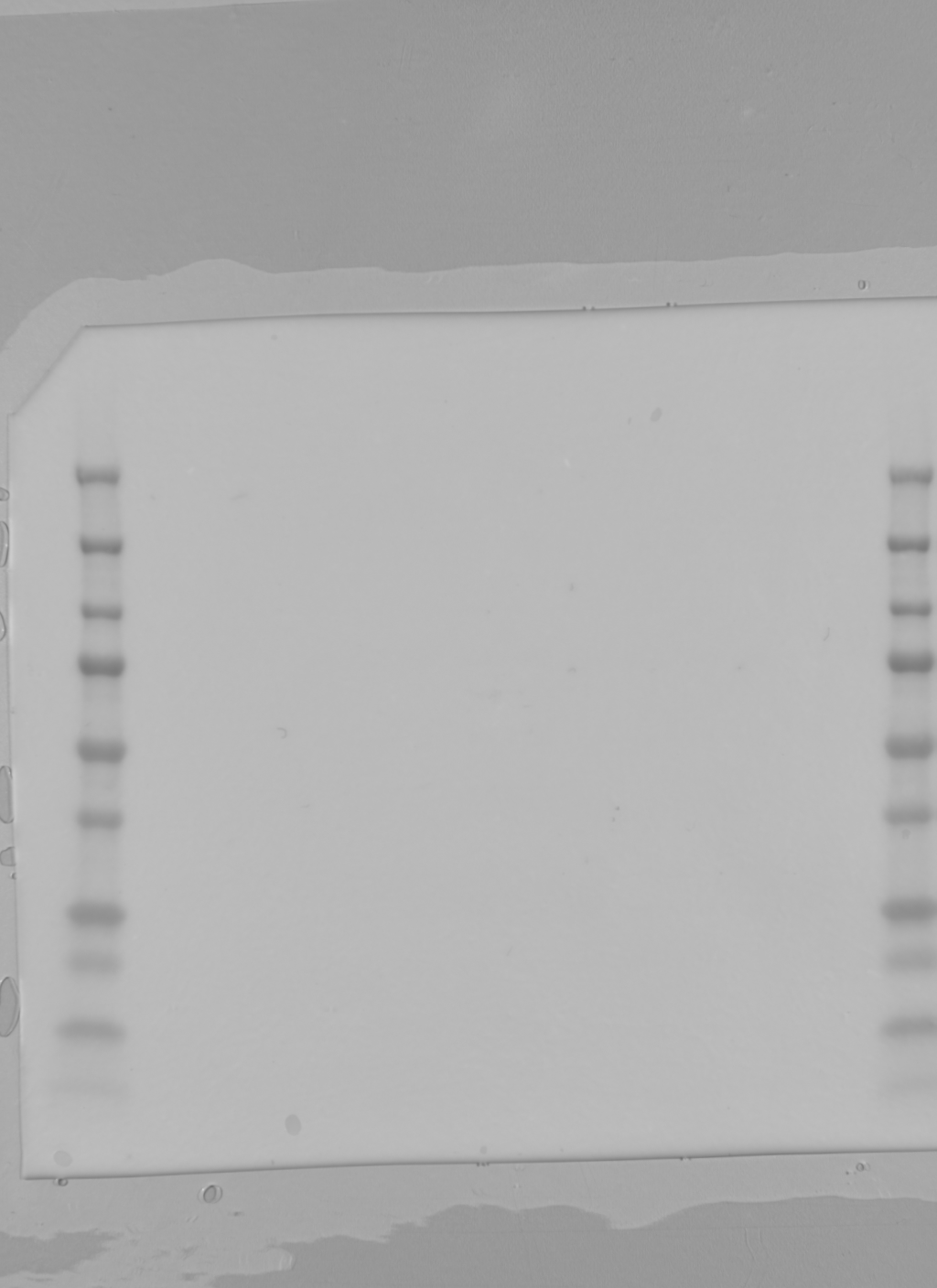

Supplement: Figure 12—figure supplement 1—source data 1. [file elife-80949-fig12-figsupp1-data1.zip › Figure 12-supplement 1 source data 1/AKT2/p-AKT2 Ser473/DR pAKT2 S473 blt82 2020.11.19_11.28.26_Ch-Marker.tif]

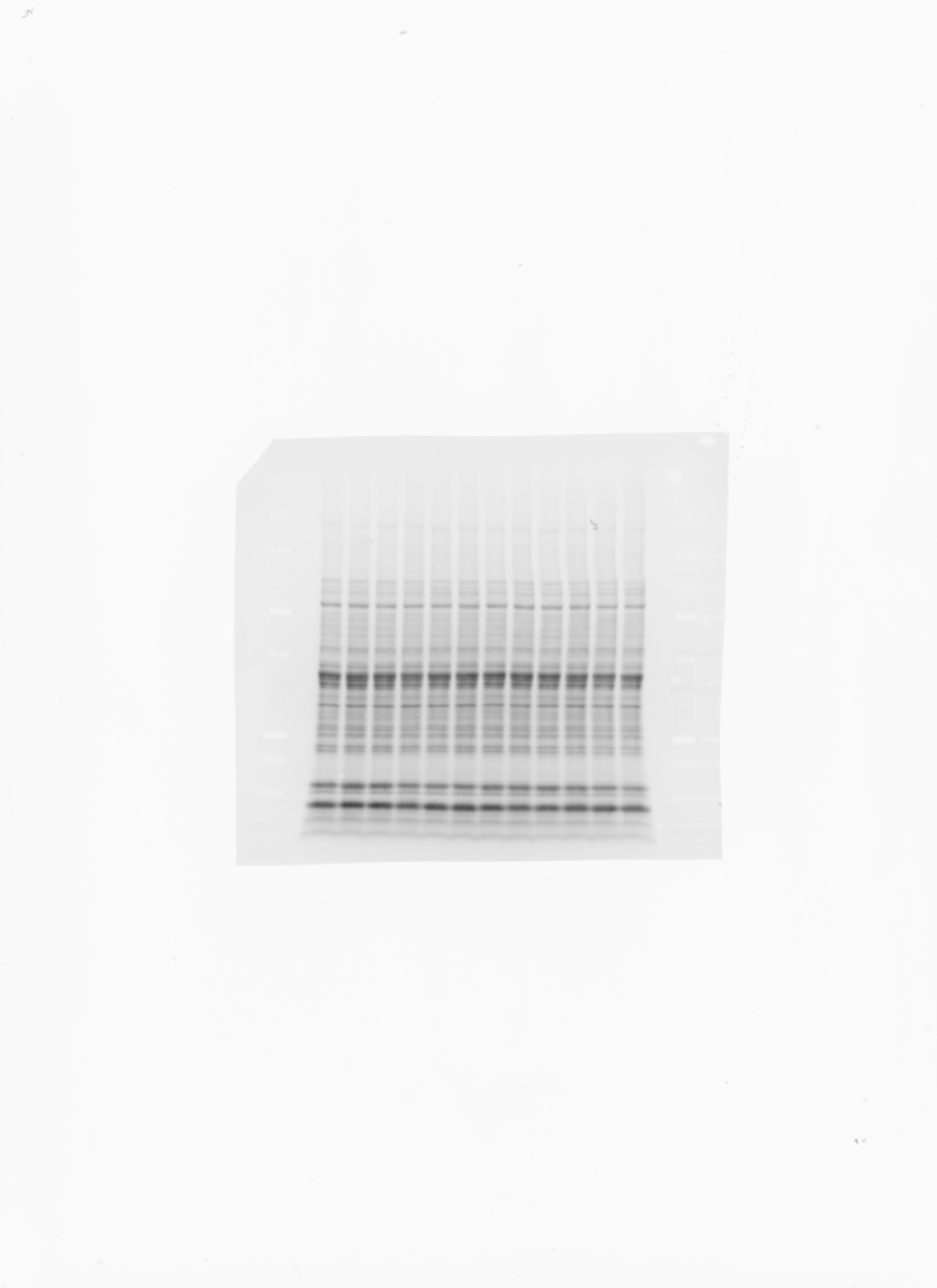

Supplement: Figure 12—figure supplement 1—source data 1. [file elife-80949-fig12-figsupp1-data1.zip › Figure 12-supplement 1 source data 1/AKT2/Total Protein/DR TProt Blot82 2020.11.18_10.34.52_Fl-UV.tif]

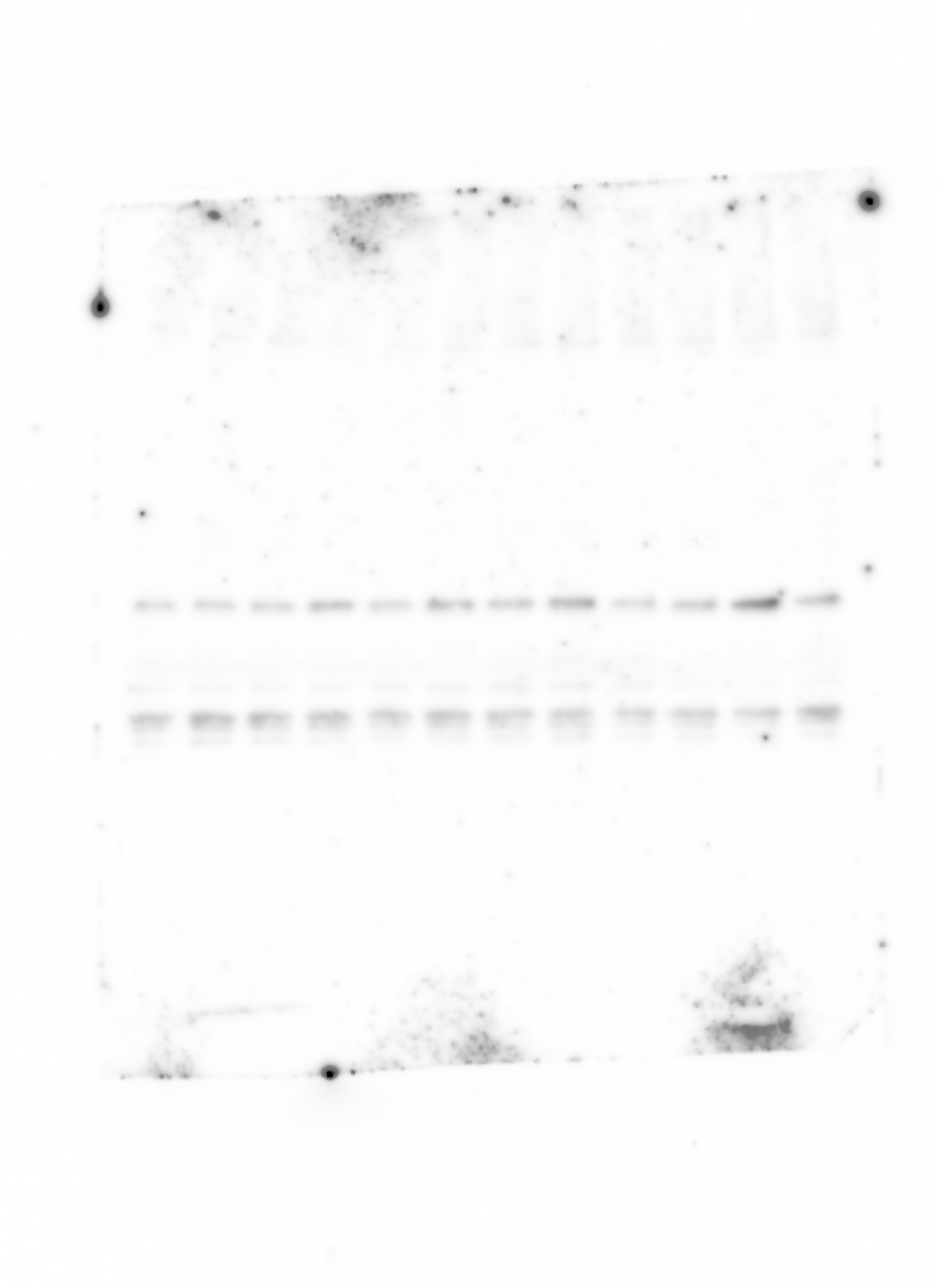

Supplement: Figure 12—figure supplement 1—source data 1. [file elife-80949-fig12-figsupp1-data1.zip › Figure 12-supplement 1 source data 1/G6PDH/G6PDH/DR G6PDH Blt93 2021.02.12_13.34.57_Ch.tif]

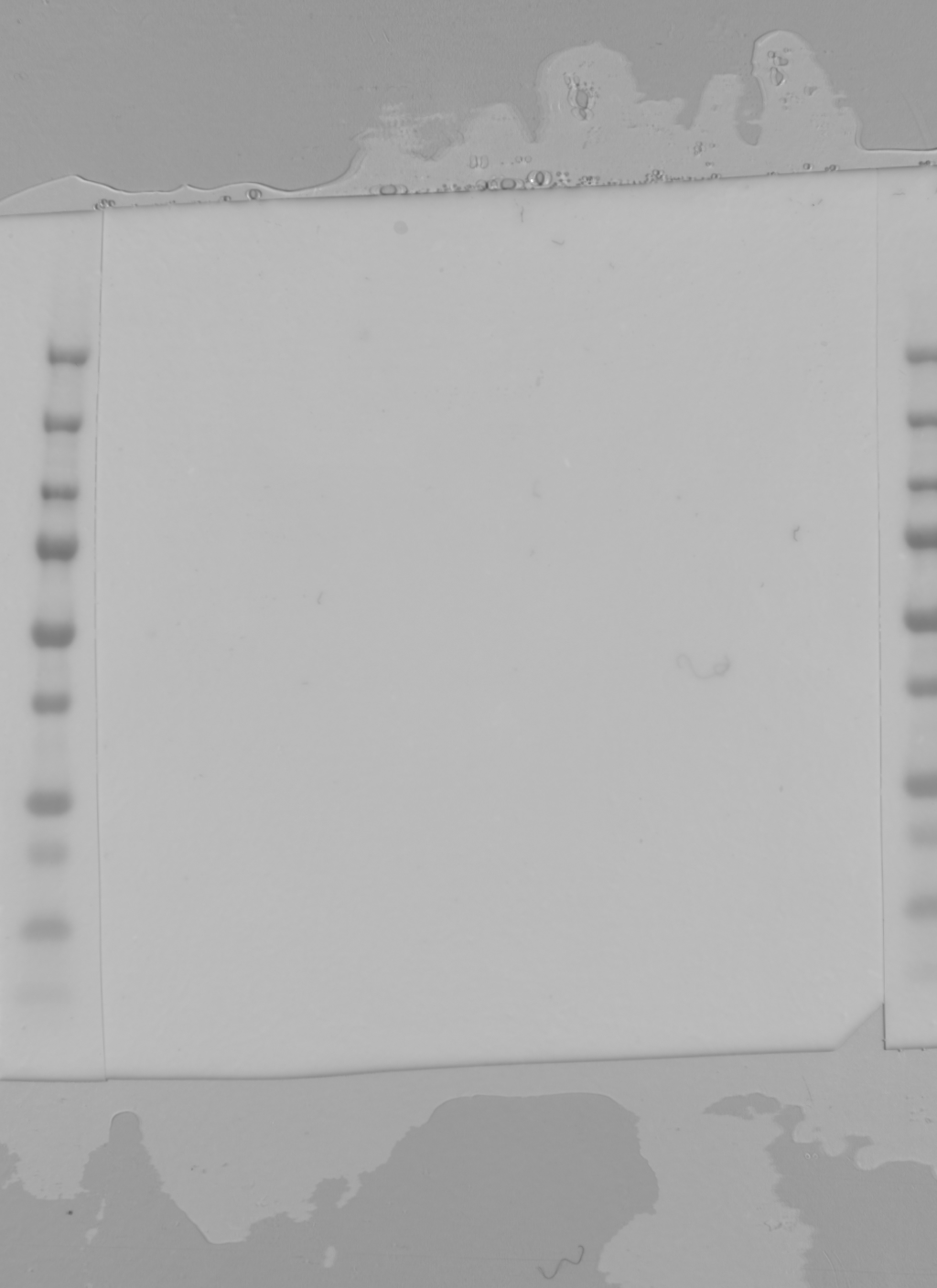

Supplement: Figure 12—figure supplement 1—source data 1. [file elife-80949-fig12-figsupp1-data1.zip › Figure 12-supplement 1 source data 1/G6PDH/G6PDH/DR G6PDH Blt93 2021.02.12_13.34.57_Ch-Marker.tif]

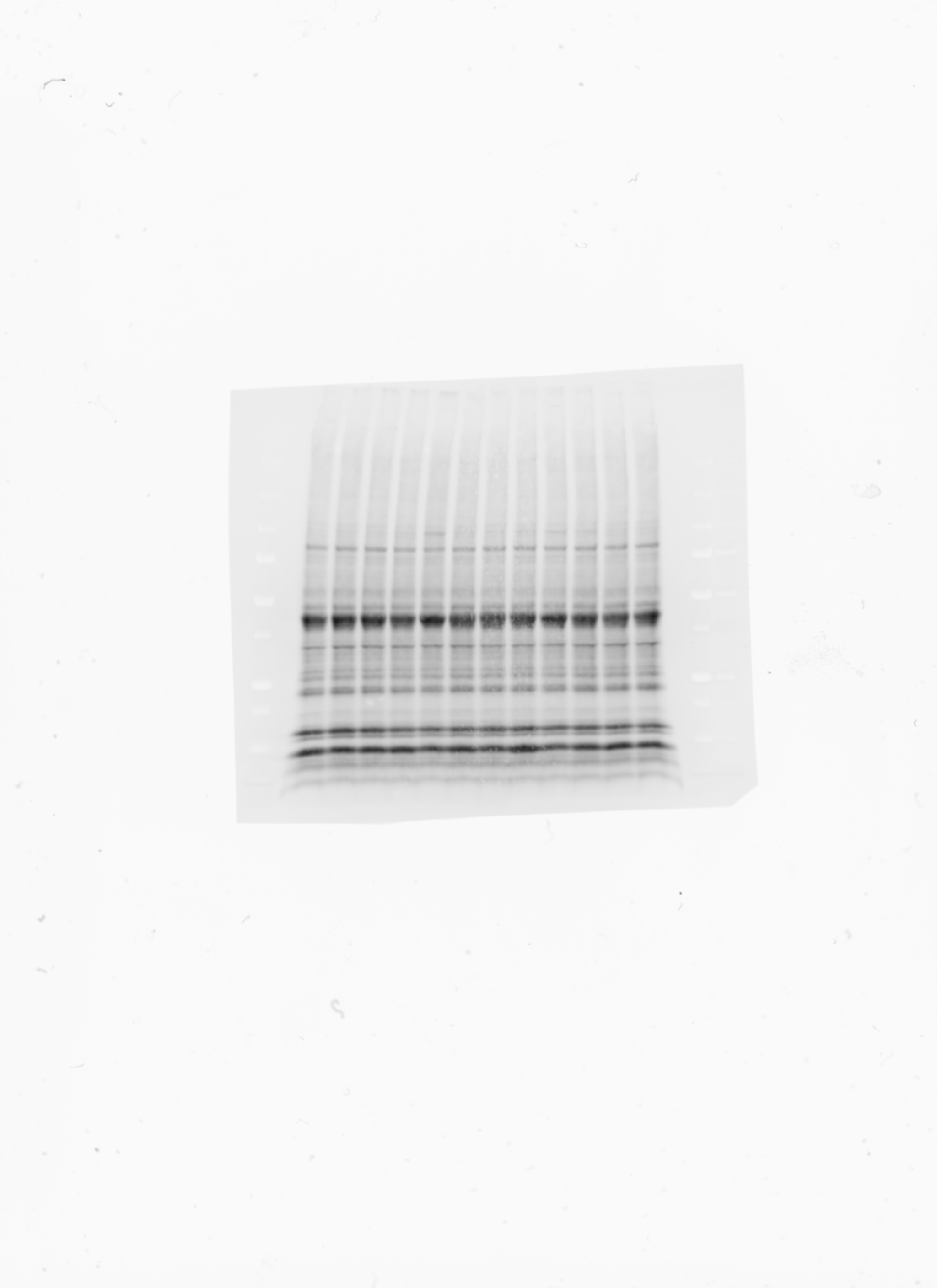

Supplement: Figure 12—figure supplement 1—source data 1. [file elife-80949-fig12-figsupp1-data1.zip › Figure 12-supplement 1 source data 1/G6PDH/Total Protein/DR T.Prot Blot93 2021.02.08_13.49.37_Fl-UV.tif]

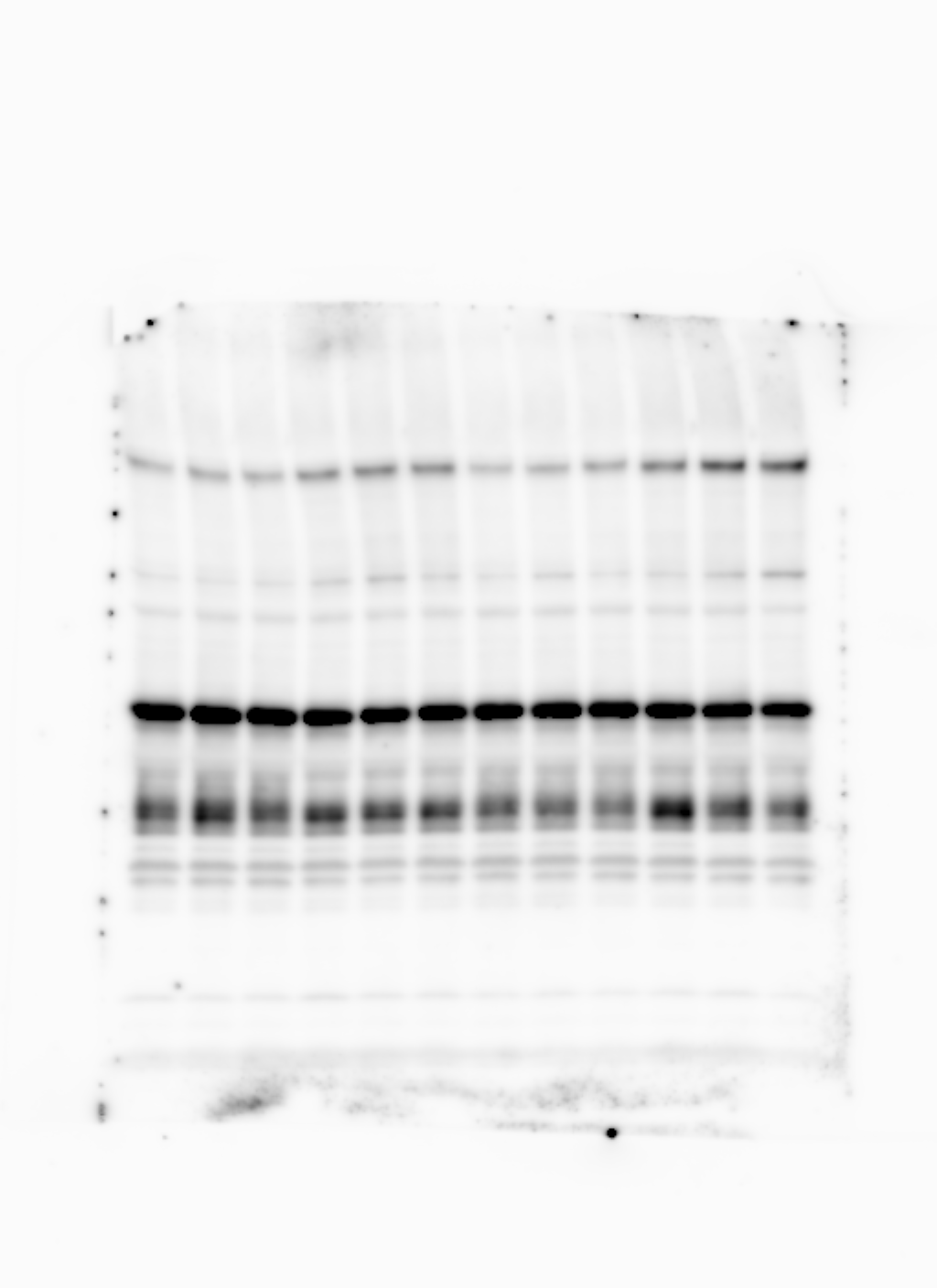

Supplement: Figure 12—figure supplement 1—source data 1. [file elife-80949-fig12-figsupp1-data1.zip › Figure 12-supplement 1 source data 1/GPD1/GPD1/DR GPD1 Blot85 2021.01.20_12.45.14_Ch.tif]

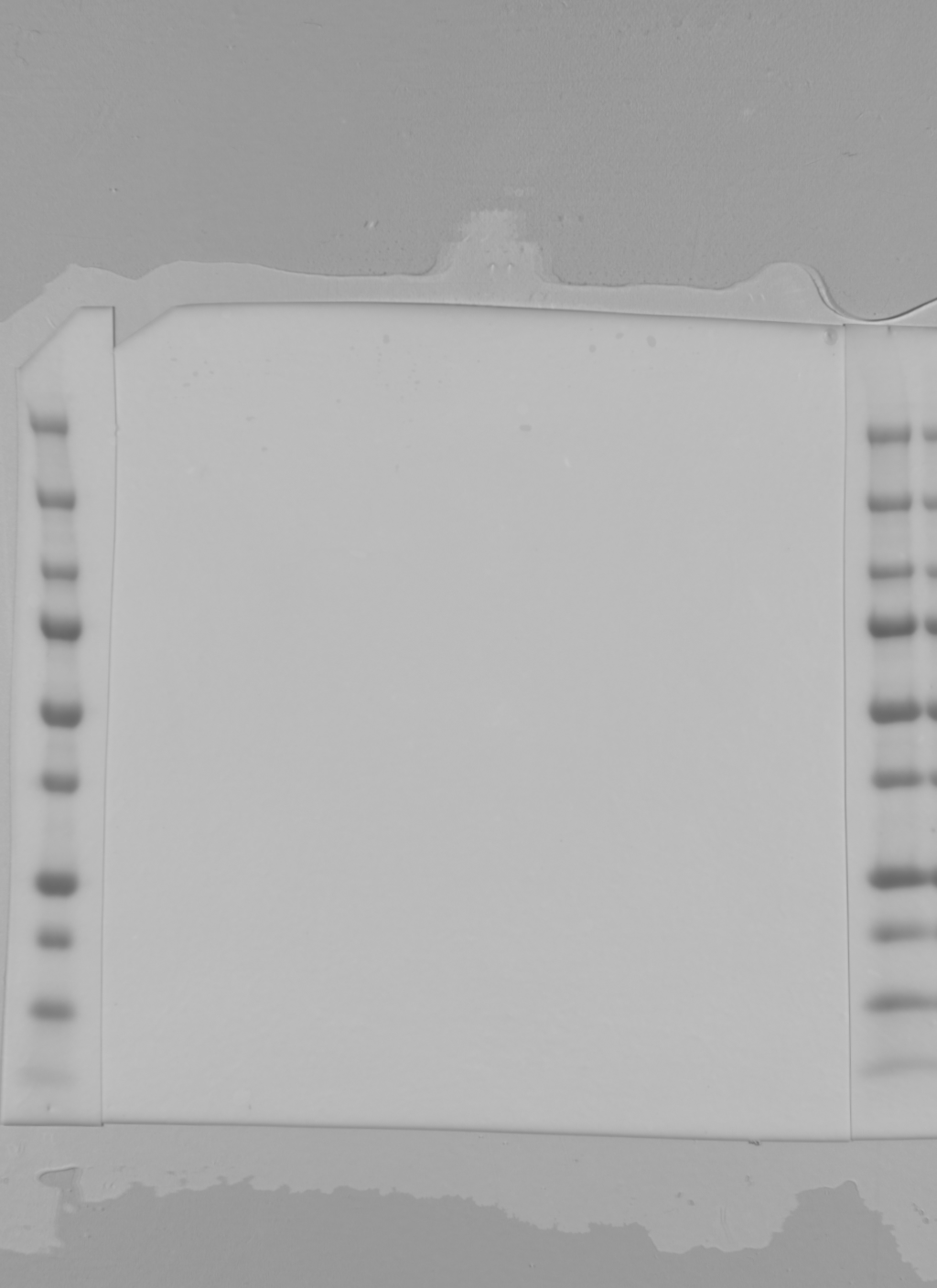

Supplement: Figure 12—figure supplement 1—source data 1. [file elife-80949-fig12-figsupp1-data1.zip › Figure 12-supplement 1 source data 1/GPD1/GPD1/DR GPD1 Blot85 2021.01.20_12.45.14_Ch-Marker.tif]

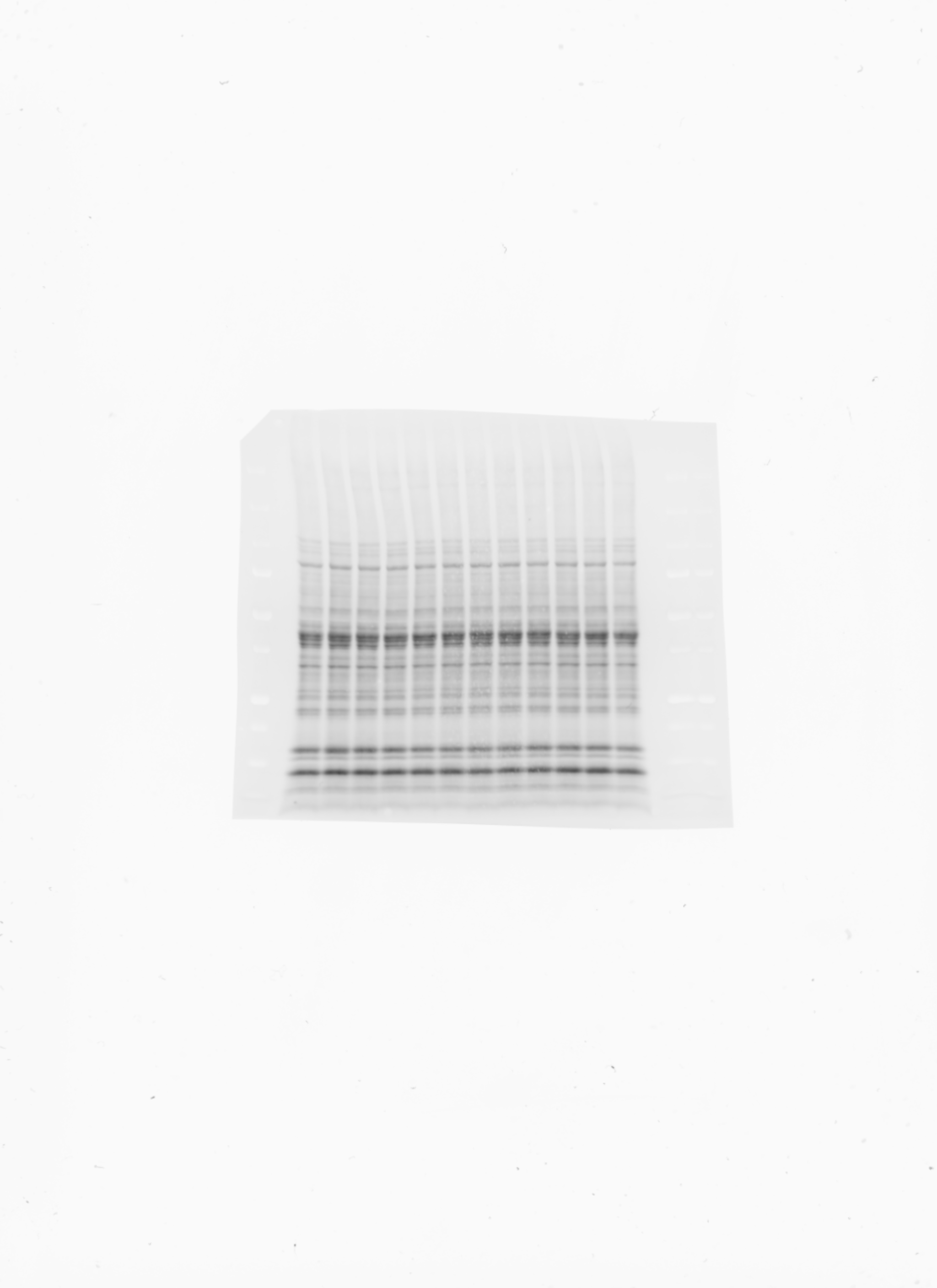

Supplement: Figure 12—figure supplement 1—source data 1. [file elife-80949-fig12-figsupp1-data1.zip › Figure 12-supplement 1 source data 1/GPD1/Total Protein/DR T.Prot. Blot85 2021.01.19_14.18.17_Fl-UV.tif]

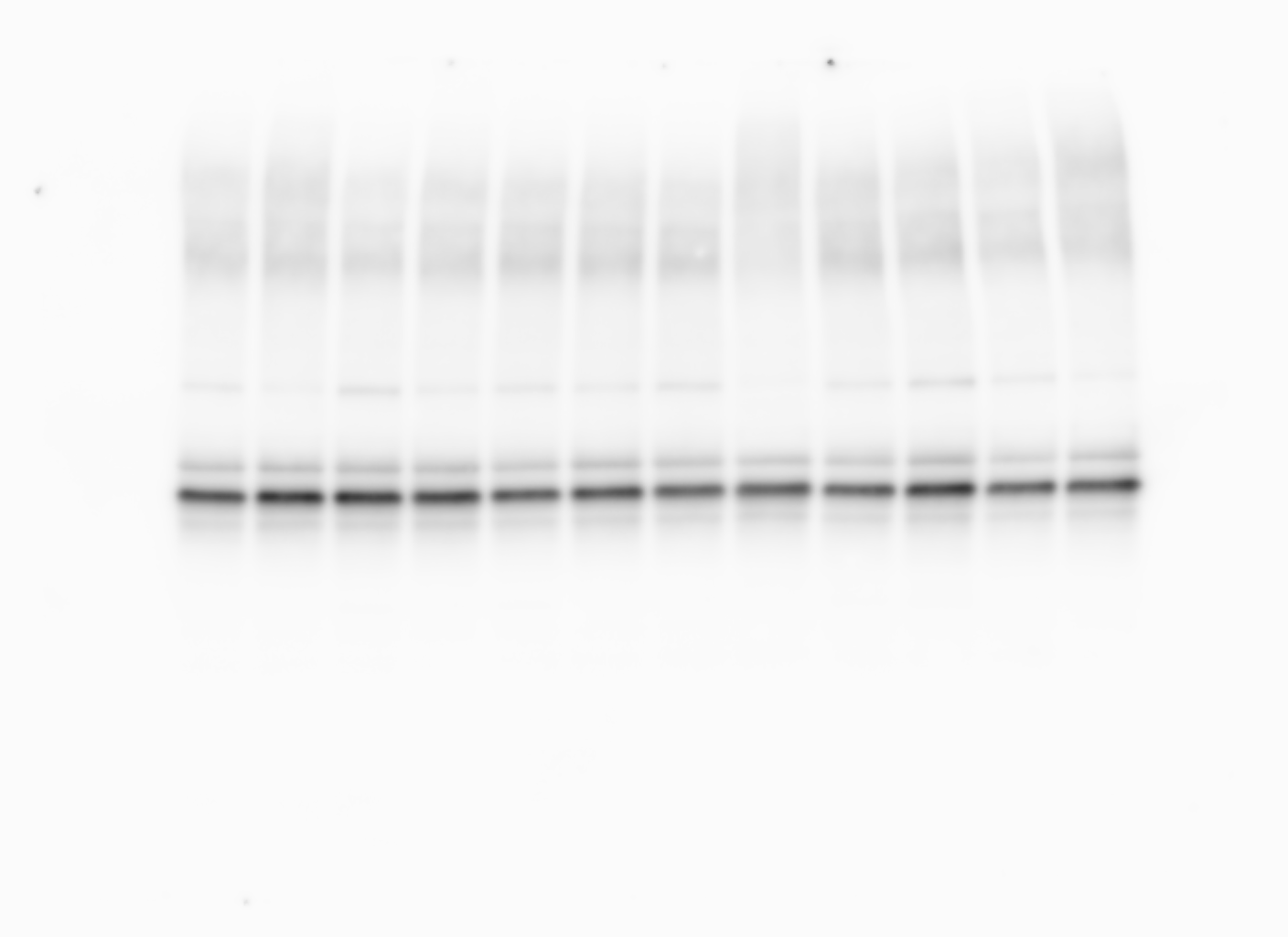

Supplement: Figure 12—figure supplement 1—source data 1. [file elife-80949-fig12-figsupp1-data1.zip › Figure 12-supplement 1 source data 1/GSK3b/GSK3b/DR GSK3ab WPP 2018.02.09_12.19.59_Ch.tif]

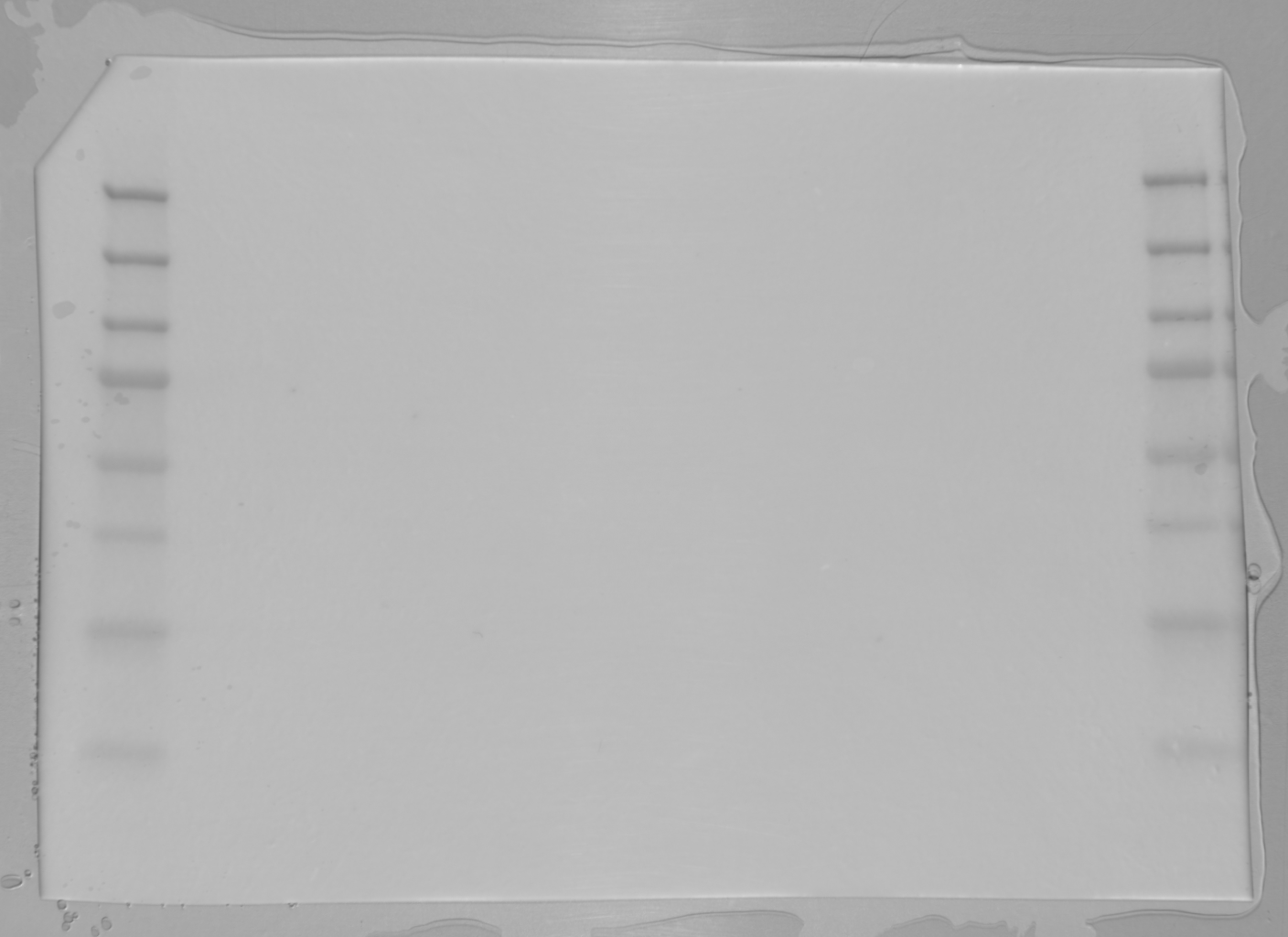

Supplement: Figure 12—figure supplement 1—source data 1. [file elife-80949-fig12-figsupp1-data1.zip › Figure 12-supplement 1 source data 1/GSK3b/GSK3b/DR GSK3ab WPP 2018.02.09_12.19.59_Ch-Marker.tif]

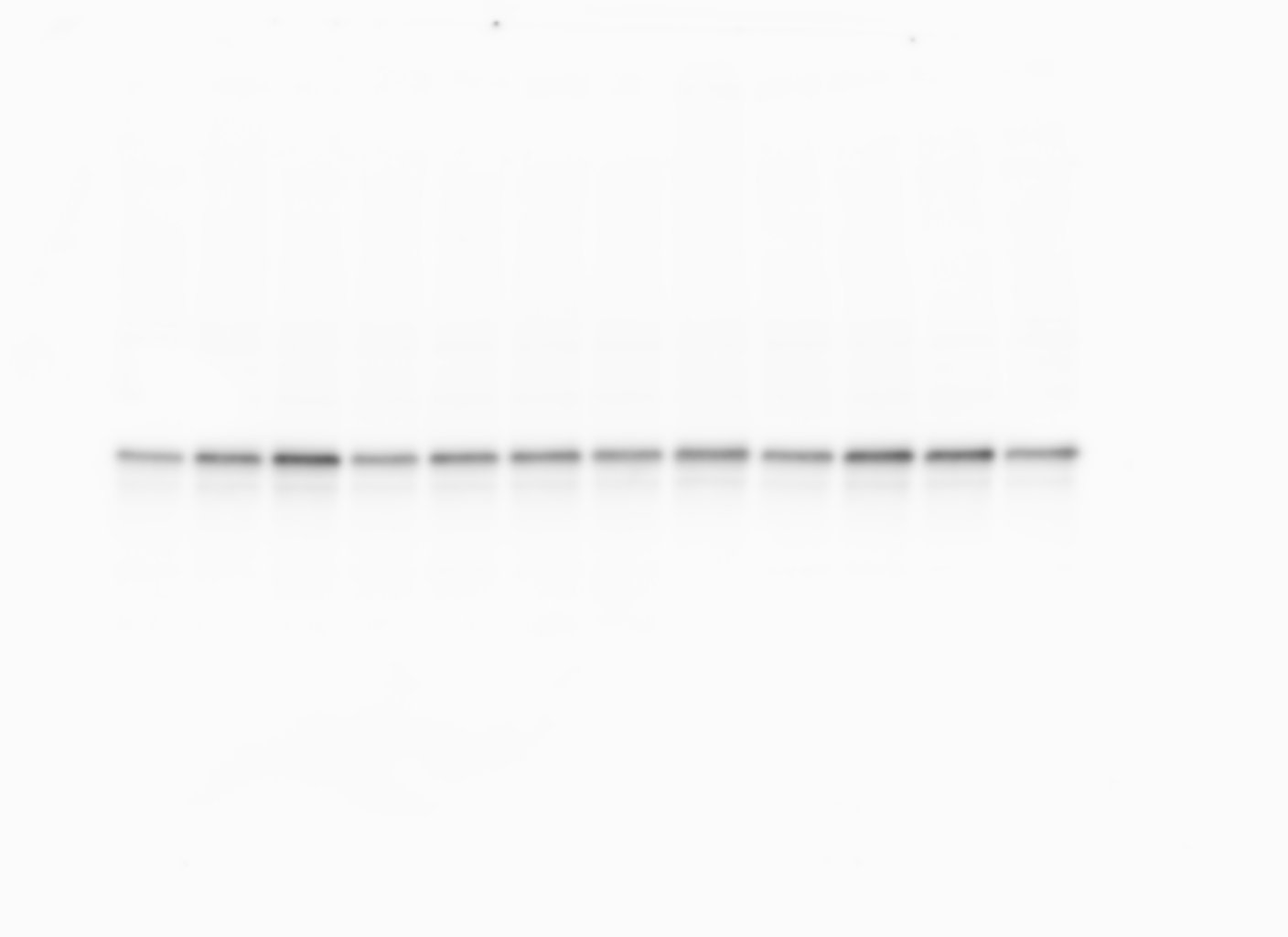

Supplement: Figure 12—figure supplement 1—source data 1. [file elife-80949-fig12-figsupp1-data1.zip › Figure 12-supplement 1 source data 1/GSK3b/p-GSK3b Ser9/DR pGSKb S9 WPP 2018.02.08_12.09.24_Ch.tif]

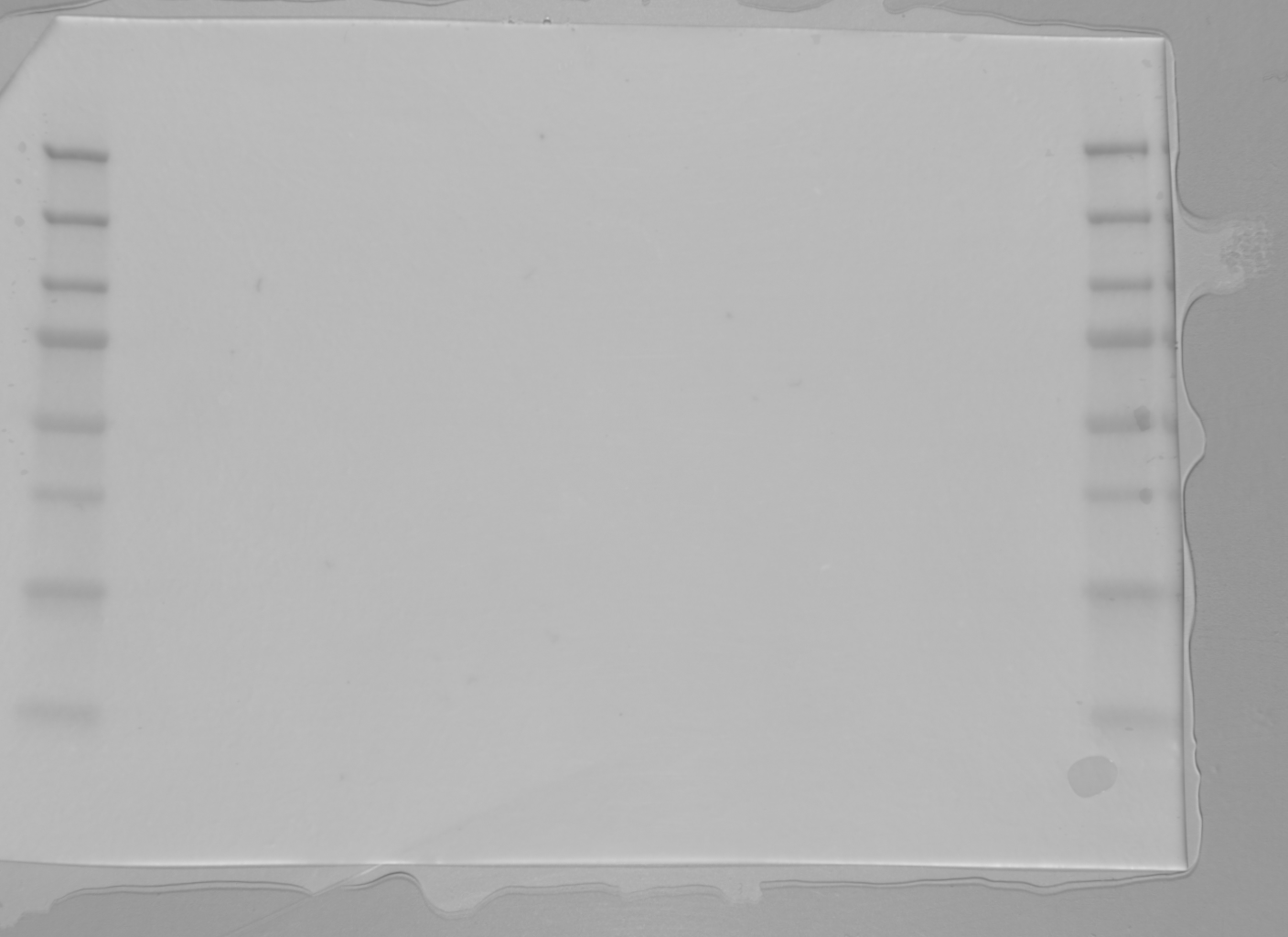

Supplement: Figure 12—figure supplement 1—source data 1. [file elife-80949-fig12-figsupp1-data1.zip › Figure 12-supplement 1 source data 1/GSK3b/p-GSK3b Ser9/DR pGSKb S9 WPP 2018.02.08_12.09.24_Ch-Marker.tif]

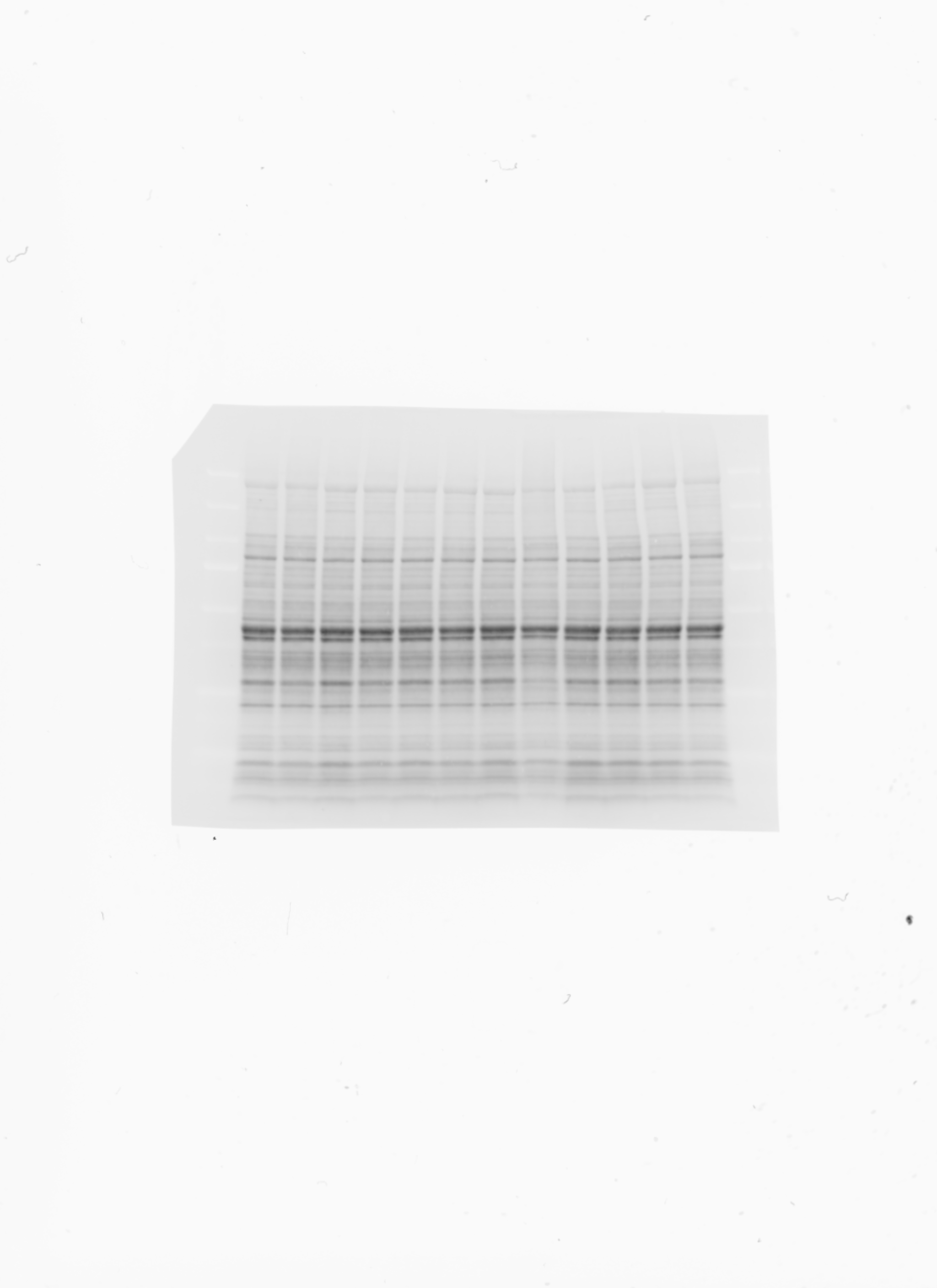

Supplement: Figure 12—figure supplement 1—source data 1. [file elife-80949-fig12-figsupp1-data1.zip › Figure 12-supplement 1 source data 1/GSK3b/Total Protein/DR TotProt LV Blot3 2018.01.29_13.19.08_Fl-UV.tif]

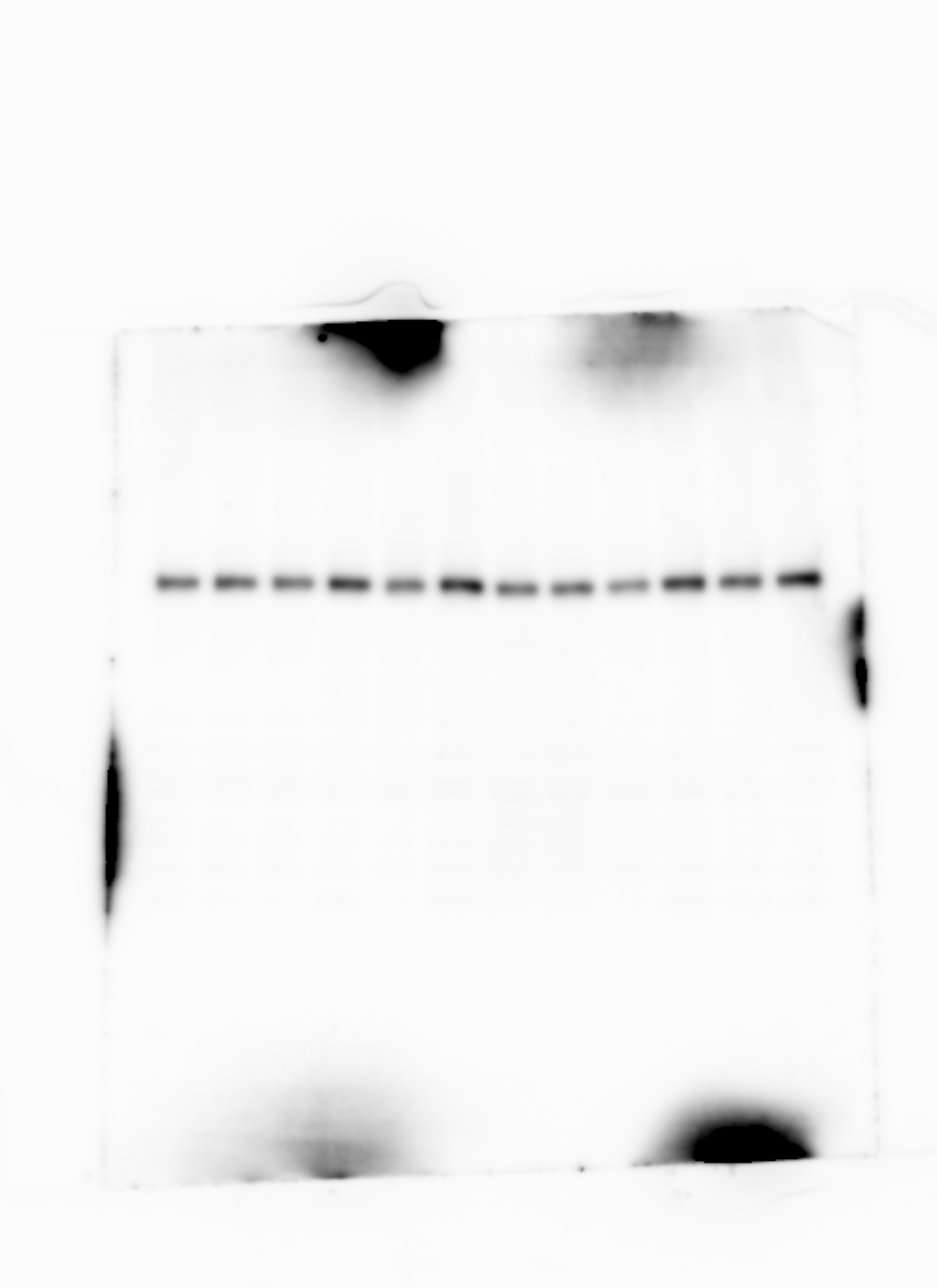

Supplement: Figure 12—figure supplement 1—source data 1. [file elife-80949-fig12-figsupp1-data1.zip › Figure 12-supplement 1 source data 1/HK1/HK1/DR HK1 Blot91 2021.02.25_12.55.56_Ch.tif]

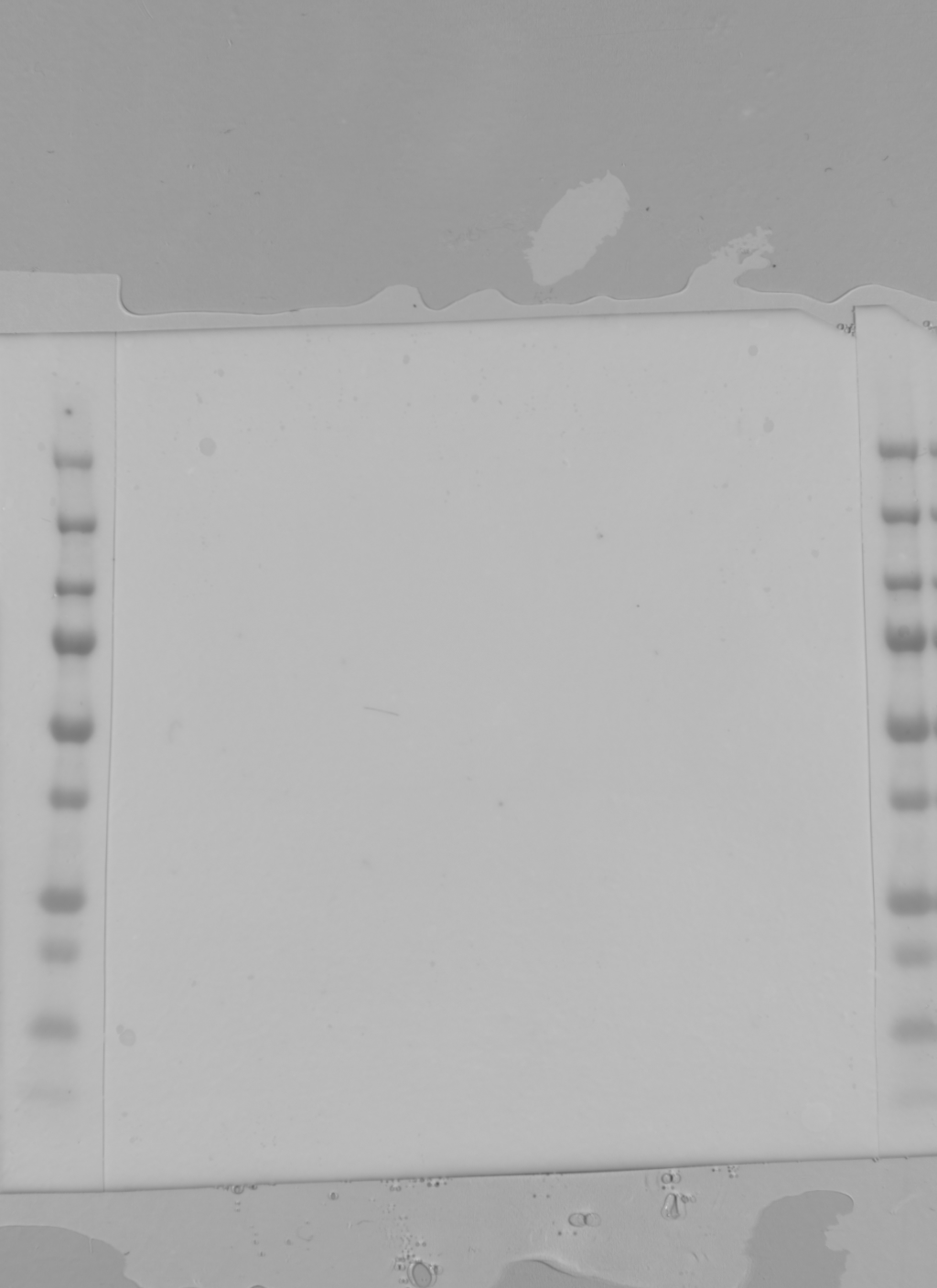

Supplement: Figure 12—figure supplement 1—source data 1. [file elife-80949-fig12-figsupp1-data1.zip › Figure 12-supplement 1 source data 1/HK1/HK1/DR HK1 Blot91 2021.02.25_12.55.56_Ch-Marker.tif]

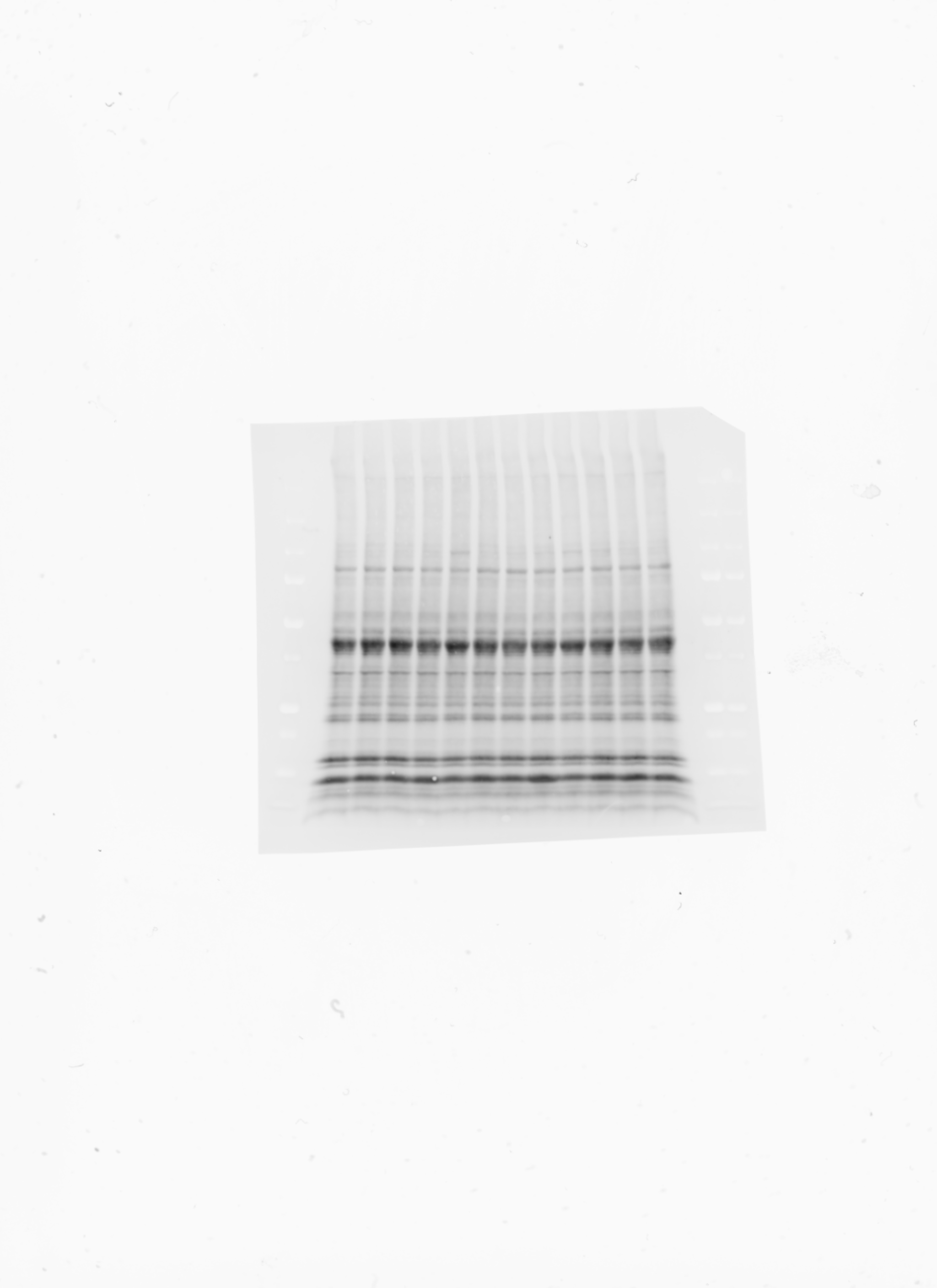

Supplement: Figure 12—figure supplement 1—source data 1. [file elife-80949-fig12-figsupp1-data1.zip › Figure 12-supplement 1 source data 1/HK1/Total Protein/DR T.Prot Blot91 2021.02.08_13.41.20_Fl-UV.tif]

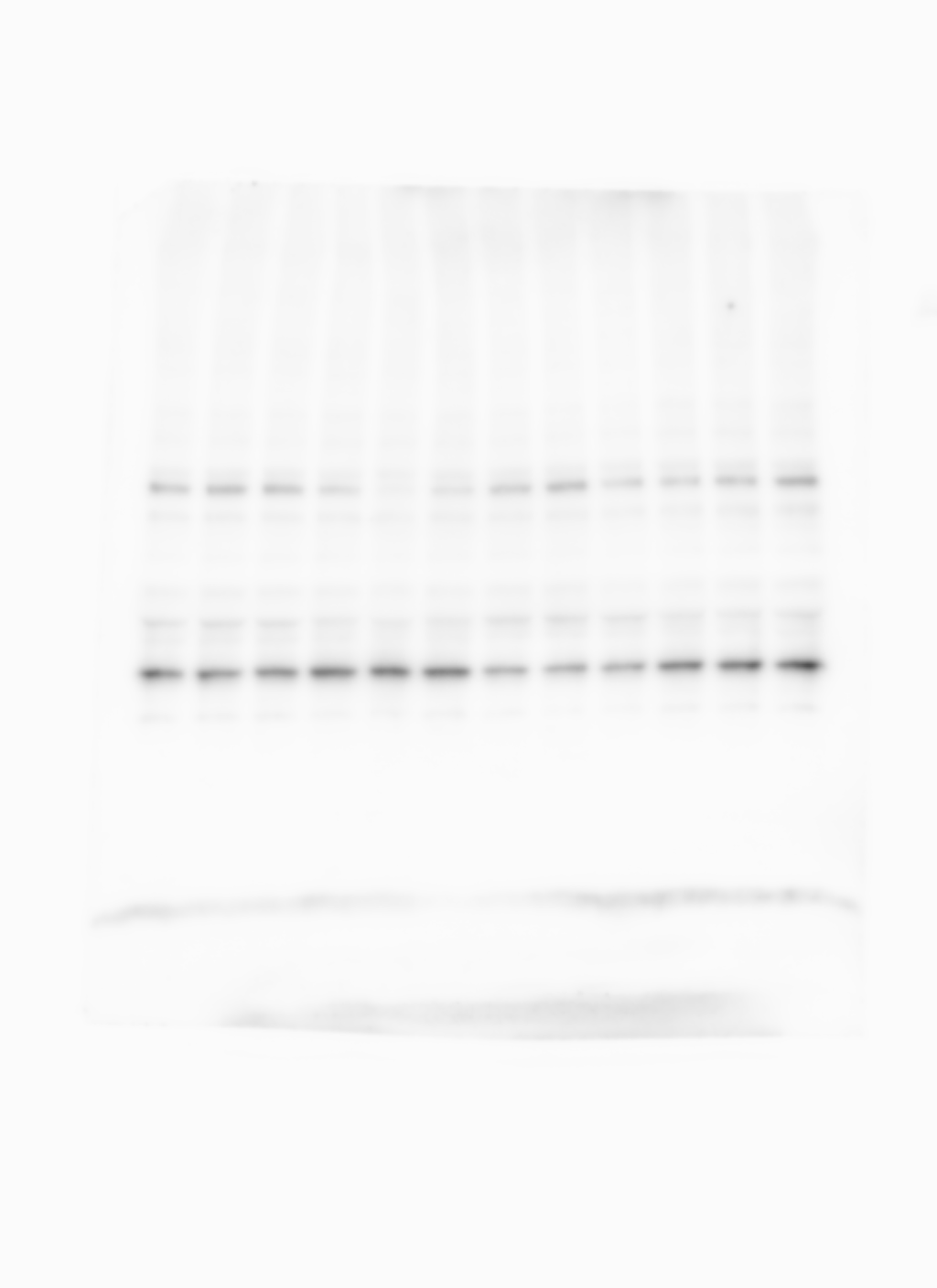

Supplement: Figure 12—figure supplement 1—source data 1. [file elife-80949-fig12-figsupp1-data1.zip › Figure 12-supplement 1 source data 1/LDHA/LDHA/DR LDHA Blot90 2021.02.25_12.32.25_Ch.tif]

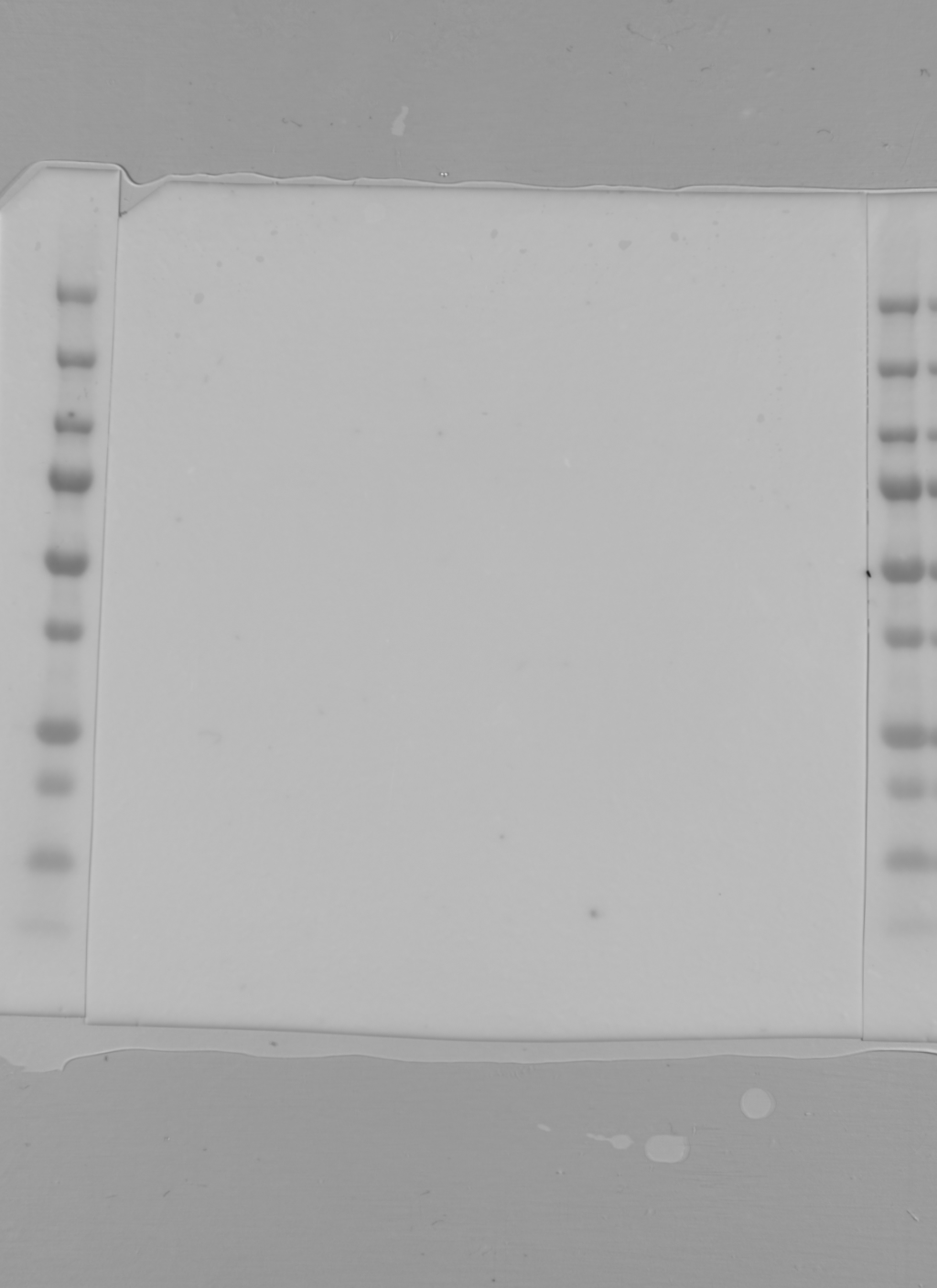

Supplement: Figure 12—figure supplement 1—source data 1. [file elife-80949-fig12-figsupp1-data1.zip › Figure 12-supplement 1 source data 1/LDHA/LDHA/DR LDHA Blot90 2021.02.25_12.32.25_Ch-Marker.tif]

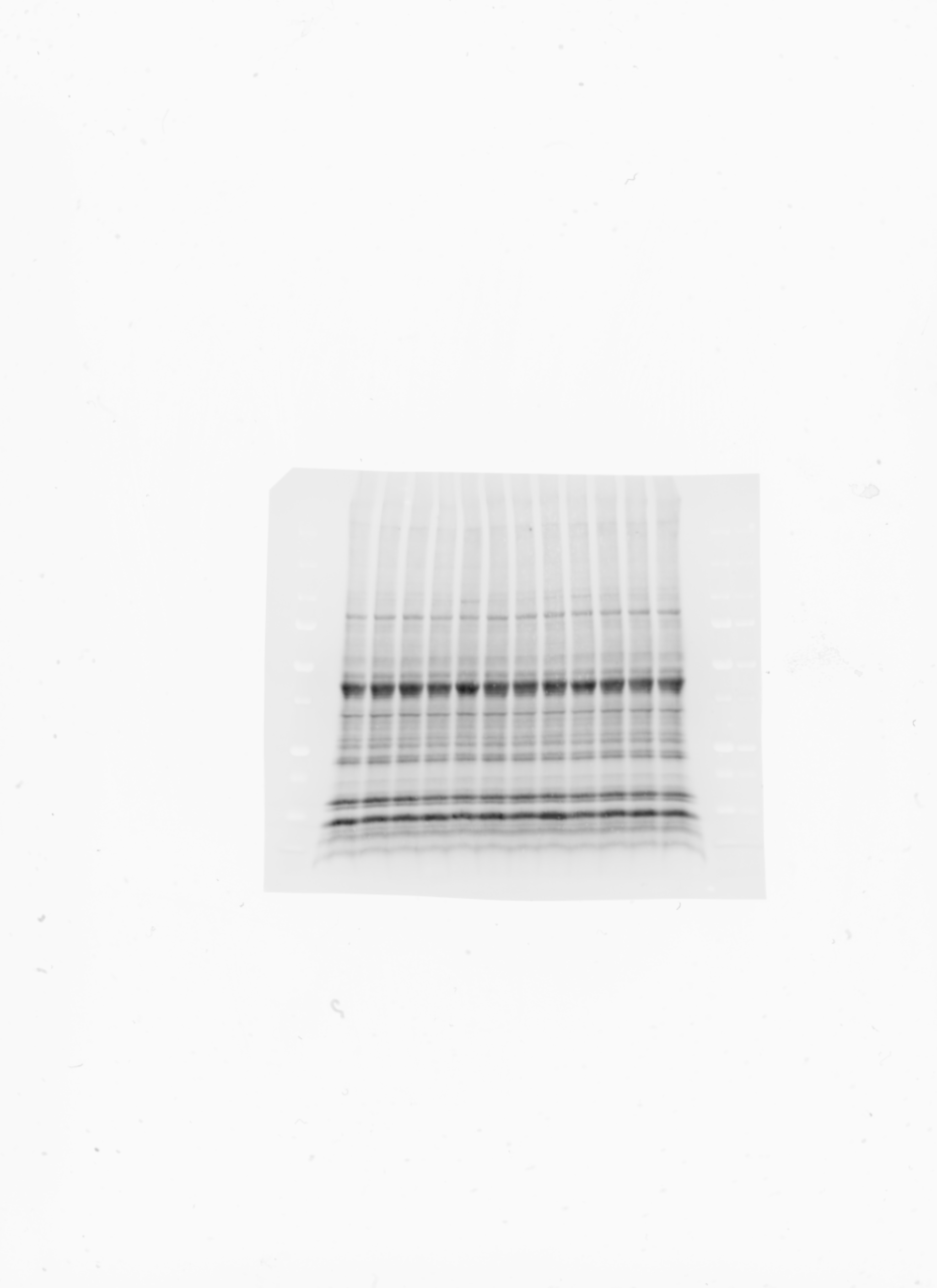

Supplement: Figure 12—figure supplement 1—source data 1. [file elife-80949-fig12-figsupp1-data1.zip › Figure 12-supplement 1 source data 1/LDHA/Total Protein/DR T.Prot Blot90 2021.02.08_13.36.43_Fl-UV.tif]

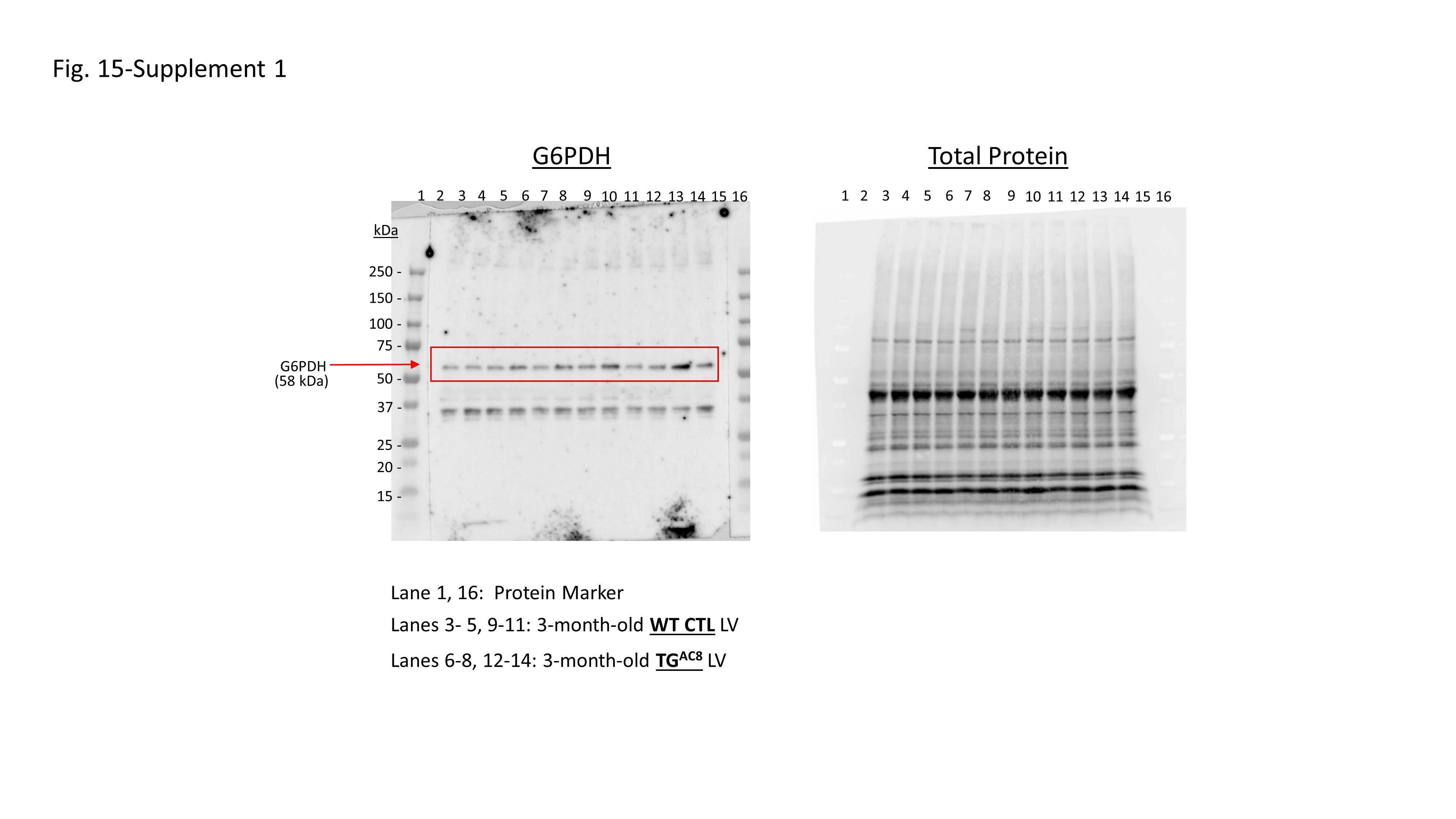

Supplement: Figure 12—figure supplement 1—source data 1. [file elife-80949-fig12-figsupp1-data1.zip › Figure 12-supplement 1 source data 1/Uncropped Images/G6PDH.JPG]

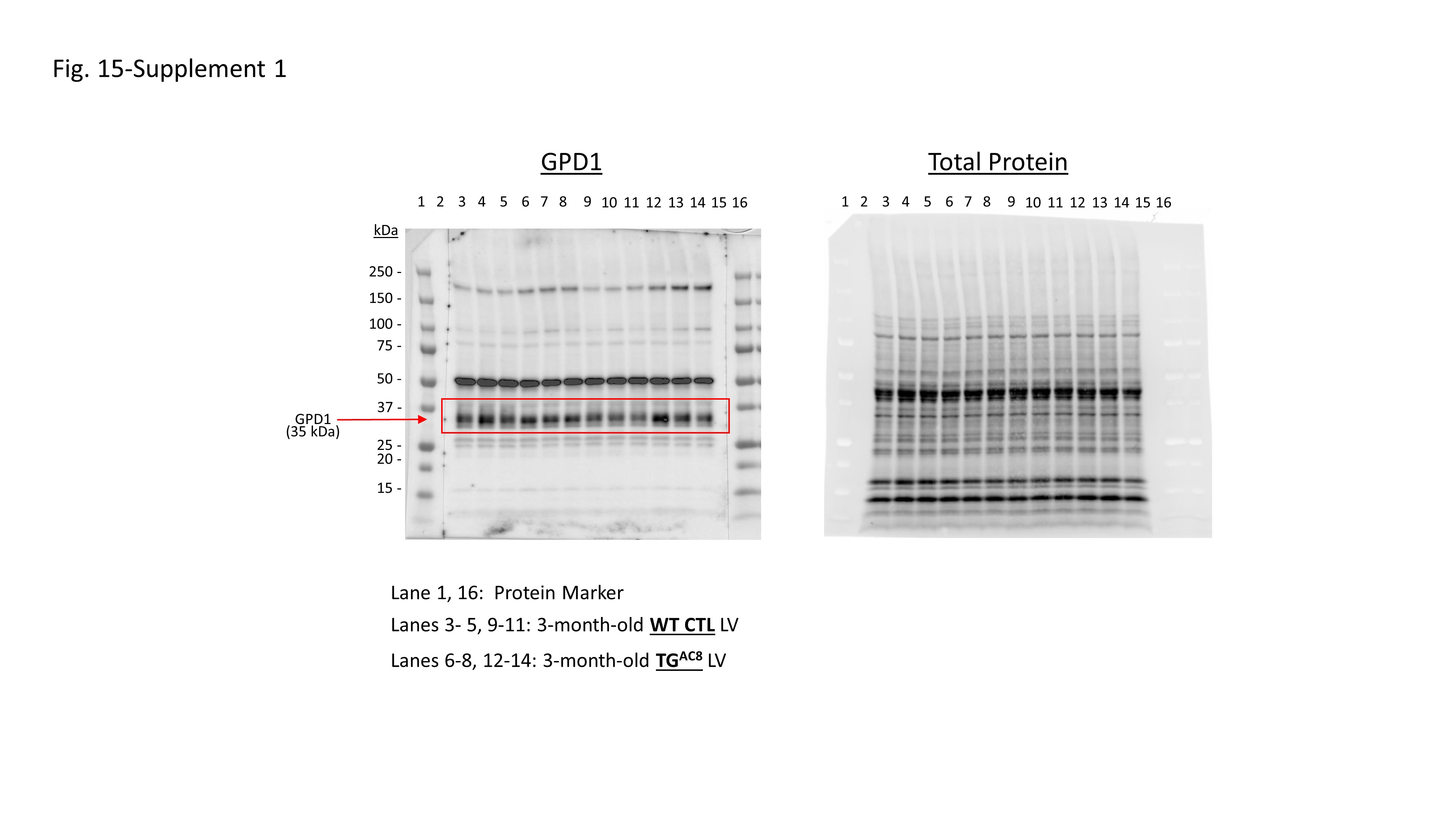

Supplement: Figure 12—figure supplement 1—source data 1. [file elife-80949-fig12-figsupp1-data1.zip › Figure 12-supplement 1 source data 1/Uncropped Images/GPD1.JPG]

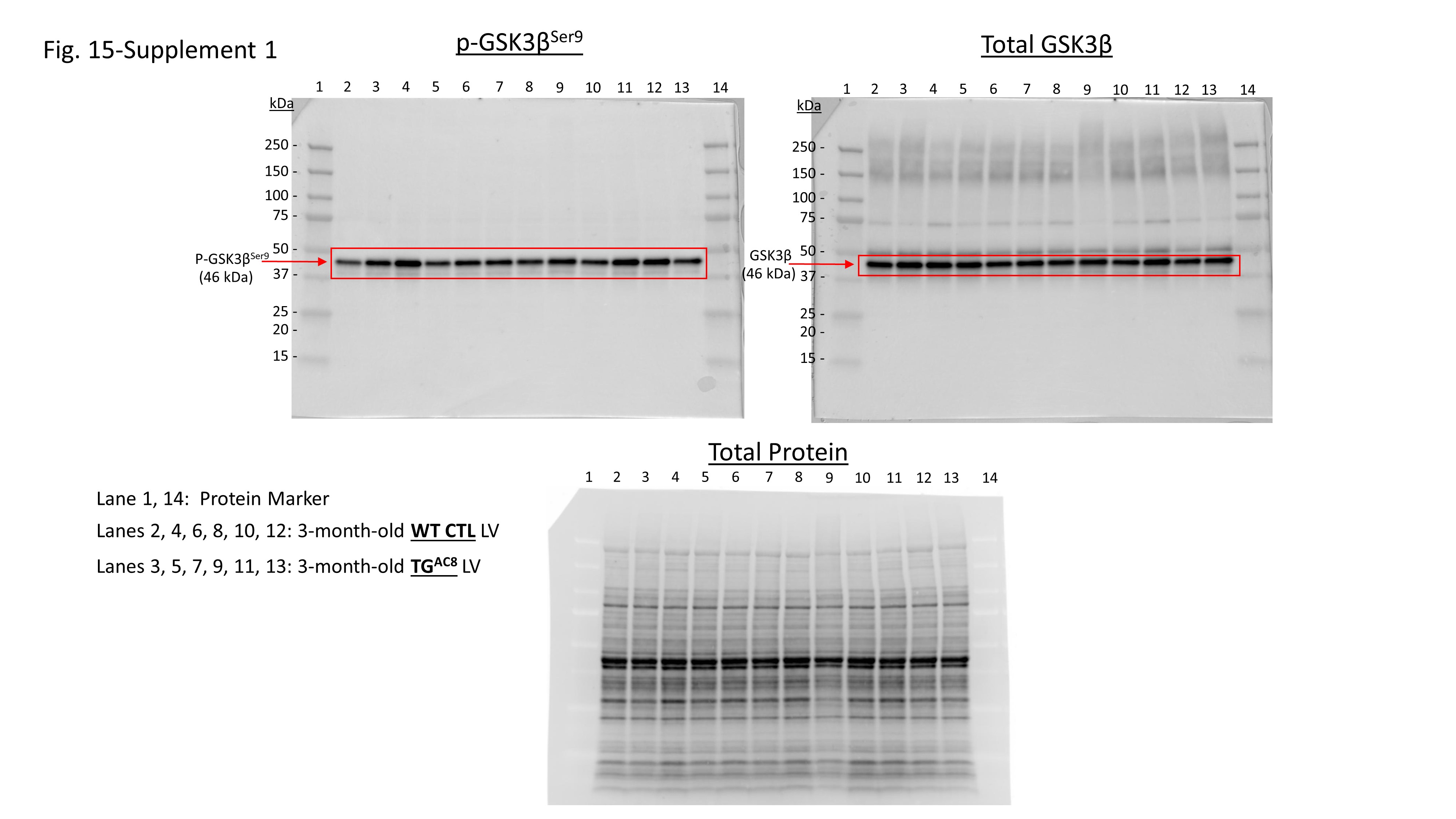

Supplement: Figure 12—figure supplement 1—source data 1. [file elife-80949-fig12-figsupp1-data1.zip › Figure 12-supplement 1 source data 1/Uncropped Images/GSK3.JPG]

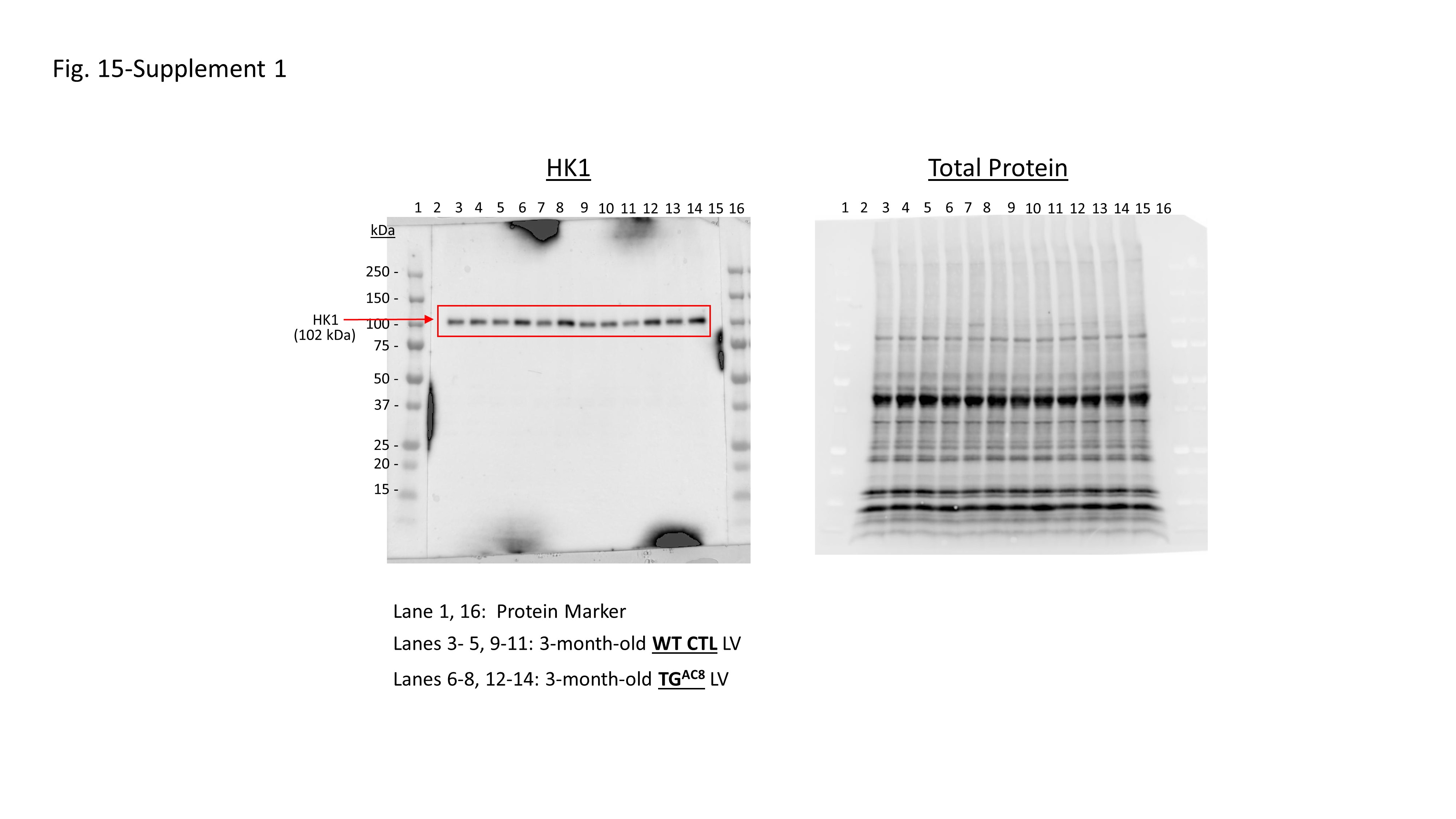

Supplement: Figure 12—figure supplement 1—source data 1. [file elife-80949-fig12-figsupp1-data1.zip › Figure 12-supplement 1 source data 1/Uncropped Images/HK1.JPG]

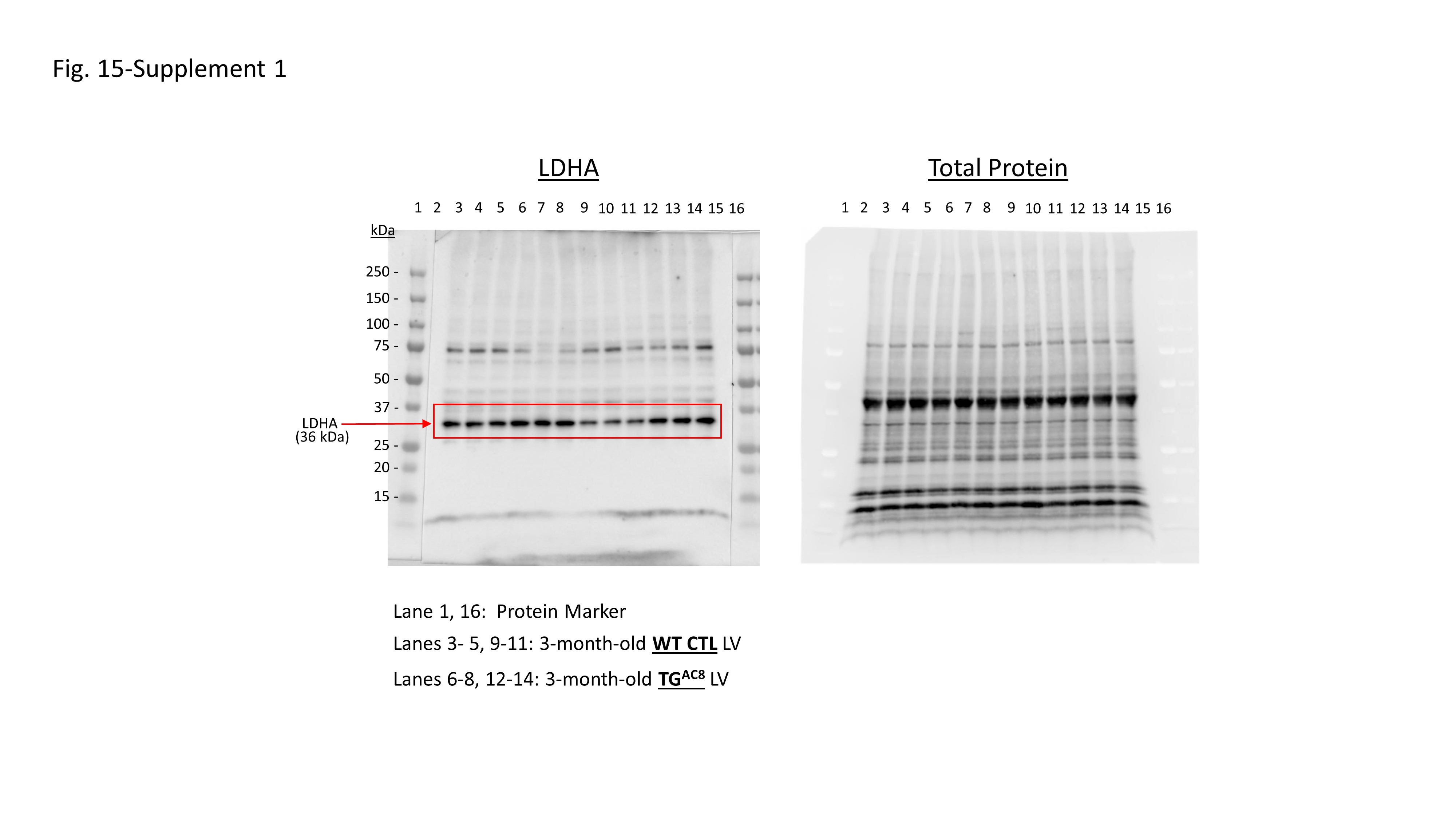

Supplement: Figure 12—figure supplement 1—source data 1. [file elife-80949-fig12-figsupp1-data1.zip › Figure 12-supplement 1 source data 1/Uncropped Images/LDHA.JPG]

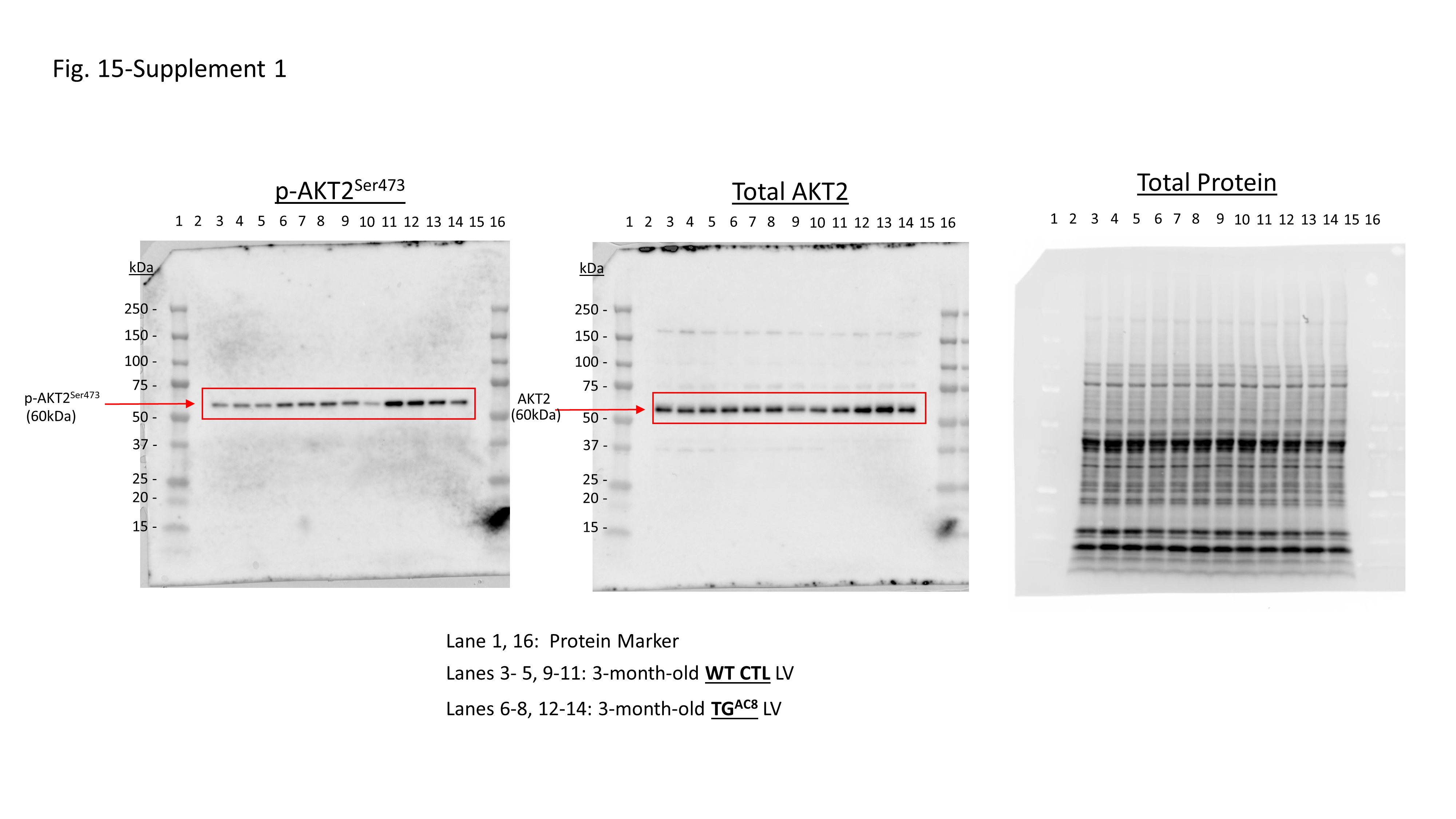

Supplement: Figure 12—figure supplement 1—source data 1. [file elife-80949-fig12-figsupp1-data1.zip › Figure 12-supplement 1 source data 1/Uncropped Images/p-AKT2Ser473.JPG]

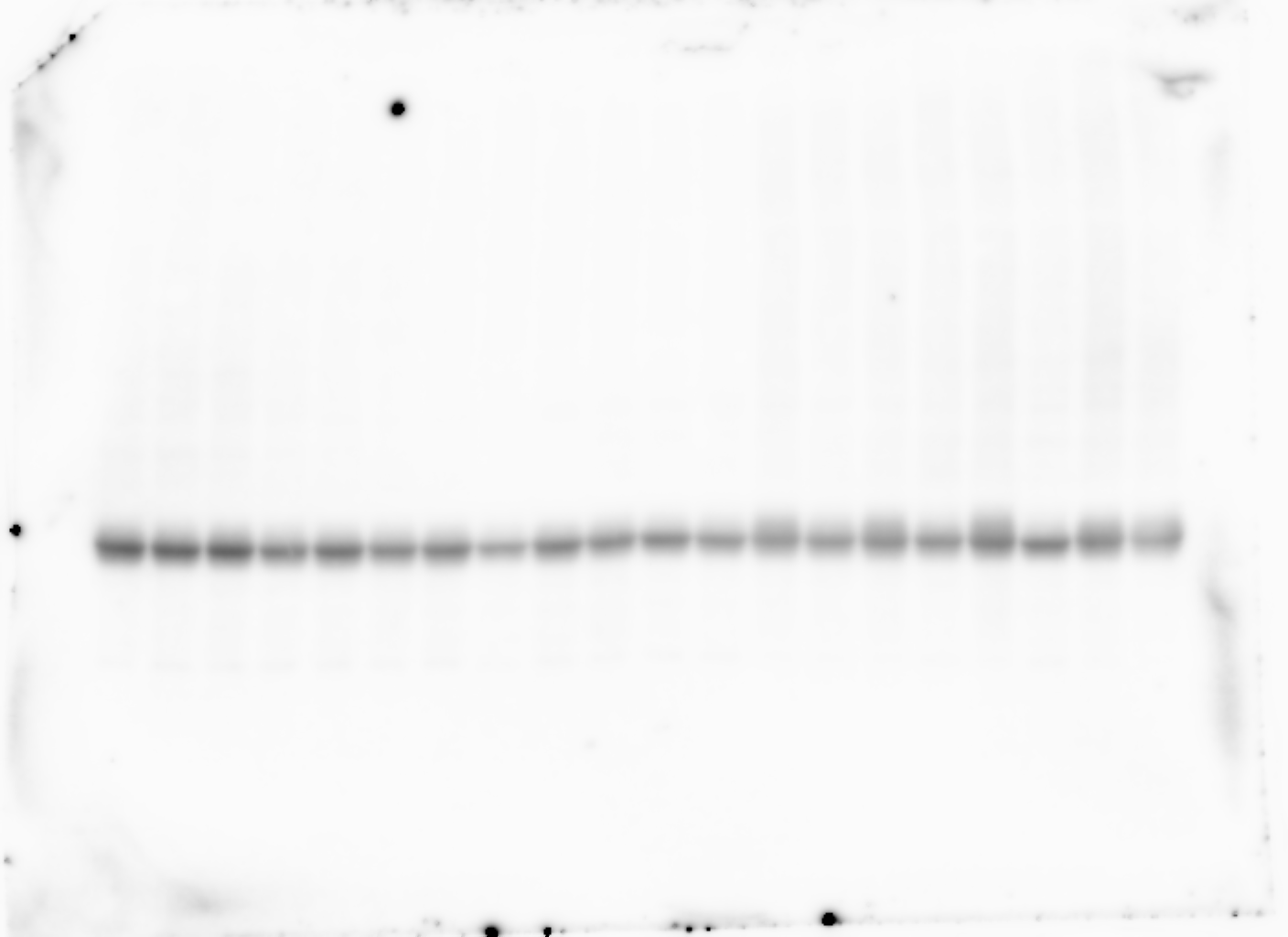

Supplement: Figure 12—figure supplement 1—source data 2. [file elife-80949-fig12-figsupp1-data2.zip › Figure 12-supplement 1 source data 2/PDHA1/PDHA1/DR PDHA1 blt40 2019.02.07_12.05.53_Ch.tif]

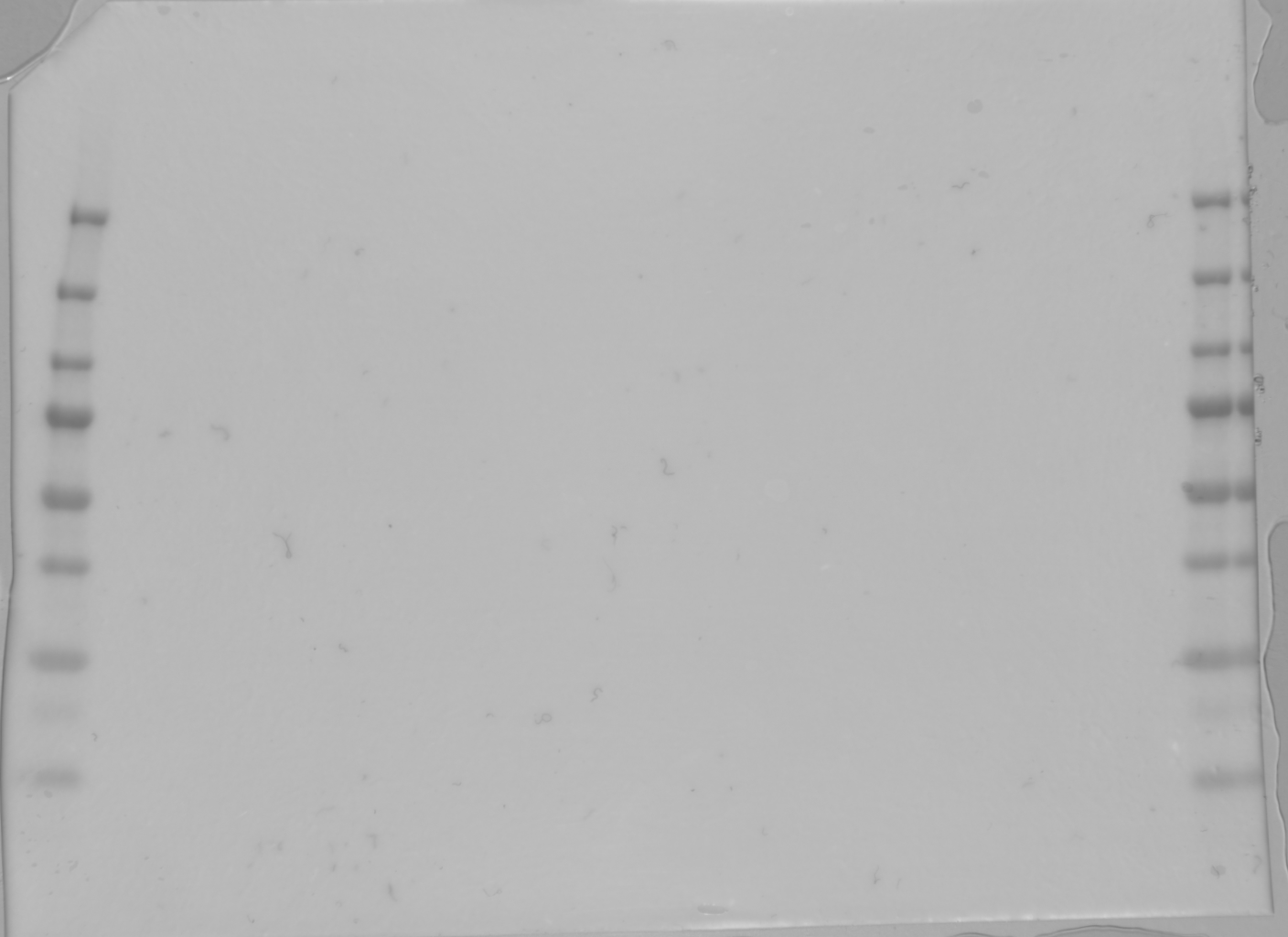

Supplement: Figure 12—figure supplement 1—source data 2. [file elife-80949-fig12-figsupp1-data2.zip › Figure 12-supplement 1 source data 2/PDHA1/PDHA1/DR PDHA1 blt40 2019.02.07_12.05.53_Ch-Marker.tif]

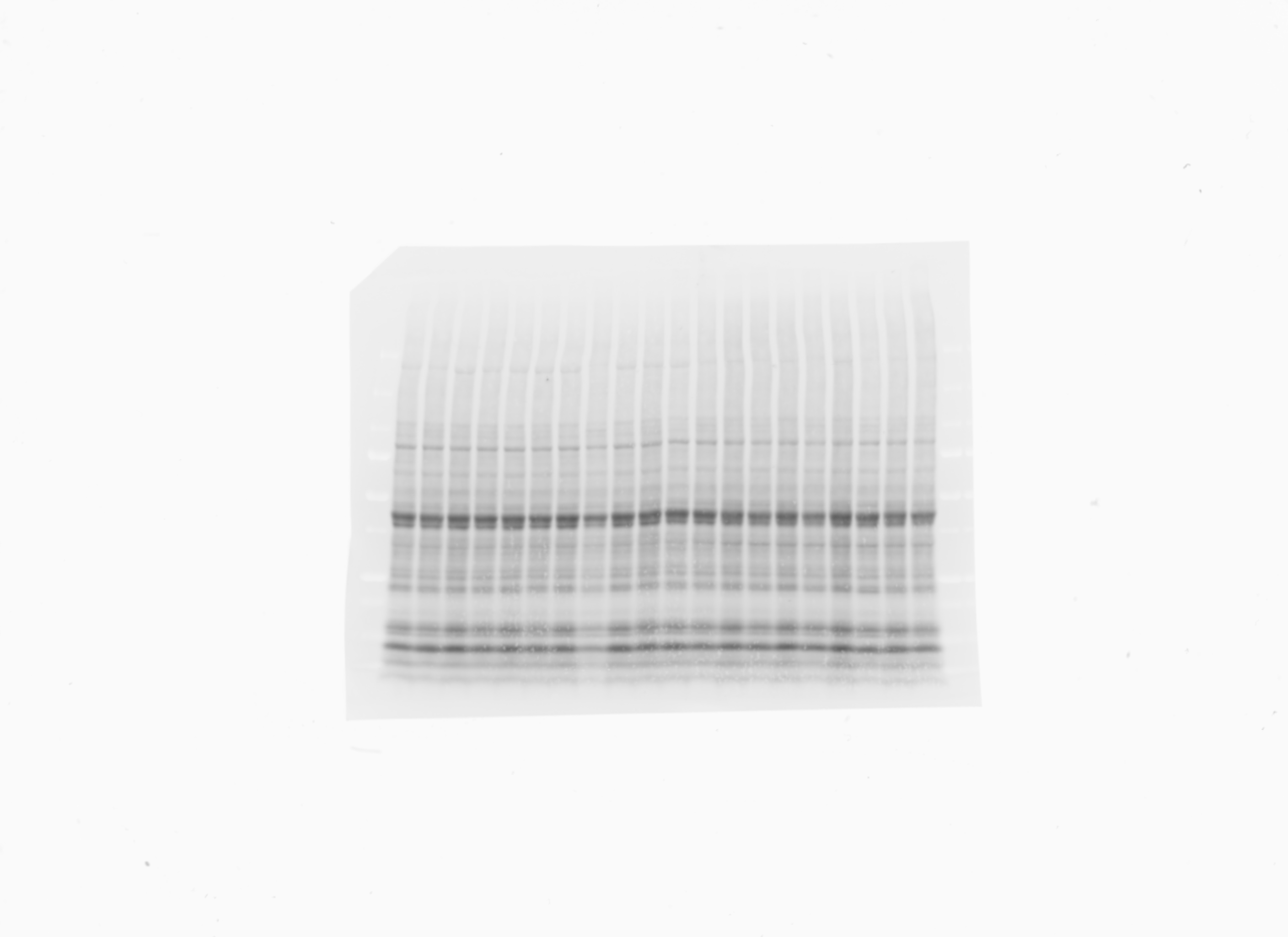

Supplement: Figure 12—figure supplement 1—source data 2. [file elife-80949-fig12-figsupp1-data2.zip › Figure 12-supplement 1 source data 2/PDHA1/Total Protein/DR T.Prot LV Blot40 2019.02.05_13.51.23_Fl-UV.tif]

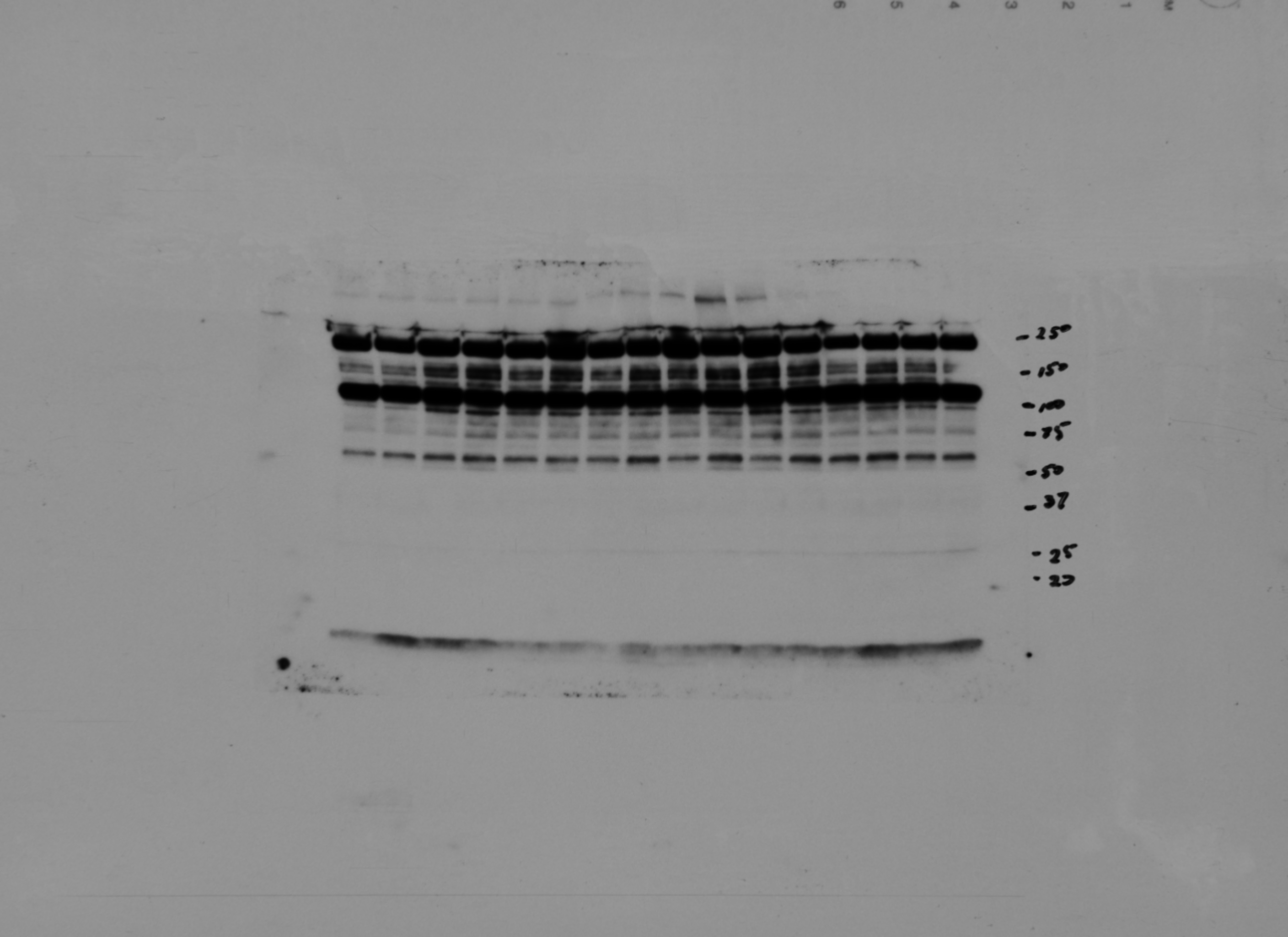

Supplement: Figure 12—figure supplement 1—source data 2. [file elife-80949-fig12-figsupp1-data2.zip › Figure 12-supplement 1 source data 2/PDK1/PDK1/PDK1 Blt22 Film 10m 2018.04.23_08.06.38_Co.tif]

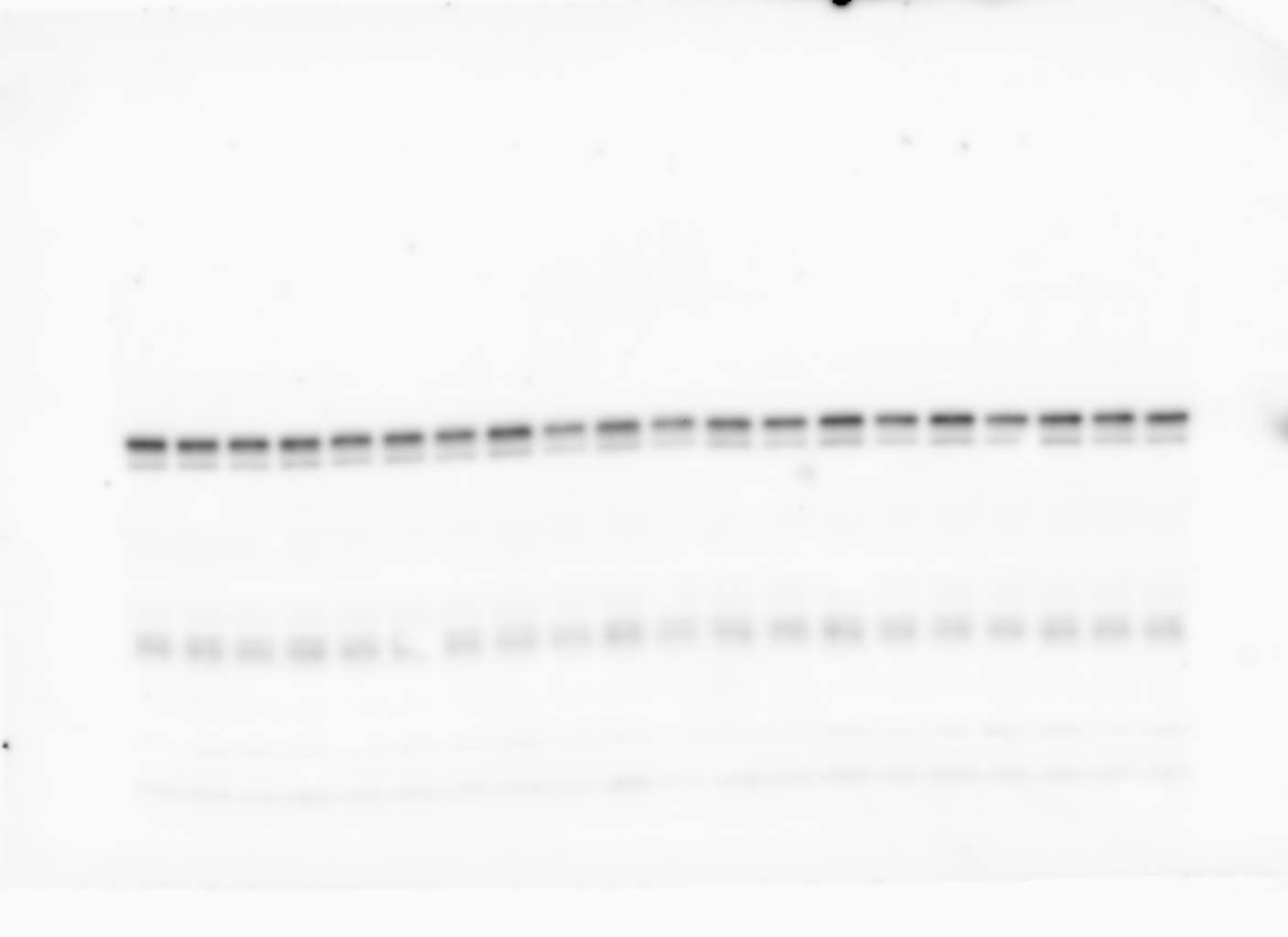

Supplement: Figure 12—figure supplement 1—source data 2. [file elife-80949-fig12-figsupp1-data2.zip › Figure 12-supplement 1 source data 2/PDK1/p-PDK1 Ser244/DR pPDK1 s241 WPP 2018.02.15_12.48.51_Ch.tif]

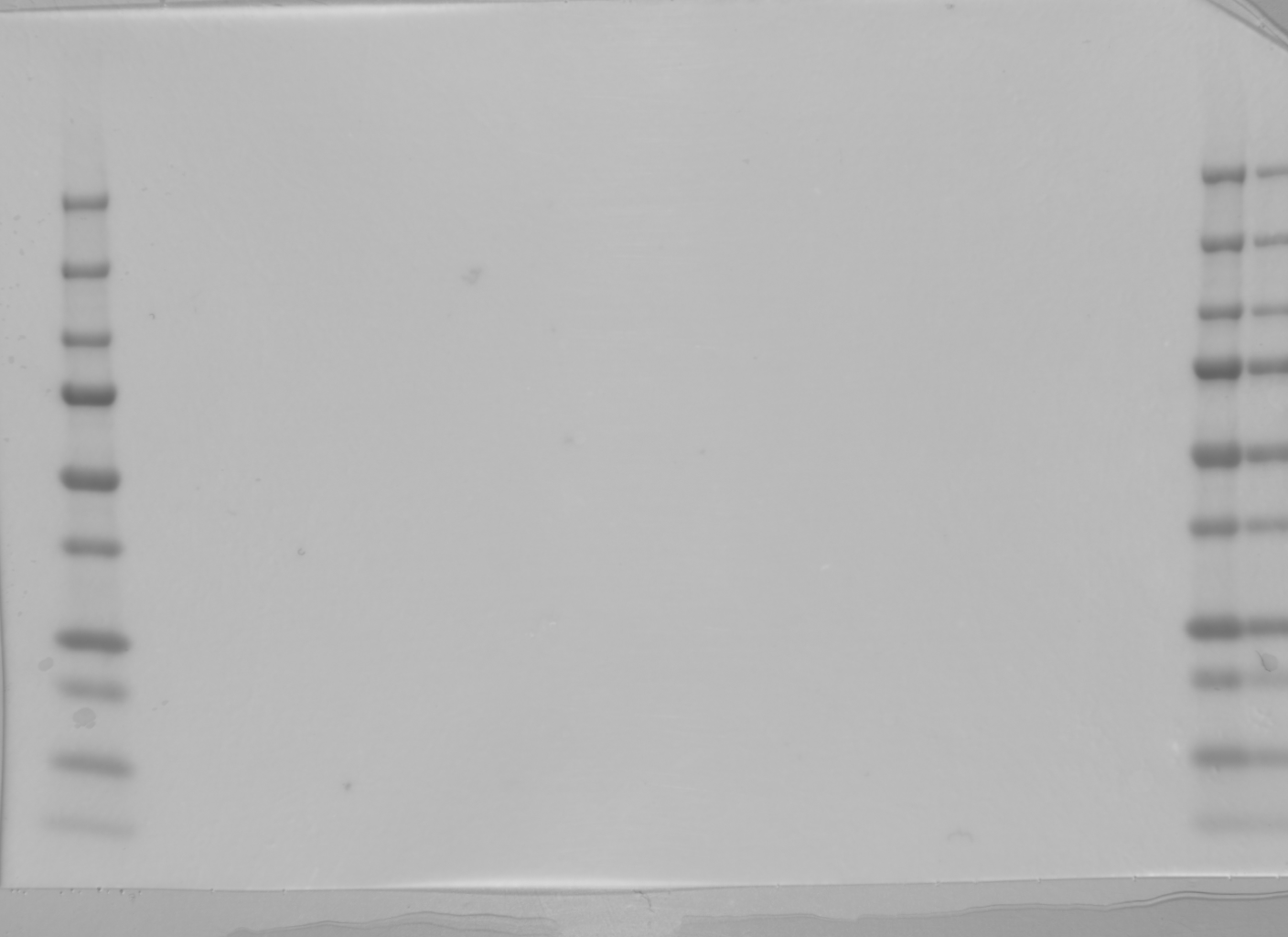

Supplement: Figure 12—figure supplement 1—source data 2. [file elife-80949-fig12-figsupp1-data2.zip › Figure 12-supplement 1 source data 2/PDK1/p-PDK1 Ser244/DR pPDK1 s241 WPP 2018.02.15_12.48.51_Ch-Marker.tif]

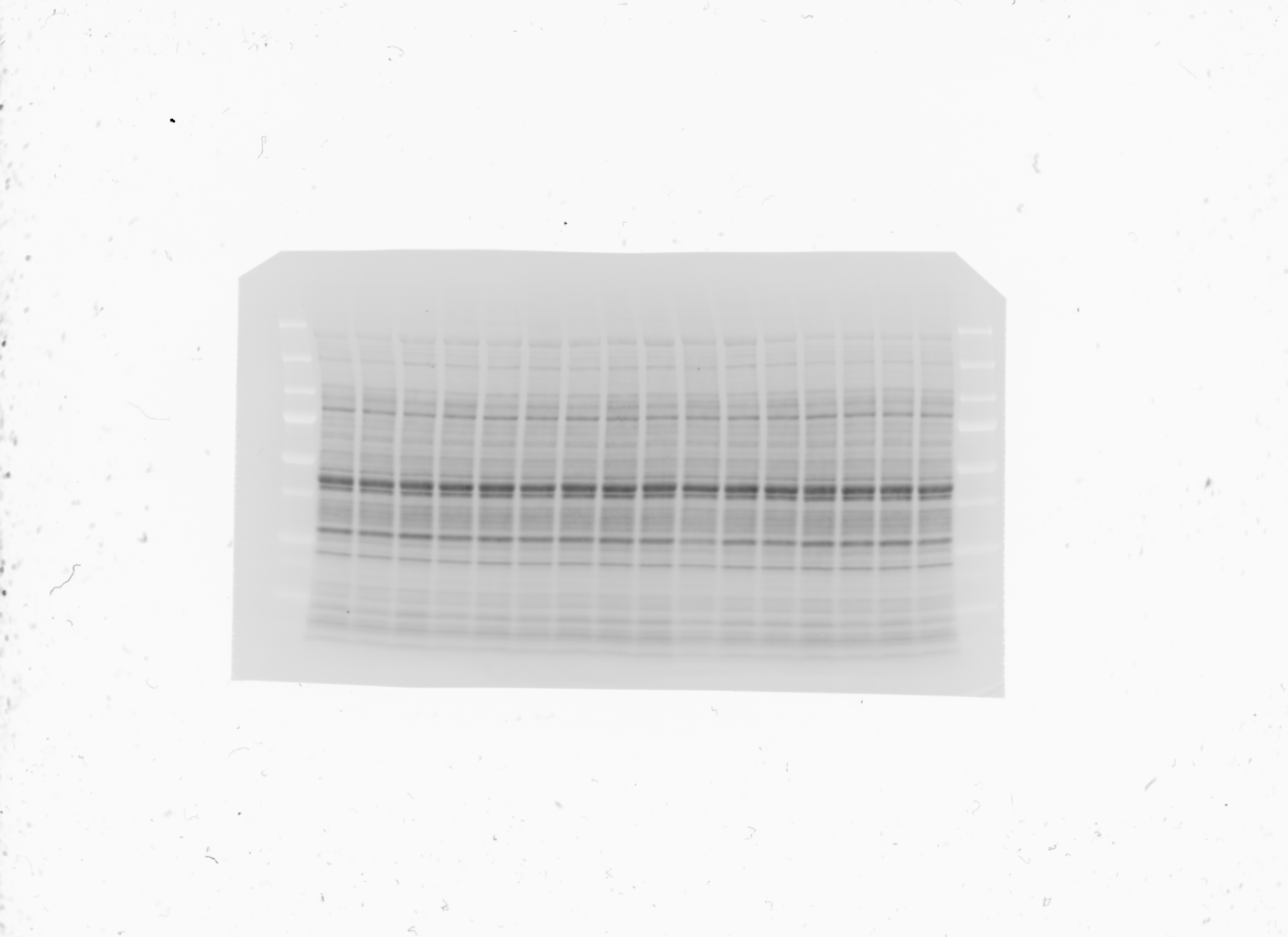

Supplement: Figure 12—figure supplement 1—source data 2. [file elife-80949-fig12-figsupp1-data2.zip › Figure 12-supplement 1 source data 2/PDK1/Total Protein/DR T.Prot. LV Blt 22 2018.04.19_14.07.49_Fl-UV.tif]

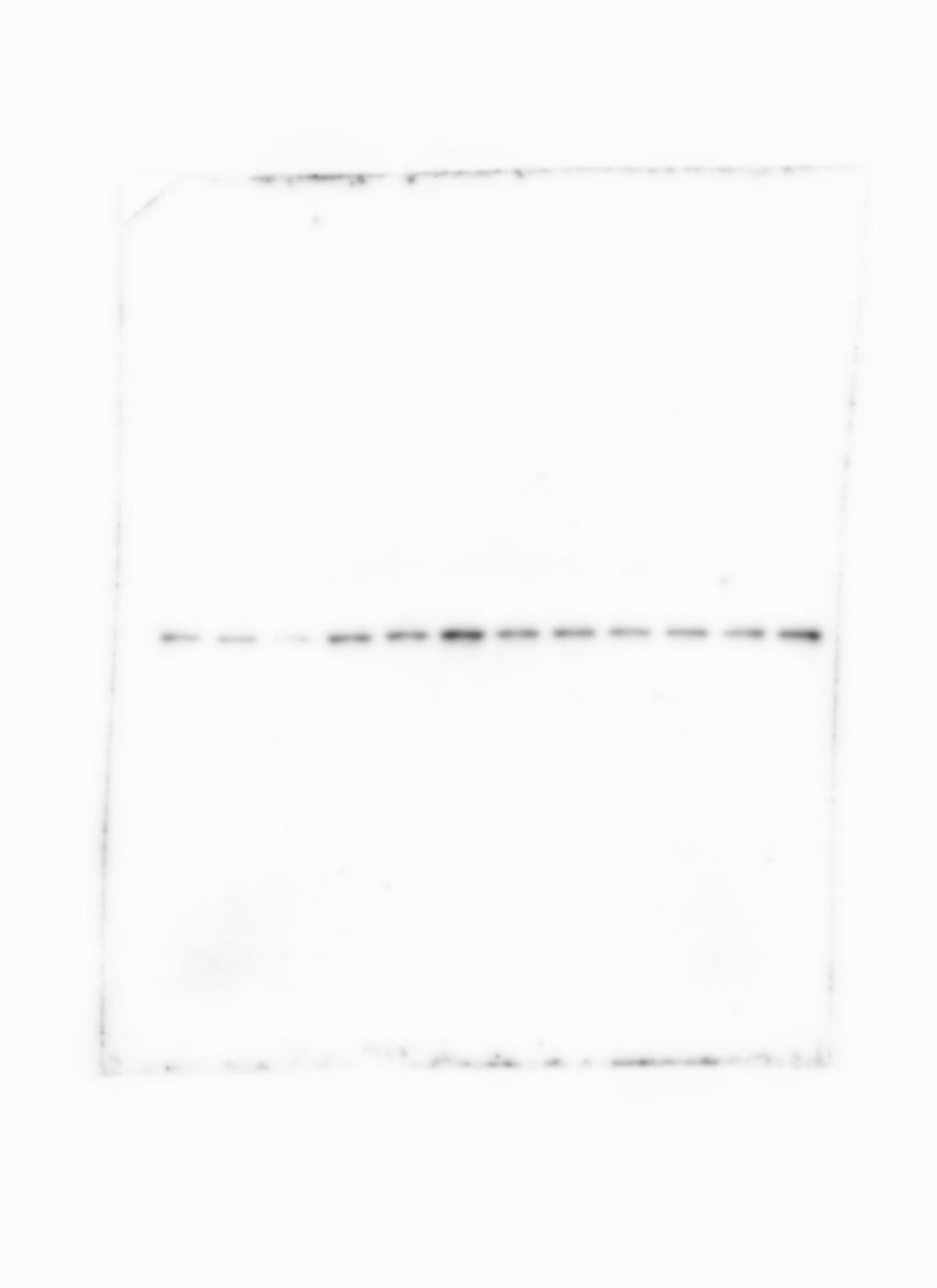

Supplement: Figure 12—figure supplement 1—source data 2. [file elife-80949-fig12-figsupp1-data2.zip › Figure 12-supplement 1 source data 2/PGD/PGD/DR PGD LV Blt75 2020.11.24_11.40.37_Ch.tif]

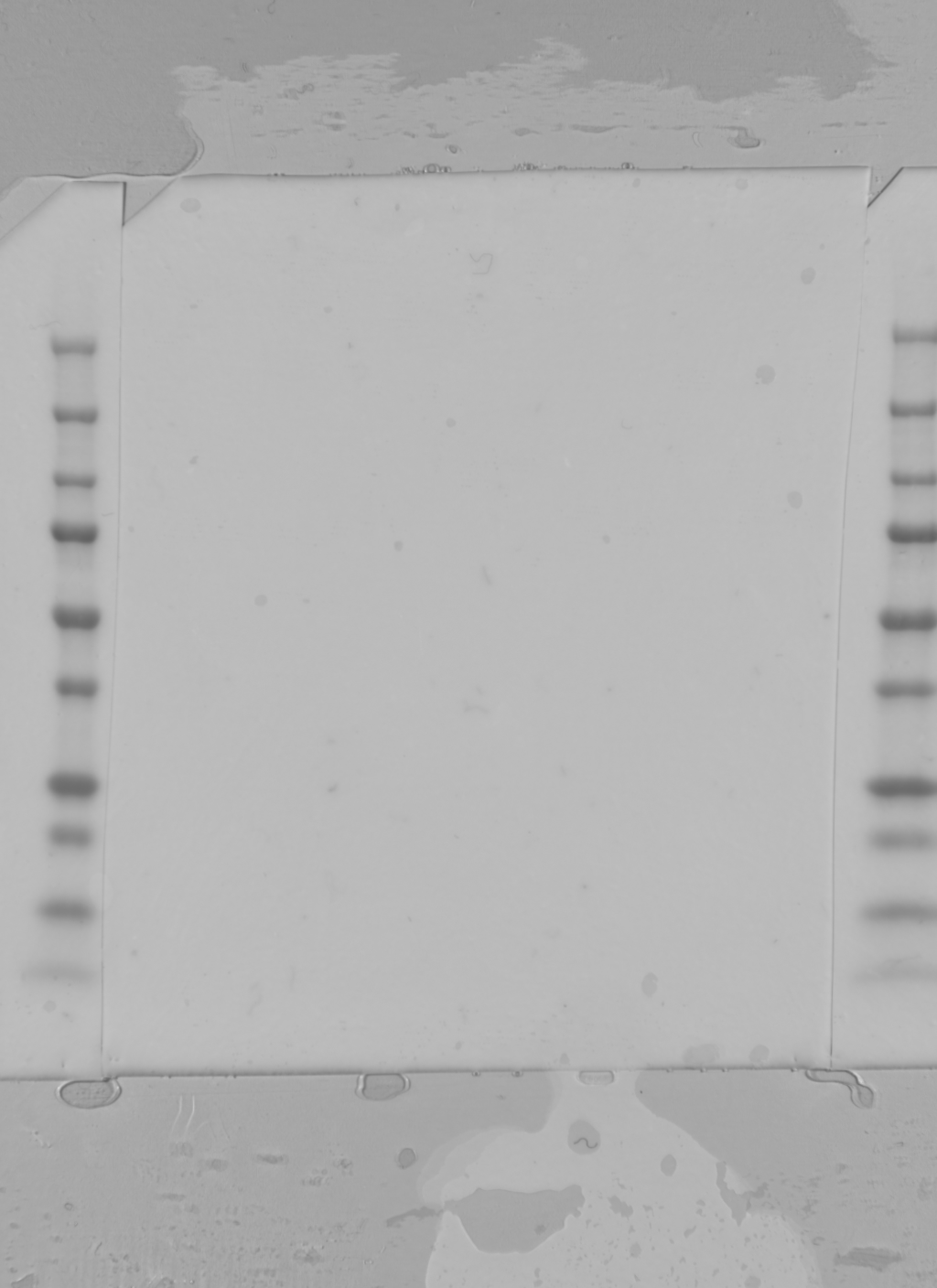

Supplement: Figure 12—figure supplement 1—source data 2. [file elife-80949-fig12-figsupp1-data2.zip › Figure 12-supplement 1 source data 2/PGD/PGD/DR PGD LV Blt75 2020.11.24_11.40.37_Ch-Marker.tif]

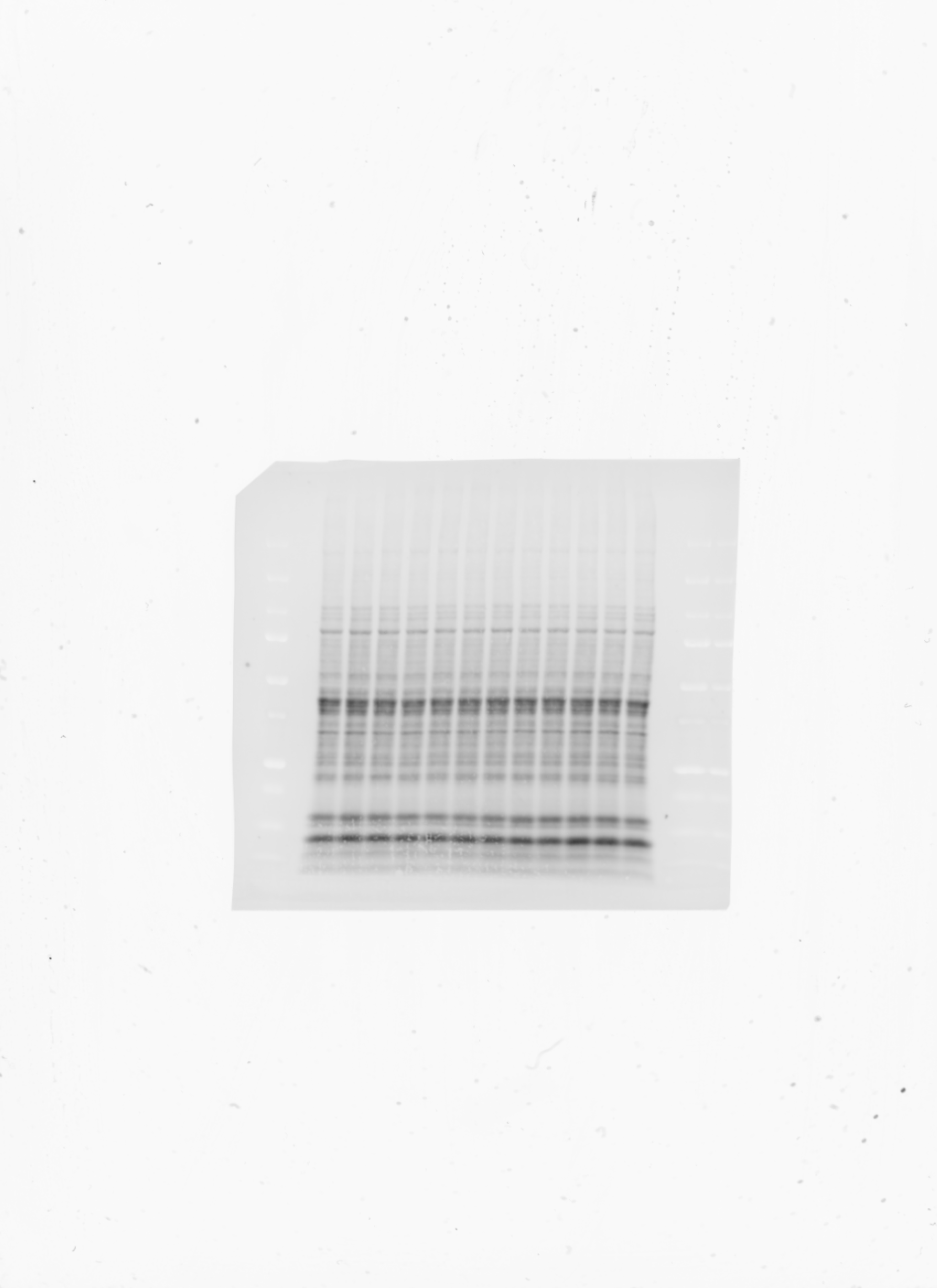

Supplement: Figure 12—figure supplement 1—source data 2. [file elife-80949-fig12-figsupp1-data2.zip › Figure 12-supplement 1 source data 2/PGD/Total Protein/DR TProt. Blot75 2020.09.21_12.10.55_Fl-UV.tif]

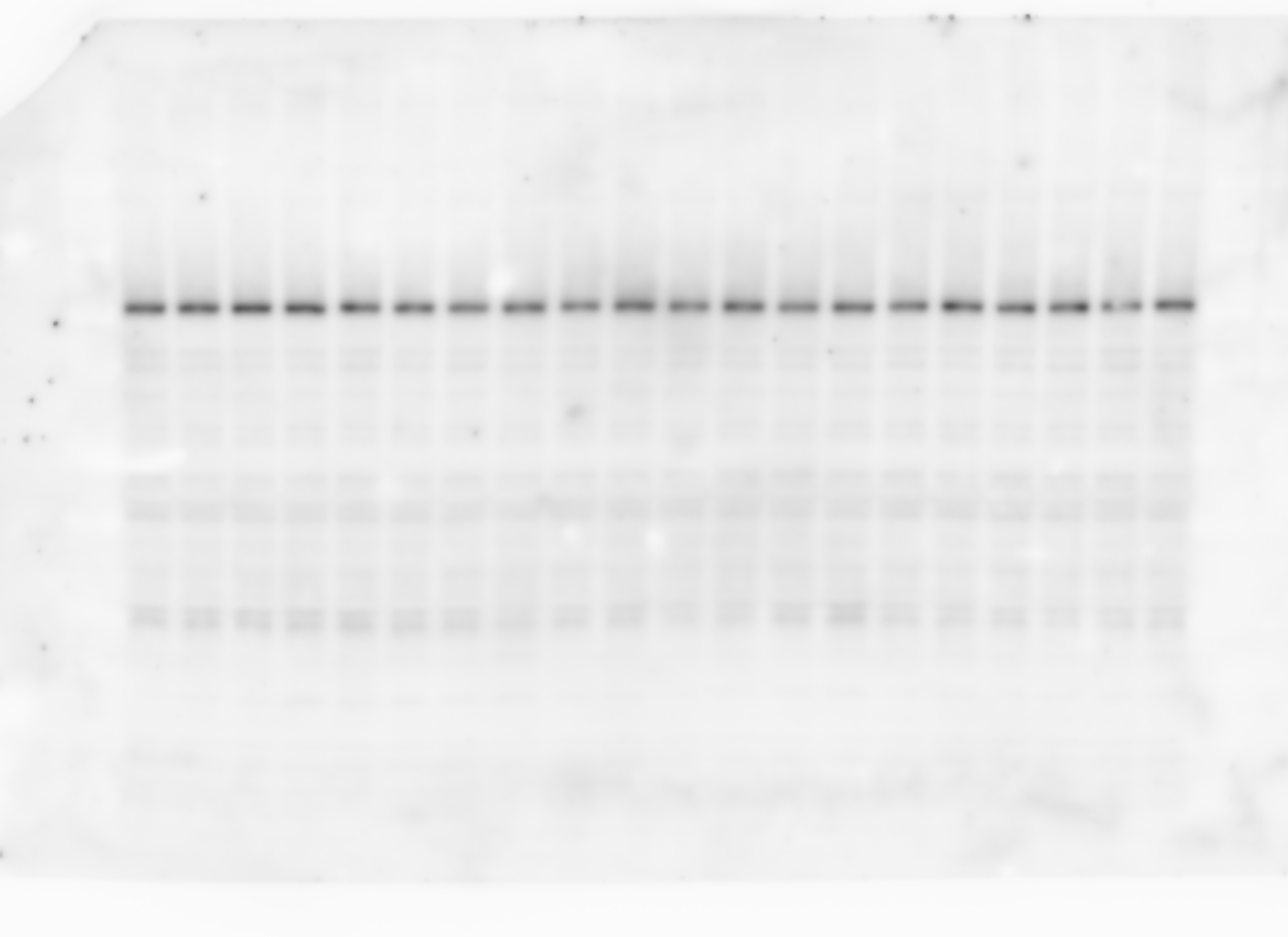

Supplement: Figure 12—figure supplement 1—source data 2. [file elife-80949-fig12-figsupp1-data2.zip › Figure 12-supplement 1 source data 2/PIK3CA/PIK3CA/DR PI3K p110a WPP 2018.02.16_12.16.27_Ch.tif]

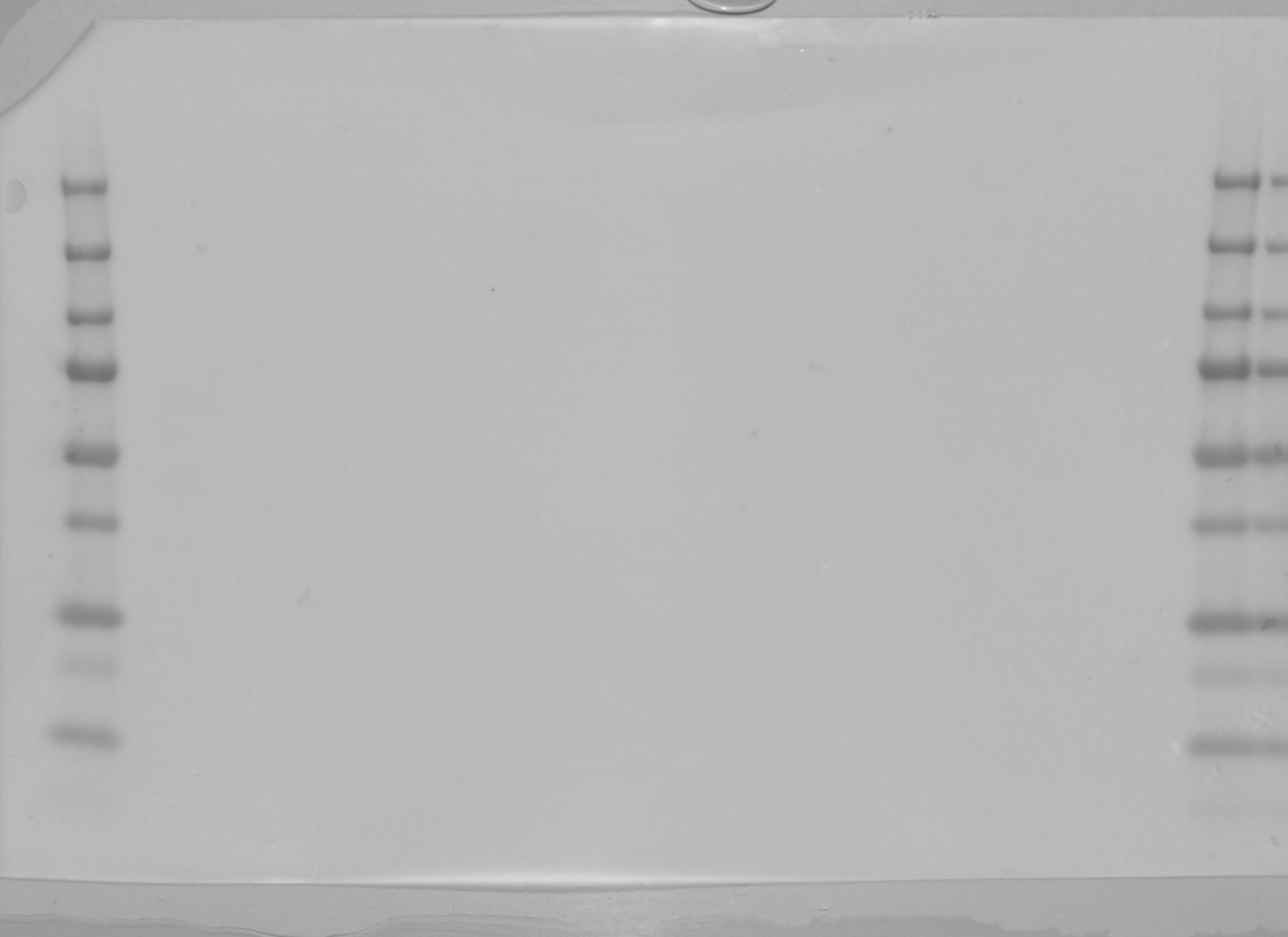

Supplement: Figure 12—figure supplement 1—source data 2. [file elife-80949-fig12-figsupp1-data2.zip › Figure 12-supplement 1 source data 2/PIK3CA/PIK3CA/DR PI3K p110a WPP 2018.02.16_12.16.27_Ch-Marker.tif]

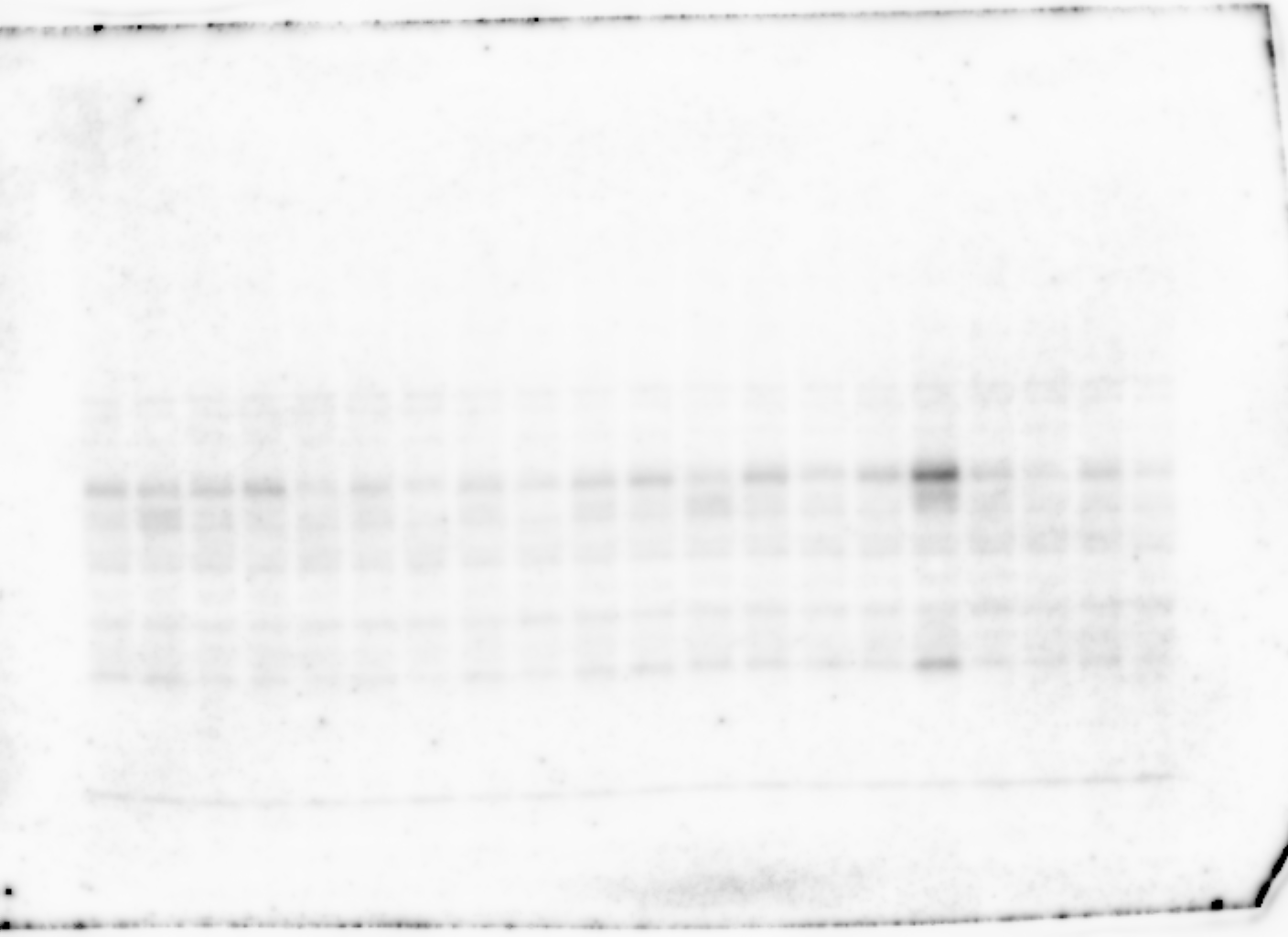

Supplement: Figure 12—figure supplement 1—source data 2. [file elife-80949-fig12-figsupp1-data2.zip › Figure 12-supplement 1 source data 2/PTEN/p-PTEN Ser380/DR pPTEN S380 WPP 2018.02.15_14.07.28_Ch.tif]

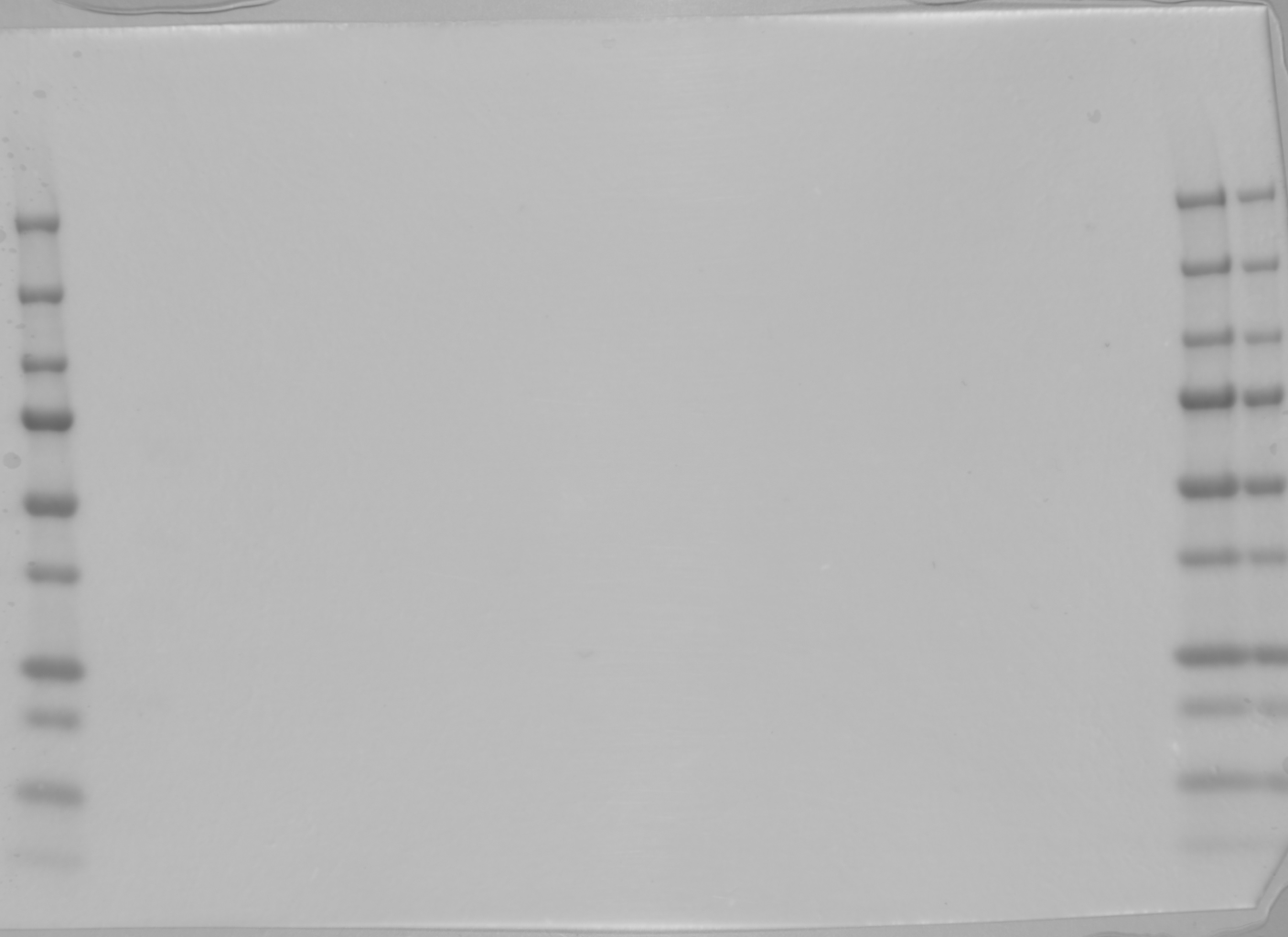

Supplement: Figure 12—figure supplement 1—source data 2. [file elife-80949-fig12-figsupp1-data2.zip › Figure 12-supplement 1 source data 2/PTEN/p-PTEN Ser380/DR pPTEN S380 WPP 2018.02.15_14.07.28_Ch-Marker.tif]

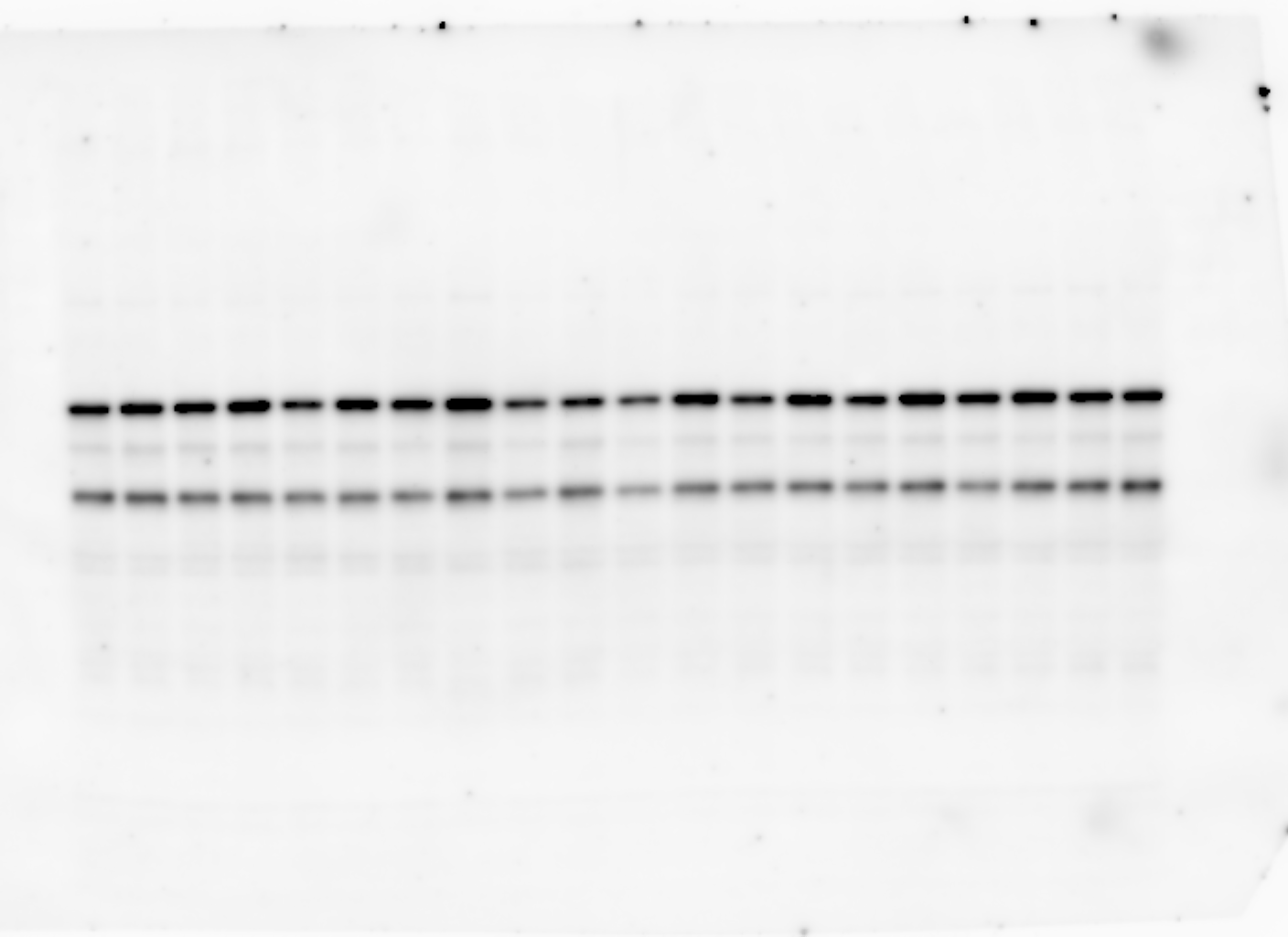

Supplement: Figure 12—figure supplement 1—source data 2. [file elife-80949-fig12-figsupp1-data2.zip › Figure 12-supplement 1 source data 2/PTEN/PTEN/DR PTEN WPP 2018.02.16_13.35.04_Ch.tif]

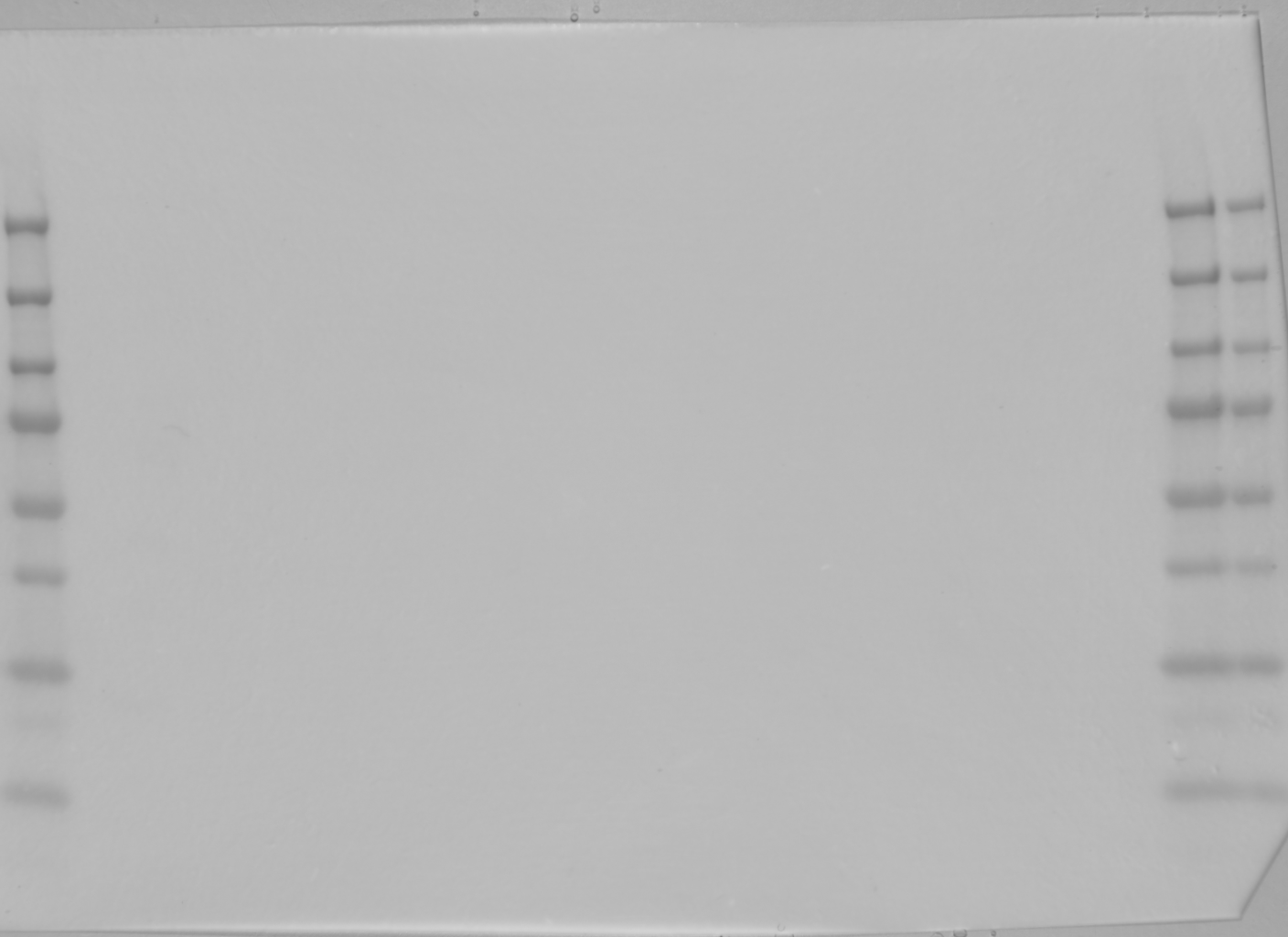

Supplement: Figure 12—figure supplement 1—source data 2. [file elife-80949-fig12-figsupp1-data2.zip › Figure 12-supplement 1 source data 2/PTEN/PTEN/DR PTEN WPP 2018.02.16_13.35.04_Ch-Marker.tif]

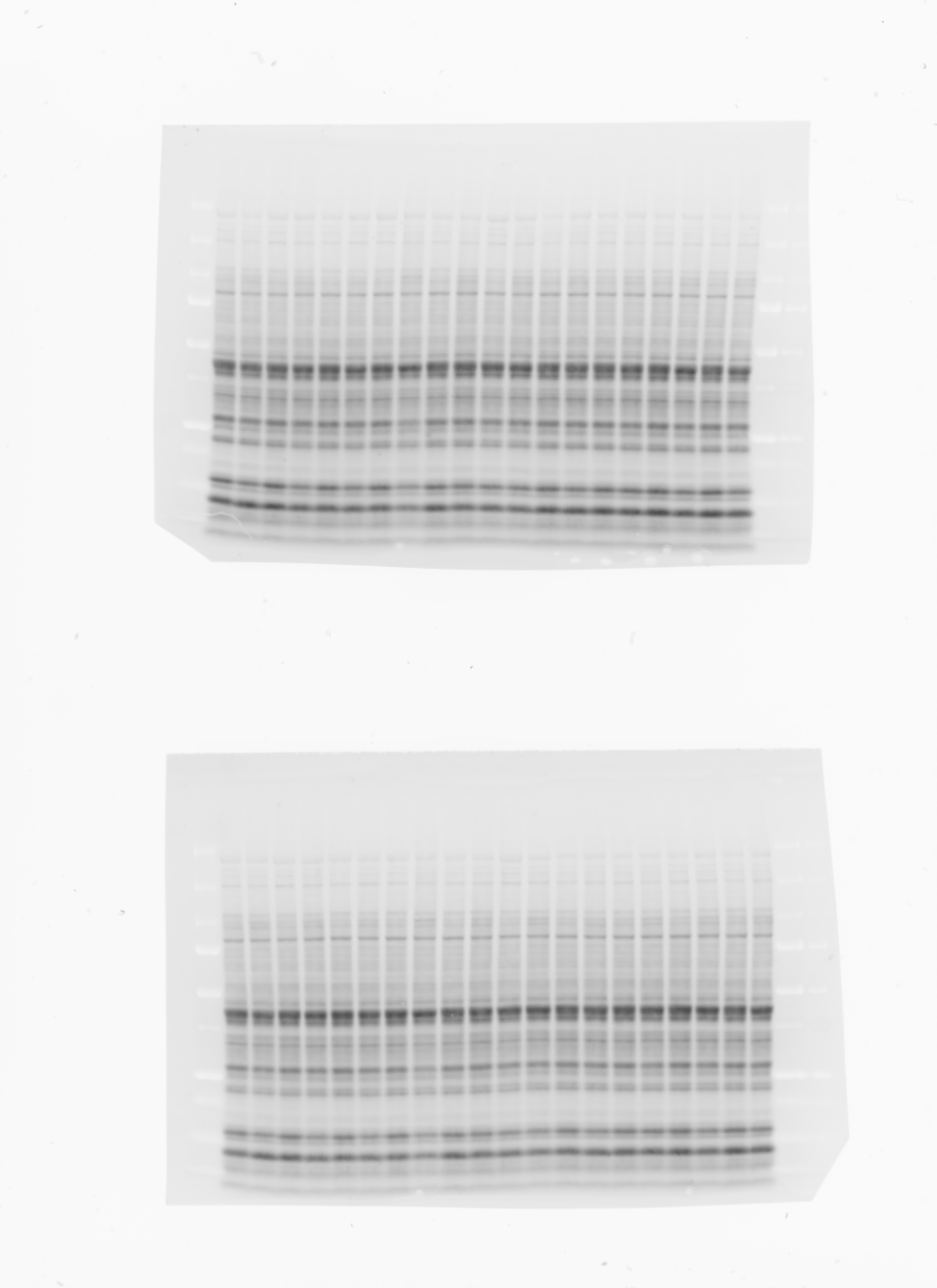

Supplement: Figure 12—figure supplement 1—source data 2. [file elife-80949-fig12-figsupp1-data2.zip › Figure 12-supplement 1 source data 2/PTEN/Total Protein/DR Tot.Prot. Blt 7,8 2018.02.14_13.43.03_Fl-UV.tif]

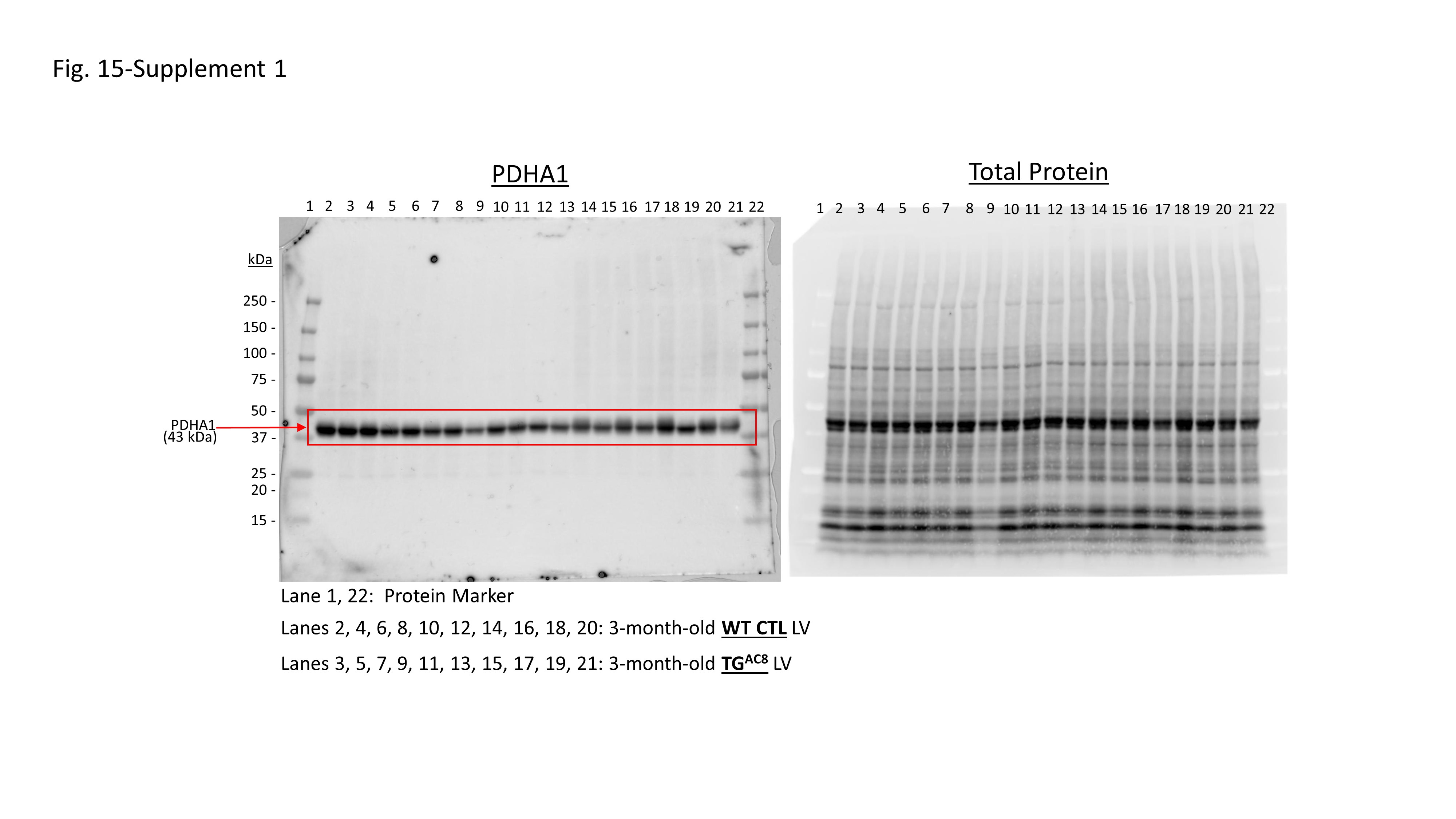

Supplement: Figure 12—figure supplement 1—source data 2. [file elife-80949-fig12-figsupp1-data2.zip › Figure 12-supplement 1 source data 2/Uncropped Images/PDHA1.JPG]

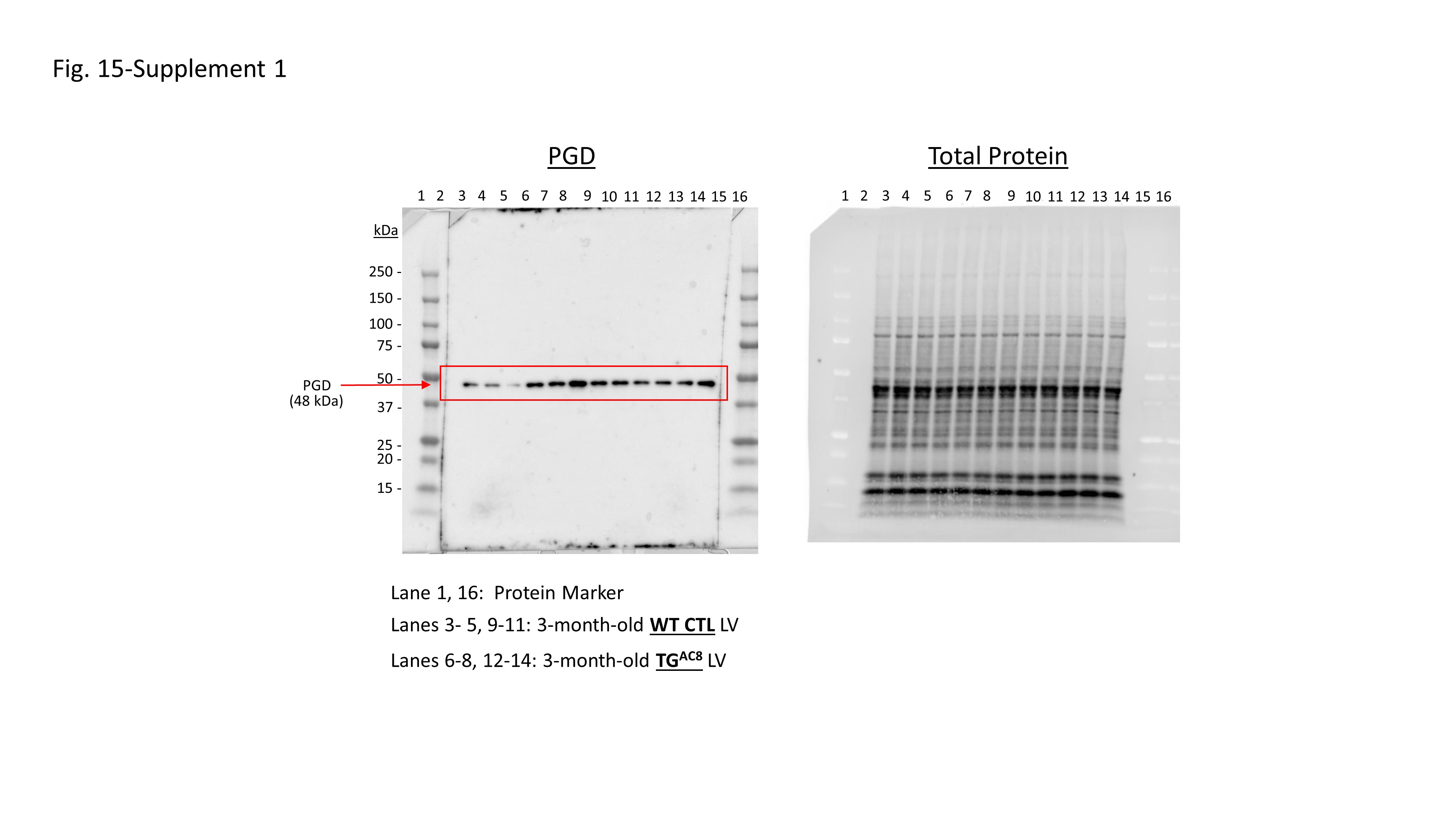

Supplement: Figure 12—figure supplement 1—source data 2. [file elife-80949-fig12-figsupp1-data2.zip › Figure 12-supplement 1 source data 2/Uncropped Images/PGD.JPG]

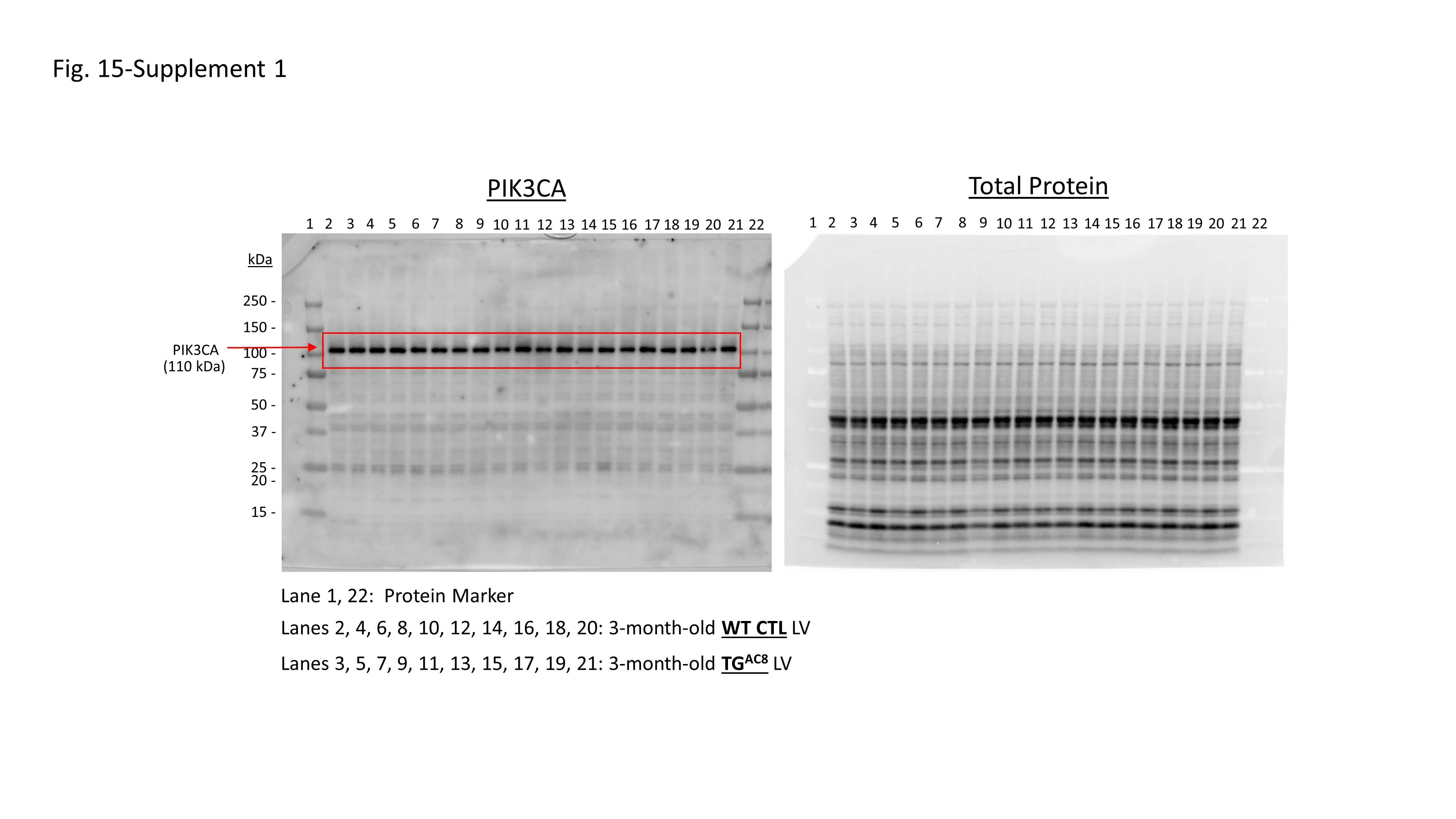

Supplement: Figure 12—figure supplement 1—source data 2. [file elife-80949-fig12-figsupp1-data2.zip › Figure 12-supplement 1 source data 2/Uncropped Images/PIK3CA.JPG]

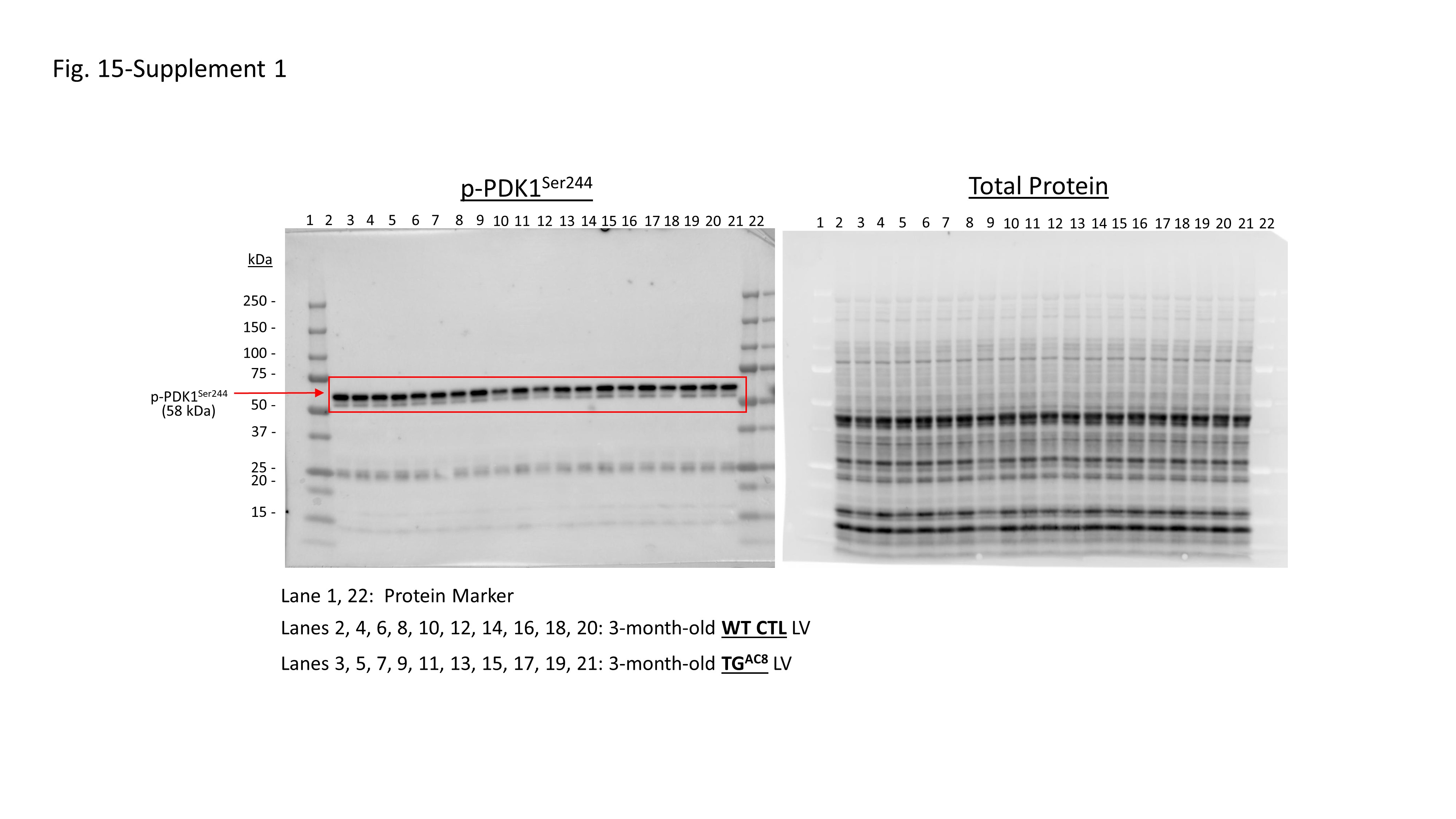

Supplement: Figure 12—figure supplement 1—source data 2. [file elife-80949-fig12-figsupp1-data2.zip › Figure 12-supplement 1 source data 2/Uncropped Images/p-PDK1Ser244.JPG]

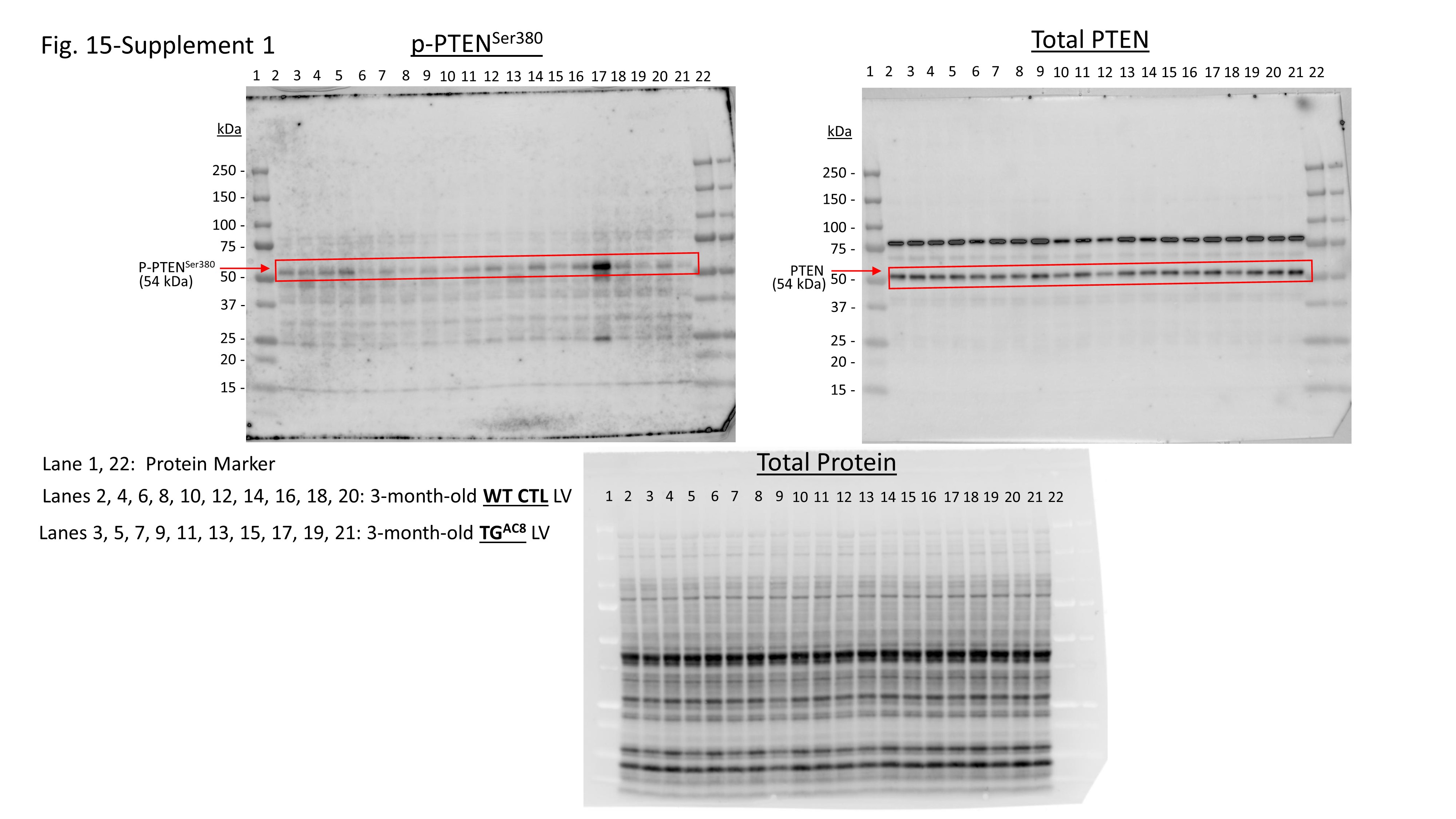

Supplement: Figure 12—figure supplement 1—source data 2. [file elife-80949-fig12-figsupp1-data2.zip › Figure 12-supplement 1 source data 2/Uncropped Images/PTEN.JPG]

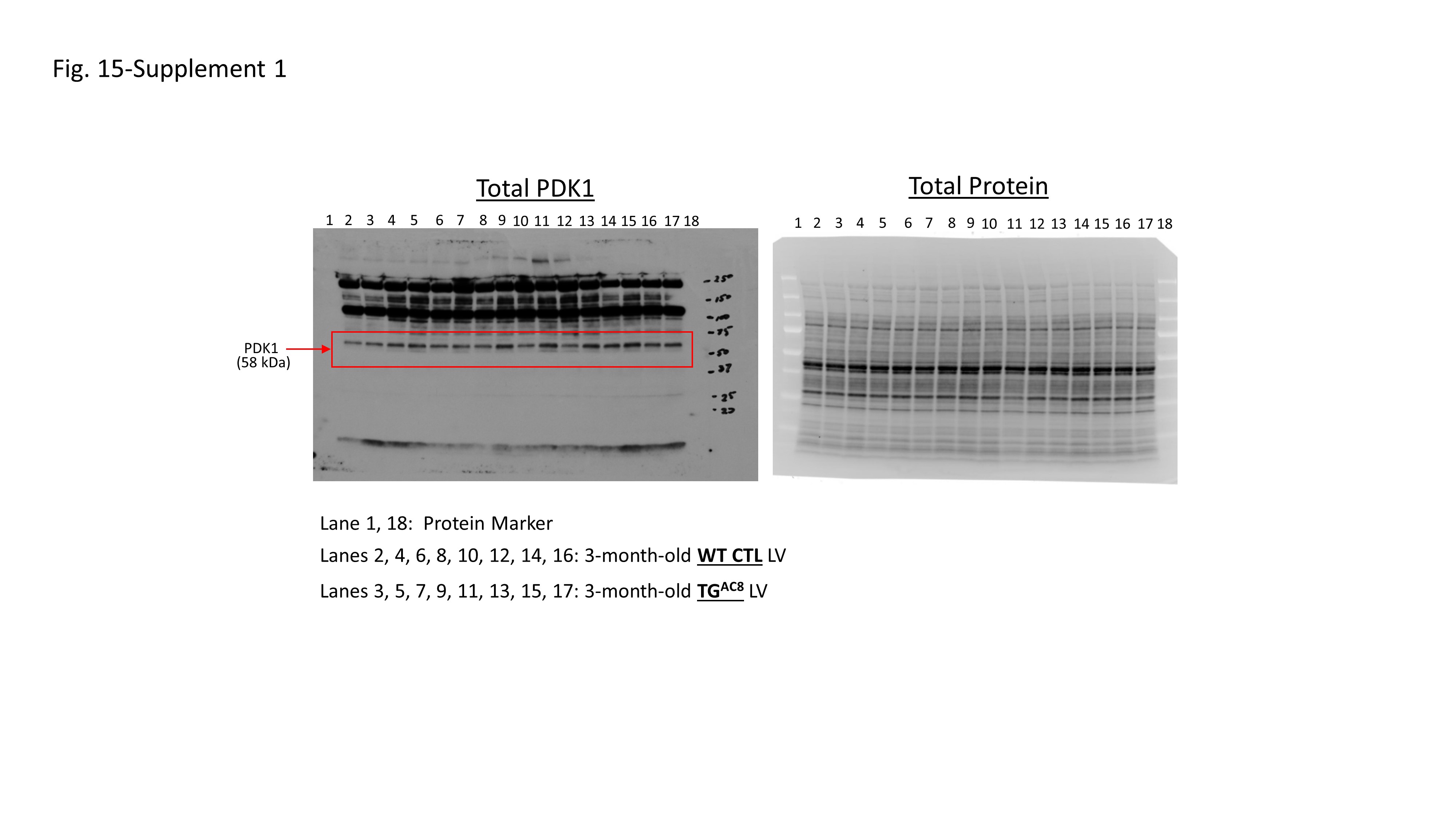

Supplement: Figure 12—figure supplement 1—source data 2. [file elife-80949-fig12-figsupp1-data2.zip › Figure 12-supplement 1 source data 2/Uncropped Images/Total PDK1.JPG]

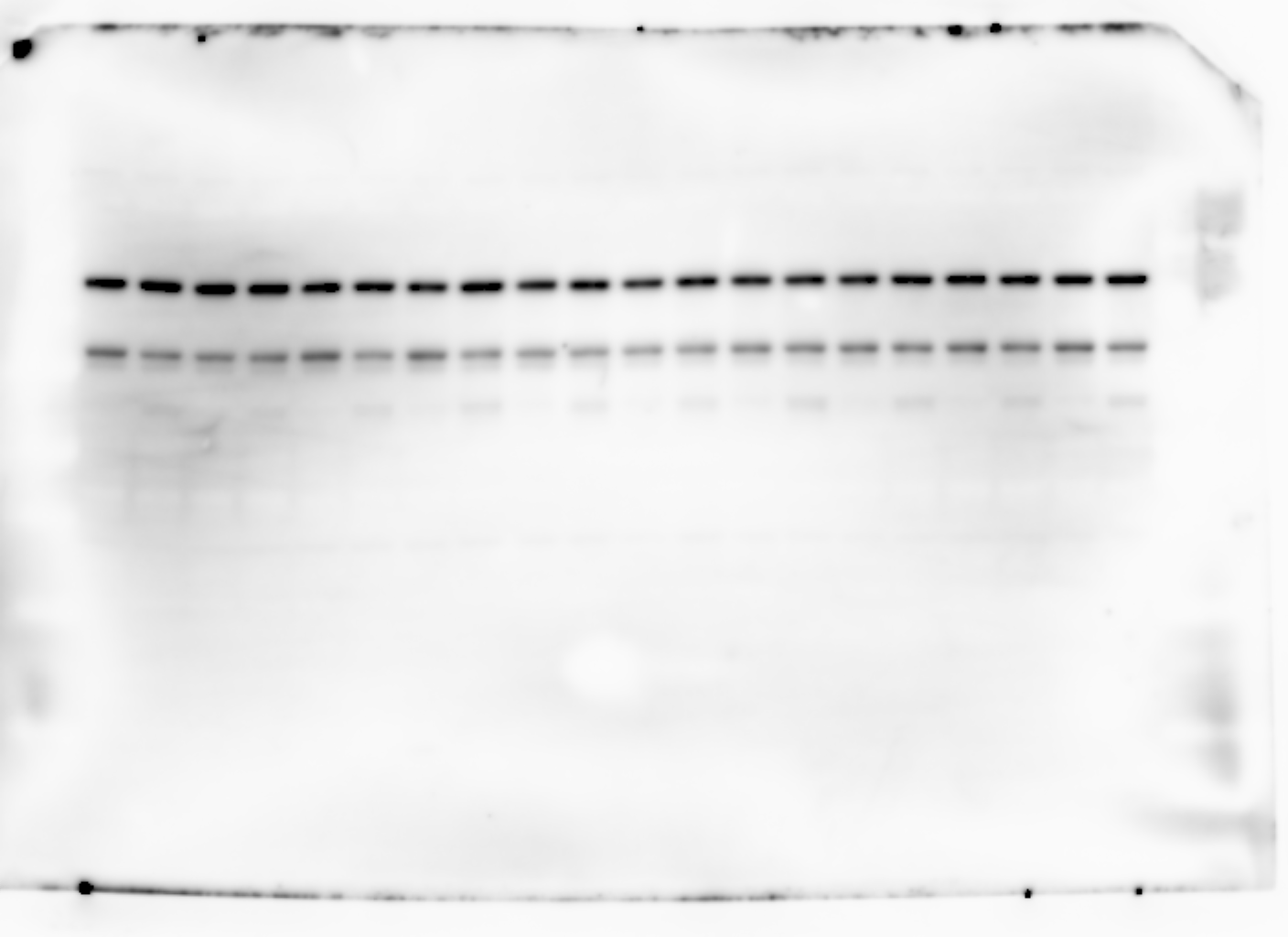

Supplement: Figure 13—figure supplement 1—source data 1. [file elife-80949-fig13-figsupp1-data1.zip › Figure 13-supplement 1 source data 1/PDE4B/PDE4B/DR PDE4B blot9 WPP 2018.02.28_13.16.46_Ch.tif]

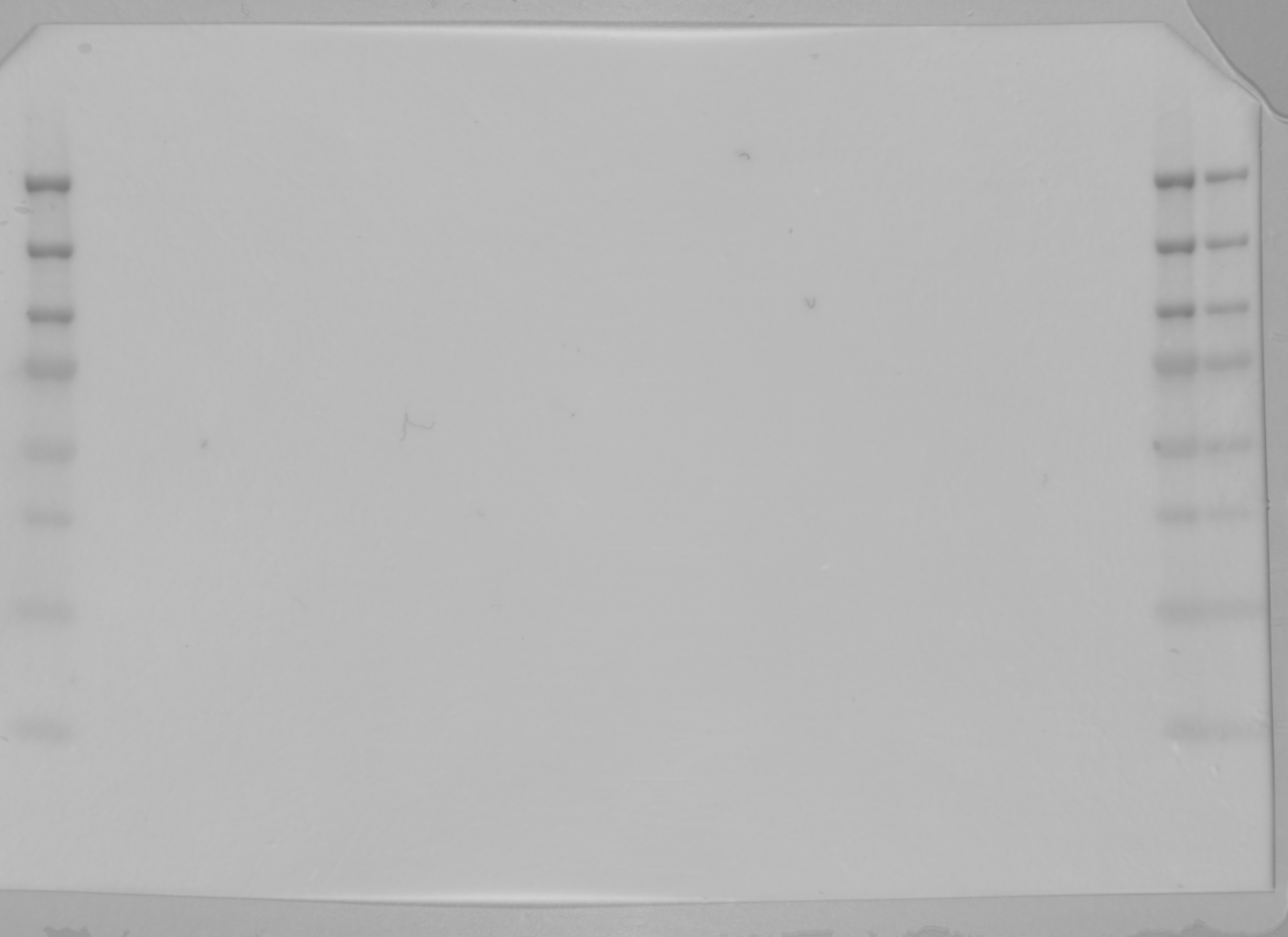

Supplement: Figure 13—figure supplement 1—source data 1. [file elife-80949-fig13-figsupp1-data1.zip › Figure 13-supplement 1 source data 1/PDE4B/PDE4B/DR PDE4B blot9 WPP 2018.02.28_13.16.46_Ch-Marker.tif]

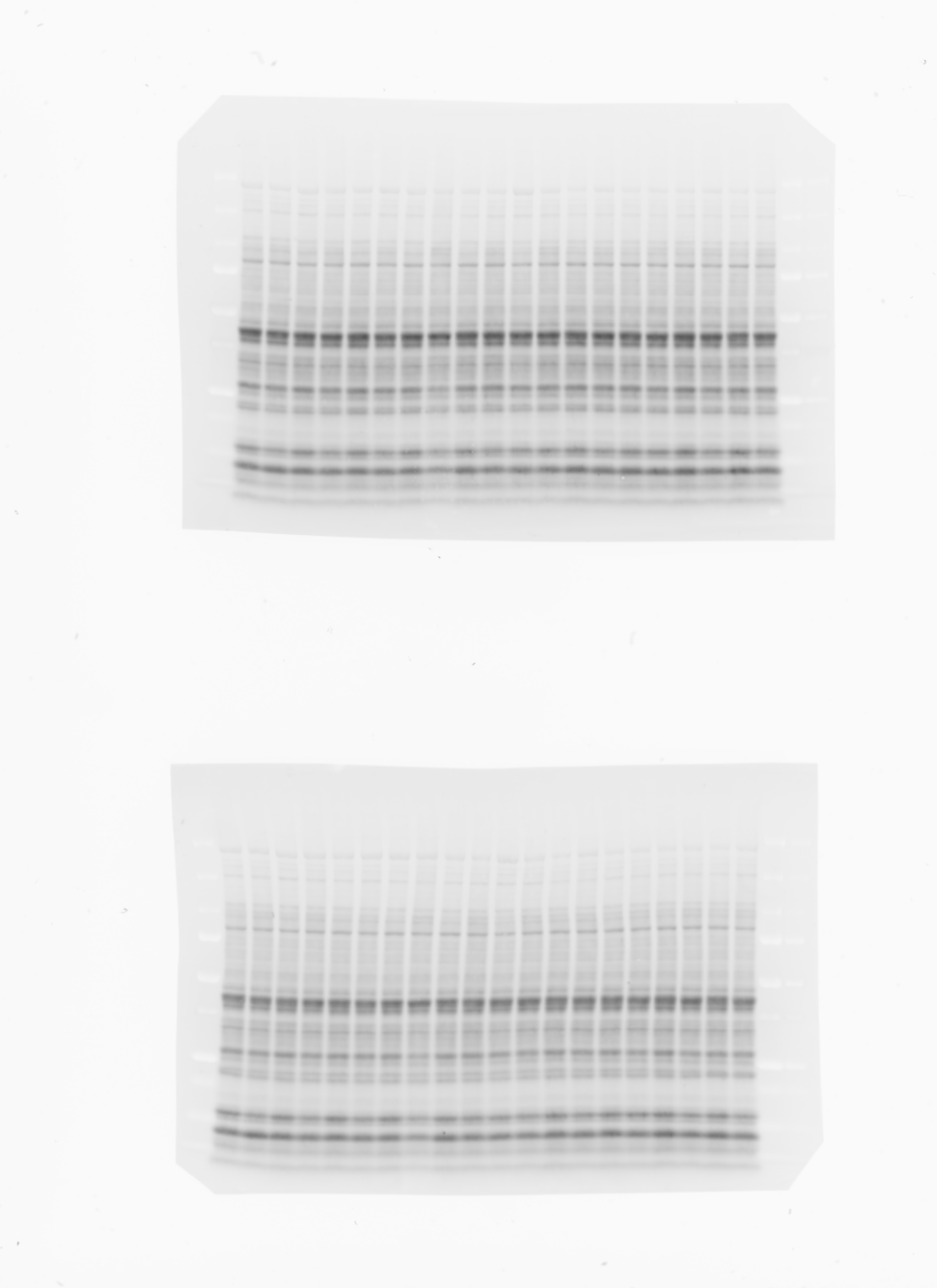

Supplement: Figure 13—figure supplement 1—source data 1. [file elife-80949-fig13-figsupp1-data1.zip › Figure 13-supplement 1 source data 1/PDE4B/Total Protein/DR Tot.Prot. Blt9,10 2018.02.14_13.46.44_Fl-UV.tif]

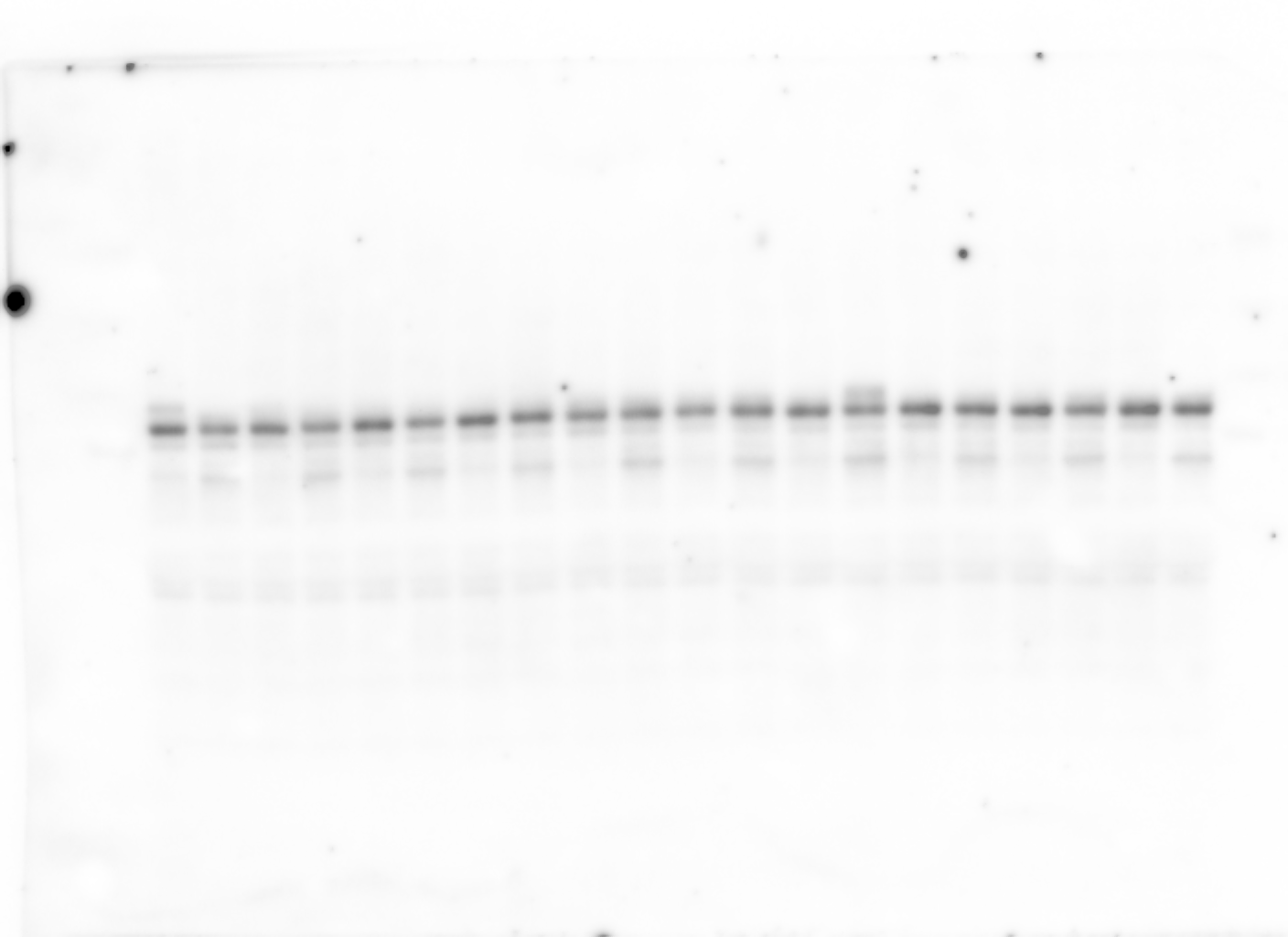

Supplement: Figure 13—figure supplement 1—source data 1. [file elife-80949-fig13-figsupp1-data1.zip › Figure 13-supplement 1 source data 1/PDE4D/PDE4D/DR PDE4D Blt6 WPP 2018.02.23_12.06.49_Ch.tif]

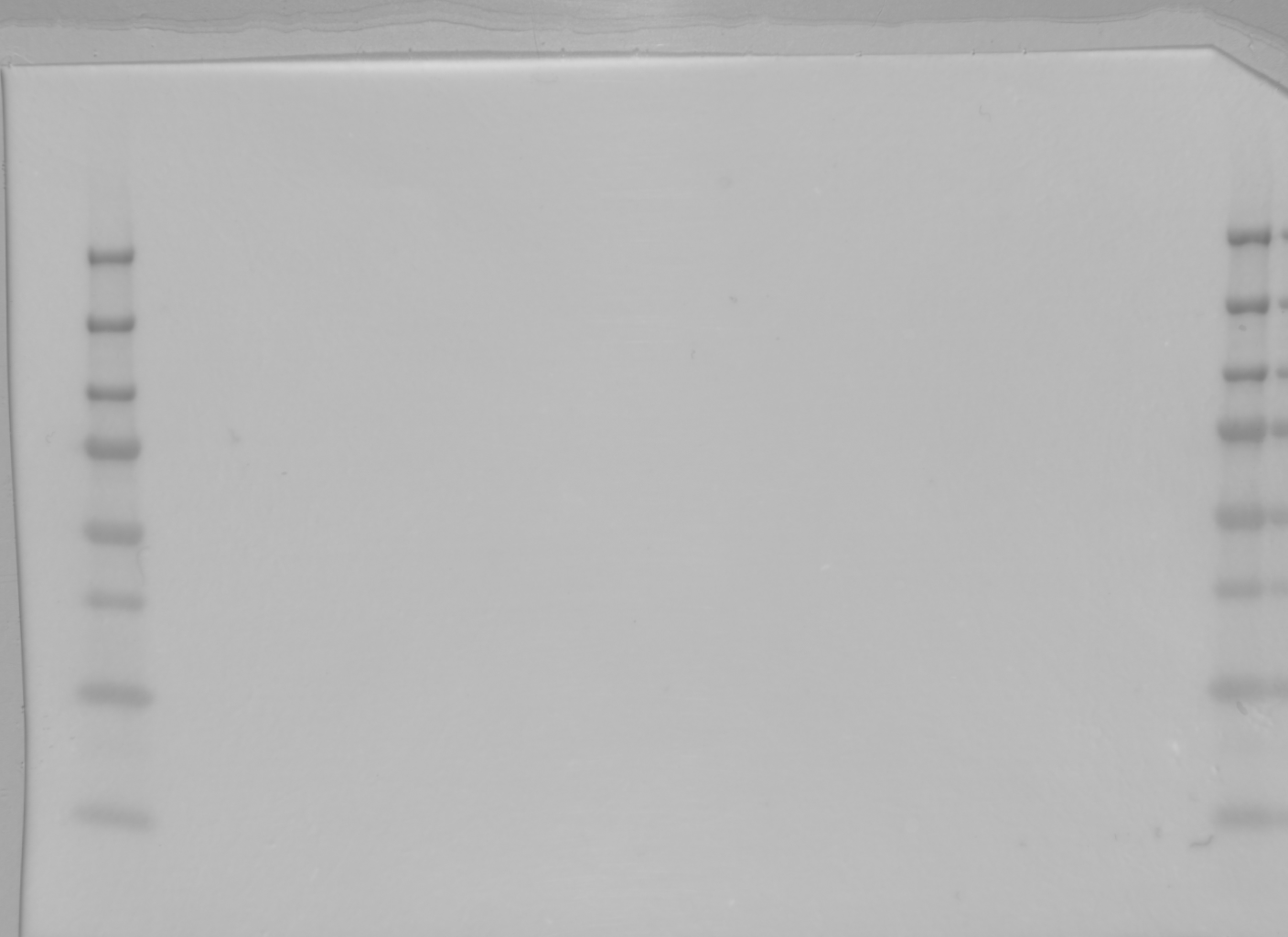

Supplement: Figure 13—figure supplement 1—source data 1. [file elife-80949-fig13-figsupp1-data1.zip › Figure 13-supplement 1 source data 1/PDE4D/PDE4D/DR PDE4D Blt6 WPP 2018.02.23_12.06.49_Ch-Marker.tif]

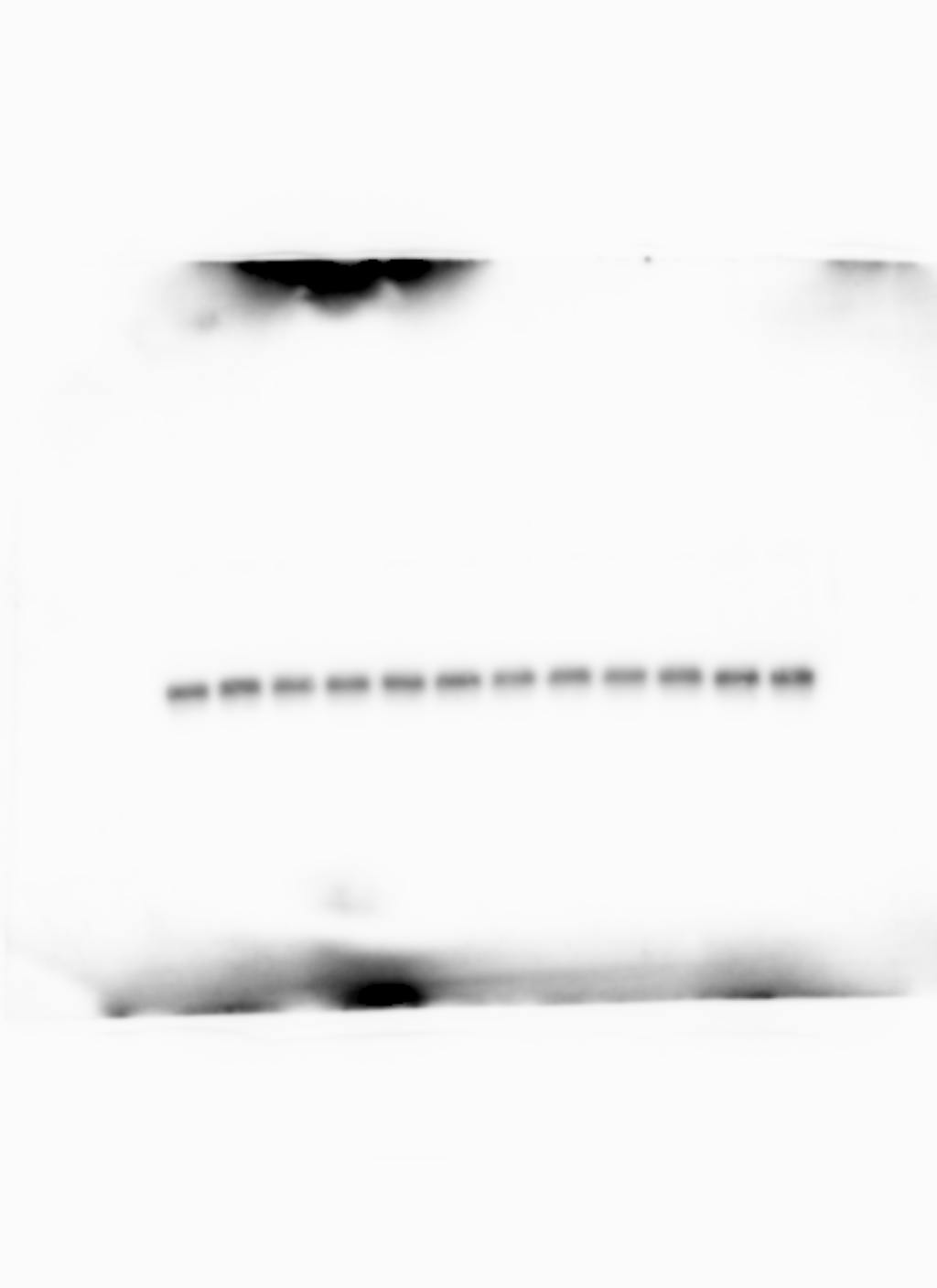

Supplement: Figure 13—figure supplement 1—source data 1. [file elife-80949-fig13-figsupp1-data1.zip › Figure 13-supplement 1 source data 1/PP1/PP1/WO_PP1_Blt87 1min 2021.01.11_12.23.18_Ch.tif]

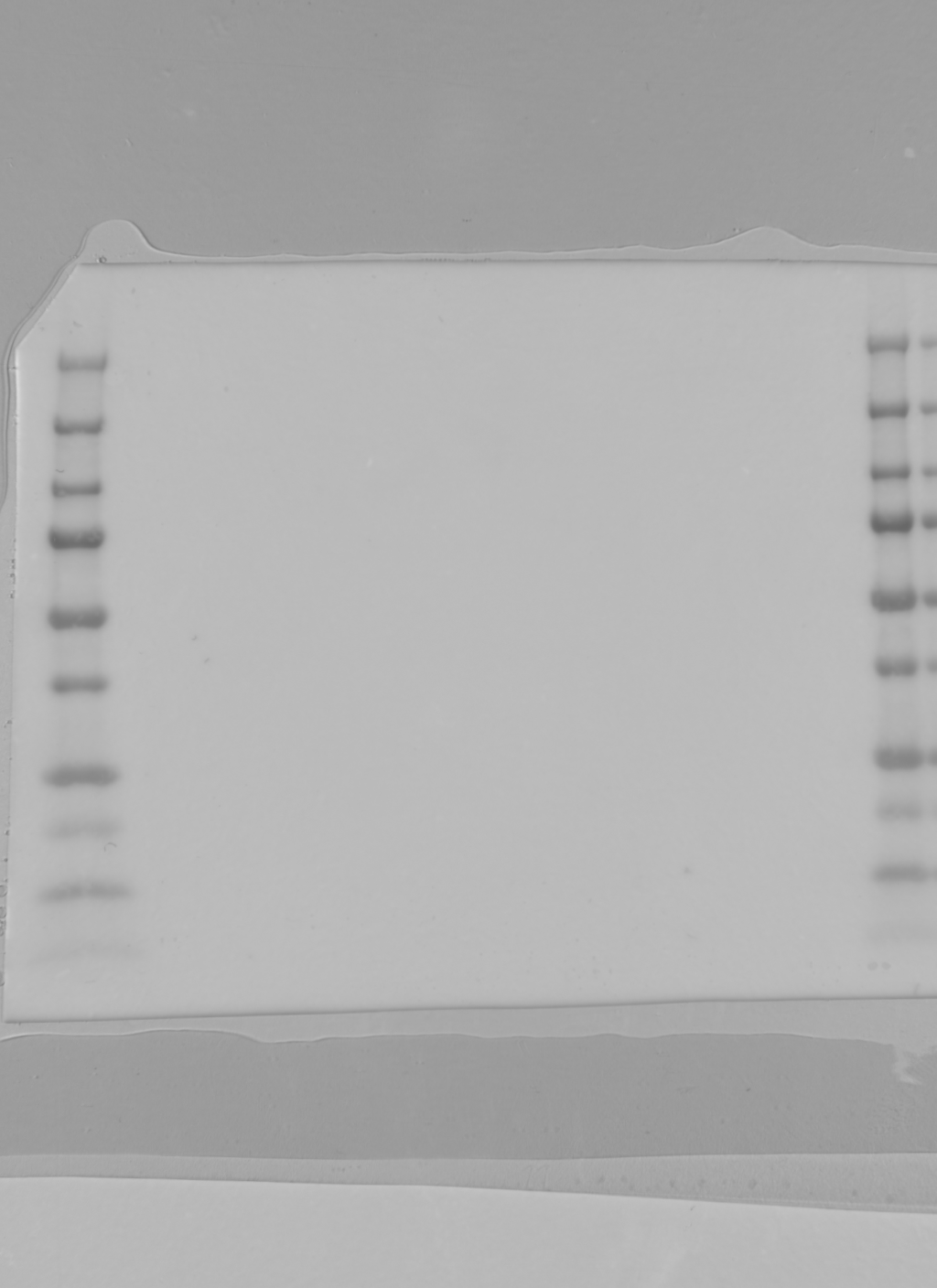

Supplement: Figure 13—figure supplement 1—source data 1. [file elife-80949-fig13-figsupp1-data1.zip › Figure 13-supplement 1 source data 1/PP1/PP1/WO_PP1_Blt87 1min 2021.01.11_12.23.18_Ch-Marker.tif]

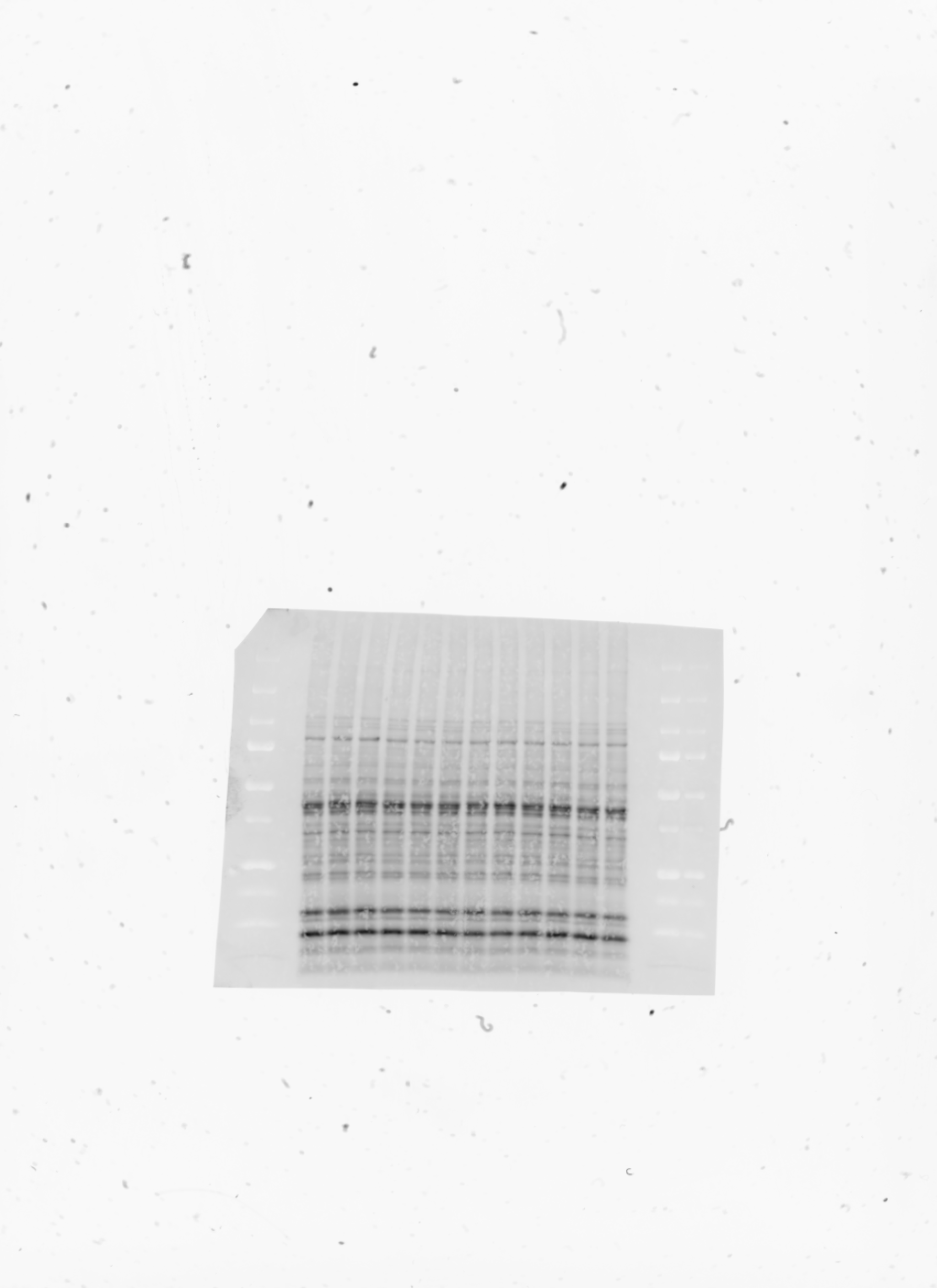

Supplement: Figure 13—figure supplement 1—source data 1. [file elife-80949-fig13-figsupp1-data1.zip › Figure 13-supplement 1 source data 1/PP1/Total Protein/WO_t.prot_blt87 2020.12.22_14.41.27.tif]

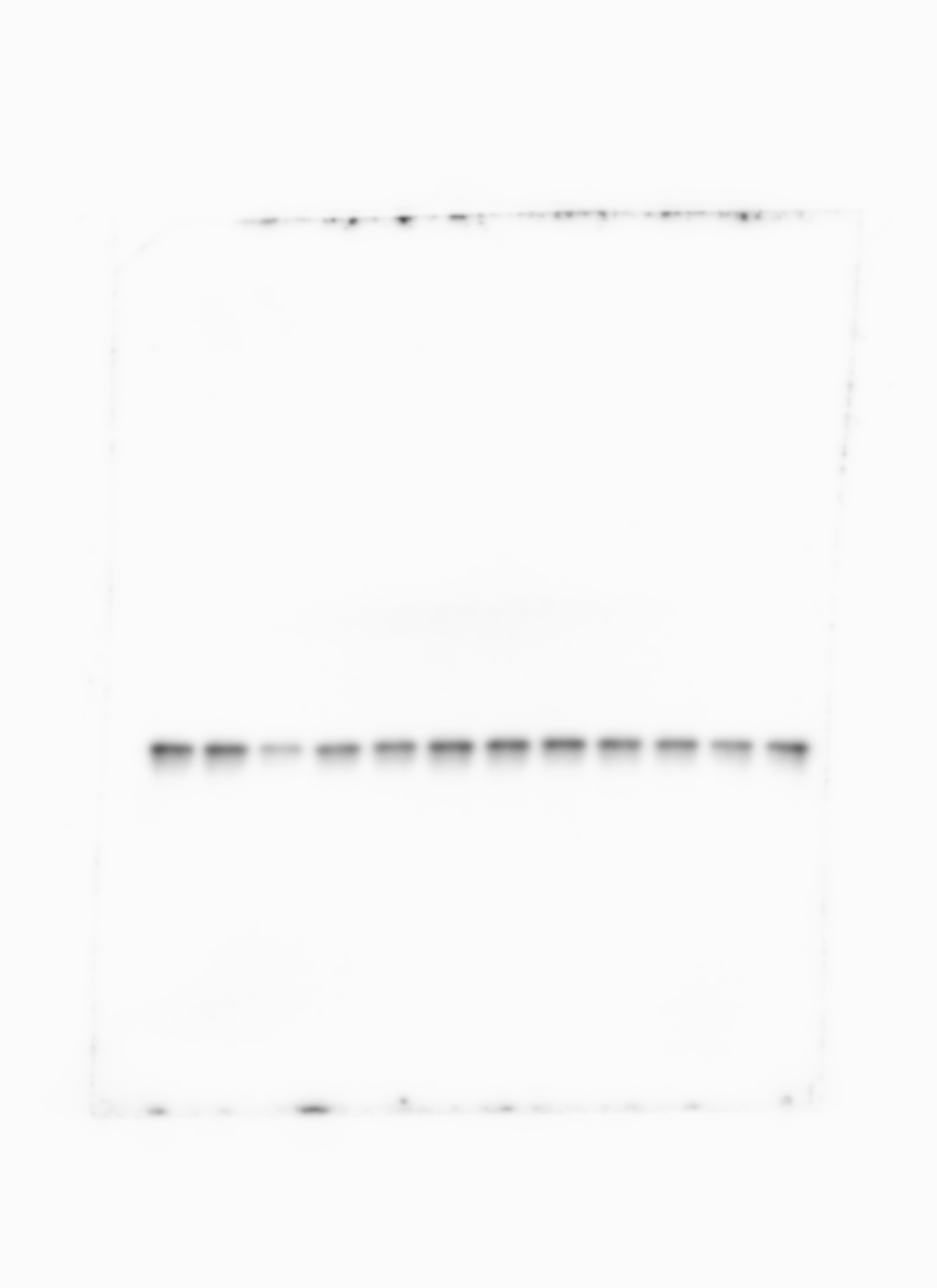

Supplement: Figure 13—figure supplement 1—source data 1. [file elife-80949-fig13-figsupp1-data1.zip › Figure 13-supplement 1 source data 1/PP2A/PP2A/DR PP2Aca Blot75 2020.09.23_11.10.56_Ch.tif]

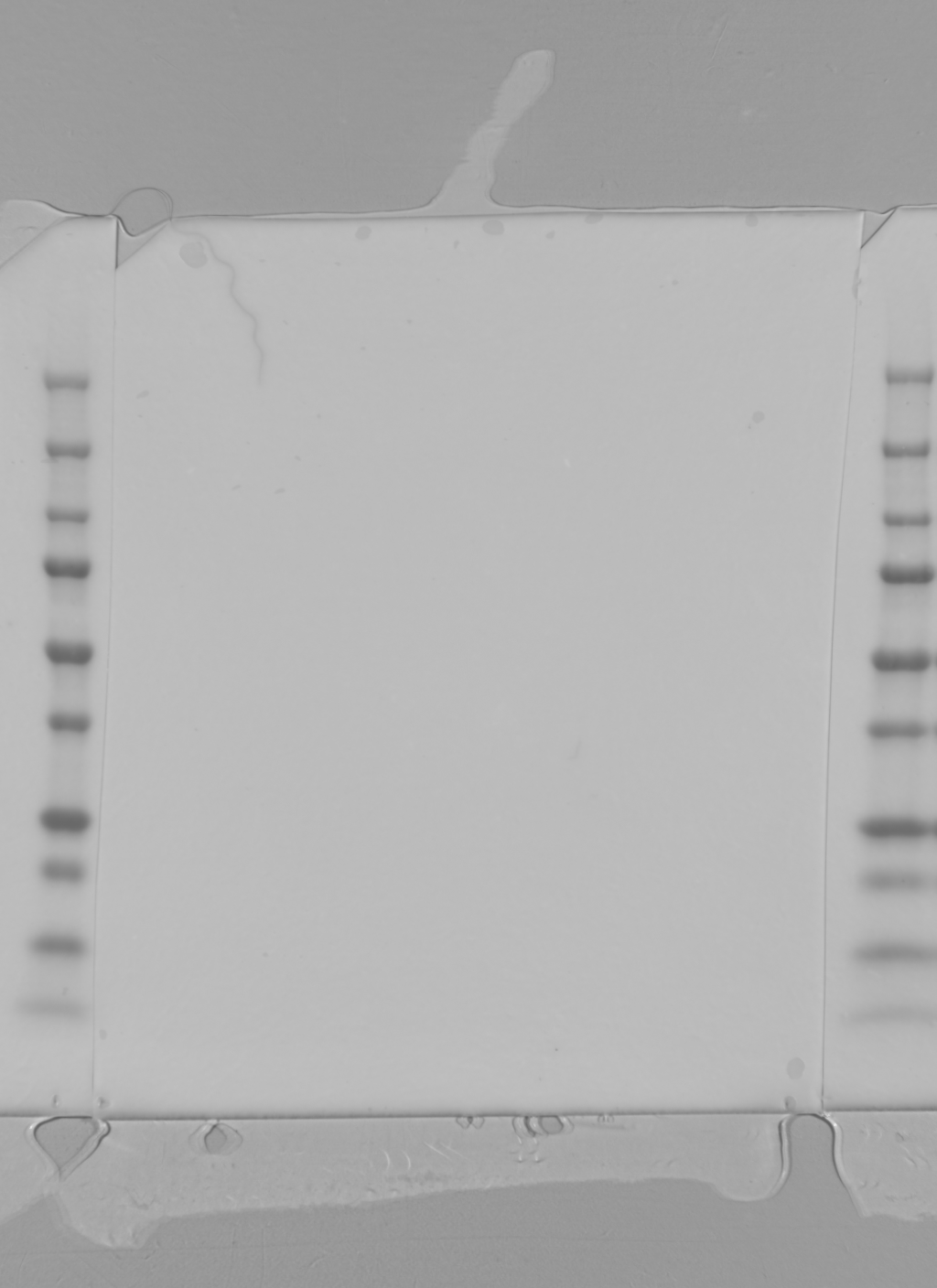

Supplement: Figure 13—figure supplement 1—source data 1. [file elife-80949-fig13-figsupp1-data1.zip › Figure 13-supplement 1 source data 1/PP2A/PP2A/DR PP2Aca Blot75 2020.09.23_11.10.56_Ch-Marker.tif]

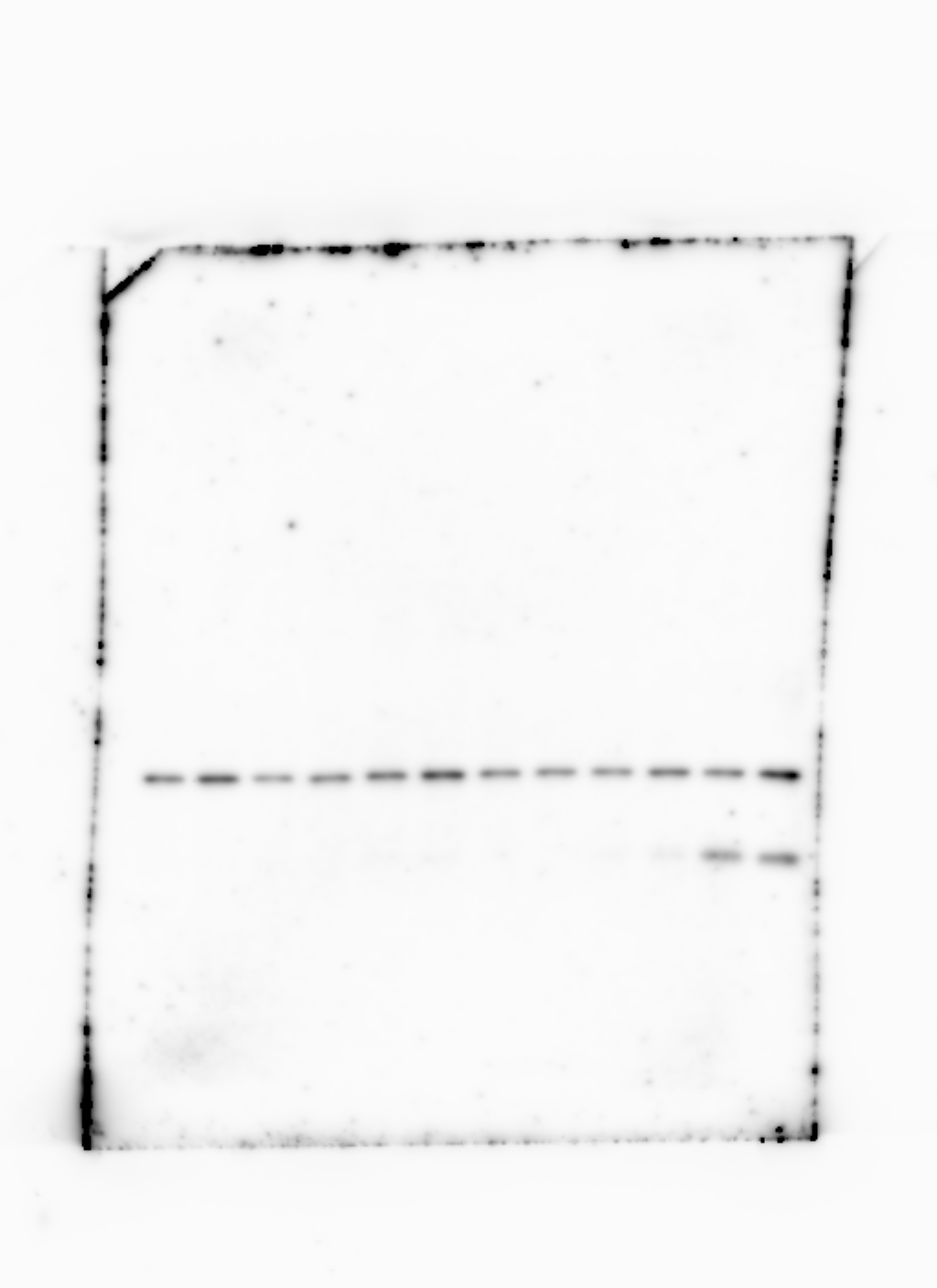

Supplement: Figure 13—figure supplement 1—source data 1. [file elife-80949-fig13-figsupp1-data1.zip › Figure 13-supplement 1 source data 1/PP2A/p-PP2A Tyr307/DR pPP2A Y307 Blt75 2020.09.22_11.30.59_Ch.tif]

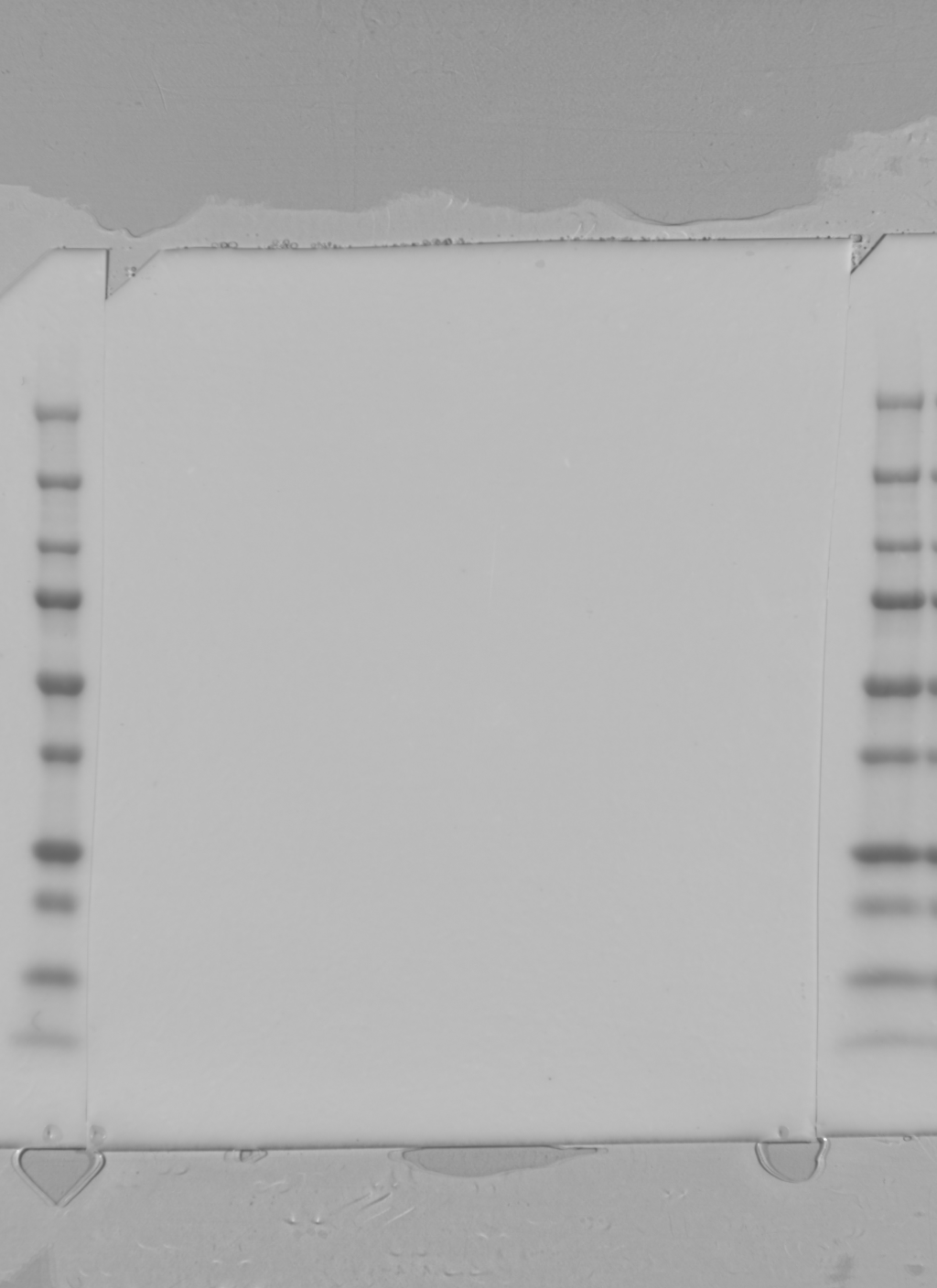

Supplement: Figure 13—figure supplement 1—source data 1. [file elife-80949-fig13-figsupp1-data1.zip › Figure 13-supplement 1 source data 1/PP2A/p-PP2A Tyr307/DR pPP2A Y307 Blt75 2020.09.22_11.30.59_Ch-Marker.tif]

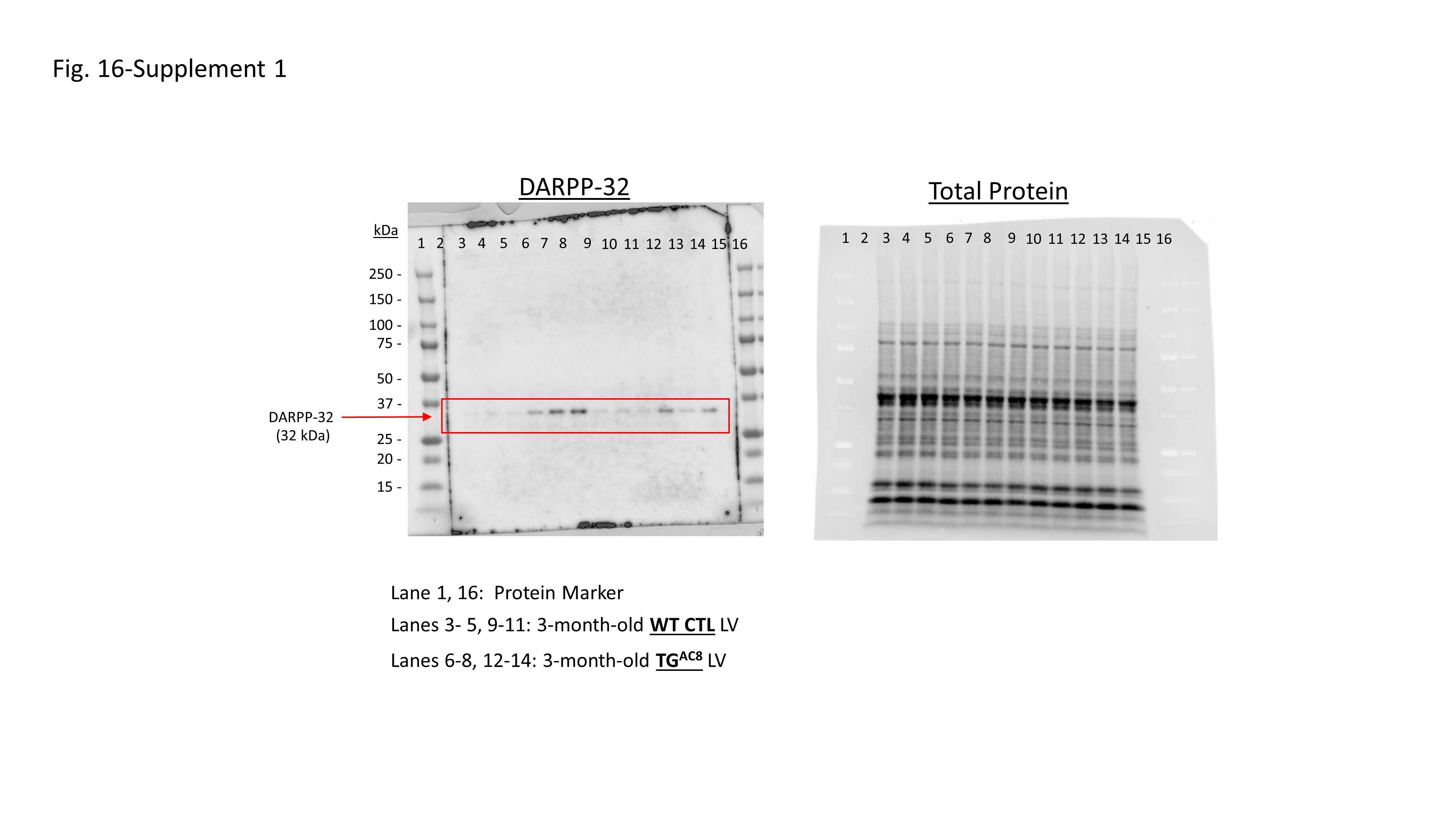

Supplement: Figure 13—figure supplement 1—source data 1. [file elife-80949-fig13-figsupp1-data1.zip › Figure 13-supplement 1 source data 1/Uncropped images/DARPP-32.JPG]

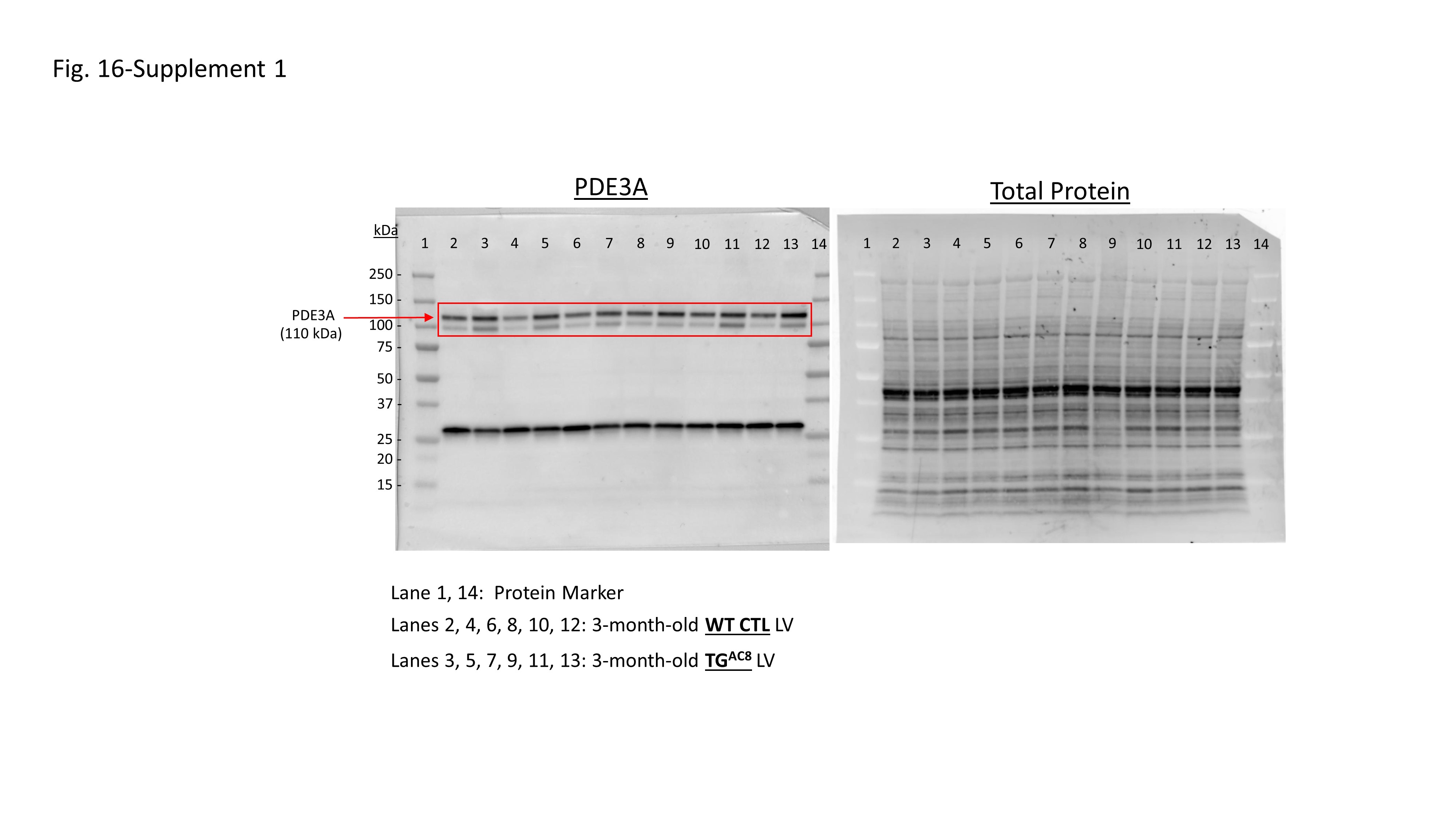

Supplement: Figure 13—figure supplement 1—source data 1. [file elife-80949-fig13-figsupp1-data1.zip › Figure 13-supplement 1 source data 1/Uncropped images/PDE3A.JPG]

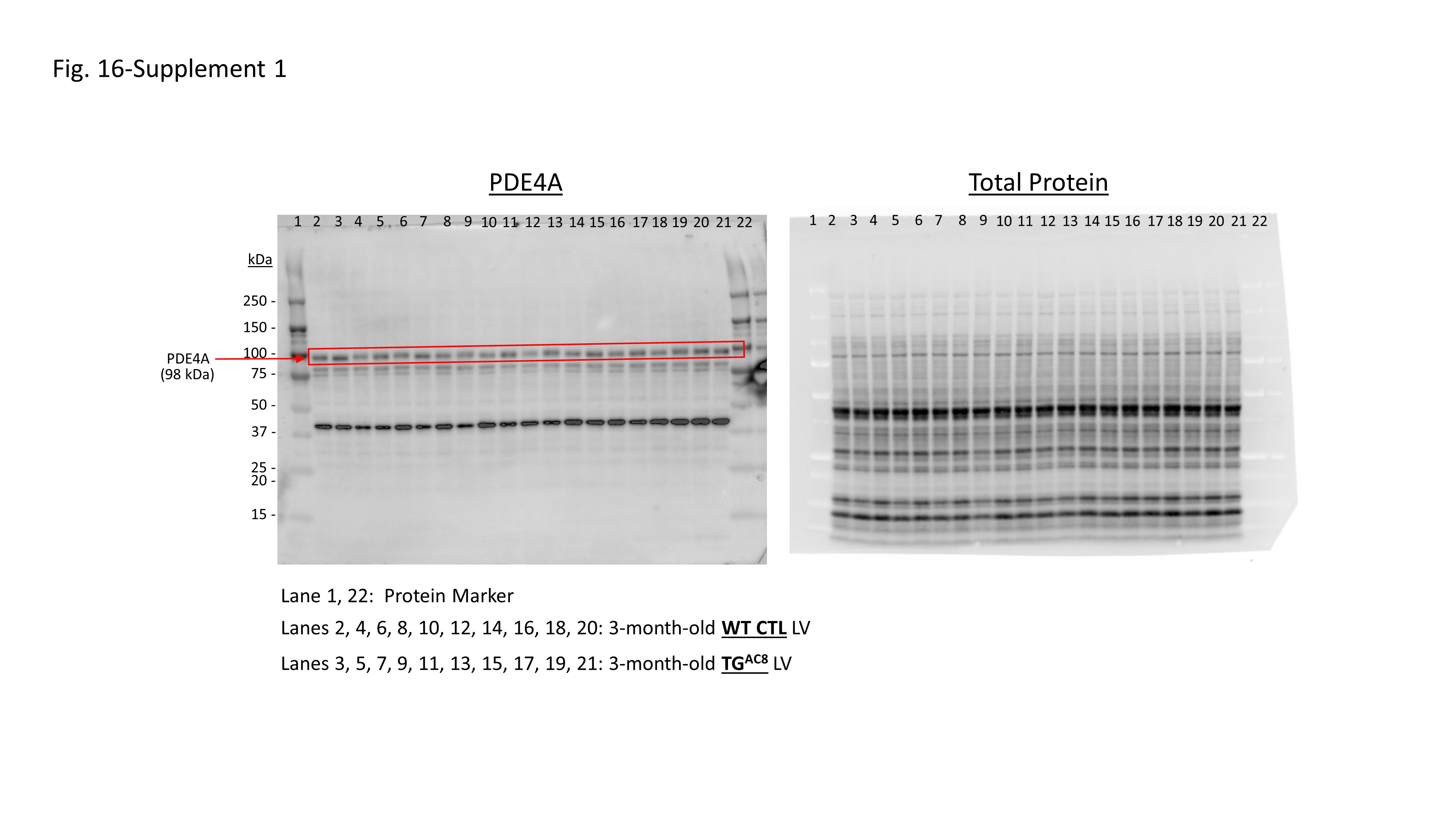

Supplement: Figure 13—figure supplement 1—source data 1. [file elife-80949-fig13-figsupp1-data1.zip › Figure 13-supplement 1 source data 1/Uncropped images/PDE4A.JPG]

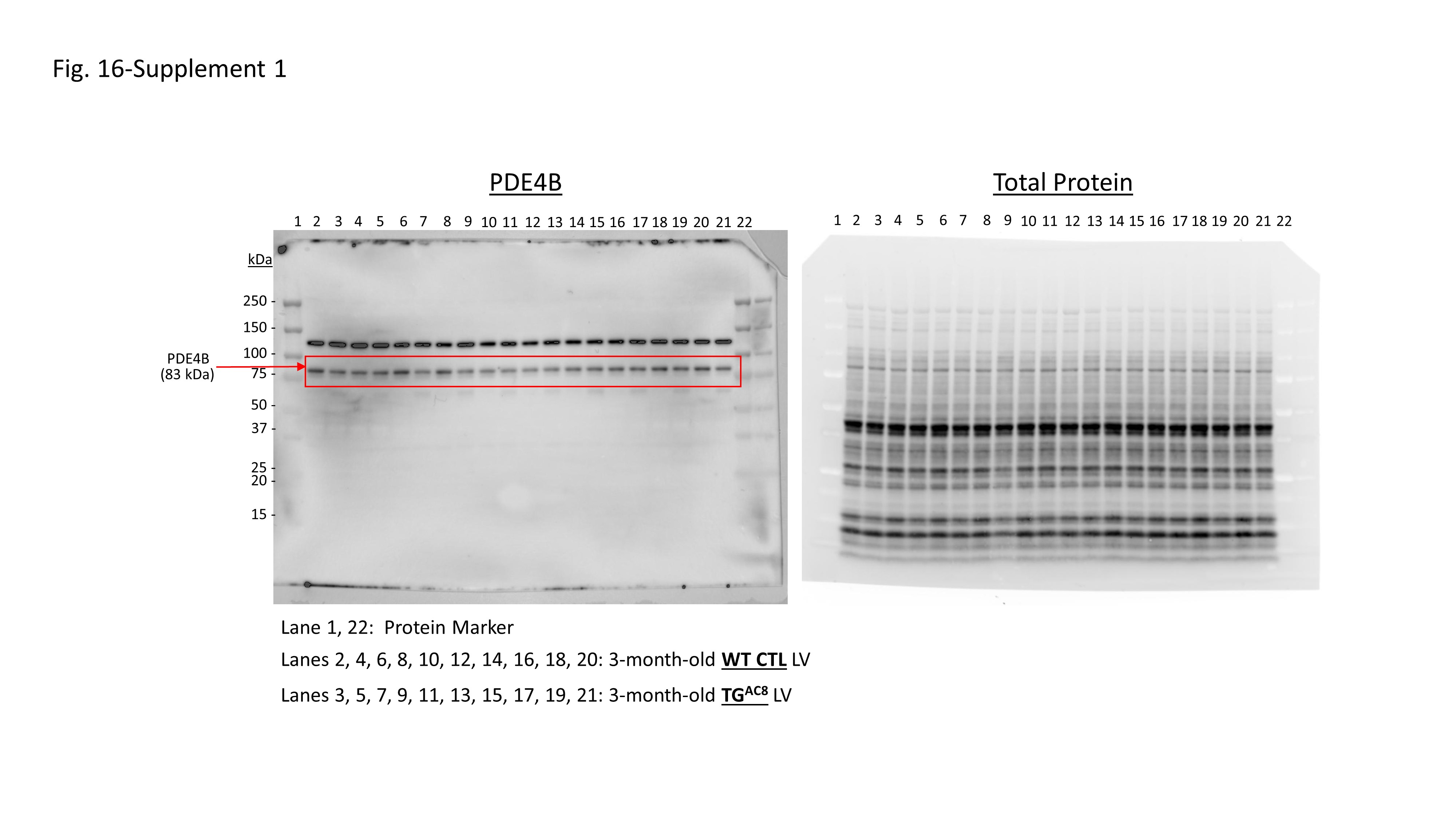

Supplement: Figure 13—figure supplement 1—source data 1. [file elife-80949-fig13-figsupp1-data1.zip › Figure 13-supplement 1 source data 1/Uncropped images/PDE4B.JPG]

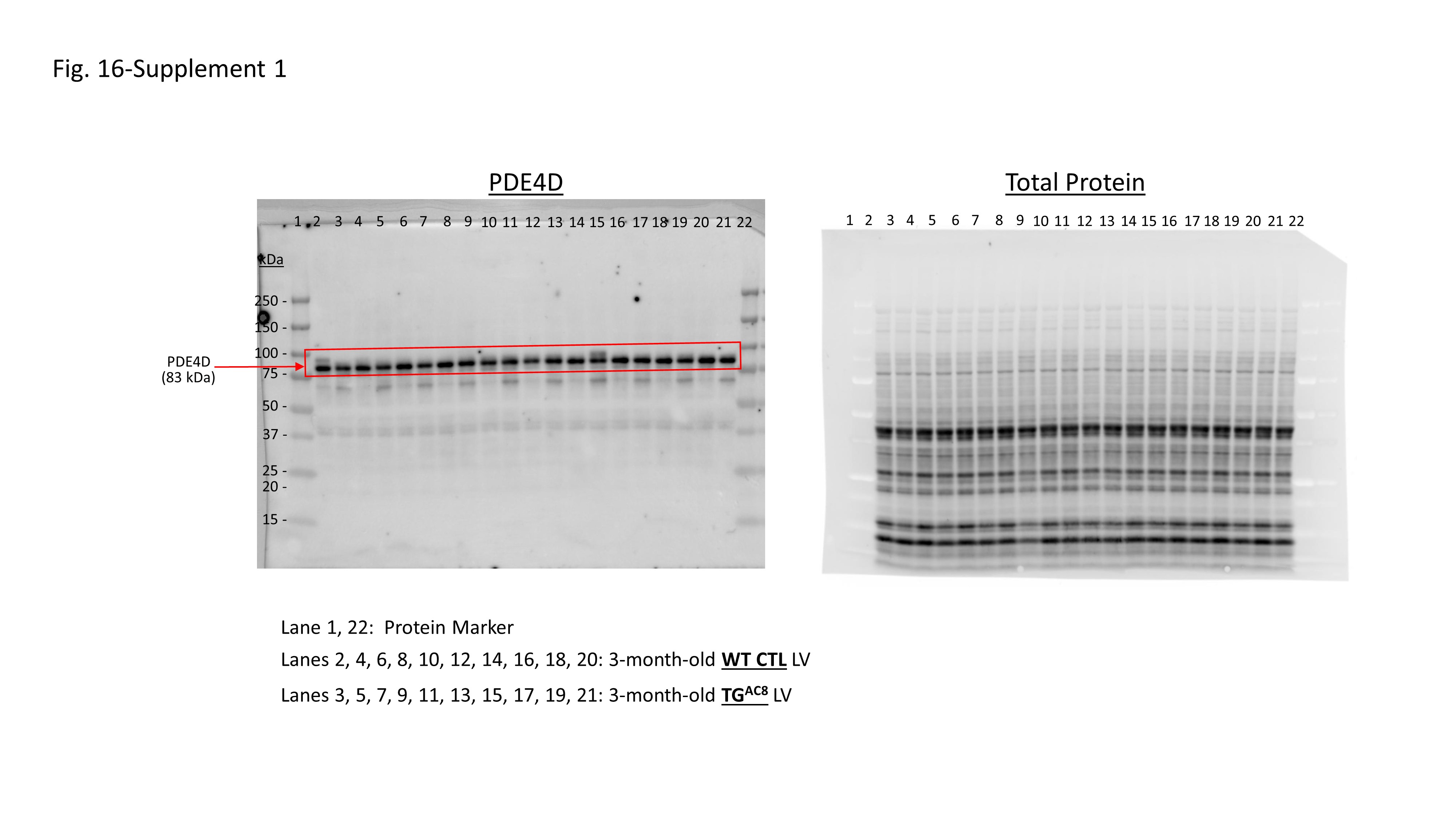

Supplement: Figure 13—figure supplement 1—source data 1. [file elife-80949-fig13-figsupp1-data1.zip › Figure 13-supplement 1 source data 1/Uncropped images/PDE4D.JPG]

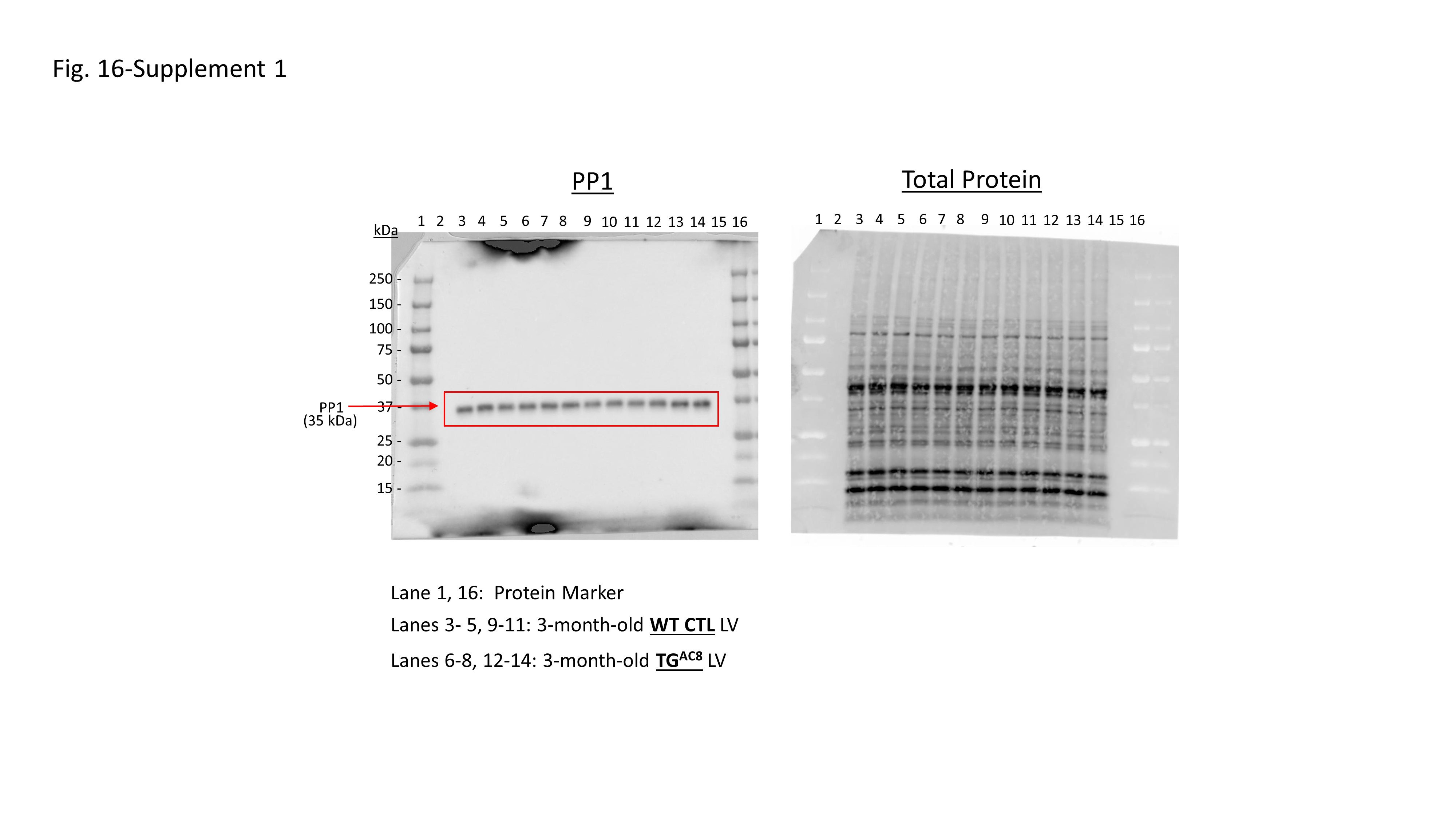

Supplement: Figure 13—figure supplement 1—source data 1. [file elife-80949-fig13-figsupp1-data1.zip › Figure 13-supplement 1 source data 1/Uncropped images/PP1.JPG]

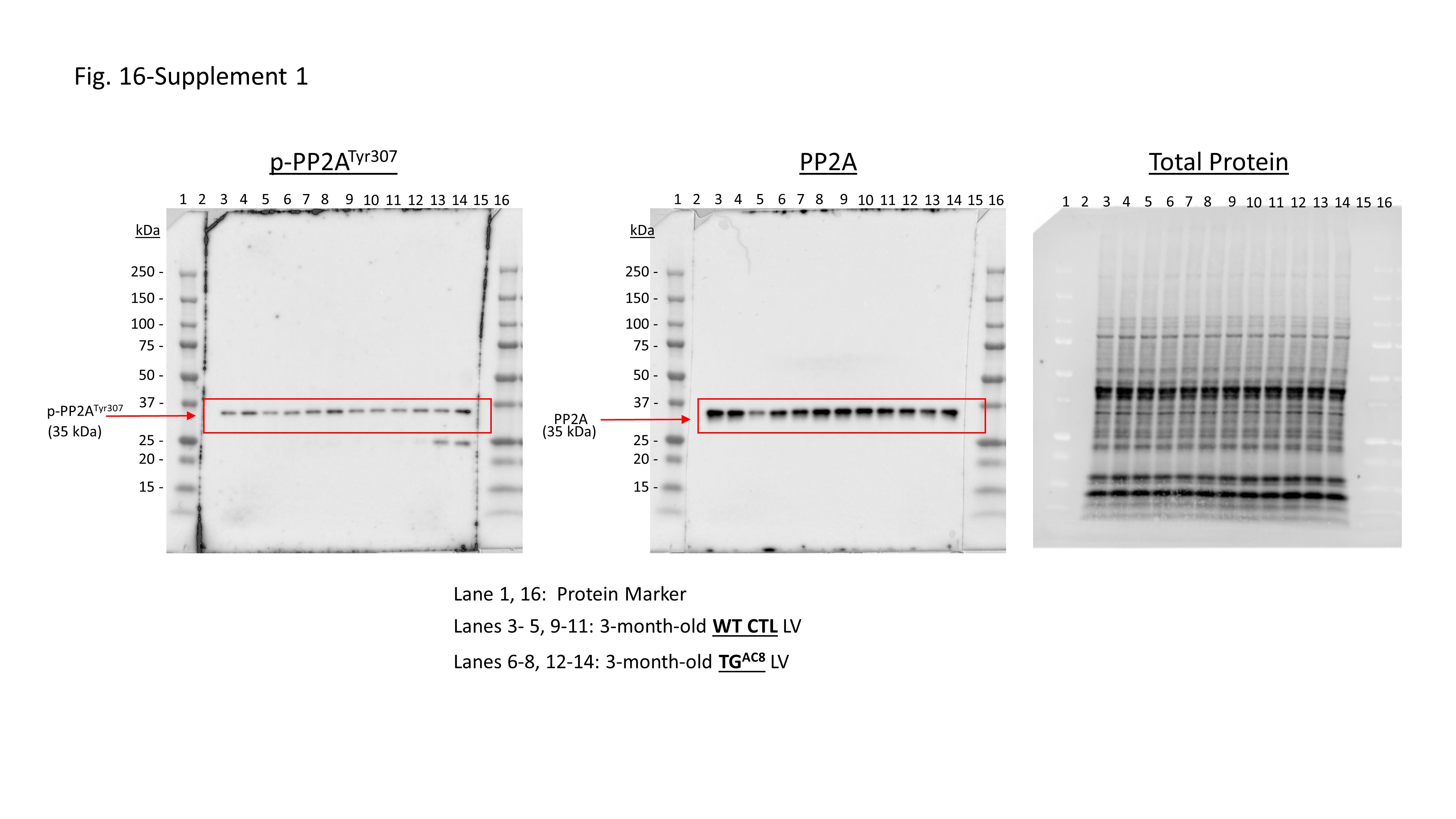

Supplement: Figure 13—figure supplement 1—source data 1. [file elife-80949-fig13-figsupp1-data1.zip › Figure 13-supplement 1 source data 1/Uncropped images/PP2A.JPG]

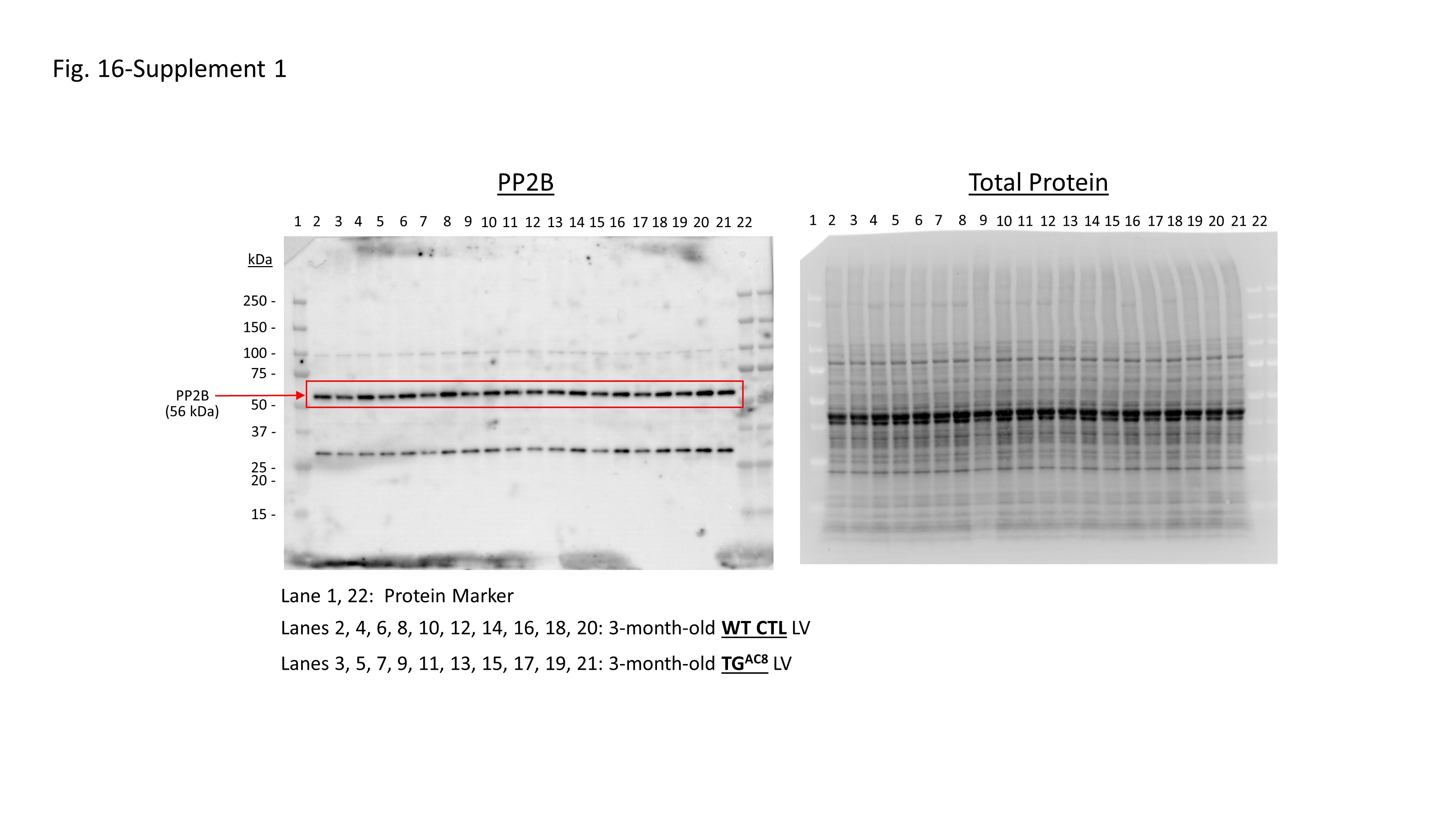

Supplement: Figure 13—figure supplement 1—source data 1. [file elife-80949-fig13-figsupp1-data1.zip › Figure 13-supplement 1 source data 1/Uncropped images/PP2B.JPG]

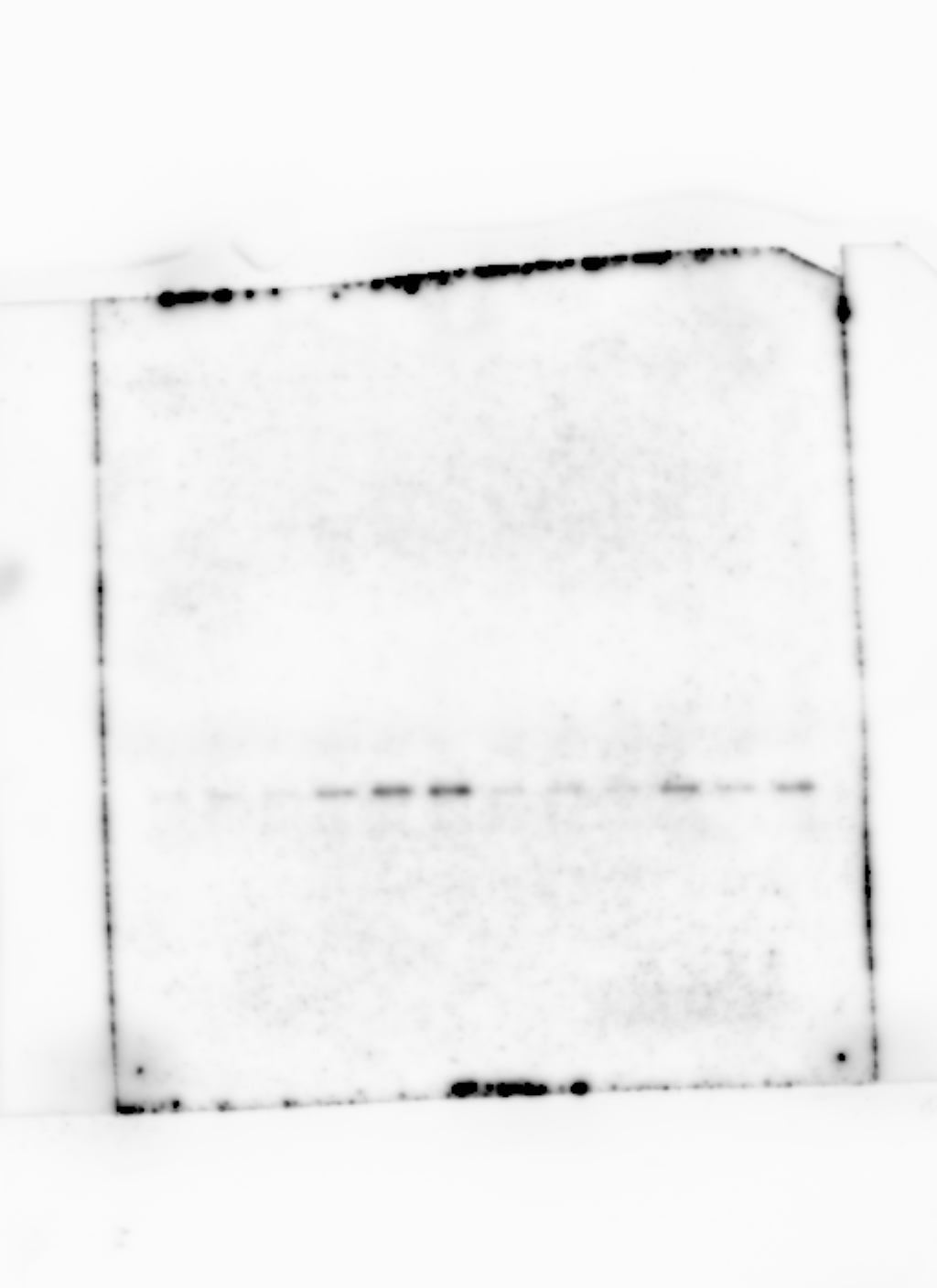

Supplement: Figure 13—figure supplement 1—source data 2. [file elife-80949-fig13-figsupp1-data2.zip › Figure 13-supplement 1 source data 2/DARPP-32/DARPP-32/DR DARPP32 Blot76 2020.09.23_12.08.51_Ch.tif]

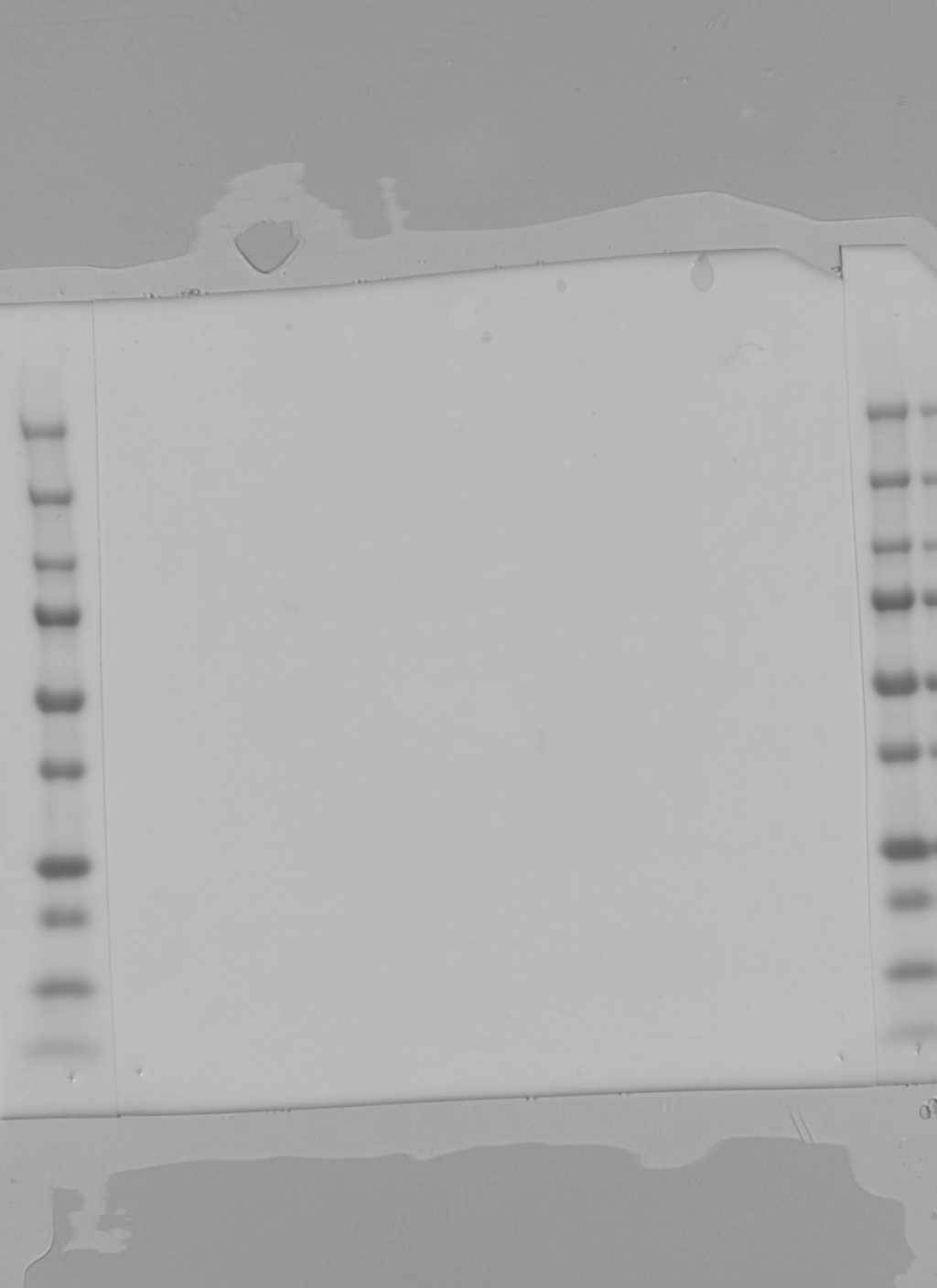

Supplement: Figure 13—figure supplement 1—source data 2. [file elife-80949-fig13-figsupp1-data2.zip › Figure 13-supplement 1 source data 2/DARPP-32/DARPP-32/DR DARPP32 Blot76 2020.09.23_12.08.51_Ch-Marker.tif]

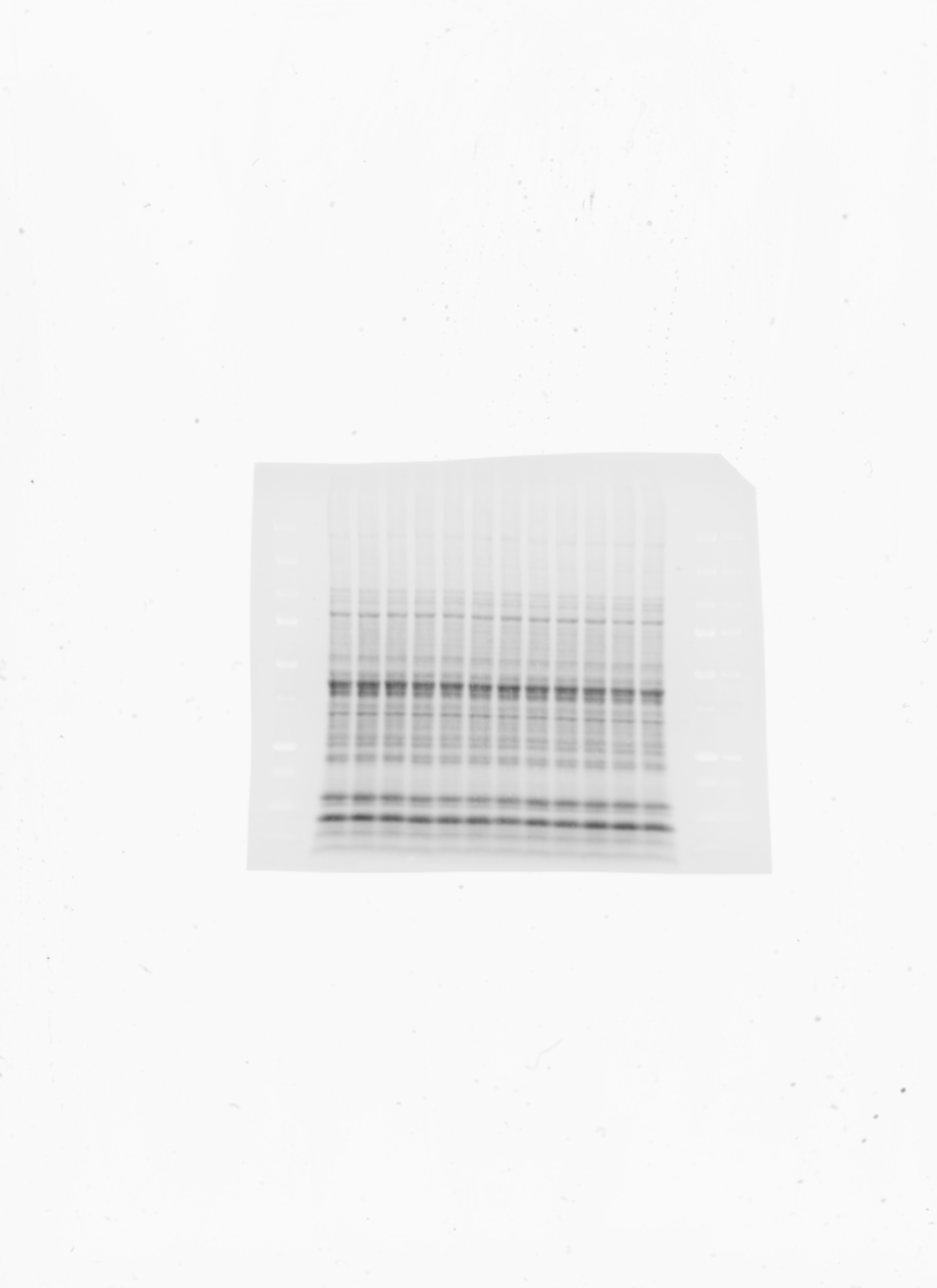

Supplement: Figure 13—figure supplement 1—source data 2. [file elife-80949-fig13-figsupp1-data2.zip › Figure 13-supplement 1 source data 2/DARPP-32/Total Protein/DR TProt. Blot76 2020.09.21_12.12.54_Fl-UV.tif]

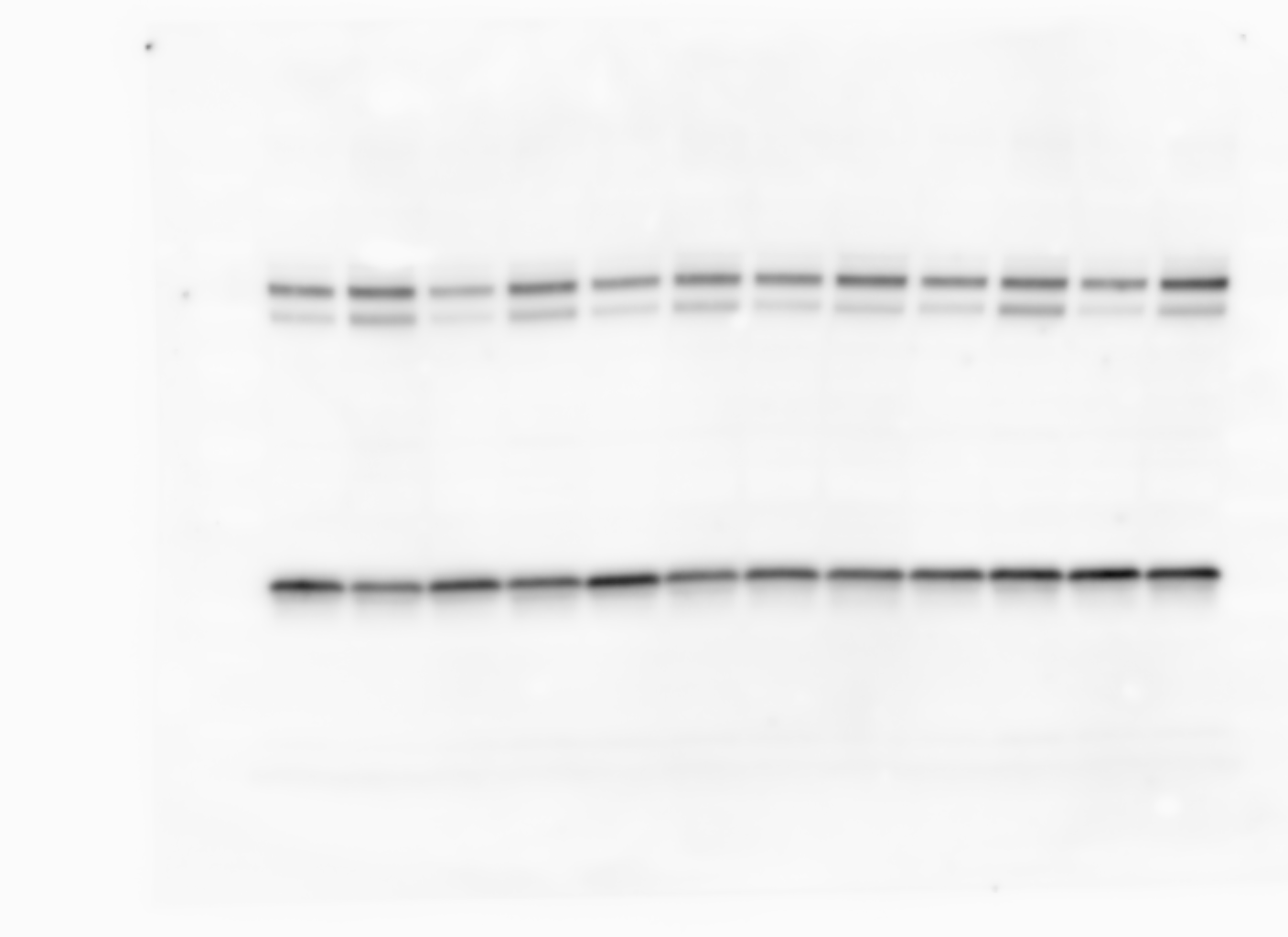

Supplement: Figure 13—figure supplement 1—source data 2. [file elife-80949-fig13-figsupp1-data2.zip › Figure 13-supplement 1 source data 2/PDE3A/PDE3A/DR Blot2 PDE3A WPP 2018.01.11_10.55.27_Ch.tif]

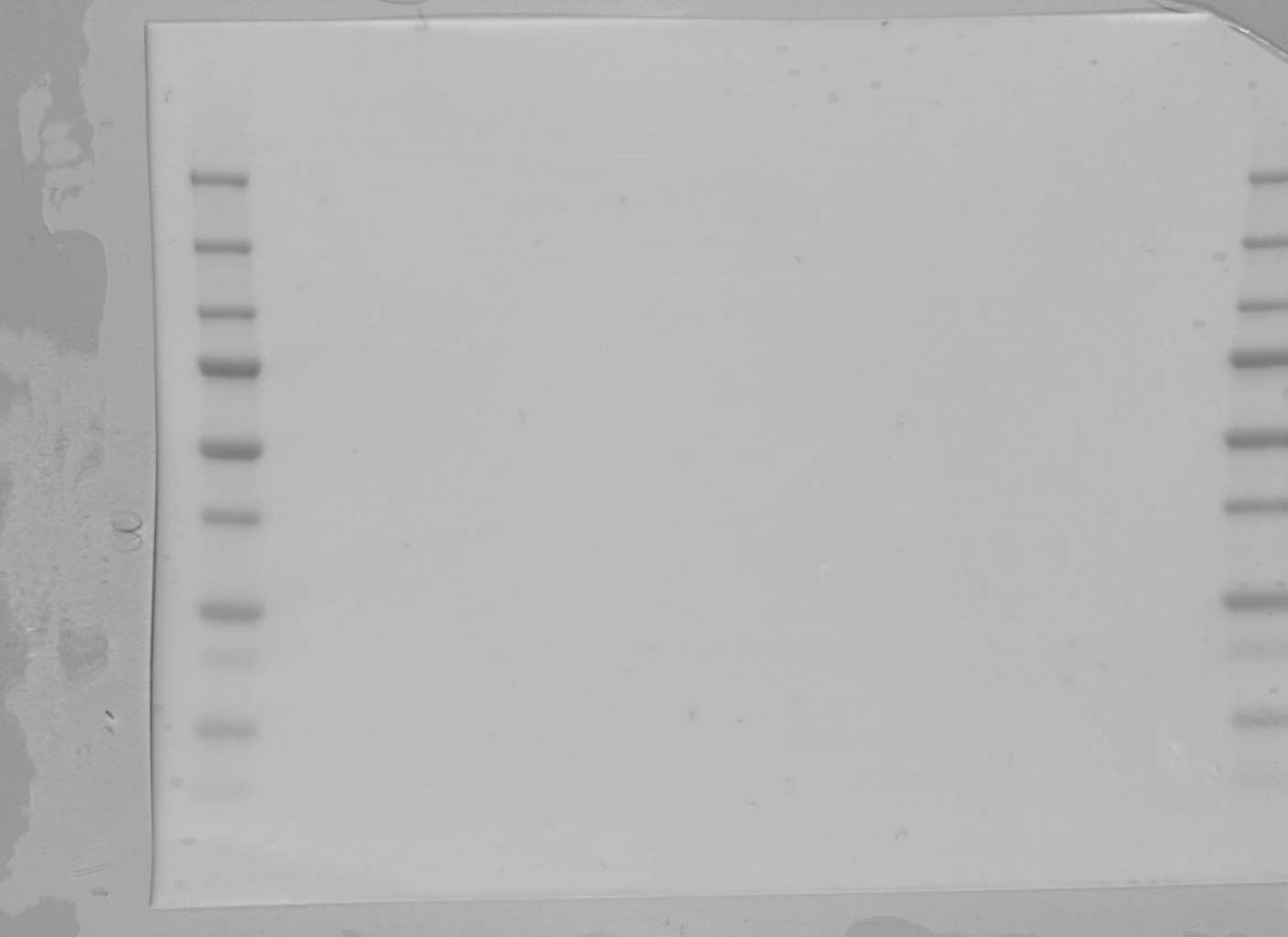

Supplement: Figure 13—figure supplement 1—source data 2. [file elife-80949-fig13-figsupp1-data2.zip › Figure 13-supplement 1 source data 2/PDE3A/PDE3A/DR Blot2 PDE3A WPP 2018.01.11_10.55.27_Ch-Marker.tif]

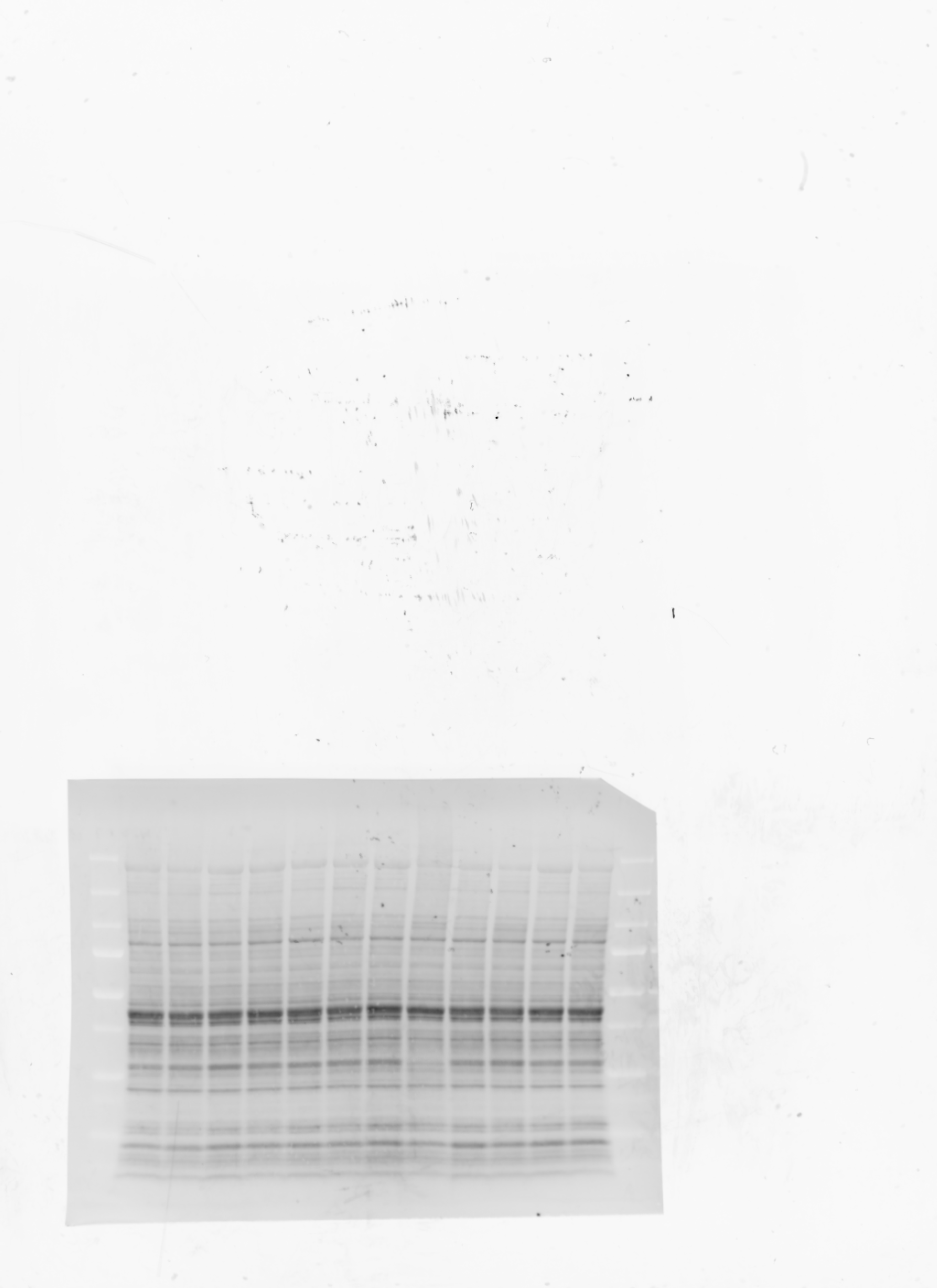

Supplement: Figure 13—figure supplement 1—source data 2. [file elife-80949-fig13-figsupp1-data2.zip › Figure 13-supplement 1 source data 2/PDE3A/Total Protein/DR Blot2 TotProt 2018.01.10_16.15.49_Fl-UV.tif]

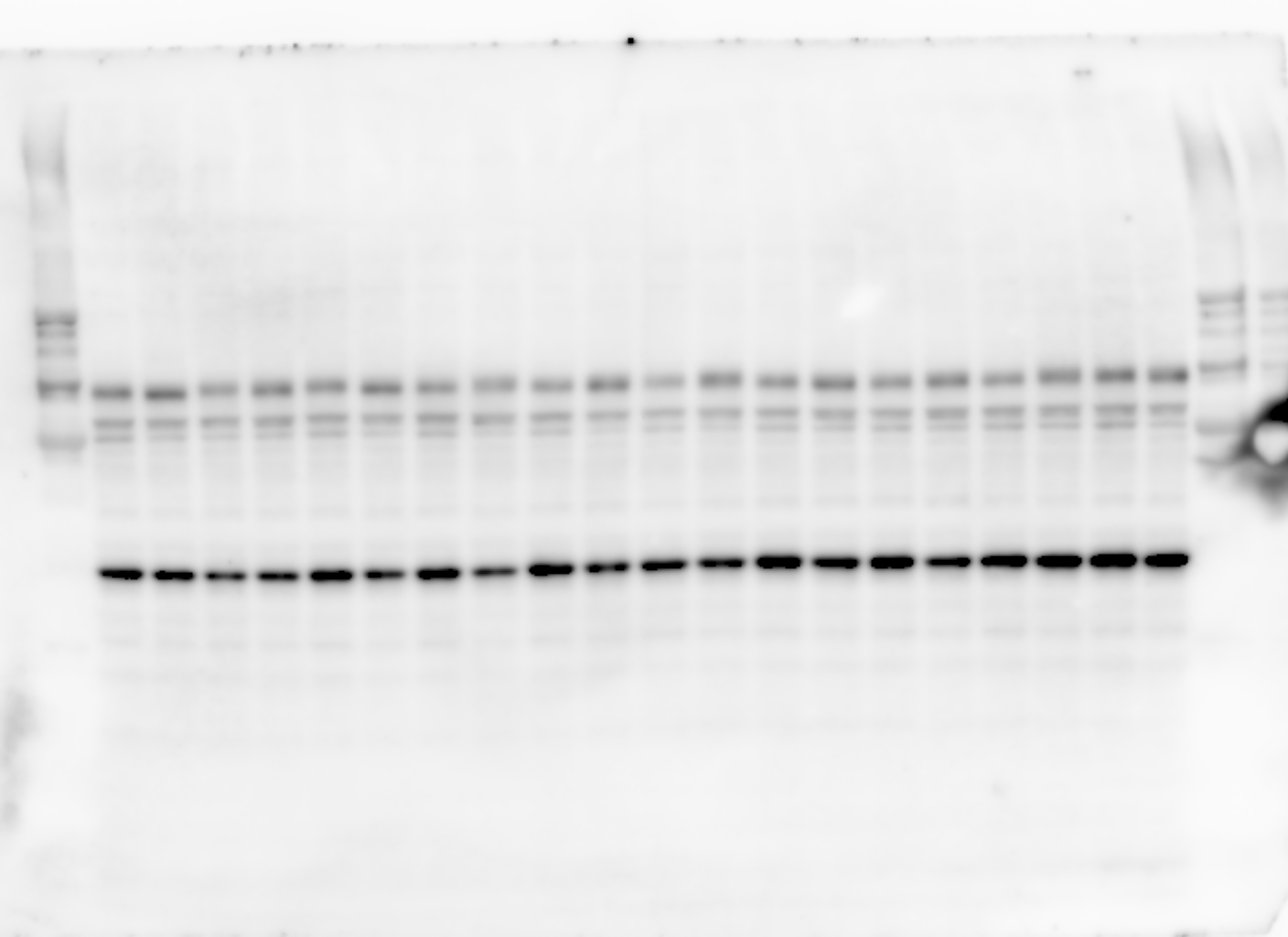

Supplement: Figure 13—figure supplement 1—source data 2. [file elife-80949-fig13-figsupp1-data2.zip › Figure 13-supplement 1 source data 2/PDE4A/PDE4A/DR PDE4A Blt8 WPP 2018.02.23_12.27.11_Ch.tif]

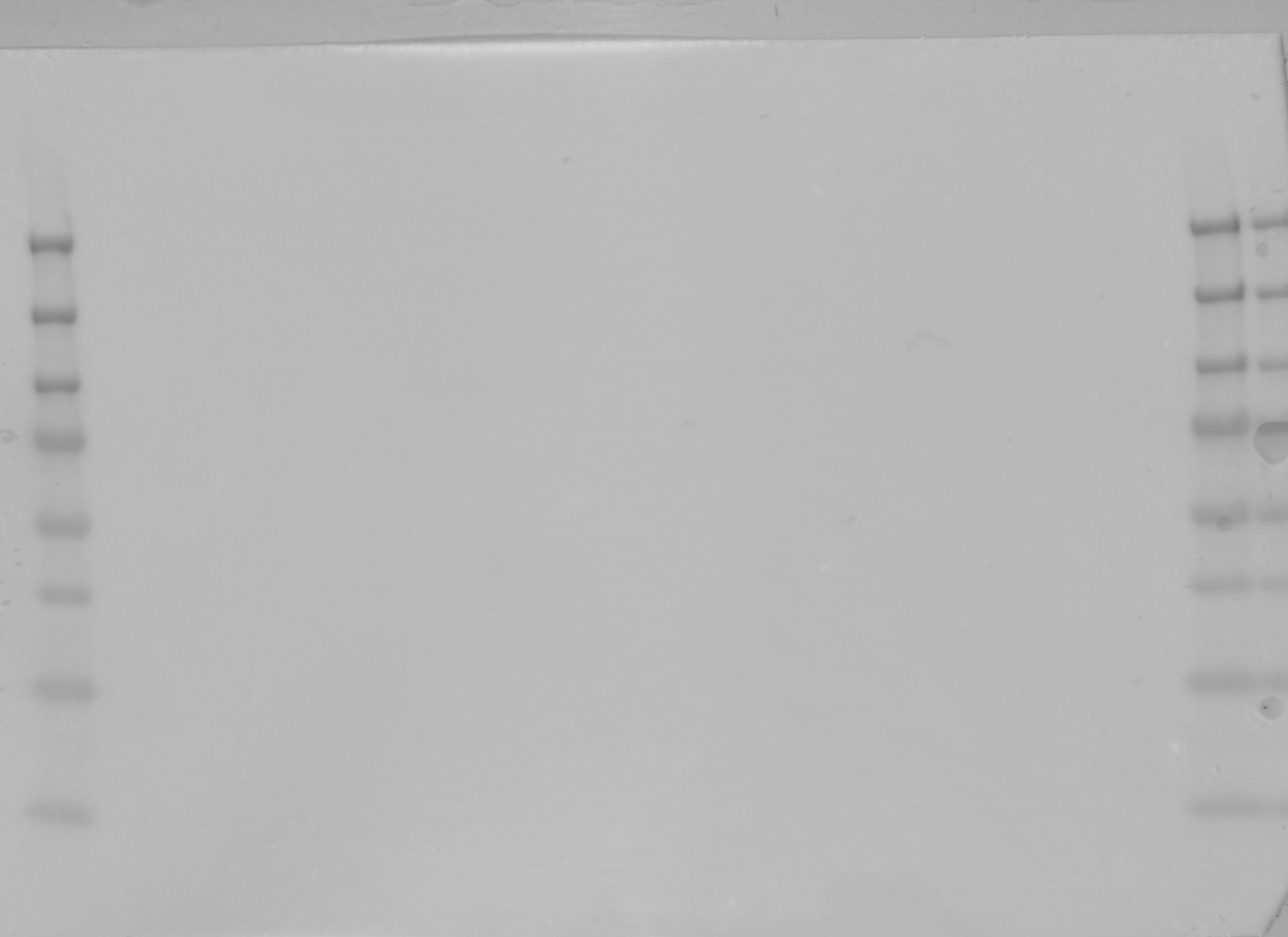

Supplement: Figure 13—figure supplement 1—source data 2. [file elife-80949-fig13-figsupp1-data2.zip › Figure 13-supplement 1 source data 2/PDE4A/PDE4A/DR PDE4A Blt8 WPP 2018.02.23_12.27.11_Ch-Marker.tif]

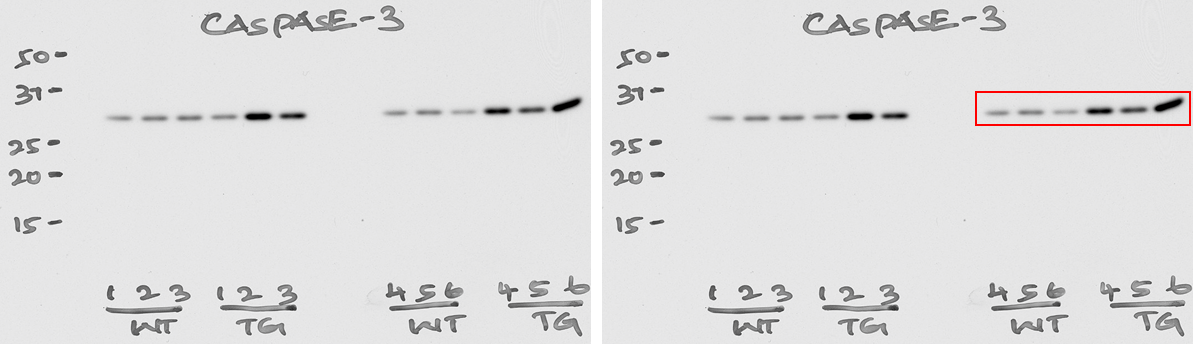

Supplement: Figure 13—figure supplement 2—source data 1. [file elife-80949-fig13-figsupp2-data1.zip › Figure 13-supplement 2 source data/Caspase3.tif]

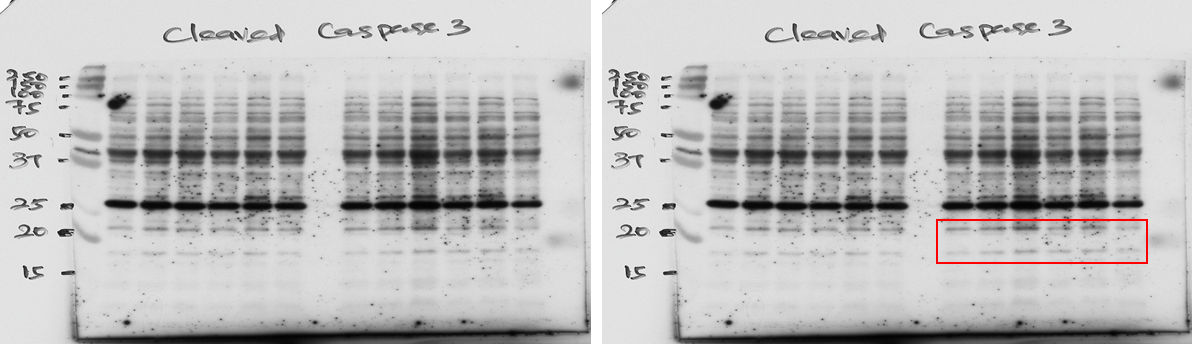

Supplement: Figure 13—figure supplement 2—source data 1. [file elife-80949-fig13-figsupp2-data1.zip › Figure 13-supplement 2 source data/Cleaved Caspase3.tif]

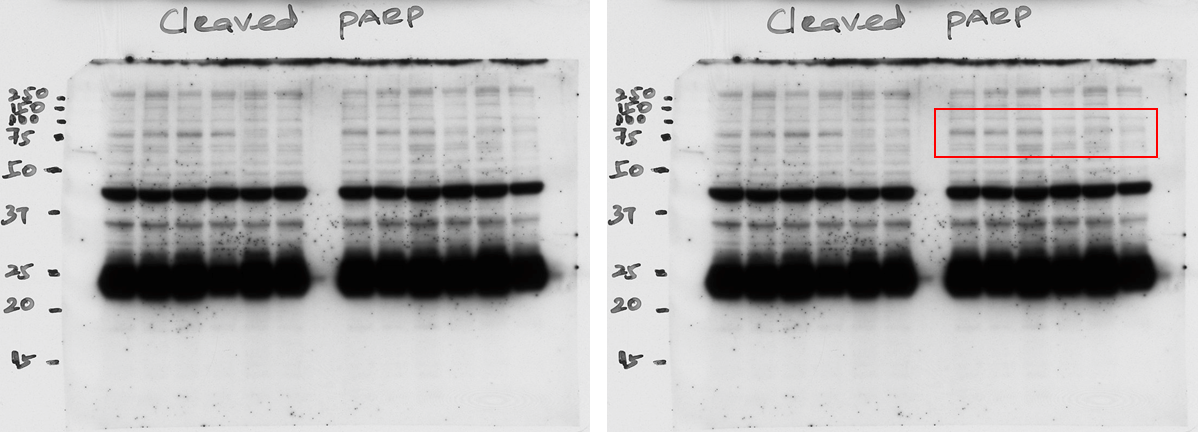

Supplement: Figure 13—figure supplement 2—source data 1. [file elife-80949-fig13-figsupp2-data1.zip › Figure 13-supplement 2 source data/Cleaved PARP.tif]

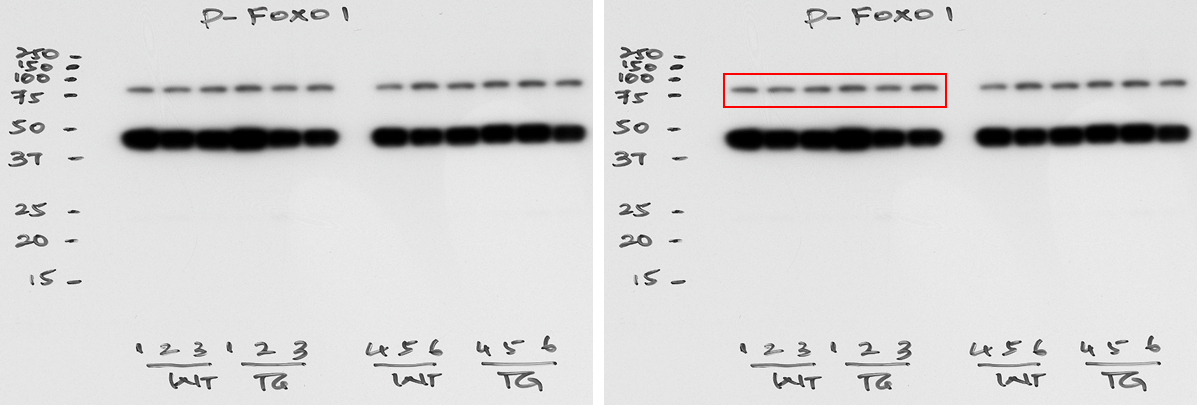

Supplement: Figure 13—figure supplement 2—source data 1. [file elife-80949-fig13-figsupp2-data1.zip › Figure 13-supplement 2 source data/FoxO1_Ser256.tif]

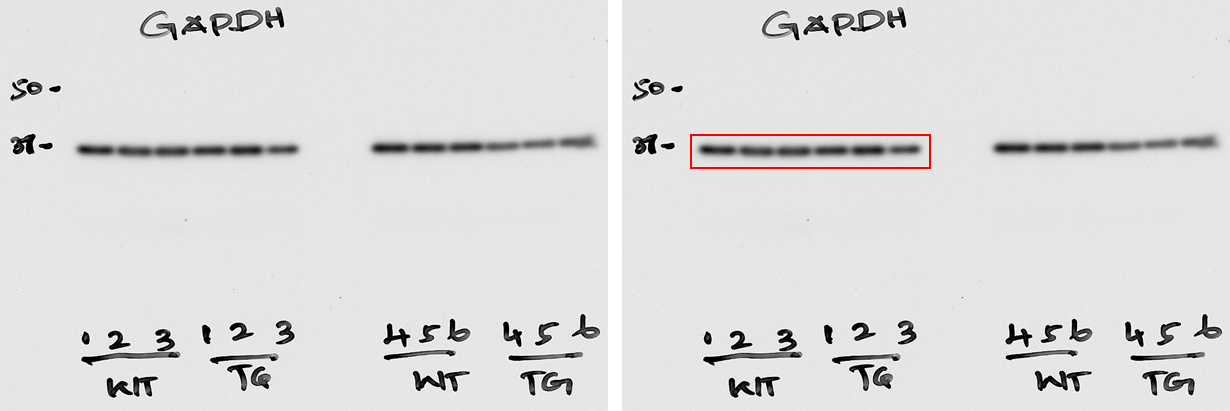

Supplement: Figure 13—figure supplement 2—source data 1. [file elife-80949-fig13-figsupp2-data1.zip › Figure 13-supplement 2 source data/GAPDH_left top panel.tif]

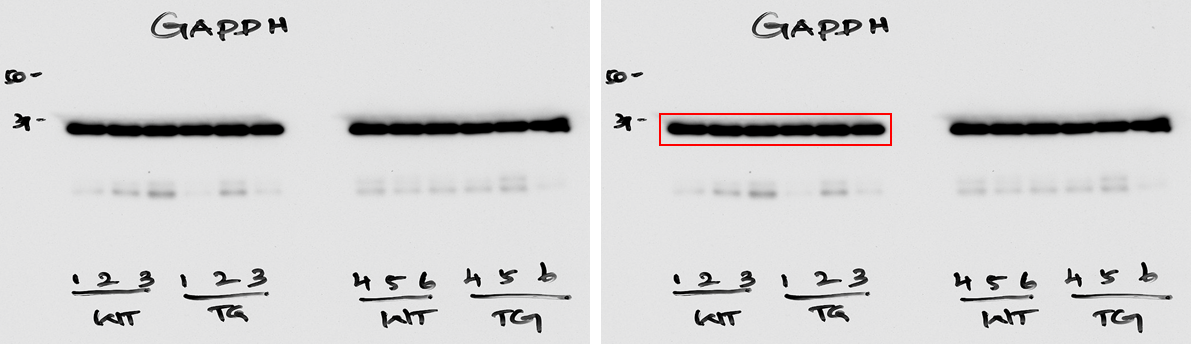

Supplement: Figure 13—figure supplement 2—source data 1. [file elife-80949-fig13-figsupp2-data1.zip › Figure 13-supplement 2 source data/GAPDH_left_bottom panel.tif]
