# Supplementary figures and images for: A remarkable adaptive paradigm of heart performance and protection emerges in response to marked cardiac-specific overexpression of ADCY8 (part 3 of 3)
Source: eLife. 2022 Dec 14;11:e80949. doi: 10.7554/eLife.80949 (PMC9822292; doi:10.7554/eLife.80949)

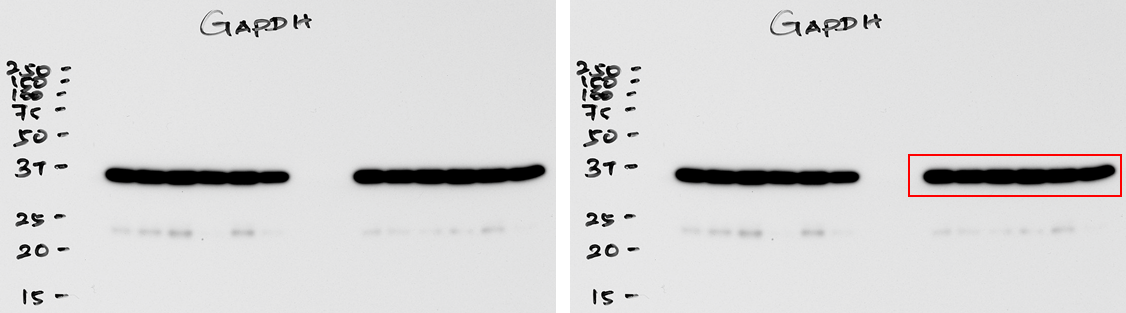

Supplement: Figure 13—figure supplement 2—source data 1. [file elife-80949-fig13-figsupp2-data1.zip › Figure 13-supplement 2 source data/GAPDH_right bottom panel.tif]

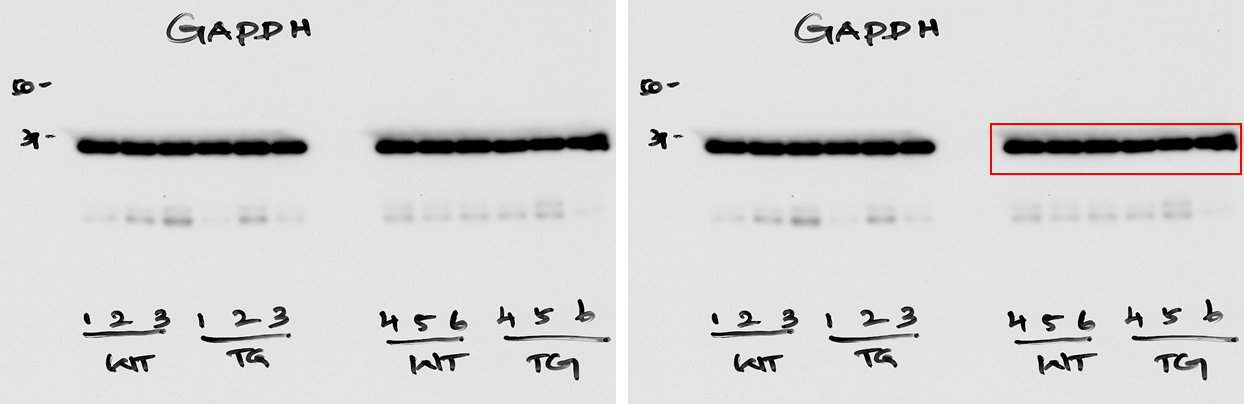

Supplement: Figure 13—figure supplement 2—source data 1. [file elife-80949-fig13-figsupp2-data1.zip › Figure 13-supplement 2 source data/GAPDH_right top panel.tif]

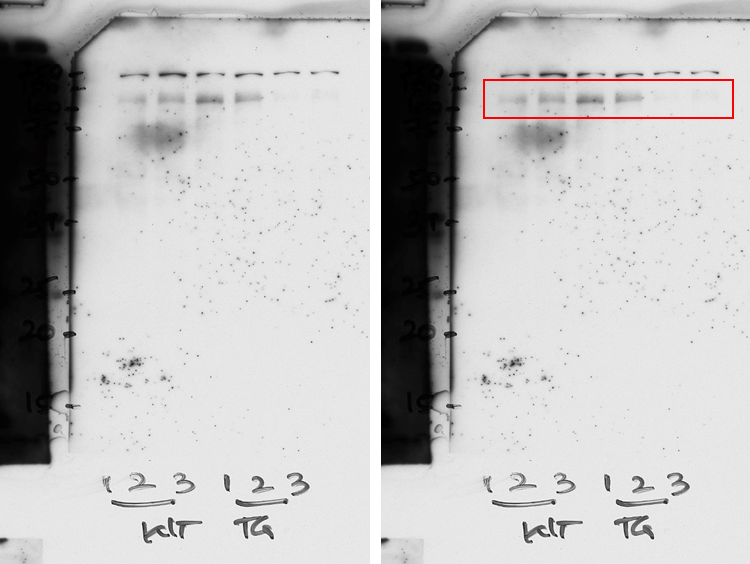

Supplement: Figure 13—figure supplement 2—source data 1. [file elife-80949-fig13-figsupp2-data1.zip › Figure 13-supplement 2 source data/HIF_1alpha.tif]

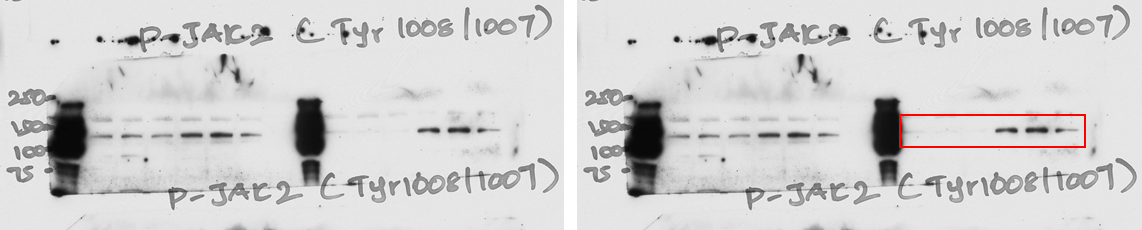

Supplement: Figure 13—figure supplement 2—source data 1. [file elife-80949-fig13-figsupp2-data1.zip › Figure 13-supplement 2 source data/JAK2_Tyr1007_1008.tif]

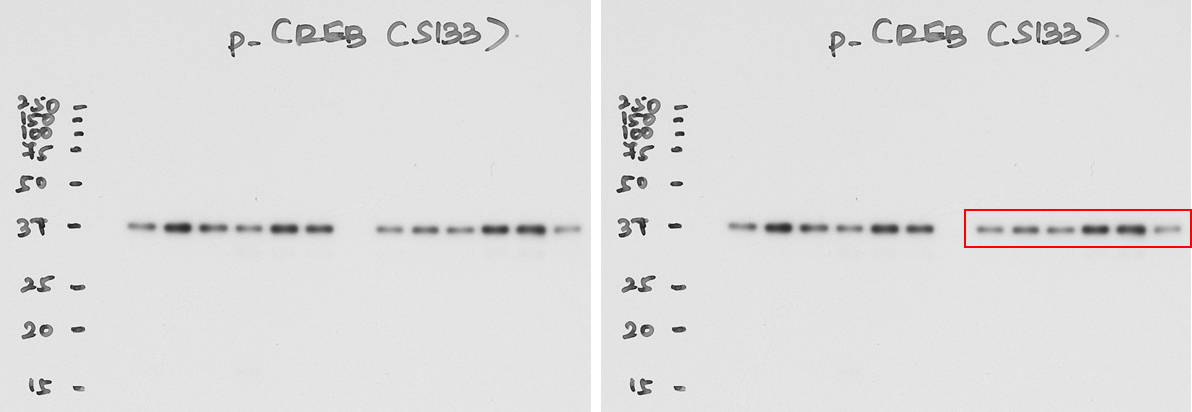

Supplement: Figure 13—figure supplement 2—source data 1. [file elife-80949-fig13-figsupp2-data1.zip › Figure 13-supplement 2 source data/p_CREB_Ser133.tif]

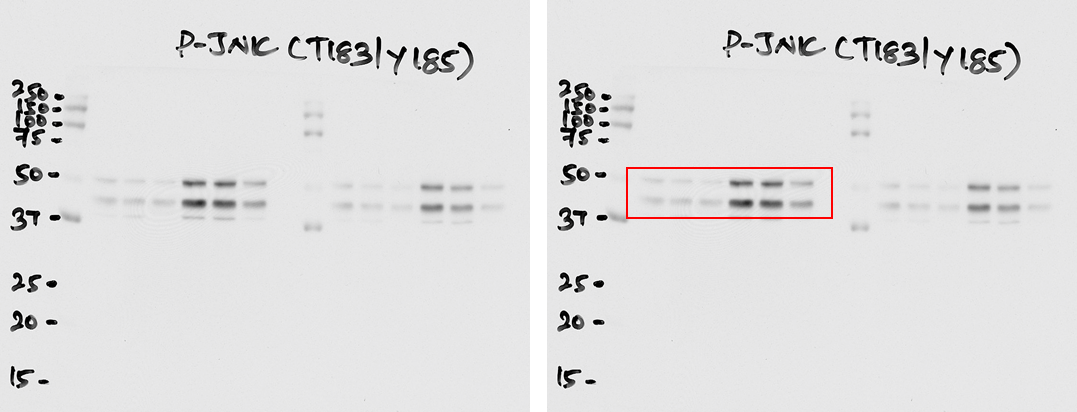

Supplement: Figure 13—figure supplement 2—source data 1. [file elife-80949-fig13-figsupp2-data1.zip › Figure 13-supplement 2 source data/p_JNK_Thr183_Tyr185.tif]

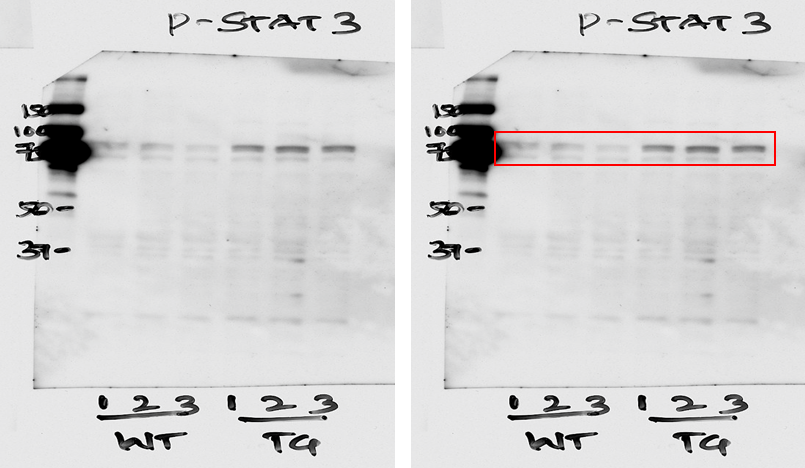

Supplement: Figure 13—figure supplement 2—source data 1. [file elife-80949-fig13-figsupp2-data1.zip › Figure 13-supplement 2 source data/p_STAT3_Tyr705.tif]

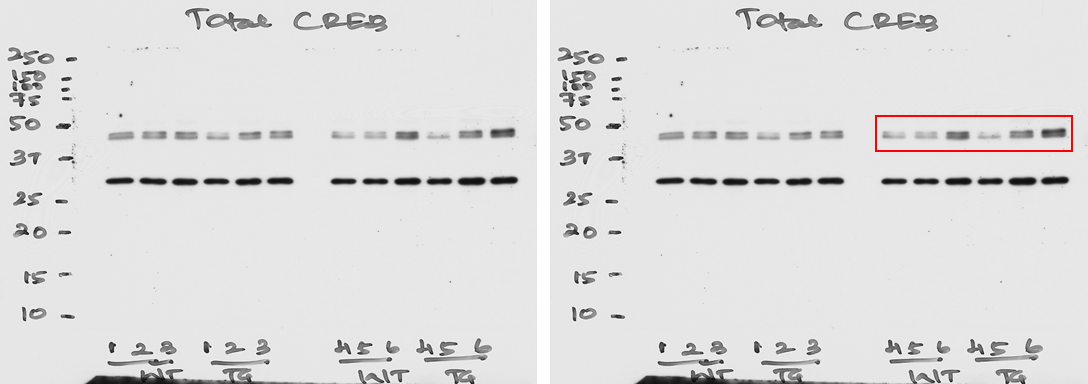

Supplement: Figure 13—figure supplement 2—source data 1. [file elife-80949-fig13-figsupp2-data1.zip › Figure 13-supplement 2 source data/Total CREB.tif]

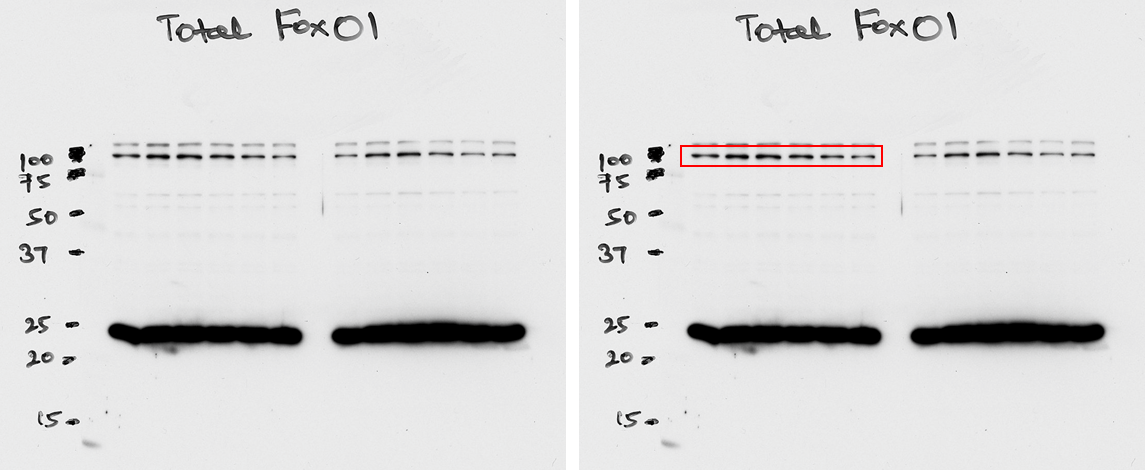

Supplement: Figure 13—figure supplement 2—source data 1. [file elife-80949-fig13-figsupp2-data1.zip › Figure 13-supplement 2 source data/Total FoxO1.tif]

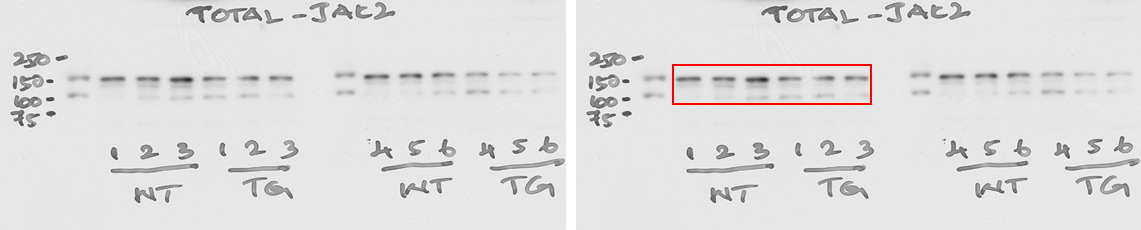

Supplement: Figure 13—figure supplement 2—source data 1. [file elife-80949-fig13-figsupp2-data1.zip › Figure 13-supplement 2 source data/Total JAK2.tif]

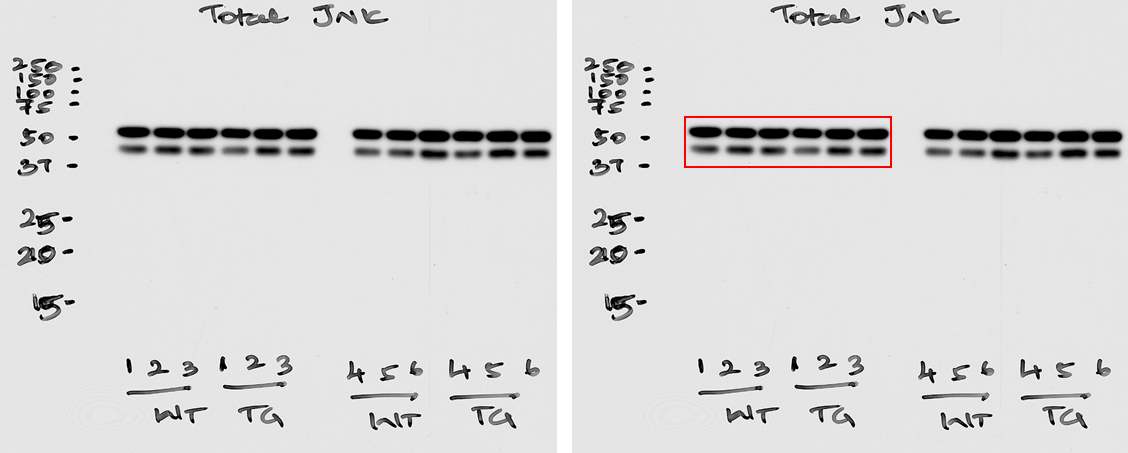

Supplement: Figure 13—figure supplement 2—source data 1. [file elife-80949-fig13-figsupp2-data1.zip › Figure 13-supplement 2 source data/Total JNK.tif]

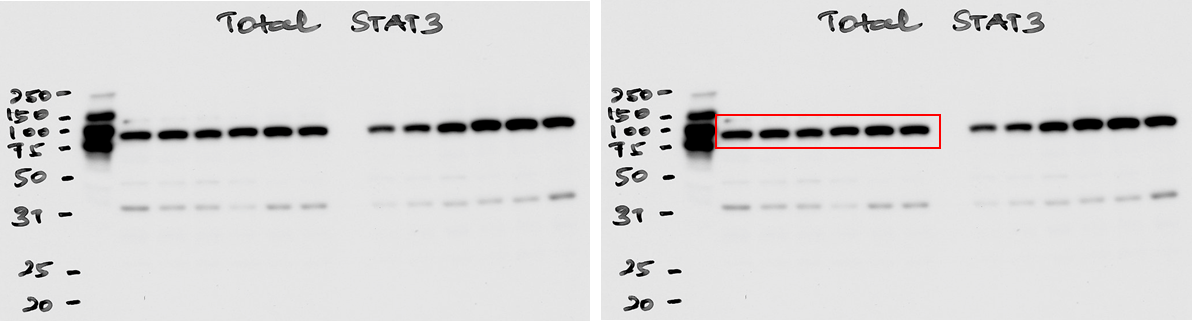

Supplement: Figure 13—figure supplement 2—source data 1. [file elife-80949-fig13-figsupp2-data1.zip › Figure 13-supplement 2 source data/Total STAT3.tif]

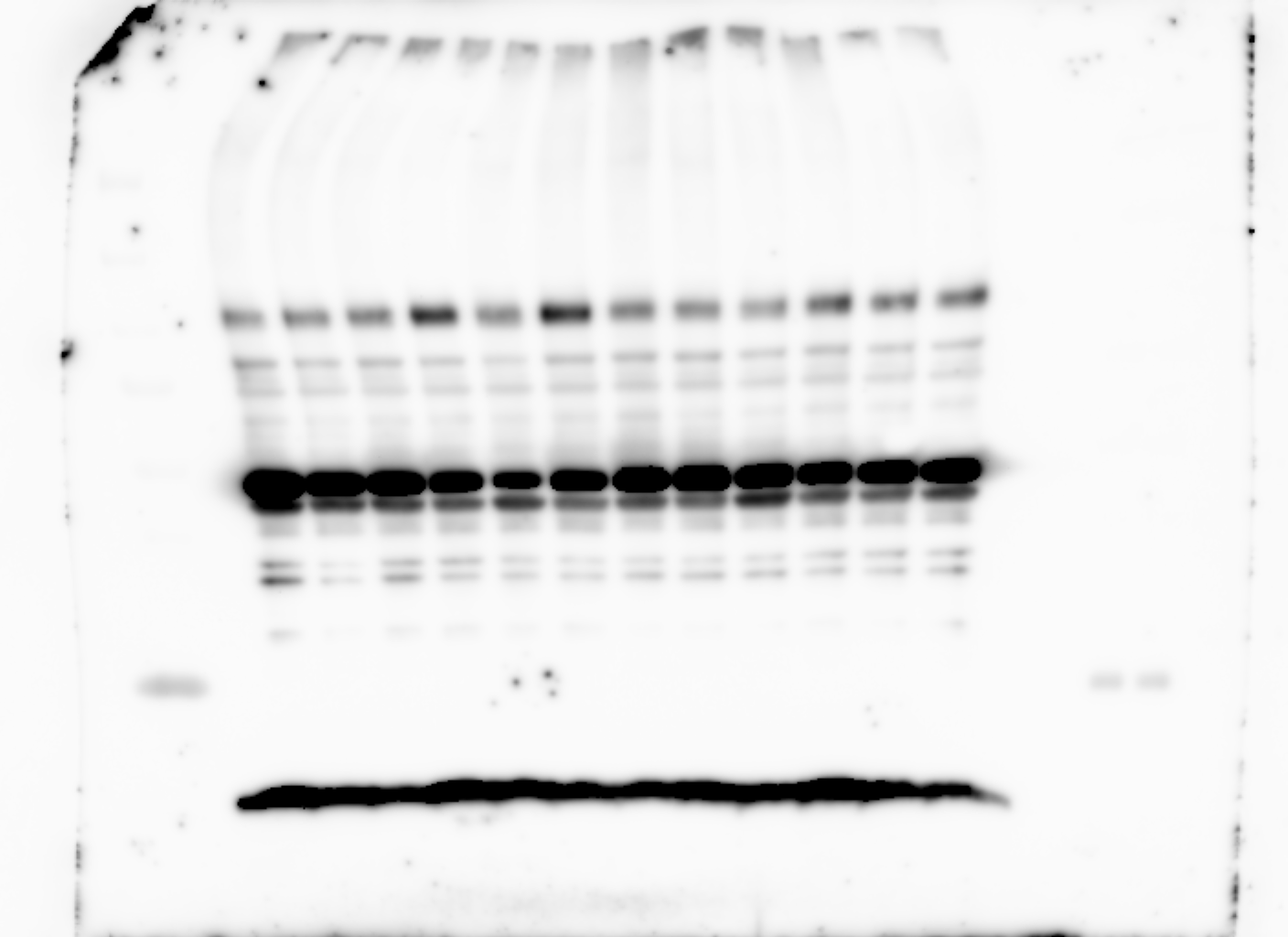

Supplement: Figure 13—figure supplement 3—source data 1. [file elife-80949-fig13-figsupp3-data1.zip › Figure 13-supplement 3 source data/ACE2/ACE2/WO_ACE2_BltW5 7min 2021.07.09_12.38.02_Ch.tif]

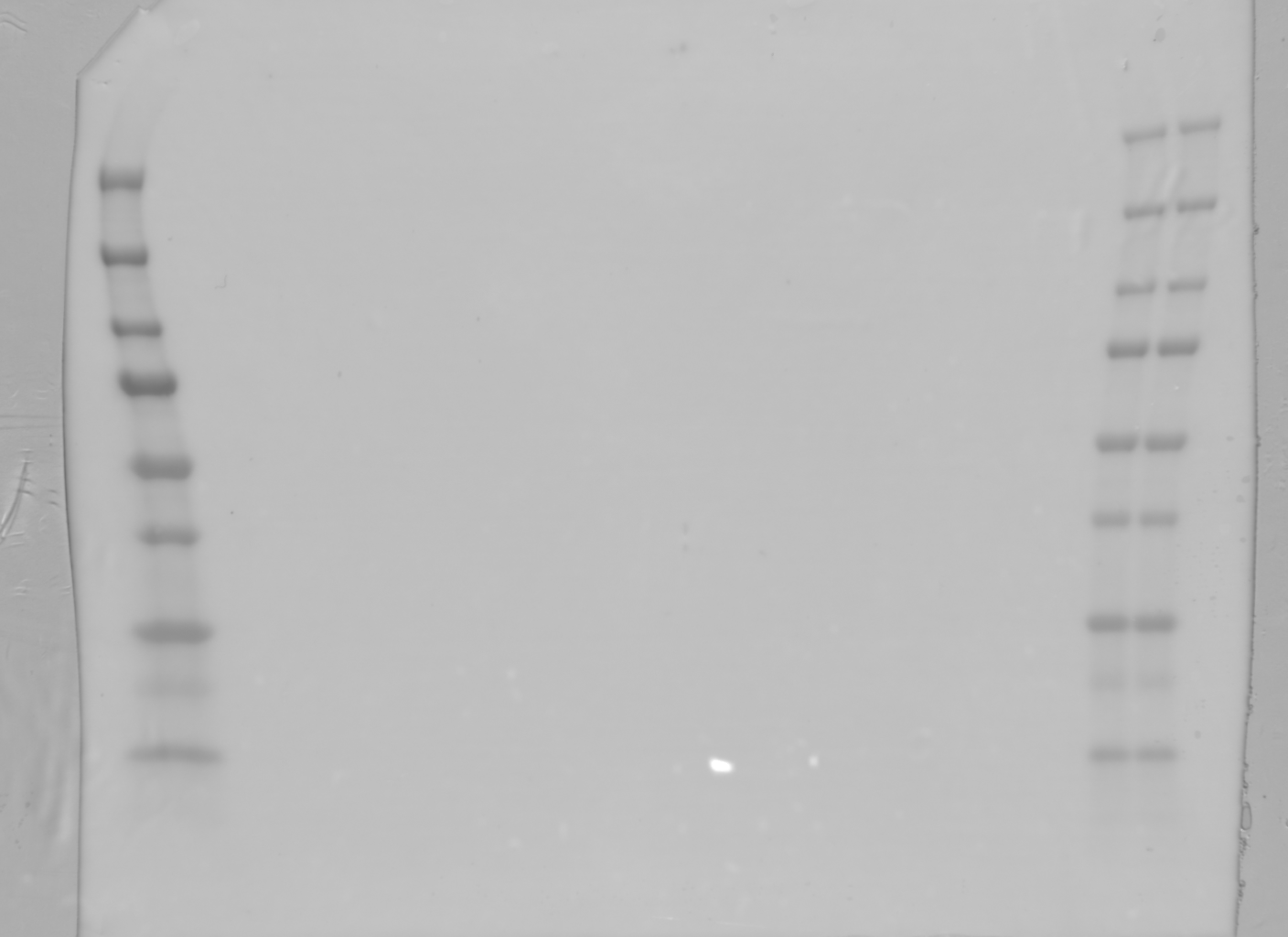

Supplement: Figure 13—figure supplement 3—source data 1. [file elife-80949-fig13-figsupp3-data1.zip › Figure 13-supplement 3 source data/ACE2/ACE2/WO_ACE2_BltW5 7min 2021.07.09_12.38.02_Ch-Marker.tif]

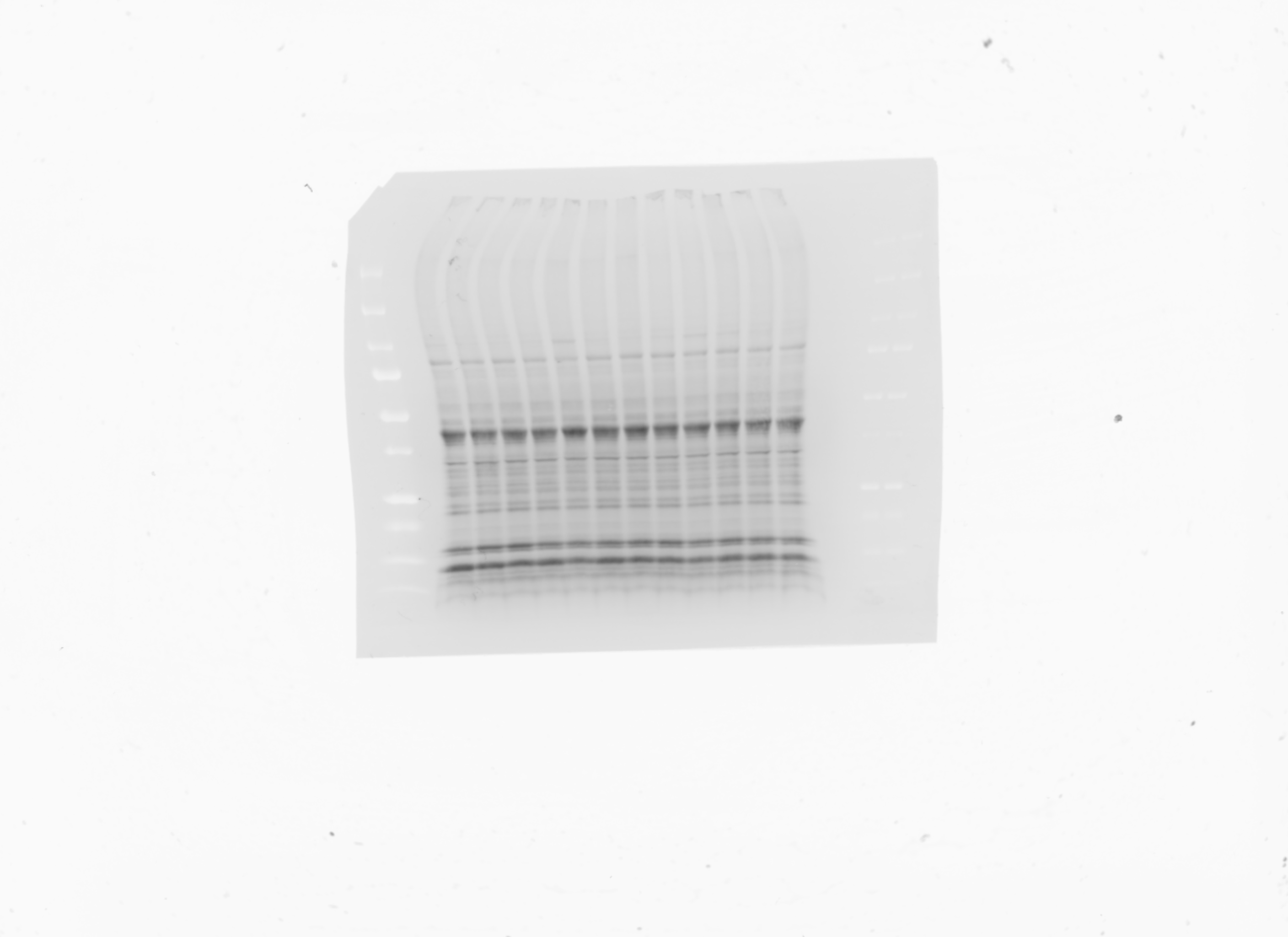

Supplement: Figure 13—figure supplement 3—source data 1. [file elife-80949-fig13-figsupp3-data1.zip › Figure 13-supplement 3 source data/ACE2/Total Protein/WO_T.Prot.Blt W5 2021.06.23_15.26.30_Fl-UV.tif]

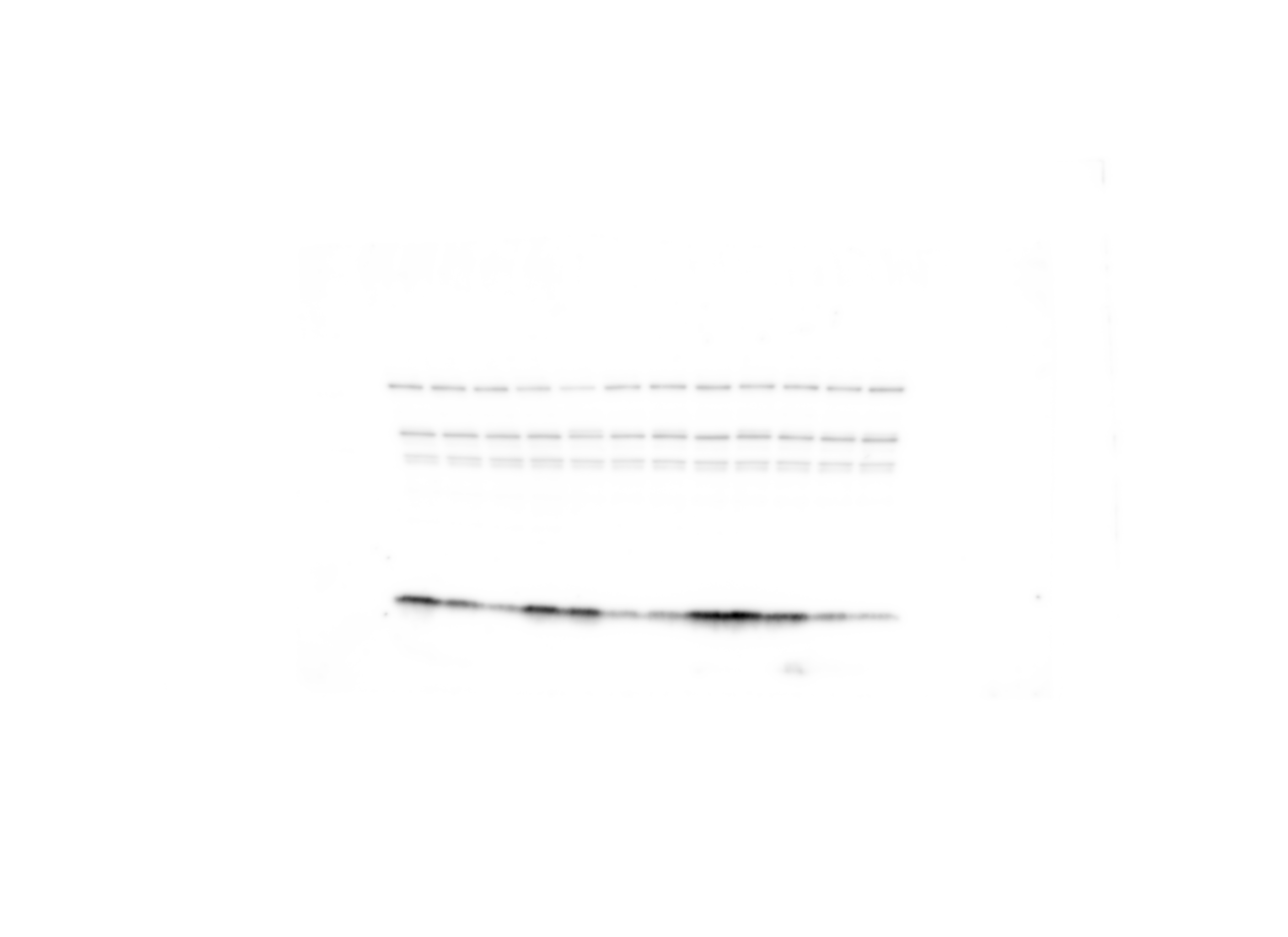

Supplement: Figure 13—figure supplement 3—source data 1. [file elife-80949-fig13-figsupp3-data1.zip › Figure 13-supplement 3 source data/AGTR1/AGTR1/WO_Agtr1_BltW6 3min 2021.09.01_11.07.43_Ch.tif]

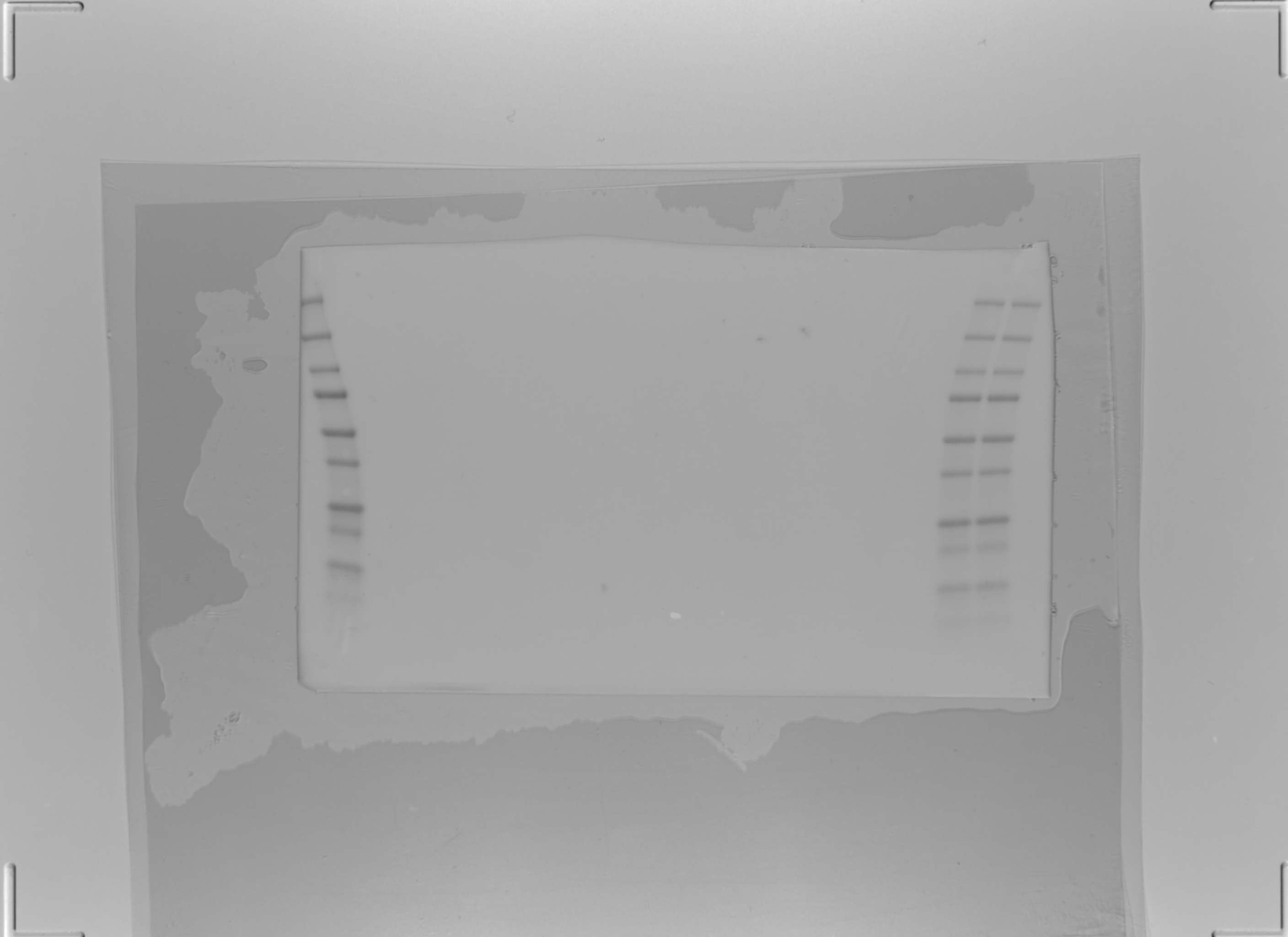

Supplement: Figure 13—figure supplement 3—source data 1. [file elife-80949-fig13-figsupp3-data1.zip › Figure 13-supplement 3 source data/AGTR1/AGTR1/WO_Agtr1_BltW6 3min 2021.09.01_11.07.43_Ch-Marker.tif]

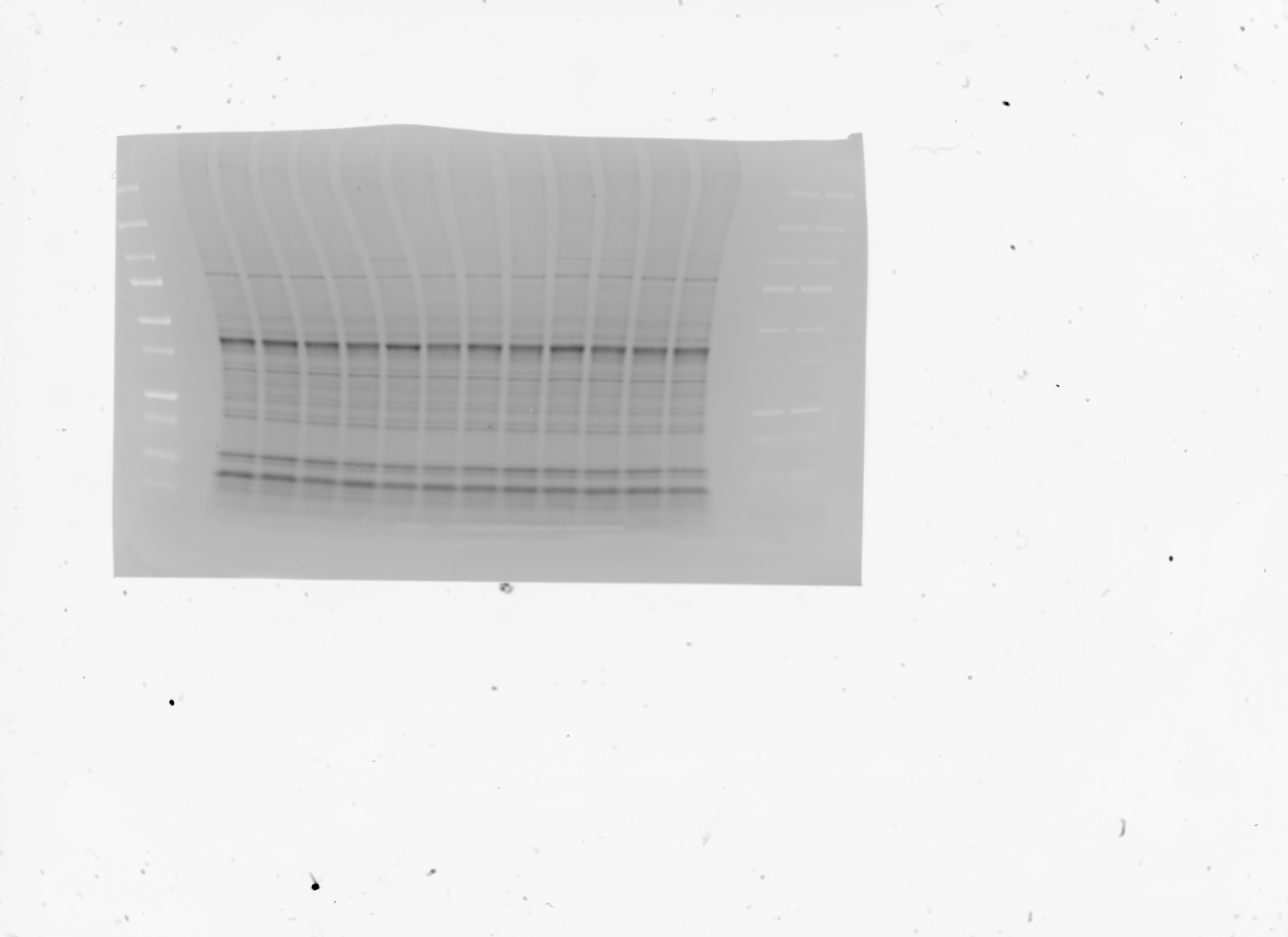

Supplement: Figure 13—figure supplement 3—source data 1. [file elife-80949-fig13-figsupp3-data1.zip › Figure 13-supplement 3 source data/AGTR1/Total Protein/WO_T.Prot_BltW6 2021.08.31_13.26.52_Fl-UV.tif]

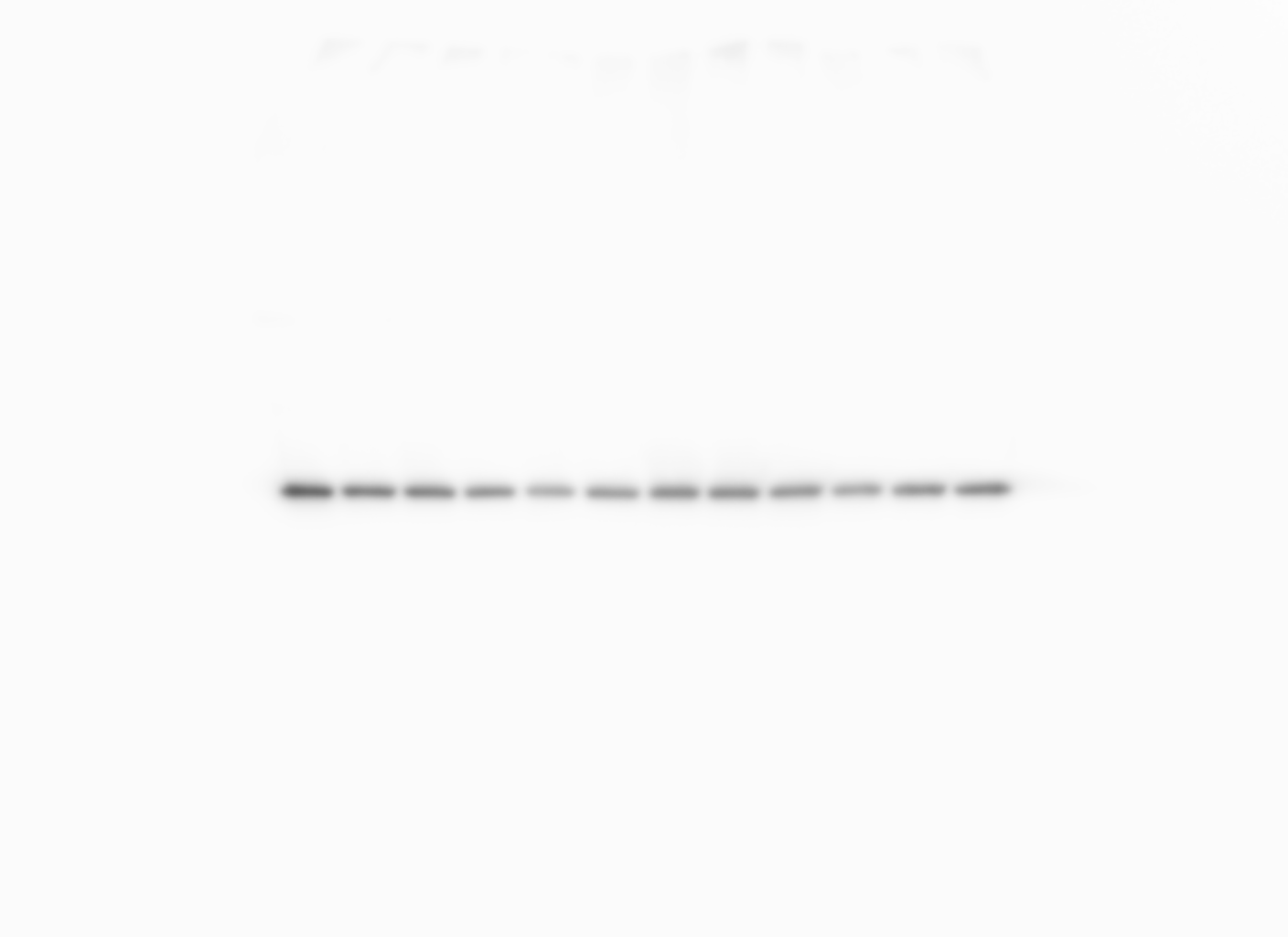

Supplement: Figure 13—figure supplement 3—source data 1. [file elife-80949-fig13-figsupp3-data1.zip › Figure 13-supplement 3 source data/AGTR2/AGTR2/WO_Agtr2_BltW5 1min 2021.06.28_15.58.37_Ch.tif]

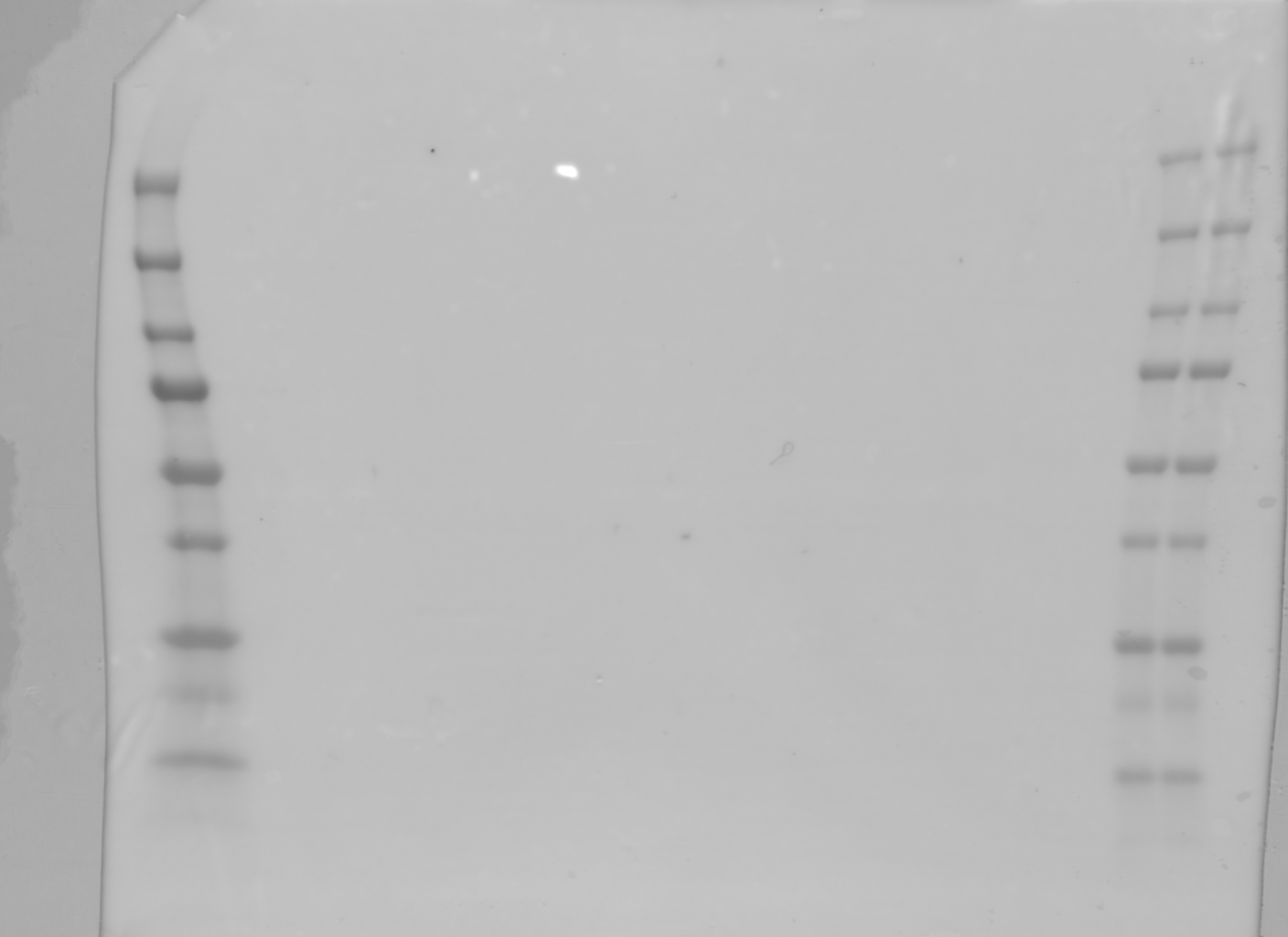

Supplement: Figure 13—figure supplement 3—source data 1. [file elife-80949-fig13-figsupp3-data1.zip › Figure 13-supplement 3 source data/AGTR2/AGTR2/WO_Agtr2_BltW5 1min 2021.06.28_15.58.37_Ch-Marker.tif]

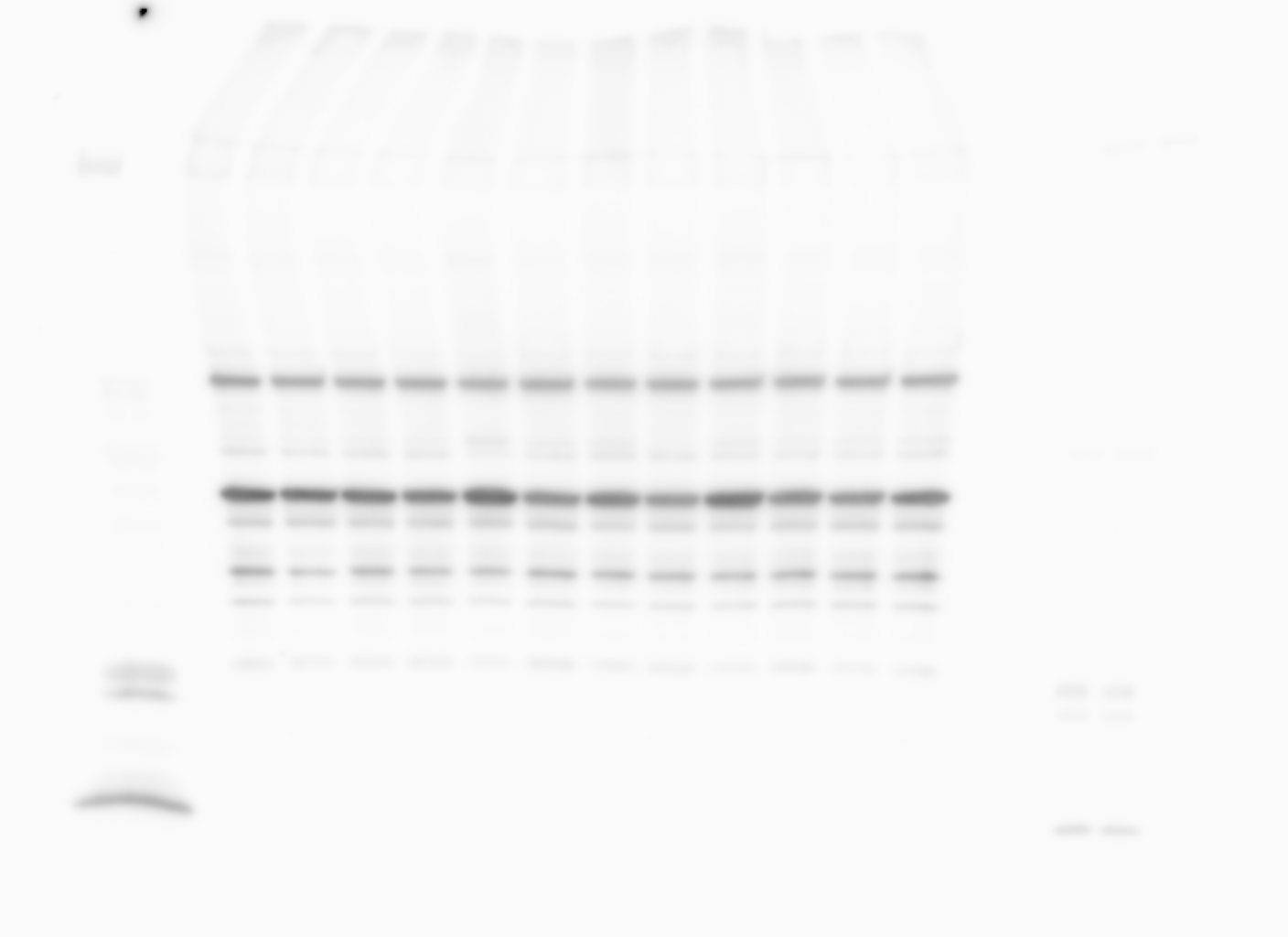

Supplement: Figure 13—figure supplement 3—source data 1. [file elife-80949-fig13-figsupp3-data1.zip › Figure 13-supplement 3 source data/MAS1/MAS1/WO_Mas1_BltW5 i 30s 2021.06.24_12.24.14_Ch.tif]

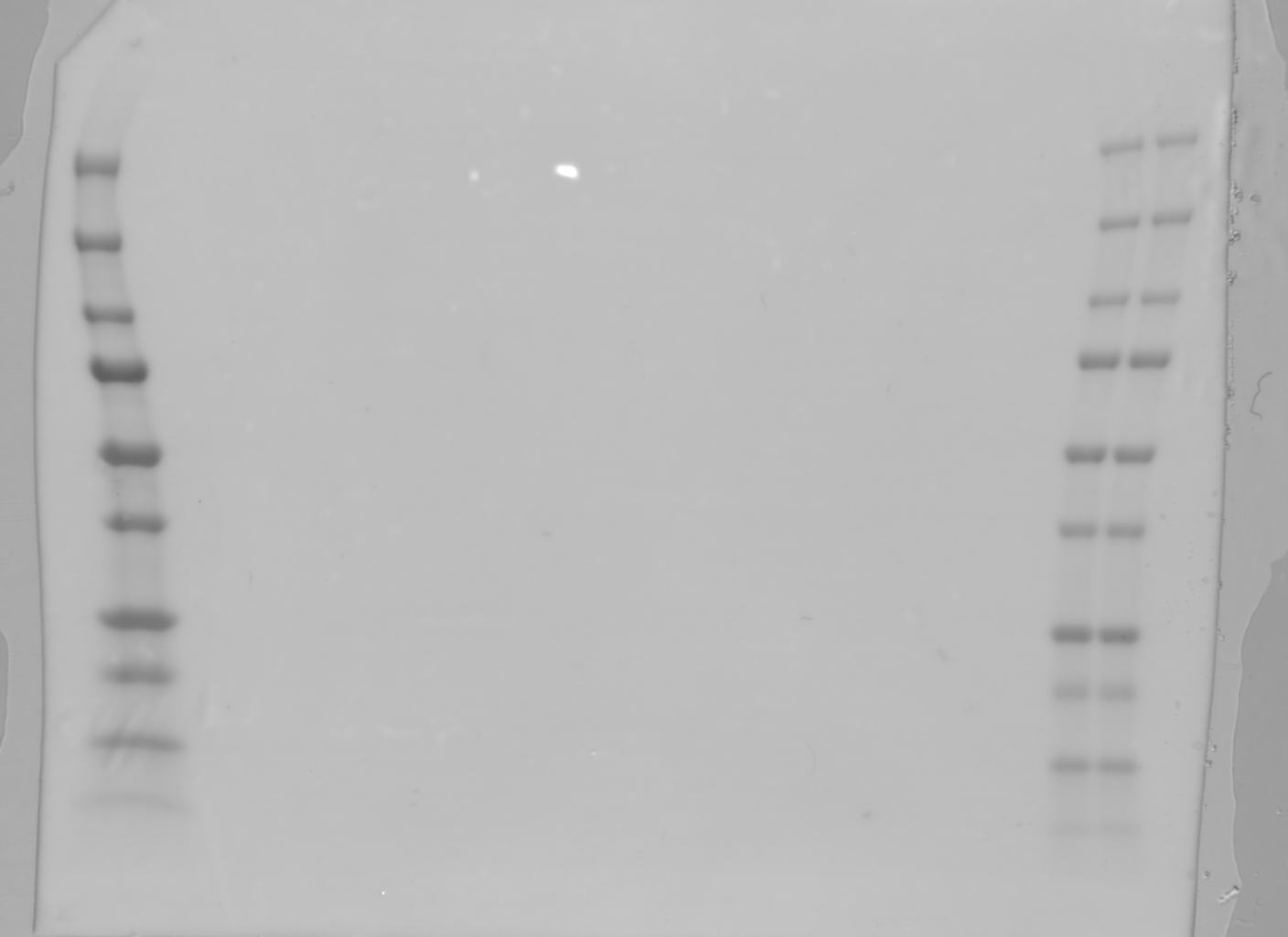

Supplement: Figure 13—figure supplement 3—source data 1. [file elife-80949-fig13-figsupp3-data1.zip › Figure 13-supplement 3 source data/MAS1/MAS1/WO_Mas1_BltW5 i 30s 2021.06.24_12.24.14_Ch-Marker.tif]

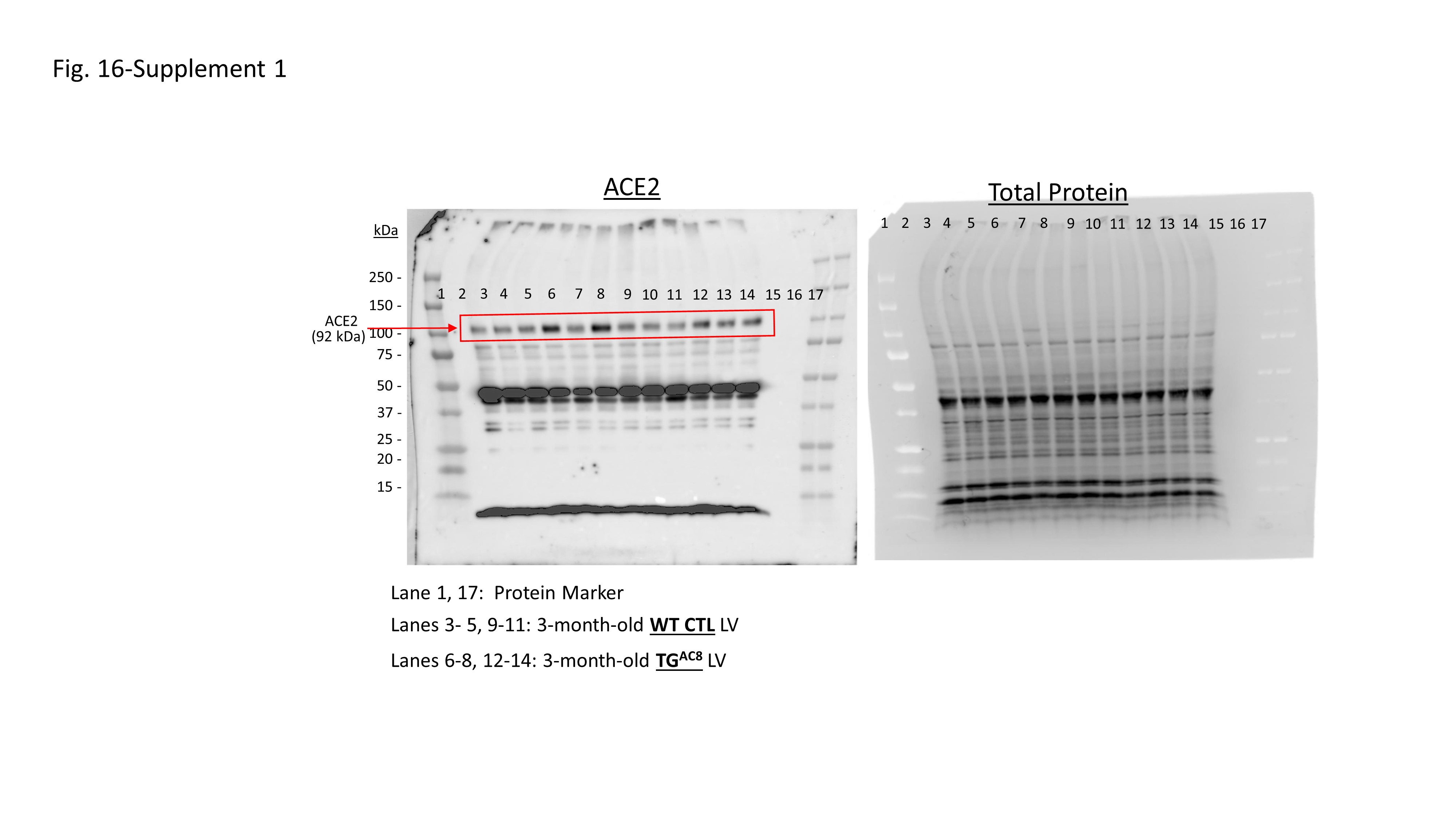

Supplement: Figure 13—figure supplement 3—source data 1. [file elife-80949-fig13-figsupp3-data1.zip › Figure 13-supplement 3 source data/Uncropped images/ACE2.JPG]

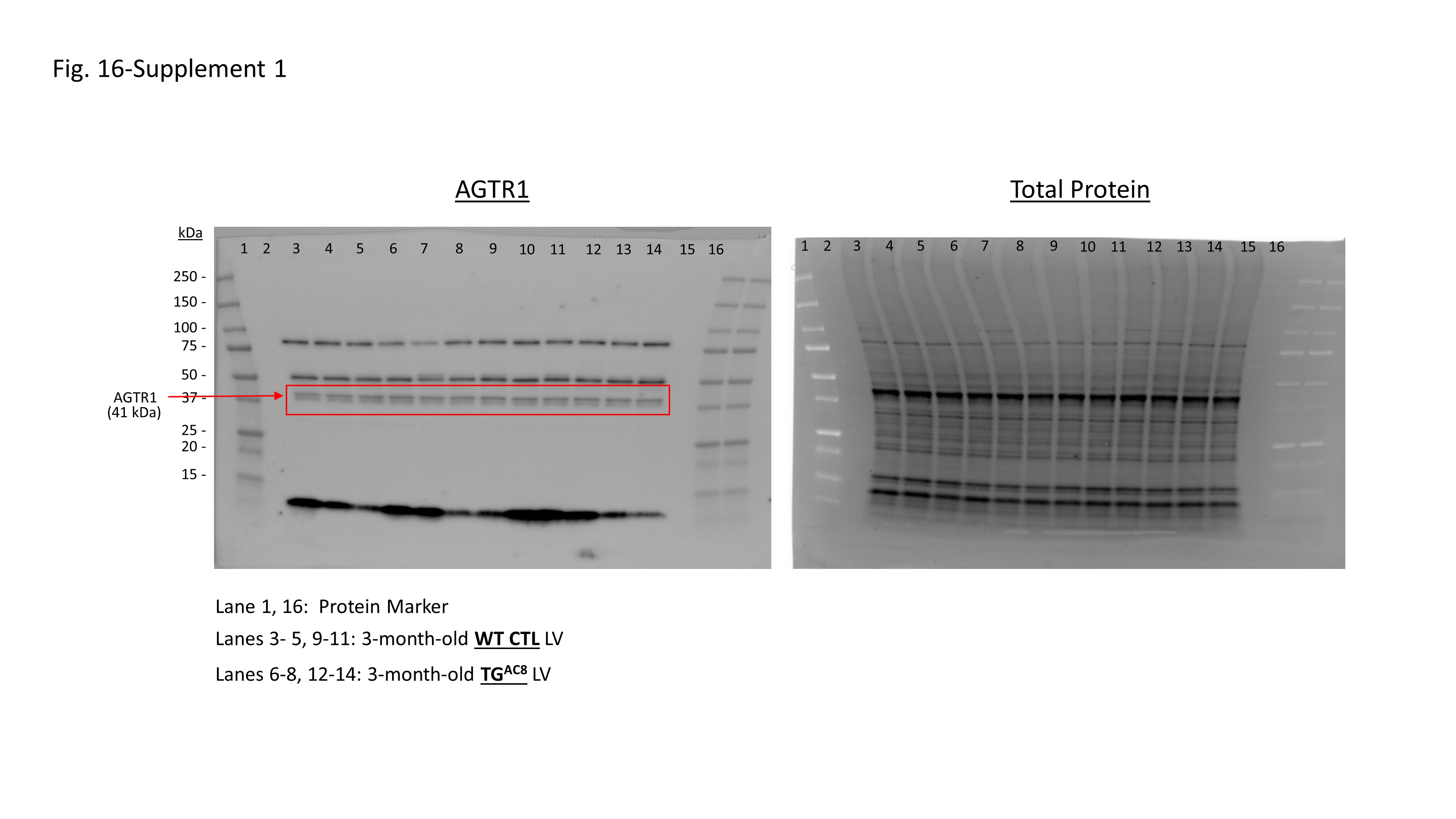

Supplement: Figure 13—figure supplement 3—source data 1. [file elife-80949-fig13-figsupp3-data1.zip › Figure 13-supplement 3 source data/Uncropped images/AGTR1.JPG]

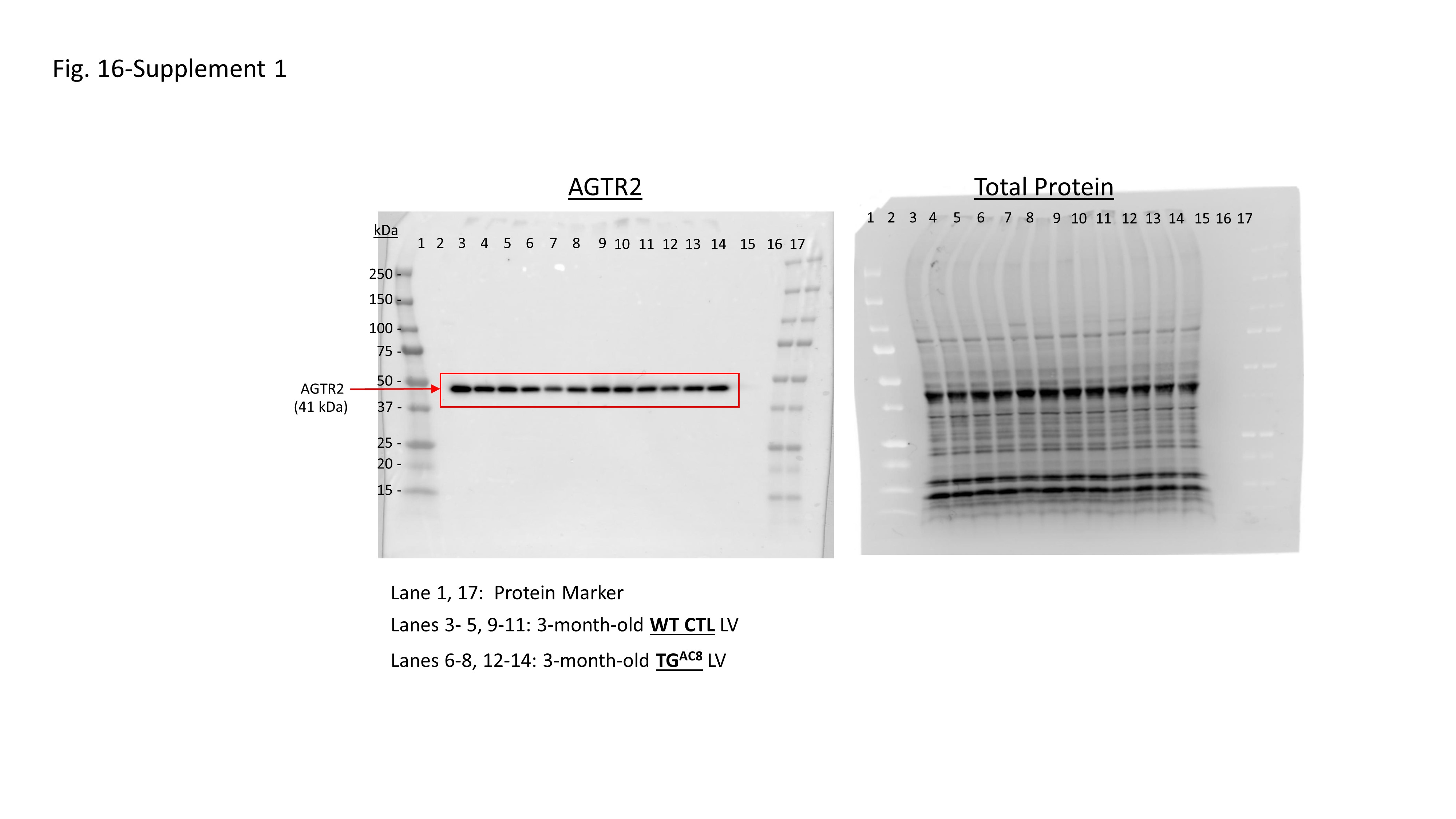

Supplement: Figure 13—figure supplement 3—source data 1. [file elife-80949-fig13-figsupp3-data1.zip › Figure 13-supplement 3 source data/Uncropped images/AGTR2.JPG]

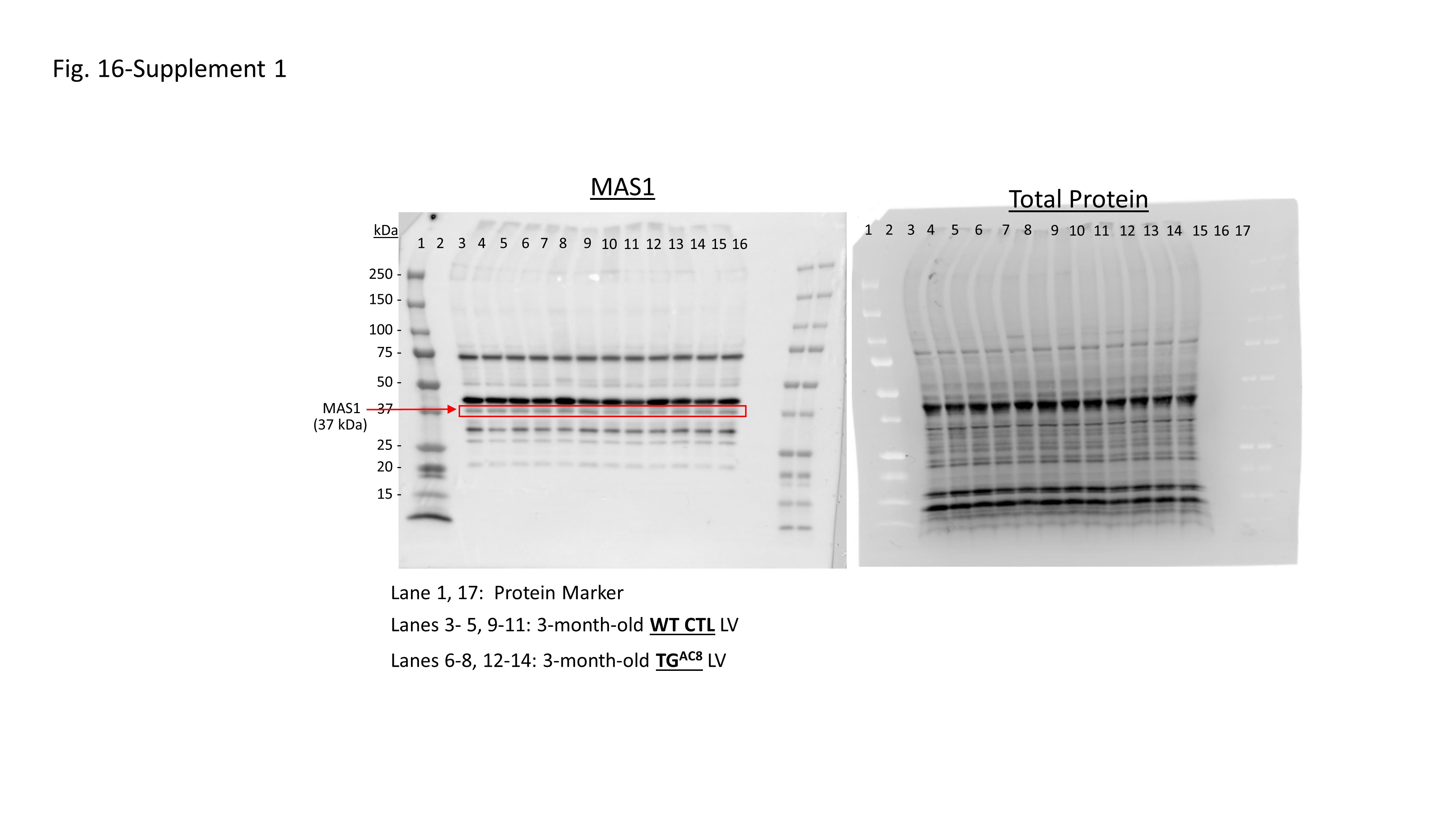

Supplement: Figure 13—figure supplement 3—source data 1. [file elife-80949-fig13-figsupp3-data1.zip › Figure 13-supplement 3 source data/Uncropped images/MAS1.JPG]

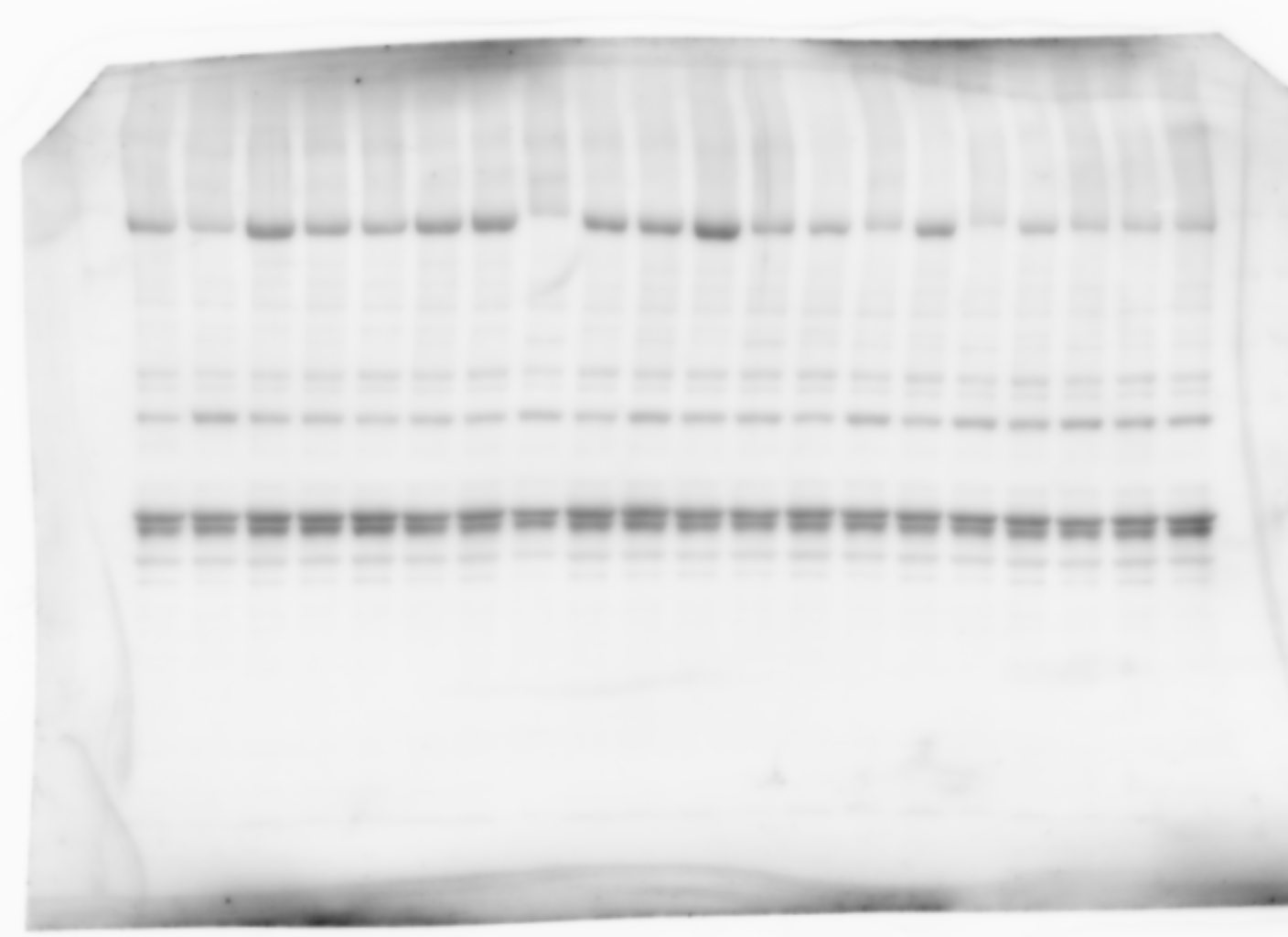

Supplement: Figure 13—figure supplement 4—source data 1. [file elife-80949-fig13-figsupp4-data1.zip › Figure 13-supplement 4 source data/CALR/CALR/DR CALR Rb Ab blot17 2018.04.12_11.06 (1).tif]

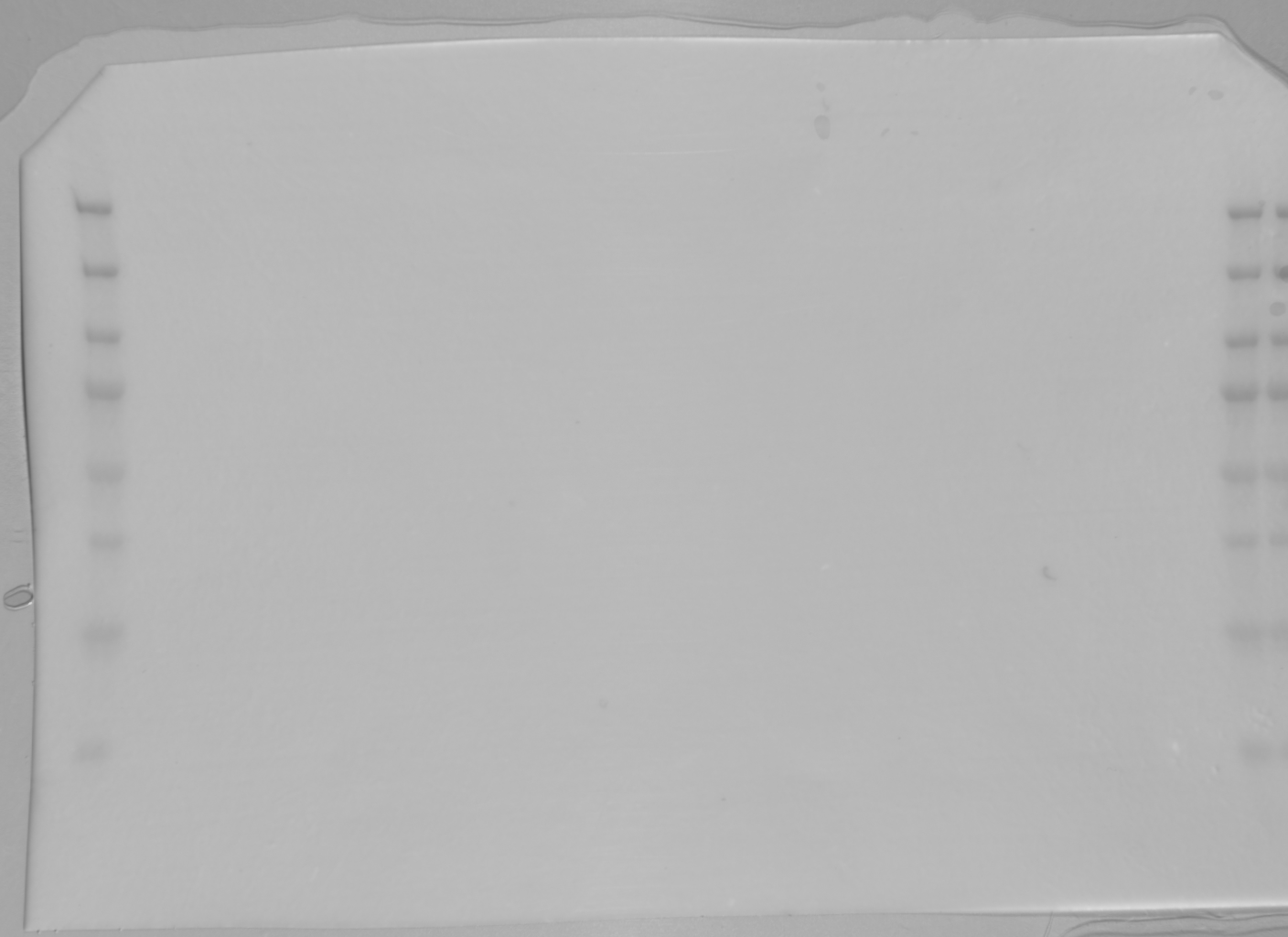

Supplement: Figure 13—figure supplement 4—source data 1. [file elife-80949-fig13-figsupp4-data1.zip › Figure 13-supplement 4 source data/CALR/CALR/DR CALR Rb Ab blot17 2018.04.12_11.06 (2).tif]

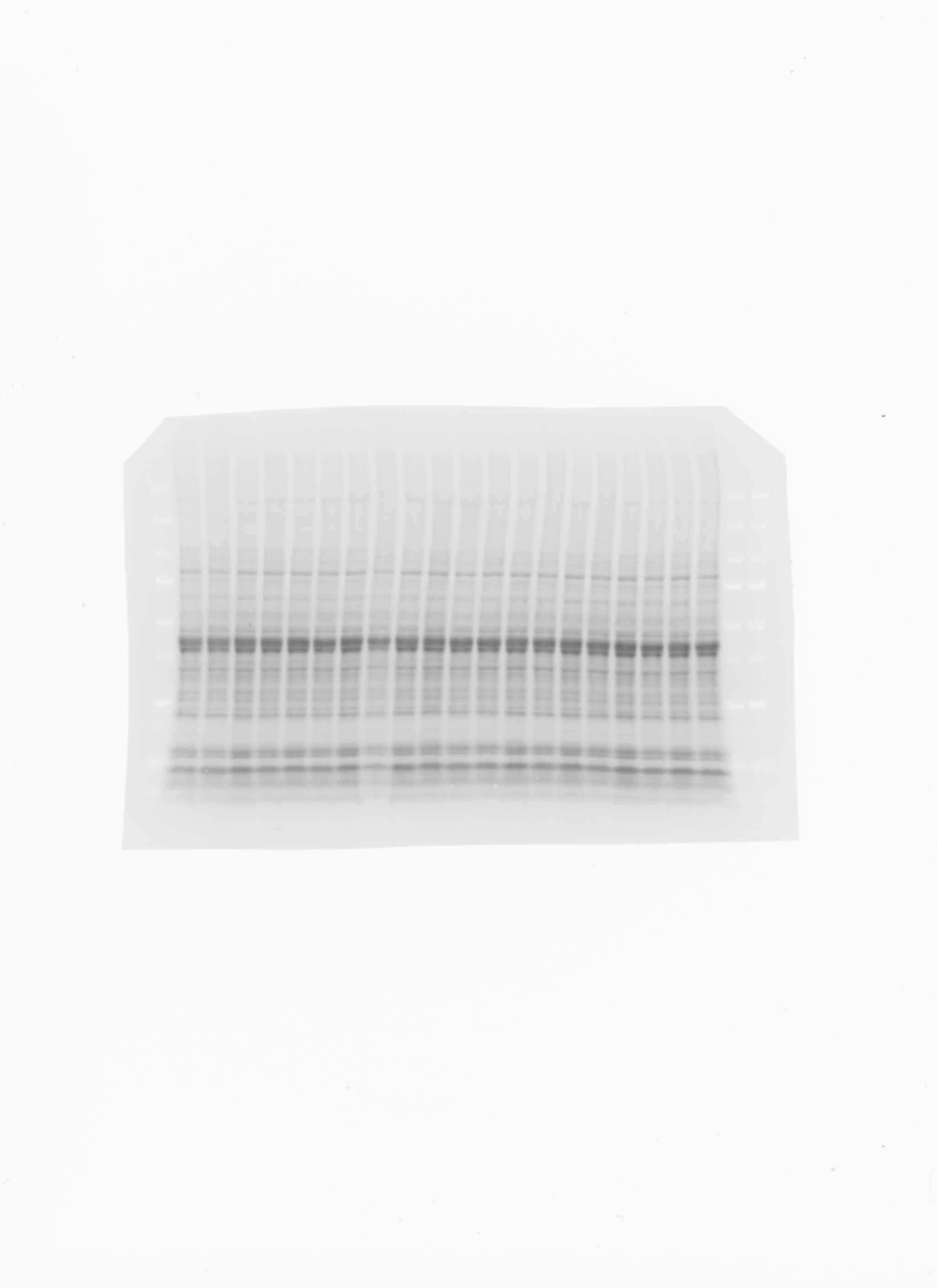

Supplement: Figure 13—figure supplement 4—source data 1. [file elife-80949-fig13-figsupp4-data1.zip › Figure 13-supplement 4 source data/CALR/Total Protein/DR TProt. LV Blot17 2018.03.20_11.52.03_Fl-UV.tif]

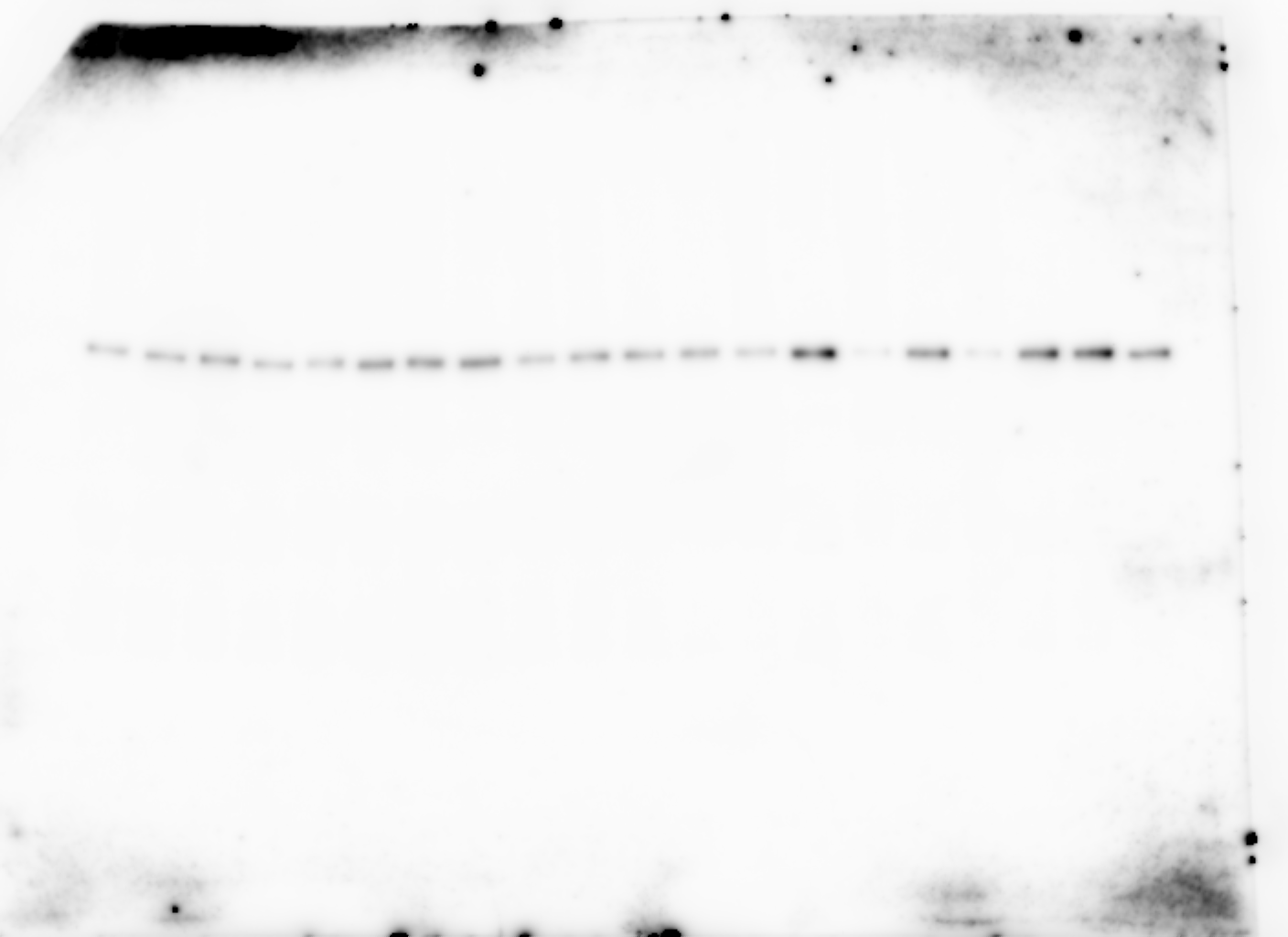

Supplement: Figure 13—figure supplement 4—source data 1. [file elife-80949-fig13-figsupp4-data1.zip › Figure 13-supplement 4 source data/CNX/CNX/DR CLNX LV blt38 2018.09.14_11.22.15_Ch.tif]

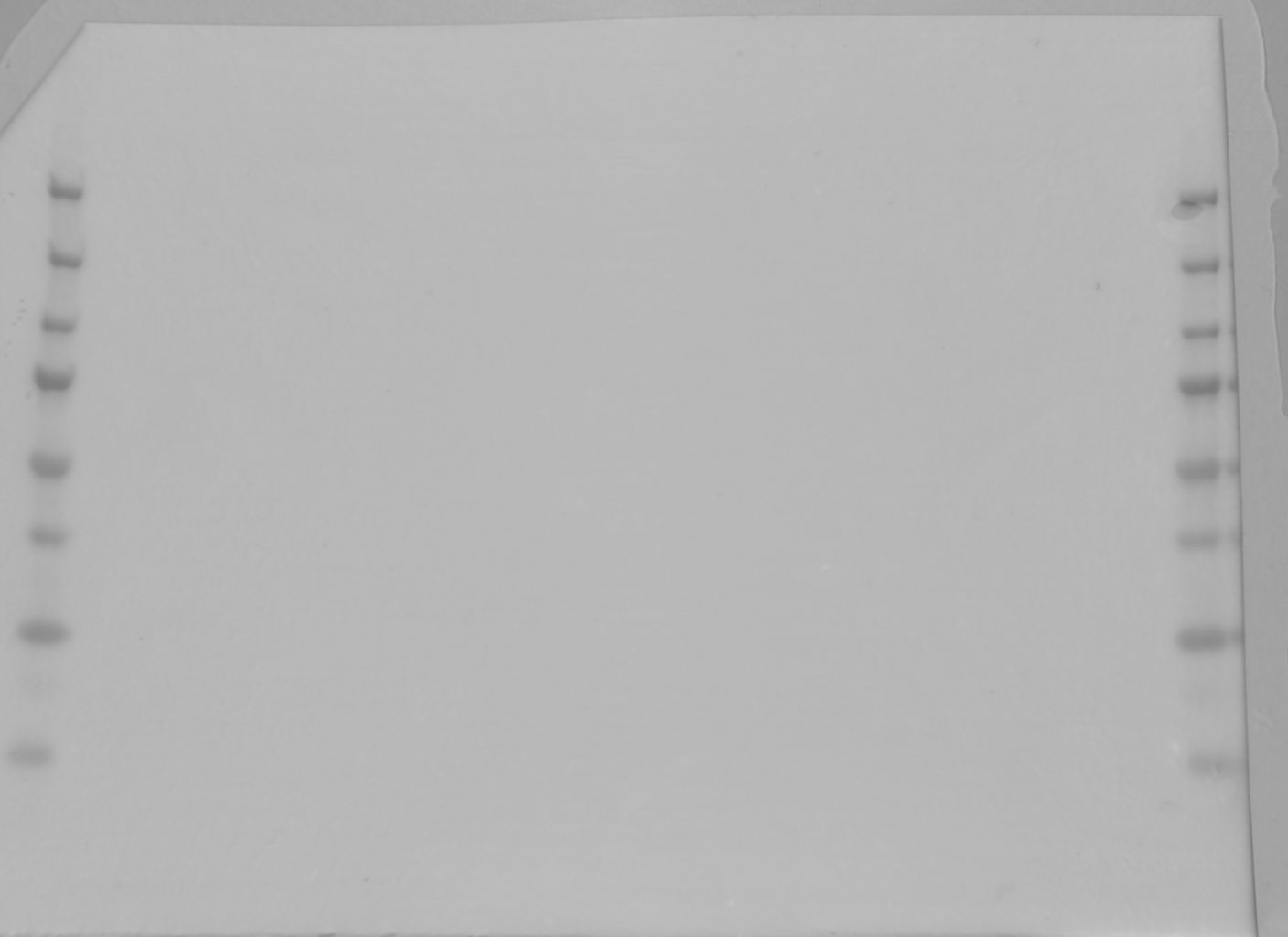

Supplement: Figure 13—figure supplement 4—source data 1. [file elife-80949-fig13-figsupp4-data1.zip › Figure 13-supplement 4 source data/CNX/CNX/DR CLNX LV blt38 2018.09.14_11.22.15_Ch-Marker.tif]

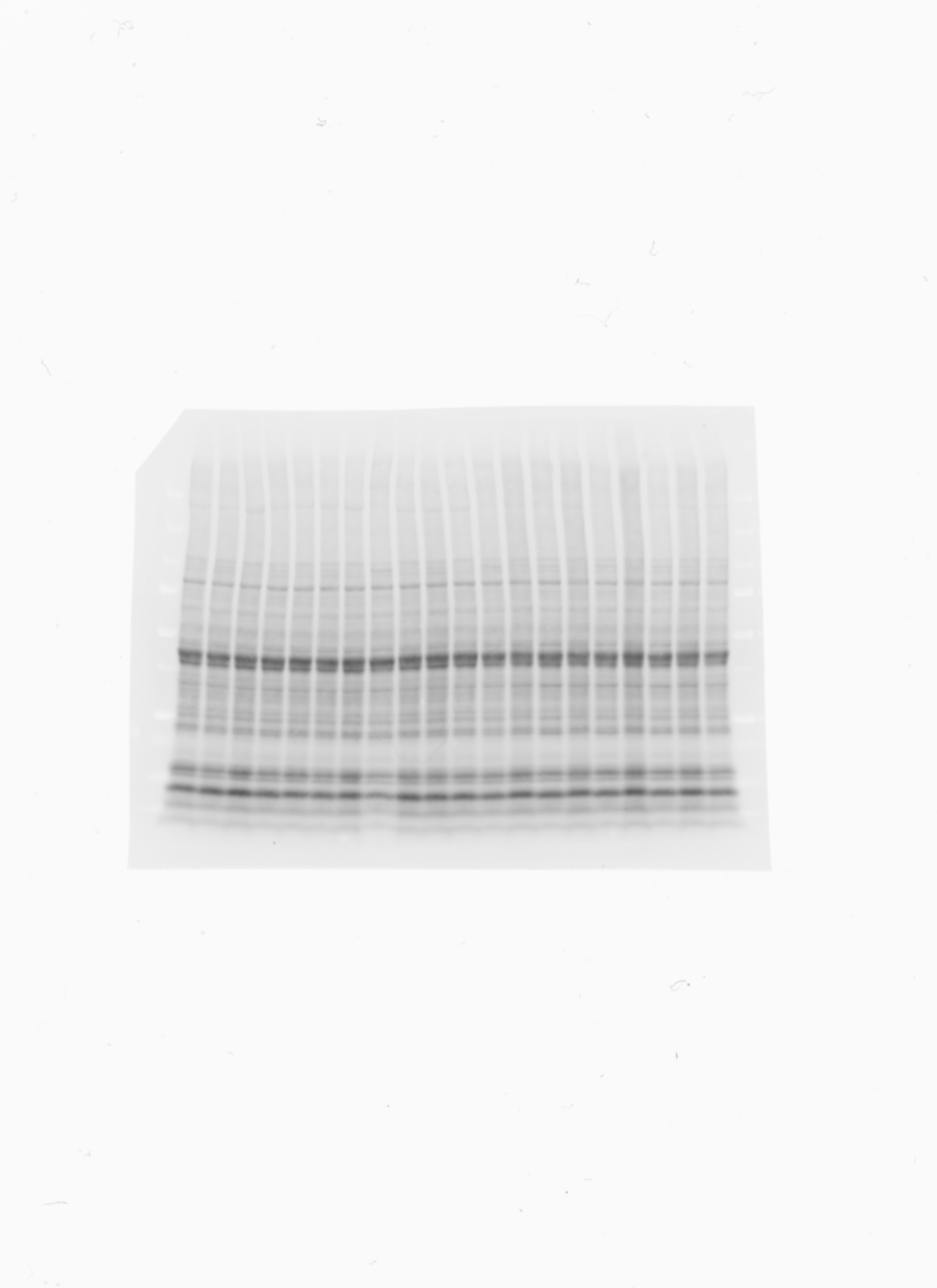

Supplement: Figure 13—figure supplement 4—source data 1. [file elife-80949-fig13-figsupp4-data1.zip › Figure 13-supplement 4 source data/CNX/Total Protein/DR TProt. LV Blot38 2018.09.10_11.54.40_Fl-UV.tif]

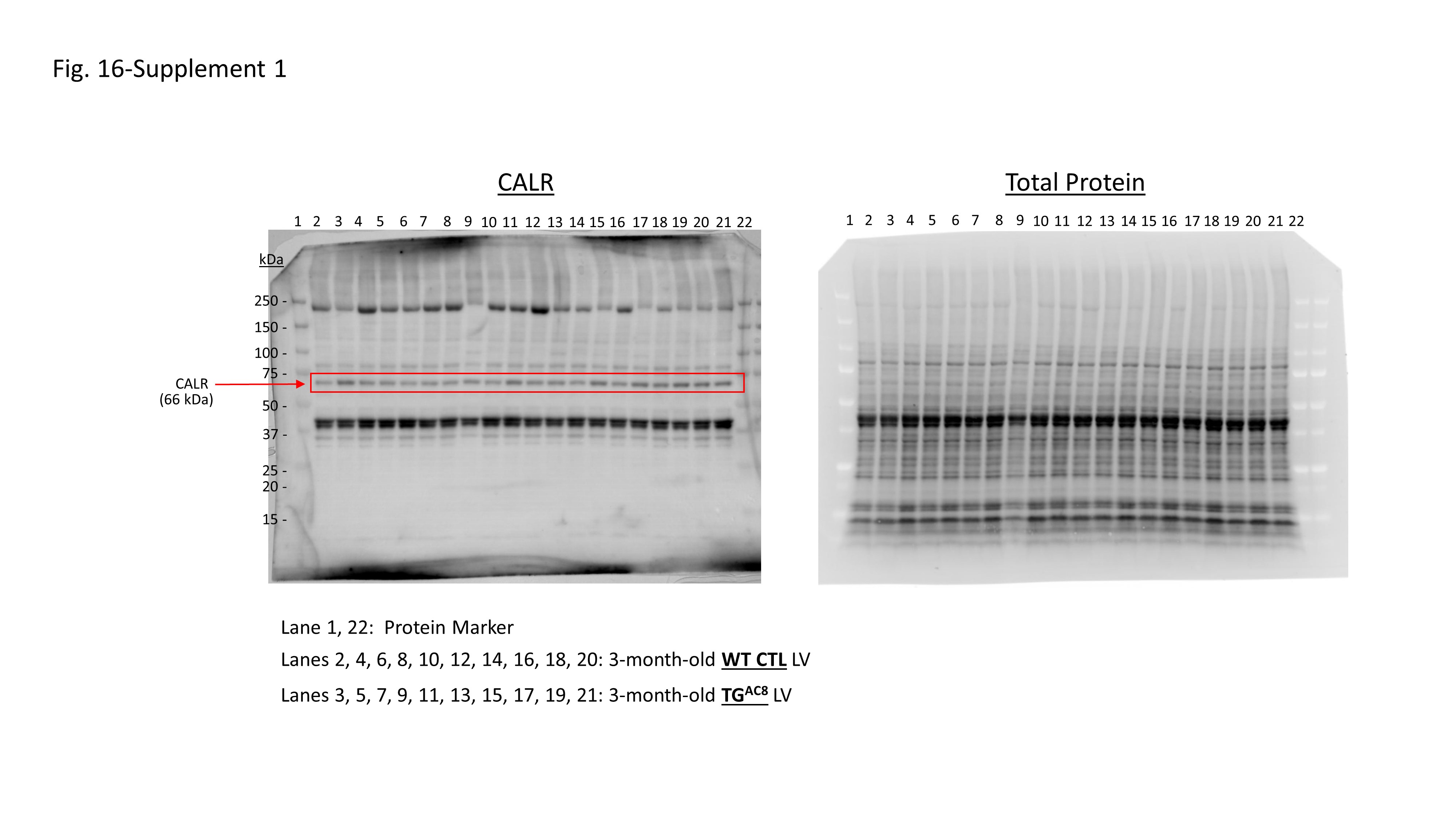

Supplement: Figure 13—figure supplement 4—source data 1. [file elife-80949-fig13-figsupp4-data1.zip › Figure 13-supplement 4 source data/Uncropped Images/CALR.JPG]

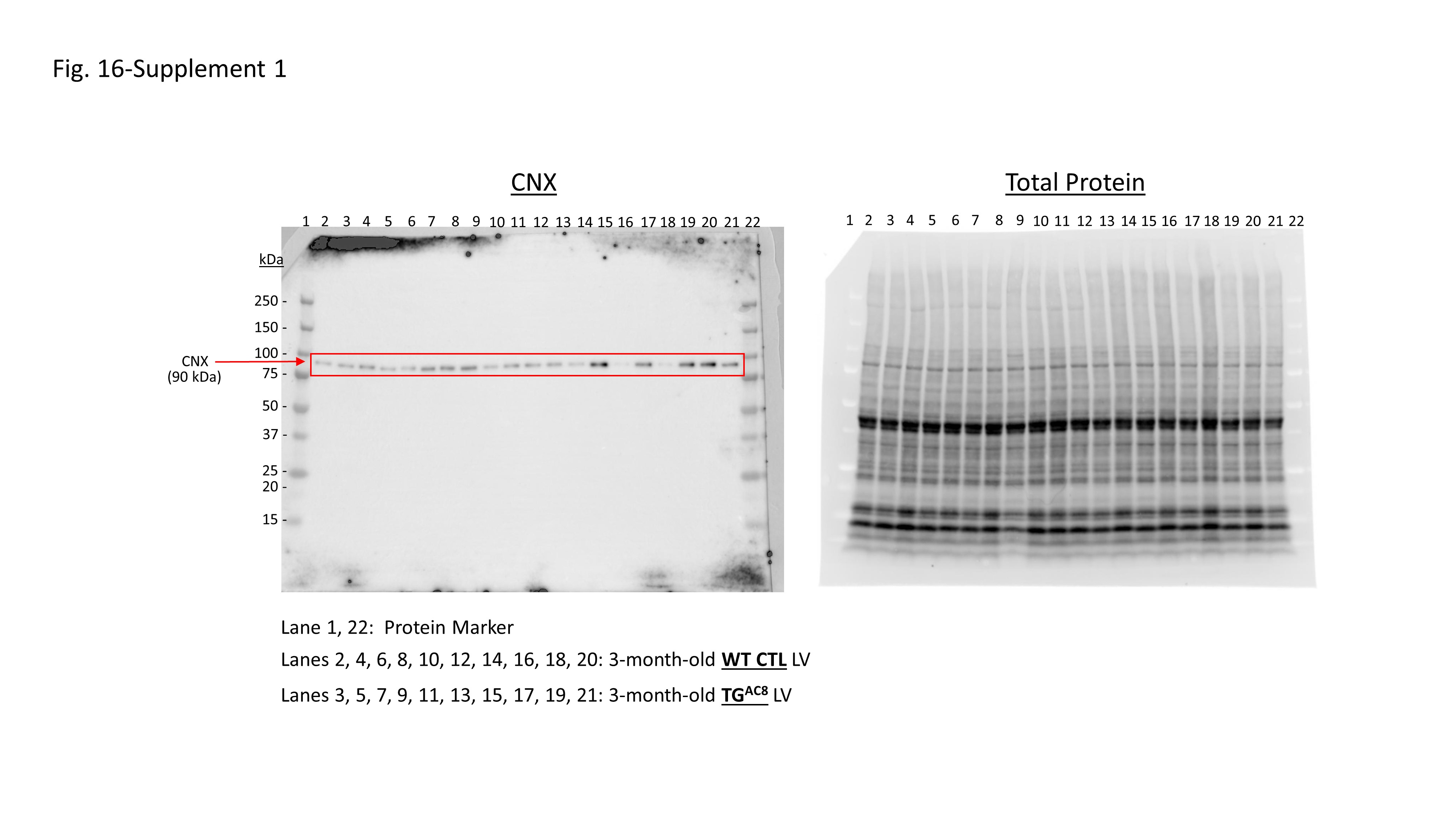

Supplement: Figure 13—figure supplement 4—source data 1. [file elife-80949-fig13-figsupp4-data1.zip › Figure 13-supplement 4 source data/Uncropped Images/CNX.JPG]
